# Biochemical Diversification through Foreign Gene Expression in Bdelloid Rotifers

**DOI:** 10.1371/journal.pgen.1003035

**Published:** 2012-11-15

**Authors:** Chiara Boschetti, Adrian Carr, Alastair Crisp, Isobel Eyres, Yuan Wang-Koh, Esther Lubzens, Timothy G. Barraclough, Gos Micklem, Alan Tunnacliffe

**Affiliations:** 1 Department of Chemical Engineering and Biotechnology, University of Cambridge, Cambridge, United Kingdom; 2 Department of Genetics, University of Cambridge, Cambridge, United Kingdom; 3 Cambridge Systems Biology Centre, Cambridge, United Kingdom; 4 Department of Life Sciences, Imperial College London, London, United Kingdom; 5 National Institute of Oceanography, Haifa, Israel; University of Michigan, United States of America

## Abstract

Bdelloid rotifers are microinvertebrates with unique characteristics: they have survived tens of millions of years without sexual reproduction; they withstand extreme desiccation by undergoing anhydrobiosis; and they tolerate very high levels of ionizing radiation. Recent evidence suggests that subtelomeric regions of the bdelloid genome contain sequences originating from other organisms by horizontal gene transfer (HGT), of which some are known to be transcribed. However, the extent to which foreign gene expression plays a role in bdelloid physiology is unknown. We address this in the first large scale analysis of the transcriptome of the bdelloid *Adineta ricciae*: cDNA libraries from hydrated and desiccated bdelloids were subjected to massively parallel sequencing and assembled transcripts compared against the UniProtKB database by blastx to identify their putative products. Of ∼29,000 matched transcripts, ∼10% were inferred from blastx matches to be horizontally acquired, mainly from eubacteria but also from fungi, protists, and algae. After allowing for possible sources of error, the rate of HGT is at least 8%–9%, a level significantly higher than other invertebrates. We verified their foreign nature by phylogenetic analysis and by demonstrating linkage of foreign genes with metazoan genes in the bdelloid genome. Approximately 80% of horizontally acquired genes expressed in bdelloids code for enzymes, and these represent 39% of enzymes in identified pathways. Many enzymes encoded by foreign genes enhance biochemistry in bdelloids compared to other metazoans, for example, by potentiating toxin degradation or generation of antioxidants and key metabolites. They also supplement, and occasionally potentially replace, existing metazoan functions. Bdelloid rotifers therefore express horizontally acquired genes on a scale unprecedented in animals, and foreign genes make a profound contribution to their metabolism. This represents a potential mechanism for ancient asexuals to adapt rapidly to changing environments and thereby persist over long evolutionary time periods in the absence of sex.

## Author Summary

Bdelloid rotifers are tiny invertebrates with unusual characteristics: they withstand stresses, such as desiccation and high levels of ionising radiation, that kill other animals, and they have survived over millions of years without sexual reproduction, which contradicts theories on the evolutionary advantages of sex. In this study, we investigate another bizarre feature of bdelloids, namely their ability to acquire genes from other organisms in a process known as horizontal gene transfer (HGT). We show that HGT happens on an unprecedented scale in bdelloids: approximately 10% of active genes are “foreign,” mostly originating from bacteria and other simple organisms like fungi and algae, but now functioning as bdelloid genes. About 80% of foreign genes code for enzymes, and these make a major contribution to bdelloid biochemistry: 39% of enzyme activities have a foreign contribution, and in 23% of cases the activity in question is uniquely specified by a foreign gene. This indicates biochemistry, such as toxin degradation and antioxidant generation, that is unknown in other animals and that is expected to improve the “robustness” of the bdelloid. It also represents a possible mechanism for survival without sex, by diversification of functional capacity and even replacement of defective genes by foreign counterparts.

## Introduction

Bdelloid rotifers (Rotifera, Bdelloidea) are abundant, ubiquitous microinvertebrates that inhabit aqueous habitats [1]. They possess an extraordinary and unique combination of characteristics among the Metazoa: they have survived for tens of millions of years without sexual reproduction, while speciating similarly to sexual organisms; they can withstand extreme desiccation by undergoing anhydrobiosis; and they display other properties usually associated with extremophiles such as tolerance of high levels of ionizing radiation [2]–[7]. In addition, the bdelloids *Adineta vaga* and *Philodina roseola* contain foreign DNA sequences in at least some subtelomeric chromosomal regions of their genomes, and these probably derive from horizontal gene transfer (HGT) [8]. Three of these genes were shown to be transcribed, and Boschetti et al. [9] showed that in a related bdelloid species, *A. ricciae*, four different foreign genes, out of a set of 36 identifiable foreign and native sequences sampled, were expressed. Of these, three were upregulated by evaporative water loss and were therefore part of the desiccation stress response.

This suggests that horizontal gene transfer (HGT) might contribute significantly to the remarkable biology of the bdelloid rotifer. However, the proportion of the bdelloid genome harbouring foreign sequences, how many of these sequences are expressed, and their contributions to bdelloid physiology, are completely unknown. To address these issues, we present the first global analysis of the transcriptome of a bdelloid rotifer, *A. ricciae*, which shows that horizontally acquired genes are expressed on a scale unprecedented in animals and that they make a profound contribution to bdelloid metabolism. We suggest this is highly significant in the context of the extremophile nature of bdelloids and their long term evolutionary persistence without sex, which theory suggests should limit their ability to adapt to changing environments [10]–[13].

## Results

### A high proportion of horizontally acquired sequences in the bdelloid transcriptome

To capture expression of genes active during the hydrated and dehydrated states, cDNA was prepared and pooled from a laboratory strain of *A. ricciae* under both conditions, then partially normalised to reduce coverage of abundant transcripts. Paired-end, massively parallel sequencing was performed on cDNA fragments of mean size 200 bp using the Illumina platform; 19.5 million 76-base reads were assembled to give an initial library of 61,219 transcript contigs of size range 118–3674 bp. Of these, 28,922 contigs gave at least one significant blast hit (E-value≤10^−5^) when compared to the UniProtKB database, allowing the identification of their likely product, and these were used for further analysis.

Those transcripts originating from horizontally acquired genes were identified by assigning each contig an HGT index, *h_U_*, defined as the difference between the “bitscore” (i.e. score in bits) of the best non-metazoan match and the bitscore of the best metazoan match in the database. The subscript, *U* here, signifies the database used, UniProtKB; *S* for Swiss-Prot is used where appropriate below. A positive *h_U_* value for a given transcript means that its translation gives a better alignment to a non-metazoan protein than to a metazoan protein, and vice versa for a negative *h_U_* value. For comparison with other invertebrates, we carried out the same analysis with transcript datasets from the monogonont rotifer *Brachionus plicatilis* (a distinct class within phylum Rotifera, that has both sexual and asexual life phases, and is not considered anhydrobiotic, but can form desiccation-tolerant resting eggs), the nematode *Caenorhabditis elegans* and the fly *Drosophila melanogaster*.

**Figure 1:**
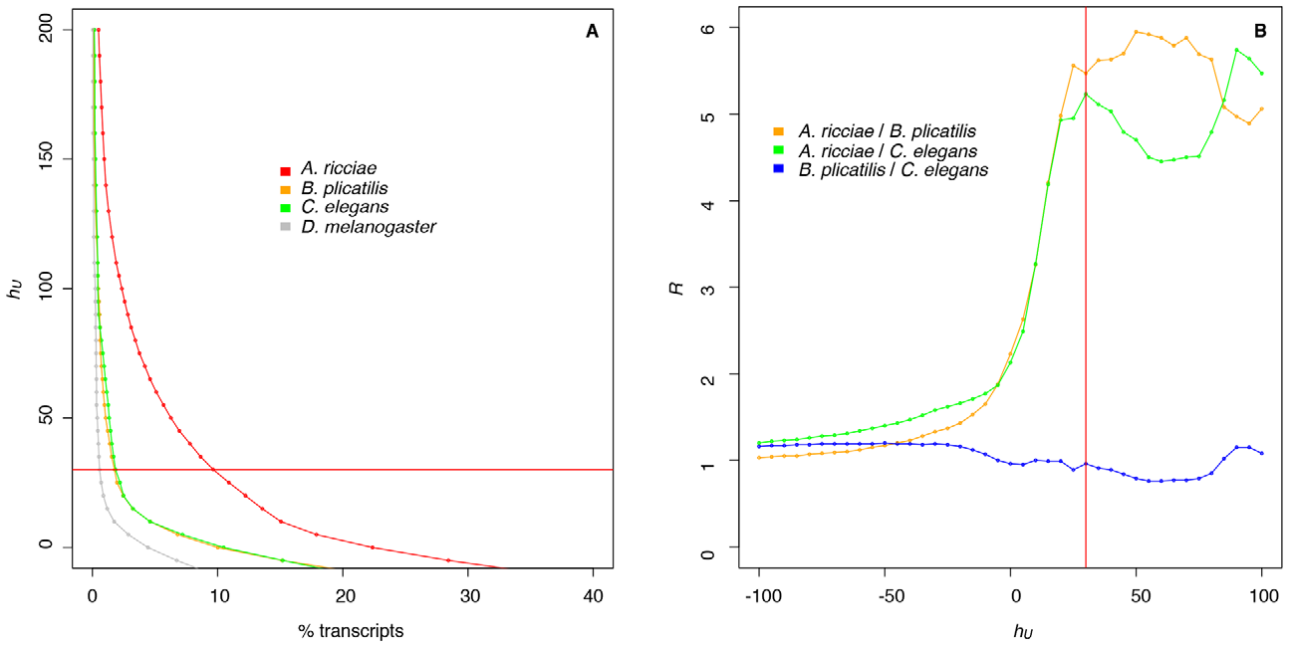
The *A. ricciae* transcriptome contains an unusually large proportion of sequences originating from other organisms. (A) Percentage of transcripts with HGT index *h_U_* above ordinate value for the bdelloid rotifer *A. ricciae* (red), the monogonont rotifer *B. plicatilis* (orange), the nematode *C. elegans* (green) and the fly *D. melanogaster* (grey), for the interval 0 to 200, with the horizontal red line at *h_U_* = 30. (B) The relative proportion, *R*, of transcripts with HGT index exceeding a given value of *h_U_*, comparing *A. ricciae* and *B. plicatilis* (orange), *A. ricciae* and *C. elegans* (green), or *B. plicatilis* and *C. elegans* (blue). The vertical red line indicates *h_U_* = 30.

Although for *h_U_*>0, a non-metazoan origin is indicated, there will be some uncertainty where non-metazoan bitscores are close to those of metazoans. Therefore, a threshold signifying foreign origin needs to be set at some value higher than zero. Figure 1A shows that the bdelloid contains many more foreign transcripts than other invertebrates, regardless of where a threshold might be set, and therefore other species can be used as a reference for ‘background’ levels of HGT in invertebrates. We calculate *R(h_U_)*, the relative proportion of transcripts with HGT index value greater than a given value of *h_U_*, where *R* = (the percentage of transcripts from species 1 with HGT index≥*h_U_*)÷(the percentage of transcripts from species 2 with HGT index≥*h_U_*). In comparisons between *A. ricciae* and other invertebrate species, we notice that, for *h_U_*≤0, *R* is relatively constant since both metazoan and non-metazoan sequences are included. However, as the *h_U_* = 0 threshold is passed, *R* increases with *h_U_* as metazoan sequences are excluded, and the greater proportion of foreign sequences in the bdelloid transcriptome becomes apparent. *R* then plateaus around *h_U_* = 25–30 and is approximately constant up to *h_U_*∼100 (Figure 1B). This suggests that, as the threshold of *h_U_* = 30 is exceeded, the proportion of sequences judged to be foreign decreases, but at a similar rate in both the bdelloid and the comparator species, i.e. the ratio between species remains constant, indicating that increasing stringency above *h_U_* = 30 only results in loss of truly foreign genes from the count, and does not give a better test of “foreignness”. Figure 1B also shows that there is approximately 5-fold more HGT in *A. ricciae* than in either *B. plicatilis* or *C. elegans*, since *R*≈5 for *h_U_*≥30. For the comparison of *A. ricciae* and *D. melanogaster*, the ratio is appreciably higher at *R*≈16 (data not shown), in line with the apparently very low levels of HGT in the fly (Figure 1A). A comparison of *B. plicatilis* with *C. elegans* (Figure 1B) does not show the inflection between *h_U_* = 0 and *h_U_* = 30, consistent with these species having a similar proportion of foreign sequences in their transcriptomes. We used linear models to test whether differences in *h_U_* were significant among taxa. Results confirmed that *A. ricciae* had both a significantly higher mean *h_U_* score and a significantly higher probability per gene of *h_U_*>30 than the other taxa, even when controlling for differences in contig length between the assemblies (all comparisons, *p*<0.001, details in legend to Figure S1).

Of the identified bdelloid contigs, 9.7% (2,792/28,922) were shown to have *h_U_*≥30 and so were considered to be of foreign origin (Figure 1A, Table S1). In *B. plicatilis*, 1.8% (171/9,685) of transcripts have *h_U_*≥30, while in *C. elegans* and *D. melanogaster* this figure is 1.8% (206/11,168) and 0.6% (105/18,368), respectively (Figure 1A). This demonstrates that, independent of the dataset dimensions, the level of expressed HGT in bdelloid rotifers is far greater than in other invertebrates tested.

**Figure 2:**
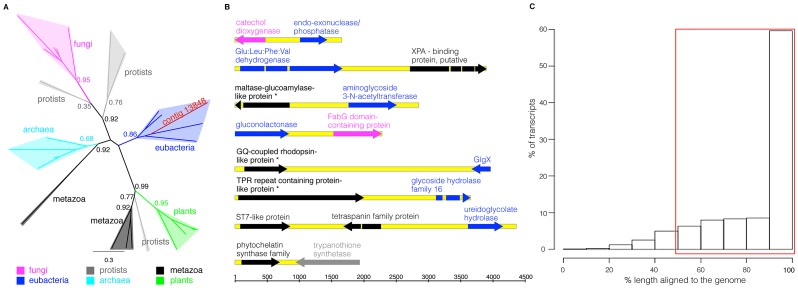
Foreign genes in the *A. ricciae* genome. (A) Phylogenetic tree for one exemplar bdelloid transcript (contig 13848) encoding an acetyl-CoA synthetase. Branch colours represent different taxa: metazoa, black; eubacteria, blue; archaea, light blue; fungi, pink; protists, grey; plants, green. Numbers on nodes represent aLRT support. (B) Physical linkage of foreign genes to neighbouring genes in the genome: eight different Sanger sequenced and assembled genomic regions, with arrows showing gene length and orientation (metazoa, black; eubacteria, blue; fungi, pink; protists, grey); introns are indicated as interruptions. Bdelloid genes previously identified in *A. vaga* are marked with an asterisk. In both the first and fourth genomic regions shown, the two foreign genes belong to different taxa (fungi and bacteria). Scale, bp. See also Figure S2 and Table S1. (C) Genomic coverage of *A. ricciae* foreign transcripts. Histogram of the percentage length aligned to the draft genome for all foreign transcripts. The red box indicates all foreign transcripts which align to the draft genome along greater than 50% of their length.

Phylogenetics was used to validate the foreign origins of putative horizontally acquired sequences [14] and this can be performed meaningfully where contigs with *h_U_*≥30 have a significant (E-value≤10^−5^) blast match to at least one metazoan sequence, allowing a phylogenetic tree to be constructed. However, two-thirds (1,884/2,792; 67%) of sequences with *h_U_*≥30 do not give a significant metazoan match, which strongly supports a foreign origin. For the remaining (908/2,792) contigs, phylogenetic trees were built in PhyML from amino-acids sequences using a JTT model [15]. Each contig was assigned to a particular group according to the aLRT support for each metazoan or non-metazoan taxon as follows: group 1 contains sequences that are monophyletic with Metazoa (or where there were only metazoan hits from the blast analysis); group 2 contains sequences for which monophyly with Metazoa cannot be strongly rejected; group 3 contains cases where there are too few sequences to define a meaningful clade; group 4 contains cases where monophyly with Metazoa can be strongly rejected; group 5 contains transcripts which are monophyletic with another single (non-metazoan) taxon. Analysis of these data showed that 98% of *A. ricciae* transcripts with *h_U_*≥30 and at least one significant metazoan hit fall into groups 4 and 5 with high node support (summarised in Table 1; Table S1; Figure S2) and therefore are supported as truly non-metazoan. For example, an acetyl-CoA synthetase (Enzyme Commission [EC] number 6.2.1.1) does not cluster with metazoan sequences for this enzyme, instead grouping within the eubacterial clade with high support (aLRT support = 0.86) (Figure 2A; Figure S2C). More than half of foreign transcripts appeared prokaryotic (59% eubacterial, 1% archaeal); the remainder were eukaryotic in origin: 23% fungal, 6% from algae or plants, and 11% from other eukaryotic taxa (largely protists).

**Table 1:** Summary of phylogenetic assignments for *A. ricciae* and *C. elegans*.

		*Adineta ricciae*		*C. elegans*	
phylogenetic group	explanation	transcripts	%	transcripts	%
1	sequences monophyletic with Metazoa (or where there were only metazoan hits from the blast analysis)	20	2.2	8	8.2
2	sequences for which monophyly with Metazoa cannot be strongly rejected	0	0.0	0	0.0
3	sequences where there are too few sequences to define a meaningful clade	1	0.1	0	0.0
4	sequences where monophyly with Metazoa can be strongly rejected	513	56.5	65	66.3
5	sequences monophyletic with another single (non-metazoan) taxon	374	41.2	25	25.5
	total	908	100.0	98	100.0

A similar analysis can be performed for other invertebrates. For example, there are 206 transcripts from *C. elegans* with *h_U_*≥30 of which 108 give significant blast matches only with non-metazoan sequences. For the remaining transcripts, phylogenetic analysis shows that 92% (90/98) fail to cluster with metazoan examples (summarised in Table 1; Table S2; Figure S3). Therefore, 96% (198/206) of these *C. elegans* transcripts were verified as foreign by the phylogenetics. Although there are no comprehensive studies in the literature, the frequency of HGT we detect in *C. elegans* is higher than inferred in an earlier study [16]. One possible confounding factor might be that the phylogenetic placement of individual *C. elegans* sequences is impaired by filtering out other nematode sequences (see Materials and Methods). To check this, we repeated the evaluation including the top blast hits from nematodes, i.e. homologous and paralogous examples (Table S2; Figure S3). From the phylogenetics, we found that 93% (91/98) of *C. elegans* sequences did not cluster with the metazoa and therefore 97% (199/206) of the total set of transcripts with *h_U_*≥30 are likely to be foreign. This shows that the vast majority still lack a close non-nematode metazoan match when additional nematode sequences are included in the analysis. We interpret this finding as evidence of HGT in an ancestor of nematode species in the sample. However, as our aim here is not to evaluate levels of HGT in other metazoa beyond providing a baseline for comparison with bdelloids, these analyses are meant to illustrate that the results are robust to variations in the method, such as which sequences are included for evaluation.

**Figure 3:**
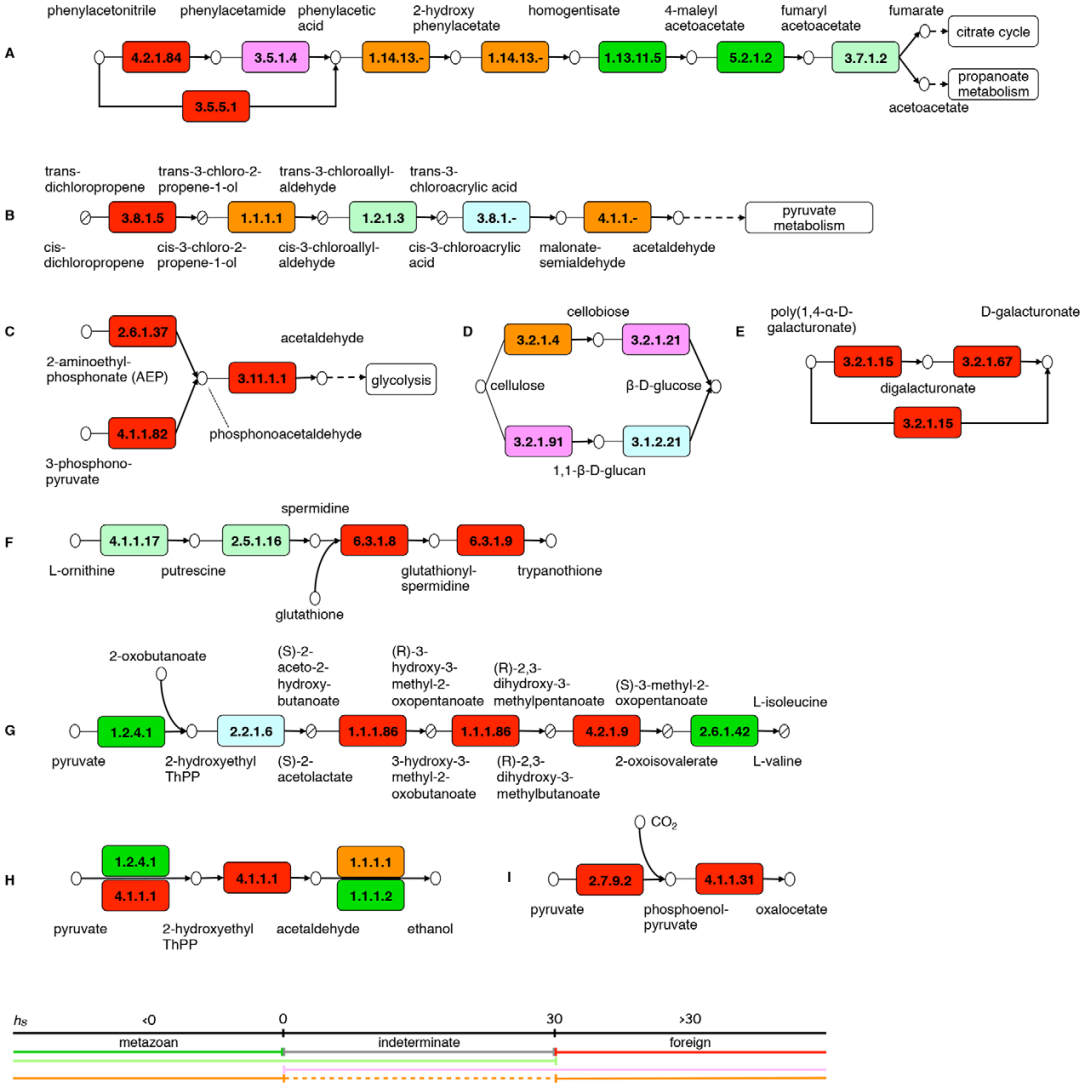
Examples of biochemical diversification encoded by the bdelloid transcriptome. Extracts of KEGG pathways where enzyme EC numbers are shown inside boxes, with the following colour scheme: green, metazoan; red, foreign; orange, both metazoan and foreign examples identified; pink, both foreign and indeterminate examples; light green, both metazoan and indeterminate examples; blue, not found in transcriptome. (A) Degradation of phenylacetonitrile (K00643); (B) degradation of 1,3-dichloropropene (K00625); metabolism of (C) AEP (2-aminoethylphosphonate) and 3-phosphono-pyruvate (K00440), (D) cellulose (K00500) and (E) polygalacturonate (K00040); (F) biosynthesis of trypanothione (K00480); (G) completion of biosynthetic pathways for valine and isoleucine (K00290); (H) generation of ethanol from pyruvate (K00010); (I) carbon fixation by phosphoenolpyruvate carboxylase (K00720). See also Figure S5, Table 2 and Table S4.

To confirm that foreign transcripts originated from the bdelloid genome and were not due to contaminating or commensal organisms, several corresponding genomic regions were PCR-amplified and Sanger sequenced, and this showed that foreign genes were linked to a gene of metazoan origin or to another foreign gene from a different taxon (Figure 2B). In some cases (asterisks in Figure 2B), the foreign transcript was close to a gene previously described in a bdelloid rotifer. The sequences were also aligned to an early draft of the *A. ricciae* genome, where 91% of foreign transcripts aligned for at least 50% of their length, compared to 90% of all transcripts and for metazoan transcripts only (Figure 2C; data not shown). Furthermore, 81% of foreign transcripts were aligned to the same genomic contig as metazoan transcripts or foreign transcripts of a different phylogenetic group, which rules out an origin from contamination for this set (examples given in Table S3 correspond to some foreign sequences in Figure 3). This proportion is likely to rise as genome assemblies improve, but even if 10–20% of foreign genes cannot be shown to be part of the bdelloid genome, and thus represent contamination, this would only reduce the foreign component of the transcriptome to 8–9%, rather than 10%, which is still remarkably high.

**Table 2:** KEGG pathways containing enzymes encoded by transcripts of foreign origin.

KEGG pathway	EC # represented by alien transcripts	total EC #
ec00230 Purine metabolism	28	58
ec00520 Amino sugar and nucleotide sugar metabolism	23	47
**ec00500 Starch and sucrose metabolism**	**22**	**34**
ec00330 Arginine and proline metabolism	22	43
ec00380 Tryptophan metabolism	19	36
ec00260 Glycine, serine and threonine metabolism	15	35
ec00627 Aminobenzoate degradation	14	20
ec00680 Methane metabolism	14	26
ec00240 Pyrimidine metabolism	14	37
ec00051 Fructose and mannose metabolism	13	27
ec00540 Lipopolysaccharide biosynthesis	12	12
ec00910 Nitrogen metabolism	12	19
ec00524 Butirosin and neomycin biosynthesis	11	12
ec00360 Phenylalanine metabolism	11	20
ec00363 Bisphenol degradation	10	10
ec00906 Carotenoid biosynthesis	10	10
ec00626 Naphthalene degradation	10	12
ec00052 Galactose metabolism	10	18
ec00620 Pyruvate metabolism	10	24
ec00362 Benzoate degradation	10	15
ec00350 Tyrosine metabolism	10	26
**ec00010 Glycolysis/Gluconeogenesis**	**10**	**27**
ec00940 Phenylpropanoid biosynthesis	9	10
ec01040 Biosynthesis of unsaturated fatty acids	9	14
ec00624 Polycyclic aromatic hydrocarbon degradation	9	12
ec00053 Ascorbate and aldarate metabolism	9	15
ec00100 Steroid biosynthesis	9	15
ec00600 Sphingolipid metabolism	9	22
ec00564 Glycerophospholipid metabolism	9	32
**ec00720 Carbon fixation pathways in prokaryotes**	**9**	**26**
**ec00625 Chloroalkane and chloroalkene degradation**	**8**	**9**
ec01057 Biosynthesis of type II polyketide products	8	9
ec00760 Nicotinate and nicotinamide metabolism	8	20
ec00561 Glycerolipid metabolism	8	21
ec00270 Cysteine and methionine metabolism	8	25
ec00130 Ubiquinone and other terpenoid-quinone biosynthesis	8	12
ec00950 Isoquinoline alkaloid biosynthesis	8	15
ec00361 Chlorocyclohexane and chlorobenzene degradation	7	8
ec00460 Cyanoamino acid metabolism	7	9
ec00730 Thiamine metabolism	7	9
ec00903 Limonene and pinene degradation	7	14
ec00340 Histidine metabolism	7	16
ec00250 Alanine, aspartate and glutamate metabolism	7	20
ec00983 Drug metabolism - other enzymes	7	19
**ec00480 Glutathione metabolism**	**7**	**22**
ec00630 Glyoxylate and dicarboxylate metabolism	7	22
ec00945 Stilbenoid, diarylheptanoid and gingerol biosynthesis	6	6
ec00623 Toluene degradation	6	8
ec00062 Fatty acid elongation	6	11
ec00061 Fatty acid biosynthesis	6	13
**ec00040 Pentose and glucuronate interconversions**	**6**	**14**
ec00650 Butanoate metabolism	6	22
ec00562 Inositol phosphate metabolism	6	28
ec00860 Porphyrin and chlorophyll metabolism	6	21

KEGG pathways and identification number, number of transcripts identified as foreign (red+pink+orange in Figure 3), and total number of matched EC numbers for each pathway are shown for the most frequently populated pathways. Pathways shown in Figure 3 are highlighted in bold. See also Figure S5 and Table S4 (full version of Table 2).

### The majority of foreign transcripts encode enzymes

Where HGT has been observed between prokaryotes, operational genes encoding, for example, enzymes, predominate over informational genes concerned with transcription and translation [17], [18]. If a similar situation pertains in bdelloids, we would expect to find many foreign genes that encode enzymes, which largely fall into the operational category [18]. Bdelloid transcripts with biochemical functions were identified by alignment to proteins with EC numbers in the Swiss-Prot database. This database was used as the quality of annotation is higher than UniProtKB and the smaller number of proteins should reduce the false positive rate (although it will also increase the number of false negatives). Of the 26,001 transcript contigs with matches in the Swiss-Prot database, 2,947 (11.3%) had *h_S_*≥30 and were categorised as foreign, i.e. a similar proportion to the previous analysis using UniProtKB (Figure S4).

Approximately 50% (13,059/26,001) of contigs (irrespective of their *h_S_* value) had a match with an assigned EC number. These were then tagged as either metazoan (*h_S_*≤0), indeterminate (0<*h_S_*<30) or foreign (*h_S_*≥30). Therefore, of the foreign transcripts, 79% (2,341/2,947) were annotated with an EC number, showing that a large majority are concerned with metabolism. In fact, when the functions of those without an EC number were analysed, a further 93 sequences could be identified as enzymes that lacked EC numbers due to poor annotation. This increases the proportion of foreign transcripts encoding metabolic functions to 83% (Figure S4).

Transcript contigs (in all categories) with assigned EC numbers were mapped onto the Kyoto Encyclopedia of Genes and Genomes (KEGG) reference pathways (denoted ‘K’ plus a number in the following). In total, 839 EC numbers assigned to the rotifer transcriptome were matched to 152 metabolic pathways (Table 2 and Table S4). Of the 839 EC numbers, 23% (191) were only assigned to foreign transcripts, with a further 16% (138) being assigned to both foreign and metazoan transcripts. This made a total of 39% of identified enzyme activities with a contribution from foreign transcripts, suggesting that HGT has the potential to radically diversify bdelloid biochemistry.

### Foreign transcripts encode biochemical functions unknown in metazoans

Many pathways containing foreign transcript products specify metabolism found only in micro-organisms and unknown in metazoans (Figure 3, Figure S5, Table 2, Table S4). Several of these are concerned with degradation of toxic compounds, and we give three examples here: 1) breakdown of phenylacetonitrile (benzyl cyanide) is initiated by the products of two genes derived from bacteria (EC 4.2.1.84 or EC 3.5.5.1; K00643; Figure 3A, Figure S5A), and other nitrile compounds, such as benzonitrile, can also be metabolised similarly (K00627, Figure S5B); 2) the organochloride pesticide, 1,3-dichloropropene, is degraded in five steps to the central metabolite, acetaldehyde, and the first of these is exclusively specified by the foreign-encoded enzyme haloalkane dehalogenase (EC 3.8.1.5; K00625; Figure 3B, Figure S5C); 3) branches of the degradation pathways for benzoate (K00362) and bisphenol (K00363) are also represented by foreign gene products (Figure S5D, S5E). Not all steps in these pathways are present in our transcriptome sample. This is partially due to the use of the Swiss-Prot database to assign EC numbers; performing the same analysis using the UniProtKB database adds steps to many pathways. However, there might also be incomplete capture of transcripts during cDNA cloning and sequencing, or bdelloids might only partially metabolise certain compounds. If the latter is correct, such partial metabolism might still be sufficient for detoxification or metabolite utilisation in other pathways.

HGT is also implicated in improved resource acquisition, e.g. two-step pathways to convert the ubiquitous natural phosphonates 2-aminoethylphosphonate (AEP) and 3-phosphonopyruvate into useable metabolites are enabled by foreign transcripts encoding 2-aminoethylphosphonate-pyruvate transaminase (EC 2.6.1.37) or phosphonopyruvate decarboxylase (EC 4.1.1.82) and phosphonoacetaldehyde hydrolase (EC 3.11.1.1) (K00440; Figure 3C, Figure S5F). Furthermore, several foreign transcripts are implicated in utilisation of a range of polysaccharides not normally directly available to metazoans, e.g. cellulose (K00500; Figure 3D, Figure S5G) and polygalacturonate (K00040; Figure 3E, Figure S5H) breakdown; α-N-arabinofuranosidase (EC 3.2.1.55; K00520, Figure S5I), glucan endo-1,3-β-glucosidase (EC 3.2.1.39; K00500, Figure S5G) and fructan β-fructosidase (EC 3.2.1.80; K00051, Figure S5J) are also encoded. Cellulase activity has been described in other invertebrates but, where this does occur, it seems to result from HGT (e.g. ref. [19]).

Other pathways novel to metazoans but represented in the bdelloid transcriptome are biosynthetic, some of which are associated with robustness. These include production of the powerful antioxidant, trypanothione, normally only produced by parasitic protozoa, which is specified by two foreign transcripts: a glutathionylspermidine synthetase (EC 6.3.1.8), and a trypanothione synthase (EC 6.3.1.9; K00480; Figure 3F, Figure S5K). Such antioxidants could play a role in desiccation tolerance, where protection of repair systems against oxidative stress is thought to be crucial [20]–[22]. Foreign gene products can also add extensions or linking steps to existing metazoan metabolism in *A. ricciae*. Valine and isoleucine are essential amino acids in animals and must normally be accumulated from the diet. However, foreign transcripts encode ketol-acid reductoisomerase (EC 1.1.1.86) and dihydroxy-acid dehydratase (EC 4.2.1.9), allowing completion of biosynthetic routes to these amino acids from pyruvate (K00290; Figure 3G, Figure S5L). *A. ricciae* also encodes a fungal form of pyruvate decarboxylase (EC 4.1.1.1; K00010), allowing an additional end step to glycolysis for the regeneration of NAD^+^ from NADH under anaerobic conditions with the production of ethanol (Figure 3H, Figure S5M); animals usually only form lactate from pyruvate under these conditions. A further intriguing possibility highlighted by the transcriptome analysis is that the bdelloid can fix carbon from CO_2_, using eubacterial forms of phosphoenolpyruvate synthase (EC 2.7.9.2) and phosphoenolpyruvate carboxylase (EC 4.1.1.31; K00720; Figure 3I, Figure S5N), by a route used in plants and bacteria, but not in fungi or animals. Where it is meaningful to do so, i.e. where there are significant metazoan blast matches, phylogenetic trees are shown in Figure S2G–S2M for example transcript contigs representing foreign-encoded activities in Figure 3.

In a few cases, where we would expect to find a metazoan sequence, this is absent from the transcriptome and the activity is instead represented by a foreign counterpart. For instance, a fungal form of stearoyl-CoA delta-9 desaturase (EC 1.14.19.1; K01040; Figure S5O), an essential enzyme for the synthesis of monounsaturated fatty acids, is present, but no metazoan equivalent was discovered in the transcriptome. To control for the possibility that relevant metazoan genes had not been expressed in study samples, we searched a draft *A. ricciae* genome sequence, but failed to find them, although the gene encoding the foreign transcript was present. While the inability to detect a particular sequence is not proof of its absence, this suggests that the metazoan form of stearoyl-CoA delta-9 desaturase has been lost in the bdelloid, perhaps following a detrimental mutation, and that a foreign gene has been co-opted in this role. Other examples of a foreign sequence potentially replacing a metazoan counterpart include nicotinate-nucleotide diphosphorylase (EC 2.4.2.19; K00760), which catalyses a step in NAD^+^ biosynthesis, and the antioxidant peptide-methionine (S)-S-oxide reductase (EC 1.8.4.11).

## Discussion

In recent years, there has been increasing interest in HGT, but most investigations have been performed in prokaryotes or in unicellular eukaryotes. In these organisms, HGT is considered a main driver of innovation, often associated with speciation [23], [24]. In multicellular eukaryotes, there has been less emphasis on HGT, partly because it is thought to occur on a much smaller scale [14], [25], and partly because there are fewer well-annotated genome sequences available. Since *de novo* whole genome assembly is still a significant challenge for complex organisms, particularly for the bdelloid rotifer with its unusual genome characterised by degenerate tetraploidy, divergence of formerly allelic sequences, and gene conversion between gene copies [7], [26], [27], we chose to assess HGT primarily at the transcriptome level. This study represents the first global analysis of the expressed genes in a bdelloid rotifer, *A. ricciae*, and the contribution of horizontally acquired sequences to its transcriptome. The results reveal a remarkable degree of HGT in the bdelloid, with approximately 10% of identifiable, transcribed sequences deriving mainly from prokaryotes, but also from fungi, plants and algae, and protists.

The method for assessing HGT in the bdelloid transcriptome is novel, but follows principles currently recognised as the most rigorous, where sequence matching is coupled with phylogenetics [14]. There have been relatively few such global analyses among the Metazoa that test for expression of horizontally acquired sequences, one example being in *Hydra magnipapillata*, where seventy-one “gene models” apparently derive from bacteria, 70% of which were shown to be transcribed [28]. For the bdelloid work, we introduced the HGT index, *h*, which is calculated as the difference in bitscores between best non-metazoan and best metazoan matches in blast alignments, to give a measure of the “foreignness” of any sequence. We preferred the HGT index to the alien index (AI), developed previously for assessing foreign sequences in bdelloid subtelomeric regions [8] and also used in the *Hydra* study [28], because *h* is calculated from bitscores and is therefore not influenced by the sizes of the databases used to perform the blast screen. In contrast, if E-values are used, as for the AI, the score changes with database size. Additionally, an arbitrary constant must be included in the AI formula so that the index does not become infinite with identical matches to database sequences; this adjustment is unnecessary with the HGT index. Although Figure 1A showed that, whatever value of *h* is chosen, there is a greater proportion of foreign sequences in the bdelloid than in other invertebrates, it is useful to adopt a threshold value to signify a foreign sequence. In principle, any sequence with *h*>0 is more likely be foreign, but there will be uncertainty at values close to zero where non-metazoan and metazoan sequences have similar degrees of divergence from the test sequence. One technique for identifying a reliable threshold value of *h* is to normalise the proportion of foreign sequences against the “background” levels found in other invertebrates. The greater proportion of horizontally acquired sequences in the bdelloid then becomes apparent above the minimum threshold level of *h* required to confidently identify their foreign nature, as shown in Figure 1B. This was validated by phylogenetics, where possible (i.e. where matching metazoan counterparts exist), which showed that the vast majority of bdelloid transcript contigs with *h_U_*≥30 did not cluster with metazoan sequences.

There are other technical considerations in any assessment of HGT. For example, we classified sequences as either metazoan or non-metazoan, and therefore any HGT from other animals (including other bdelloids) into the *A. ricciae* genome would be missed. Of course, there is no reason to believe that bdelloids are unable to acquire genes from other metazoans, or indeed from other rotifers; in fact, this might be more efficient than acquisition from microorganisms, since fewer changes to metazoan genes should be required before they become expression competent. Therefore, our approach is likely to give a minimum estimate of the extent of HGT in the bdelloid. Another factor that might influence this estimate is the approximately half of transcript contigs that showed no match with known sequences and therefore had to be excluded from further analysis. If all these sequences originate from vertical transmission into the bdelloid lineage, then this would reduce the estimate of HGT. However, there is no *a priori* reason to assume this: the proportion of foreign sequences in this non-matched set could be higher, lower or about the same as in the matched set. How the matched and non-matched sequence sets are defined could also potentially influence the proportion defined as HGT. We used 10^−5^ as a maximum value for a significant match when blast screening the transcript contigs against the databases and this gave 28,922 contigs in the matched set. If 10^−10^ or 10^−15^ is used as a cut-off value, the number of matched contigs decreases to 22,719 and 17865, respectively, but the fraction scored as foreign (i.e. with *h_U_*≥30) remains high, at 11.5% and 11.7% of matched sequences, respectively. Which database is used for blast matching also does not seem to be a major factor since both UniProtKB and Swiss-Prot gave similar proportions of foreign transcripts at 9.7% and 11.3%, respectively.

A final technical consideration might be to ask whether the HGT resulting from the endosymbiosis of the mitochondrial precursor affects our results. Endosymbiosis was a primordial event for eukaryotes, with acquisition of mitochondrial precursors taking place in the earliest eukaryotic cells, perhaps two billion years ago [29]. The horizontal gene transfer we describe is very unlikely to have occurred before the divergence of bdelloids from monogonont rotifers (or *B. plicatilis* would share similarly high levels of foreign genes), and therefore probably took place at most 100, more likely 65–80, million years ago [30]. If horizontal gene transfer has continued throughout bdelloid evolution, many events will be more recent. Consequently, most, perhaps all, gene flux from mitochondrial precursor to nucleus would have occurred before bdelloids arose. Thus, we would not expect significant differences in numbers of nuclear mitochondrial genes between bdelloids and the other major class of rotifers, the monogononts, as exemplified by *B. plicatilis* in our study. To test what proportion of foreign genes apparently derive from mitochondrial nuclear genes, we blast aligned sequences of 1,098 known nuclear mitochondrial genes from MitoCarta (www.broadinstitute.org/pubs/MitoCarta) against our transcripts. Using a cut-off of 10^−5^, only 0.7% of transcripts of foreign origin (*h_U_*≥30) matched mitochondrial nuclear genes, whereas 2.9% of those of metazoan origin (*h_U_*≤0) gave matches. If we adjust the blast cut-off to 10^−10^ and 10^−15^, these proportions are approximately the same: 0.7% vs. 3.3%, and 0.8% vs. 3.6%, respectively. This shows that transcripts for nuclear mitochondrial genes are less likely to be found in the foreign sequence set than among metazoan transcripts and therefore will not cause an overestimate of HGT.

The complexity of foreign gene expression observed in the bdelloid rotifer *A. ricciae* is comparable to that in prokaryotes [31] and is far greater than in other animals where relatively few genes are involved [14], [25], [28]. For example, while in *Hydra* perhaps 50 foreign genes are active [28], in *Drosophila ananassae*, which has acquired most of the genome of its endosymbiont, *Wolbachia*, by HGT, only 28 genes are transcribed; the model fly, *D. melanogaster*, has not acquired the *Wolbachia* genome [32], [33]. In pea aphids, red body colour results from the expression of carotenoid genes acquired and diversified from fungal counterparts [34], [35]. In the sea slug, *Elysia chlorotica*, HGT and expression of the algal *psbO* gene allows photosynthesis in plastids also acquired from the alga [36]. However, there is a need for more animal studies at the whole transcriptome level. It is surprising, for example, that there are no comprehensive global studies of HGT in *C. elegans* in the literature [37], as our analysis suggests there are approximately 200 foreign transcripts in the model nematode. The software pipeline developed for this study has the potential to be used more widely where expression data are available to gain a more complete picture of HGT in metazoans.

Nevertheless, the scale of HGT in the bdelloid seems to be unusual among animals and it would be interesting to address the importance of asexuality and desiccation tolerance in this phenomenon. For example, transcriptome data from the nematode *Panagrolaimus superbus*, which is anhydrobiotic, but gonochoristic (i.e. reproduces only sexually), has recently been published [38]. The authors highlighted one foreign sequence in the *P. superbus* transcriptome, but did not perform a global analysis for HGT. If this nematode contains low numbers of foreign sequences, it would rule out that desiccation tolerance *per se*, without asexuality, is associated with extensive HGT. Another characteristic of HGT in *A. ricciae* is that the source organisms are extremely diverse and include examples that are unlikely to be symbionts or even in the bdelloid's immediate habitat, such as the trypanosome relative from which trypanothione biosynthesis genes derive. Therefore, bdelloids are likely able to readily scavenge and incorporate DNA from the environment, and desiccation, which could lead to both leakiness in cell membranes and double-strand breaks in rotifer chromosomes, might facilitate this.

HGT in the bdelloid has the potential to radically extend and complement metazoan biochemistry, since approximately 80% of foreign sequences are concerned with enzyme activity, much of which is novel in animals. This supports the complexity hypothesis, which states that genes whose products are involved in relatively few protein-protein interactions, such as those specifying enzymes, are more likely to be horizontally transferred than those with a higher degree of connectivity, like transcription factor genes [17], [39], [40]. Thus, although the complexity hypothesis was developed to explain observations in prokaryotes, it also seems to apply to the large scale HGT observed in the bdelloid. It would be interesting to investigate in the bdelloid a more recent suggestion from a study in prokaryotes that highly expressed genes are less likely to be horizontally transferred between organisms [41]. Technically, this might be difficult to achieve, as we estimate there are at least 533 source organisms that have contributed to the bdelloid genome by HGT, but we will explore this in future work.

The novel biochemistry implicated includes the ability to degrade toxins, and indeed to exploit them and a range of otherwise unmetabolisable organic molecules as food sources, and to use novel biosynthetic pathways to generate protective molecules, for example antioxidants, or nutrients that are rare in the environment. This is expected to increase bdelloid stress tolerance and competitiveness, and could be important for anhydrobiosis. Bdelloids do not produce trehalose or other non-reducing disaccharides [42]–[44] and have unusual LEA proteins [26], [44], [45], and therefore mechanisms associated with desiccation tolerance in other anhydrobiotes do not apply. Recently, the bdelloid *A. vaga* was shown to have high antioxidant capacity; this reduces protein oxidation, which is thought to be a major problem caused by desiccation and the dry state [22]. Antioxidants in bdelloids have not been characterised, but it will be of interest to determine how far HGT plays a role; this is currently under investigation.

It is also tempting to speculate that HGT facilitates long-term persistence in the absence of sex: asexuals are unable to bring together novel gene combinations arising within a population since they lack conventional genetic exchange mechanisms; equally, asexuals cannot eliminate detrimental mutations readily [10], [11]. Uptake and expression of genes from other organisms is a means of diversifying functional capacity, particularly biochemical capacity, and the potential to replace defective genes with foreign counterparts could protect against loss of function through mutation.

## Materials and Methods

### *Adineta ricciae* cDNA library preparation, sequencing, and assembly

The bdelloid rotifer *Adineta ricciae* was supplied by Claudia Ricci, University of Milan. A clone culture was split into four populations: one was kept hydrated and the other three were dehydrated for 24, 48 and 72 h, as described previously [9]. RNA was extracted separately from each bdelloid population with TRI reagent (Sigma) according to manufacturer's instructions. RNA purity and concentration were measured with a NanoDrop spectrophotometer. Oligo(dT)-primed cDNA from all four sets was prepared with a Clontech/Takara SMART PCR cDNA Synthesis Kit and an Advantage 2 PCR Enzyme System using Invitrogen SuperScript III Reverse Transcriptase. 1 µg cDNA from each preparation was pooled and the resulting mixed cDNA library was normalized with Evrogen Trimmer cDNA normalization kit, according to manufacturer's instructions. About 8 µg of both the normalized and non-normalised cDNA library (each made of the mixed of hydrated and desiccated rotifers) were pooled and a paired-end sequencing library with insert size 200 bp was prepared. Massively parallel Illumina sequencing was performed, resulting in 19.5 million 76-base reads. These were assembled with the CLC-bio (www.clcbio.com) assembler, using a k-mer size of 22, no minimum contig length and all other options at the default settings. The resulting assembly used 9,048,520 of the reads (46.4%) for a total length of 27,227,333 bp giving an average coverage of 25.3 times. This produced a library of 61,219 transcript contigs of size range 118–3674 bp, with median size 341 bp, and mean size 445 bp (standard deviation 295 bp). Transcript contigs have accession numbers HE687510 to HE716431.

### HGT index determination

Analysis of the bdelloid transcriptome was performed using R (The R Project for Statistical Computing, http://www.r-project.org/) complemented with NCBI-Blast 2.2.23+–2.2.25+ (Basic Local Alignment Search Tool) [46], ClustalW2 (EMBL-EBI) and PhyML 3.0 [47]. Blastx was used to compare the complete set of 61,219 bdelloid transcripts against taxa-specific subsets of UniProtKB, labelled as Metazoa, Plantae, Fungi, Eubacteria, Archea and “Other Eukaryotes” (Eukaryotes which are neither Metazoa nor Plants nor Fungi). The taxa-specific subsets only included sequences from complete proteomes (keyword: KW-0181) in order to reduce the search space and to avoid bias towards specific types of proteins that have been sequenced in many organisms. E-value and bitscores were collected for the best five hits of each transcript against each taxon, and 32,297 sequences that did not have any match with at least one taxon with an E-value≤10^−5^ were excluded from further analysis. The alien index [8] and the HGT index (*h_U_*) were calculated for each of the remaining 28,922 sequences. The HGT index (*h_U_*) is calculated as the difference between the highest non-metazoan and the highest metazoan bitscore. Bitscores, being independent of the search space, do not depend on the size of the database used to calculate the blast score, reducing the incorrect determination of sequences. Setting the *h_U_* threshold value is explained in the text. Similar analyses were performed for the *C. elegans* (WormBase release WS226; www.wormbase.org), *D. melanogaster* (FlyBase release r5.37) and *B. plicatilis* transcriptomes. In the first two of these cases, proteins from the phylum containing the test organism (i.e. Nematoda/Arthropoda) were excluded from the Metazoan database, as is common practice [8], [28]. For both *A. ricciae* and *B. plicatilis* this exclusion was not necessary as there are currently no complete proteomes available for the phylum Rotifera. For *B. plicatilis*, ESTs with accession numbers FM897377–FM945301 [48] were first assembled with CAP3 [49] into 16024 contigs, which became 9685 contigs after filtering for a blastx E-value≤10^−5^.

### Phylogenetic analysis

To confirm the non-metazoan origin of the sequences with *h_U_*≥30 and at least one significant metazoan hit, each transcript meeting these conditions was translated and aligned using ClustalW2 to the output (the best hits for each of the five taxa) of the previous blastx analysis. Each alignment was then trimmed to exclude regions where only one of the sequences was present, and phylogenetic trees were built in PhyML from amino-acids sequences using a JTT model [15]; branch support was calculated with the aLRT (approximate Likelihood-Ratio Test) method. The transcripts were then assigned to one of five groups according to the aLRT support for each metazoan or non-metazoan taxon: group 1 contains sequences that are monophyletic with Metazoa (or where there were only metazoan hits from the blast analysis); group 2 contains sequences for which monophyly with Metazoa cannot be strongly rejected; group 3 contains cases where there are too few sequences to define a meaningful clade; group 4 contains cases were monophyly with Metazoa can be strongly rejected; group 5 contains transcripts which are monophyletic with another single (non-metazoan) taxon. Analysis of these data showed that 98% of the sequences with at least one significant metazoan hit and *h_U_*≥30 are truly non-metazoan as they fall into groups 4 and 5 (Table 1; Table S1). To compare the bdelloid transcriptome to those of other species, the same analysis was performed on the published transcriptomes from the monogonont rotifer *B. plicatilis*, the nematode *C. elegans* and the fly *D. melanogaster*, calculating the percentage of sequences above threshold for a given value of *h_U_* as shown in Figure 1A.

### Manual sequencing of selected genomic DNA regions around some foreign genes

To confirm the presence of foreign genes in the bdelloid genome and to assess the possibility of contamination from food, symbionts, parasites and other organisms, we manually sequenced the genomic DNA around some genes of interest. A number of assembled transcript fragments, chosen at random from a subset of foreign sequences encoding biochemical functions that have never been reported in metazoans, were blast screened against a (partial) genome assembly of *A. ricciae* and the longest genomic DNA contig for each transcript was identified. This was then compared using blastx to the NCBI non-redundant database to find other genes on the same genomic DNA fragment, and primers were designed around these regions. Genomic DNA was extracted from an *A. ricciae* population derived from the original, and 11 individual regions, were PCR amplified using Finnzymes Phusion High Fidelity Taq polymerase, adding an A overhang after PCR with Advantage 2 PCR Enzyme System. The resulting PCR product was cloned into pCR 2.1 TOPO TA (Invitrogen), inserted into competent *E. coli* (New England BioLabs) and white-blue colony screening was performed. Ten positive colonies for each PCR product were chosen, and plasmid DNA was purified and restriction digested to check for insert size. One clone for each genomic DNA region was sequenced via primer walks using a standard dideoxy method at the University of Cambridge Department of Biochemistry Sequencing Facility. Of the 11 attempted, eight are shown as Figure 2B. For the remaining three examples, one amplification worked, but sequencing could not be completed since the insert was long and unstable in *E. coli*: although the sequence of the middle of this fragment could not be determined, we confirmed that one end contained a metazoan gene and the other contained two genes of bacterial origin. Another amplification was not of the target region, and one amplification failed altogether.

The successfully amplified and sequenced genomic DNA regions were then manually aligned in Geneious (www.geneious.com) with the relevant transcripts from the library, then blastx aligned against the non-redundant NCBI database and annotated. Each annotated gene was considered metazoan or not-metazoan according to its best hits in the published database. Figure 2B represents eight genomic regions with the annotated genes colour-coded according to the tree in Figure 2A (metazoa, black; eubacteria, blue; fungi, pink; protists, grey). In two cases, two foreign genes are present on the same genome fragment, but they derive from different taxa. Occasionally, a gene next to a foreign representative has been identified previously in a bdelloid rotifer species, and is annotated with an asterisk in the figure. Figure 2B shows the shortest region including one foreign gene and one metazoan (or another non-metazoan from a different taxon) gene, but in a few examples the actual sequenced region was longer than shown. Accession numbers for these eight genomic regions are HE662868 to HE662875.

### Comparison with the genome

Transcripts were aligned to the draft genome using blastn. To determine the total length of alignment of a transcript all matches for that transcript fulfilling the following criteria were used: 1) E-value≤10^−3^; 2) non-overlapping with any previous matches; 3) longer than 40 bp (a minimum exon length constraint); and 4) on the same genomic contig as a previous match OR within 1000 bp of the start/end of a genomic contig when a previous match was also within 1000 bp of the start/end of a genomic contig (a maximum intron length constraint). The total aligning length (sum of the length of the matches that fulfill these conditions) was then divided by the length of the transcript and this percentage plotted as a histogram for all transcripts. Transcripts were considered to be “on” a genomic contig if they had a match on it fulfilling the above criteria. For each genomic contig with a foreign transcript on it the number of metazoan transcripts and foreign transcripts with a different origin was calculated.

### EC number assignation and KEGG pathways analysis

The 28,922 sequences with at least one match with E-value≤10^−5^ were blastx aligned to the whole of Swiss-Prot (532,146 proteins at time of analysis) and the results were filtered to give only transcripts with at least one match to a protein that was annotated with an EC number. An HGT index for these transcripts was then calculated as before (but denoted *h_S_*, to show that the comparison was done with Swiss-Prot rather than with UniProtKB, cf. *h_U_*). Based on *h_S_*, the transcripts were then subdivided into three groups: horizontally transferred (*h_S_*≥30), indeterminate (0<*h_S_*<30) and metazoan (*h_S_*≤0). For horizontally transferred and metazoan transcripts the EC numbers of their first match were collated and input into the KEGG website (http://www.genome.jp/kegg/tool/map_pathway1.html) to determine which KEGG pathways they occurred in.

EC numbers were also assigned a colour: green (EC number is only annotated to matches of transcripts with metazoan origin), red (EC number is only annotated to matches of horizontally transferred transcripts), grey (EC number is only annotated to matches of transcripts with indeterminate origin), orange (green plus red (and possibly grey)), pink (red plus grey), light green (green plus grey). These were input into the KEGG website (http://www.genome.jp/kegg/tool/map_pathway2.html) to produce the coloured pathway diagrams shown in Figure 3 and Figure S5.

The results were then extracted from the KEGG website and a hypergeometric test performed to calculate which KEGG pathways were enriched for horizontal transfer as compared to the total number of unique EC numbers found for all transcripts and the total number of unique EC numbers found for horizontally transferred transcripts. Benjamini-Hochberg multiple testing correction was performed to reduce the false positive rate (Table S4). The workflow is shown in Figure S6.

## Supporting Information

Figure S1 Plot of *h_U_* against transcript contig length for the four invertebrate species examined with the best fit line in each case shown as a dotted red line and coefficient of determination (*r^2^*) indicated in each panel. The threshold value of *h_U_* = 30 is shown as a solid line. To test whether differences in contig lengths might explain the difference in HGT indices found among taxa, we performed two statistical tests: i) We used the threshold *h_U_* = 30 to assign contigs as horizontally transferred or not (a binary response variable with 1 or 0), as discussed in the main text. We then used a generalized linear model with binomial error structure to test whether the probability per gene of being assigned as horizontally transferred varied among taxa and/or with contig length. Transcripts in *A. ricciae* had a significantly higher probability of having an HGT score above 30 than in the other taxa (*p* for all comparisons<0.001), even when controlling for any correlation with contig length. Across the range of contig lengths observed in the *A. ricciae* assembly, the predicted probabilities of *h_U_*>30 were over five-fold higher for *A. ricciae* than for the other taxa (*A. ricciae* ranged from 0.094 to 0.117, *B. plicatilis*: 0.017 to 0.022, *D. melanogaster*: 0.0045 to 0.0058, *C. elegans*: 0.017 to 0.021). ii) To check whether the differences among taxa were significant irrespective of the choice of threshold used to assign horizontally transferred genes, we also performed an ANCOVA with *h_U_* as the response variable, taxon as an explanatory factor and contig length as a covariate. We square-root transformed the magnitude of *h_U_*. There was a significant correlation between *h_U_* and contig length (slope = −0.0013, t = −95.8, *p*<0.0001), but *A. ricciae* had a significantly higher mean *h_U_* than the other taxa even when controlling for contig length (estimates: *A. ricciae* = −2.40, *B. plicatilis* = −4.20, *D. melanogaster* = −6.29, *C. elegans* = −4.381, all SE<0.064, all *p*<0.001 for comparison with *A. ricciae*). Contig length explained 9.6% of the variation and taxon explained 19.3% of the variation in *h_U_*.
(PDF)

Figure S2 Phylogenetic trees for a selection of *A. ricciae* transcript contigs. Colour coding: the bdelloid sequence under analysis is represented in red; metazoa, black; eubacteria, blue; archaea, light blue; fungi, pink; protists, grey. Figure 2A is shown again as (C) here to allow comparison in the same format. Panels G–M represent those examples from Figure 3 where it is meaningful to construct a tree (i.e. there are significant matches with metazoan counterparts).
(PDF)

Figure S3 Phylogenetic trees for the 98 *C. elegans* transcripts with *h*≥30 and significant blast matches to metazoan sequences. Colour coding as in Figure S2: the nematode sequence under analysis is represented in red; metazoa, black; eubacteria, blue; archaea, light blue; fungi, pink; protists, grey. For each example, two phylogenetic trees are shown: the left-hand tree is constructed without nematode sequences other than the *C. elegans* test sequence, while the right-hand tree also includes the top five nematode matches.
(PDF)

Figure S4 Summary of transcript assignment in UniProtKB or Swiss-Prot. Foreign contigs in red, indeterminate plus metazoan in pale green.
(PDF)

Figure S5 Complete KEGG representation of pathways listed in Figure 3. Colour coding as in Figure 3: green, metazoan; red, foreign; orange, both metazoan and foreign examples identified; pink, both foreign and indeterminate examples; light green, both metazoan and indeterminate examples; blue, not found in transcriptome.
(PDF)

Figure S6 Workflow for determination of the number of horizontally transferred enzymes in each biochemical pathway. Rotifer transcripts were compared with blastx against Swiss-Prot and enzyme-matching transcripts were selected; the HGT index (*h_S_*) was recalculated and every enzyme (EC number) was assigned a colour according to it being represented by sequences that are purely metazoan (green), purely foreign (red), indeterminate (grey) or a combination (pink, red plus grey; orange, red plus green; or pale green, green plus grey). The outputs of these calculations were used to colour code enzymes in KEGG pathways and to calculate statistics for the over-representation of HGT genes in each pathway.
(PDF)

Table S1 List of 28,922 sequences with at least one hit with E-value≤10^−5^. Shown are transcript name, accession number, sequence length, minimum E-value and bit score for each taxon, alien index and best taxon, *h_U_* and best taxon, monophyly support of each taxon, monophyly support of the transcript with each taxon, phylogenetic classification, and summary table of the phylogenetic assignment.
(XLSX)

Table S2 List of 206 *C. elegans* transcripts with *h*≥30. Shown are transcript name, sequence length, minimum E-value and bit score for each taxon, alien index and best taxon, *h_U_* and best taxon, monophyly support of each taxon, monophyly support of the transcript with each taxon, phylogenetic classification, and summary table of the phylogenetic assignment. Data are presented corresponding to both types of analysis shown in Figure S3, i.e. without or with the inclusion of nematode sequences other than the *C. elegans* test sequence.
(XLSX)

Table S3 Mapping of foreign transcript contigs corresponding to enzyme activities in Figure 3 to genomic contigs also containing genes of metazoan origin.
(DOC)

Table S4 An extended version of Table 2. Pathway and identification number, number of contigs labelled as foreign (red), foreign plus indeterminate (pink), indeterminate (grey), metazoan (green), metazoan plus indeterminate (pale green), foreign plus metazoan (orange) and total number of unique matched EC numbers for each one, together with hypergeometric and Benjamini-Hochberg multiple testing correction to check for enrichment of foreign representation in each pathway.
(XLS)
