# Supplementary figures and images for: Biochemical Diversification through Foreign Gene Expression in Bdelloid Rotifers
Source: PLoS Genet. 2012 Nov 15;8(11):e1003035. doi: 10.1371/journal.pgen.1003035 (PMC3499245; doi:10.1371/journal.pgen.1003035)

Boschetti Figure S1

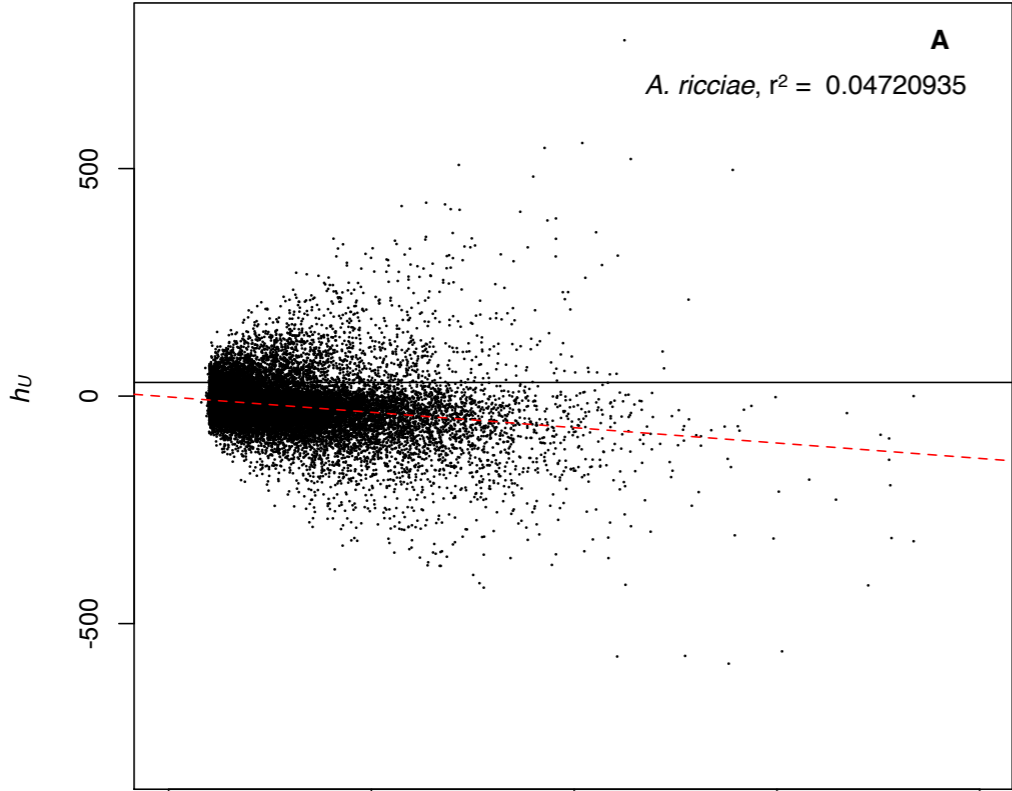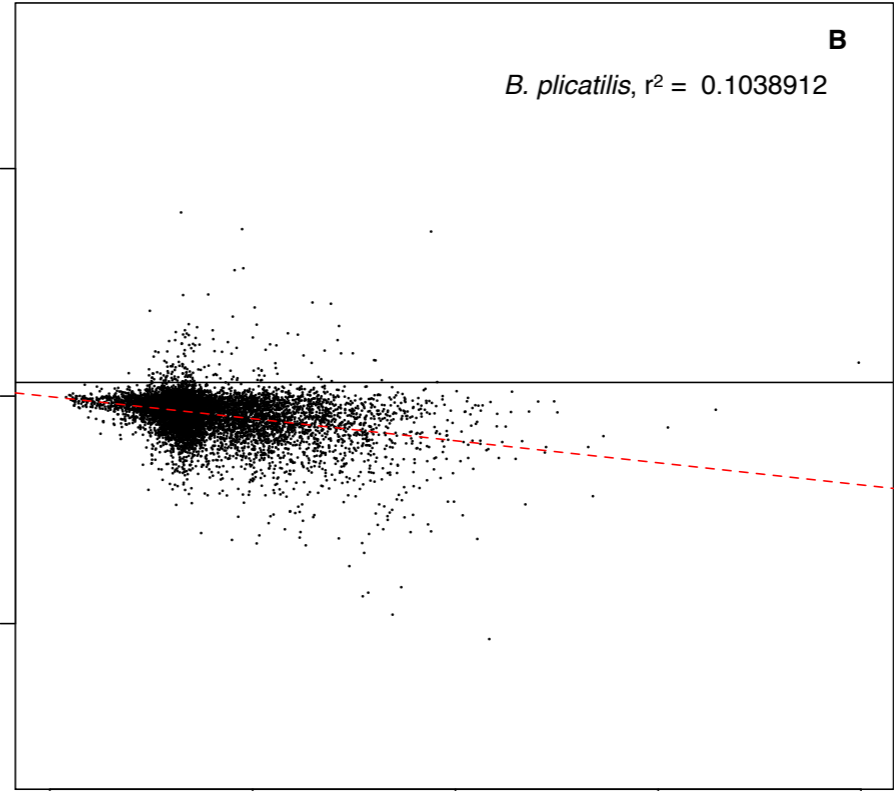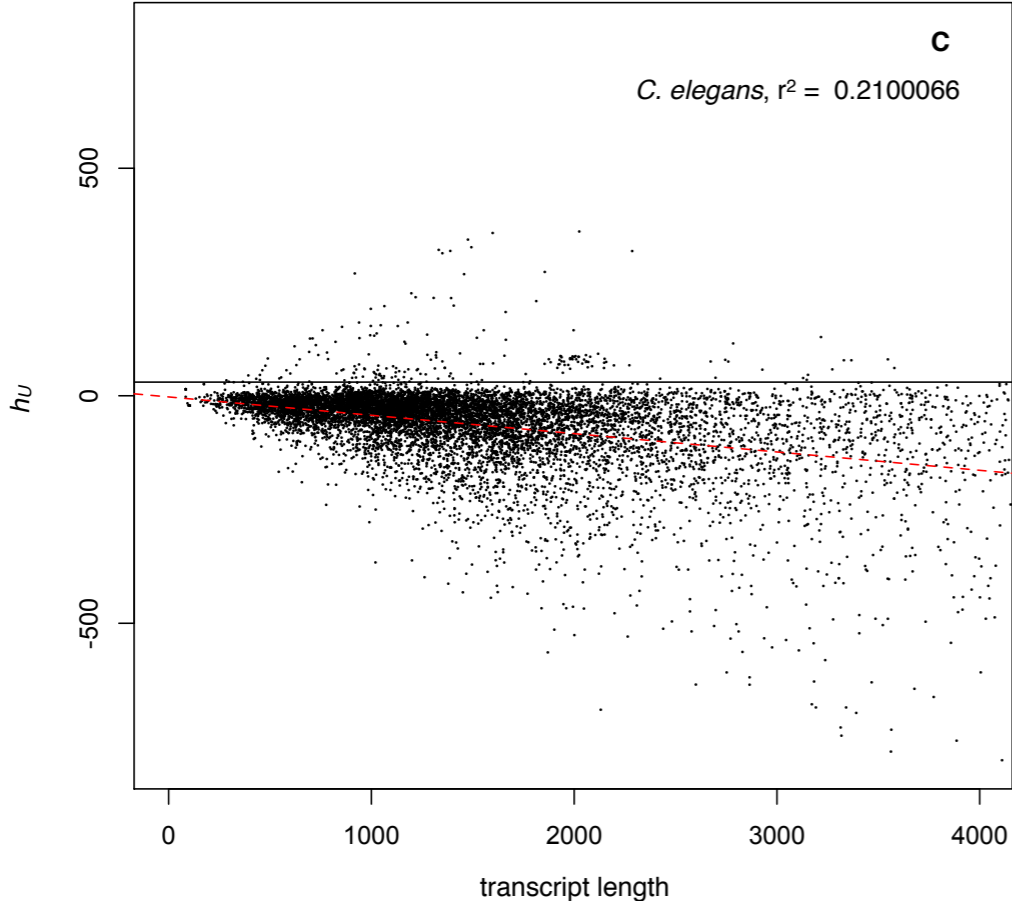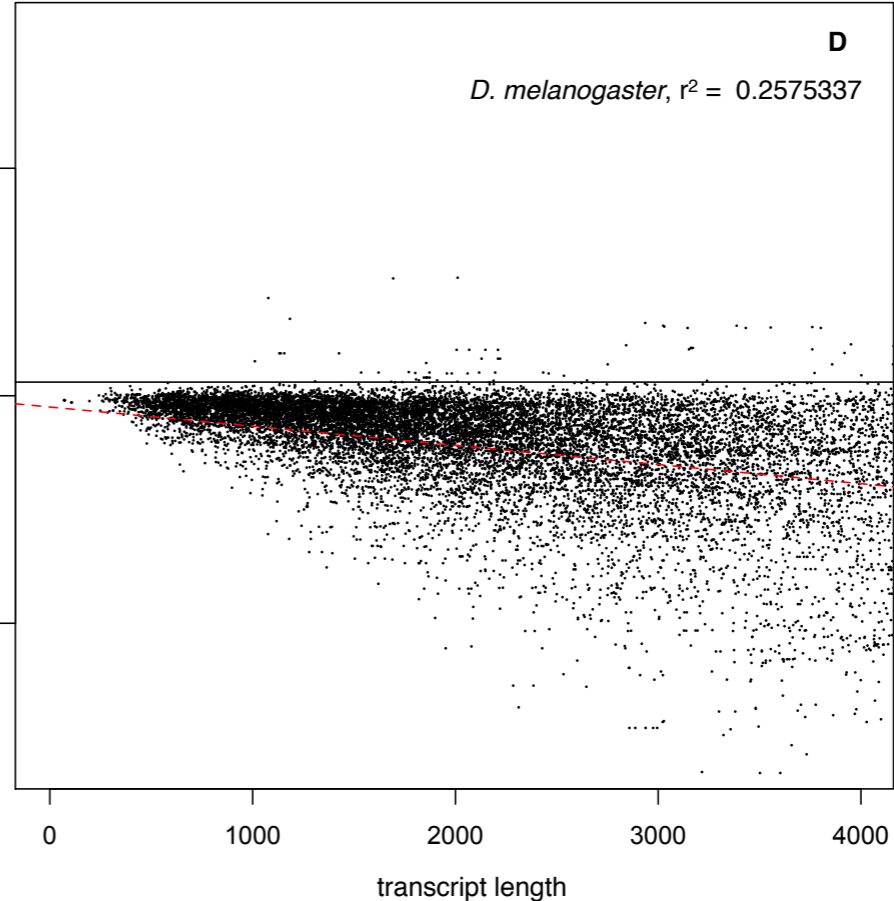

Supplement: Figure S1 — Plot of hU against transcript contig length for the four invertebrate species examined with the best fit line in each case shown as a dotted red line and coefficient of determination (r2) indicated in each panel. The threshold value of hU = 30 is shown as a solid line. To test whether differences in contig lengths might explain the difference in HGT indices found among taxa, we performed two statistical tests: i) We used the threshold hU = 30 to assign contigs as horizontally transferred or not (a binary response variable with 1 or 0), as discussed in the main text. We then used a generalized linear model with binomial error structure to test whether the probability per gene of being assigned as horizontally transferred varied among taxa and/or with contig length. Transcripts in A. ricciae had a significantly higher probability of having an HGT score above 30 than in the other taxa (p for all comparisons<0.001), even when controlling for any correlation with contig length. Across the range of contig lengths observed in the A. ricciae assembly, the predicted probabilities of hU>30 were over five-fold higher for A. ricciae than for the other taxa (A. ricciae ranged from 0.094 to 0.117, B. plicatilis: 0.017 to 0.022, D. melanogaster: 0.0045 to 0.0058, C. elegans: 0.017 to 0.021). ii) To check whether the differences among taxa were significant irrespective of the choice of threshold used to assign horizontally transferred genes, we also performed an ANCOVA with hU as the response variable, taxon as an explanatory factor and contig length as a covariate. We square-root transformed the magnitude of hU. There was a significant correlation between hU and contig length (slope = −0.0013, t = −95.8, p<0.0001), but A. ricciae had a significantly higher mean hU than the other taxa even when controlling for contig length (estimates: A. ricciae = −2.40, B. plicatilis = −4.20, D. melanogaster = −6.29, C. elegans = −4.381, all SE<0.064, all p<0.001 for comparison with A. ricciae). [file pgen.1003035.s001.pdf]

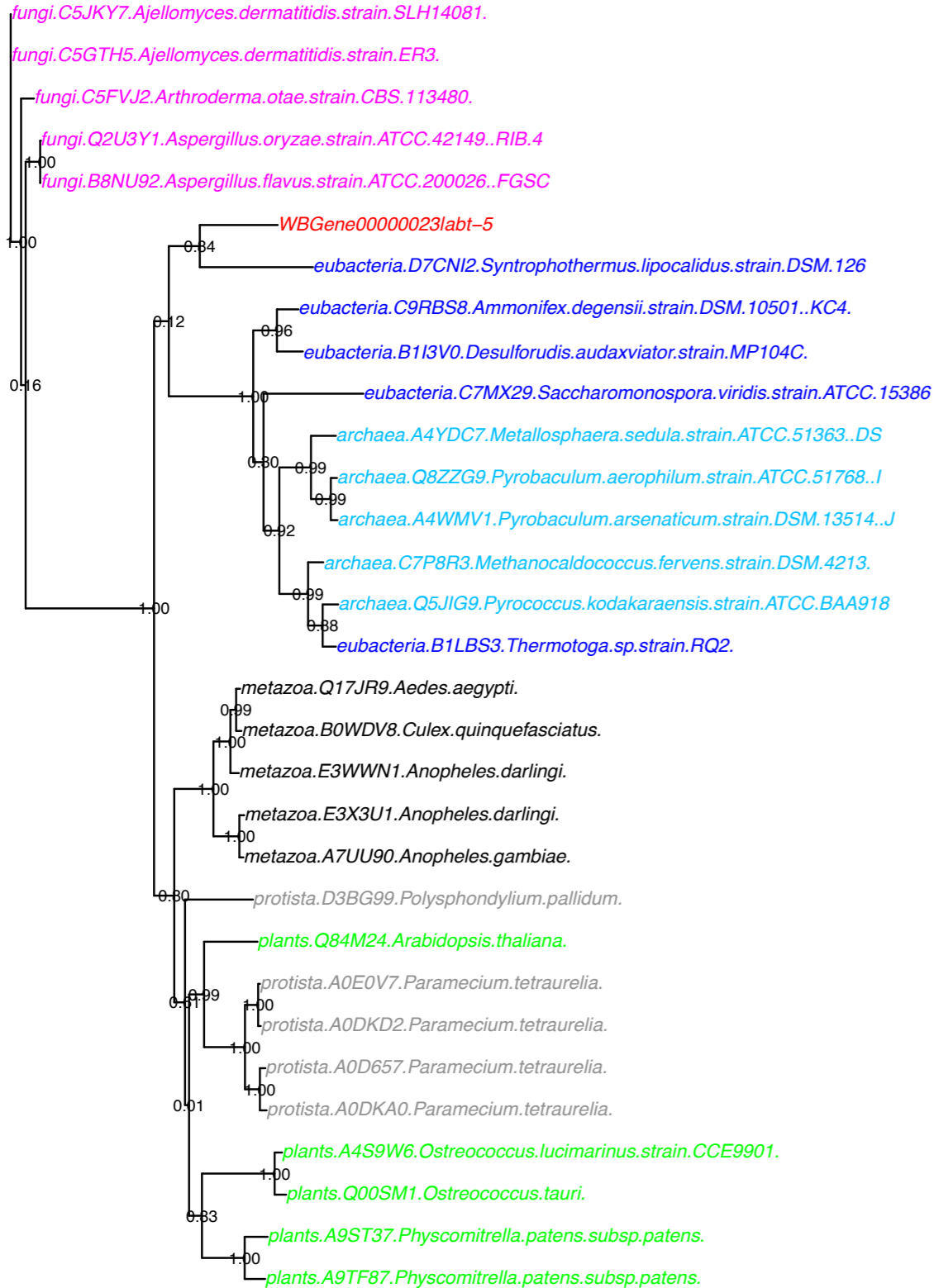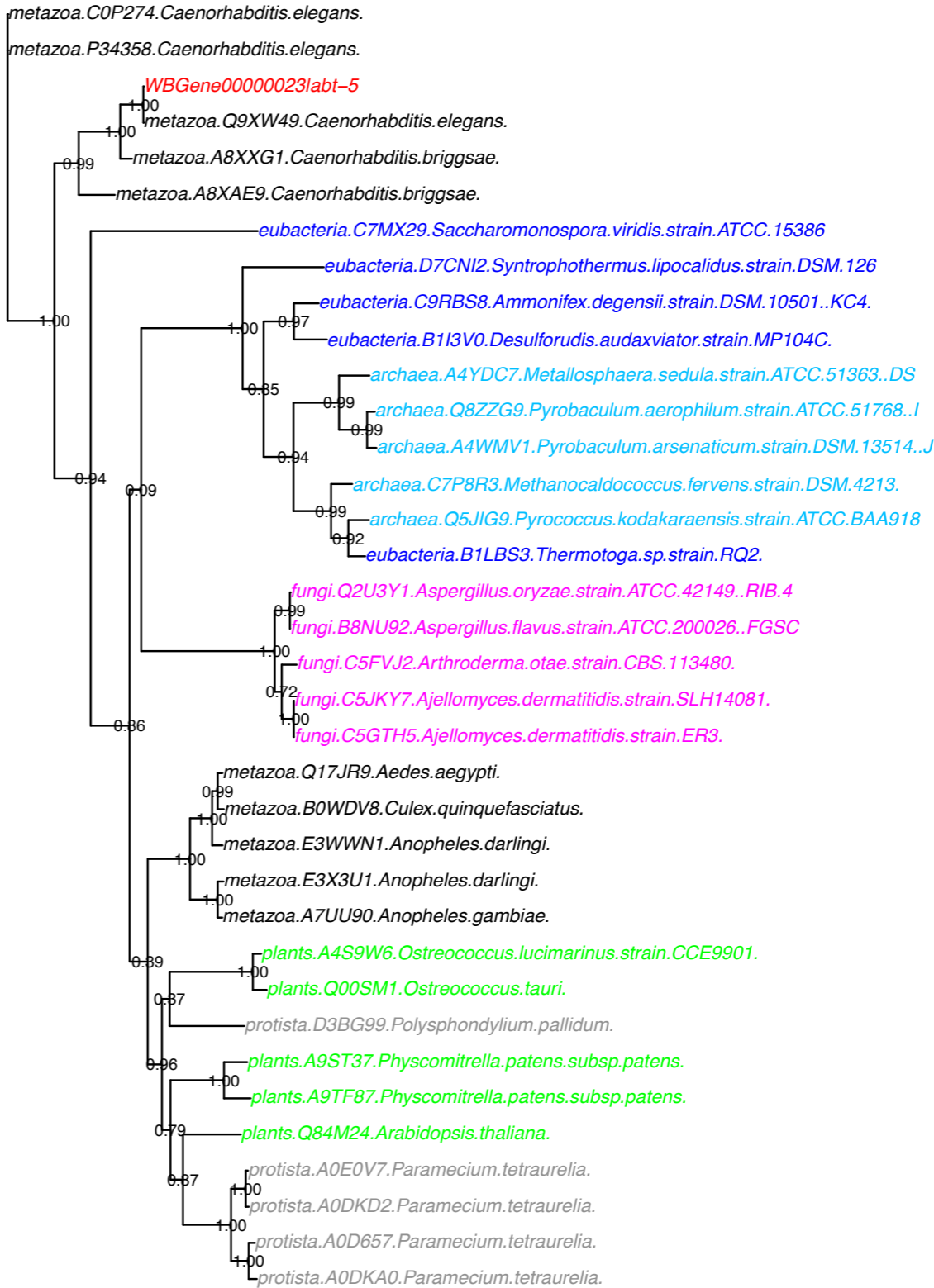

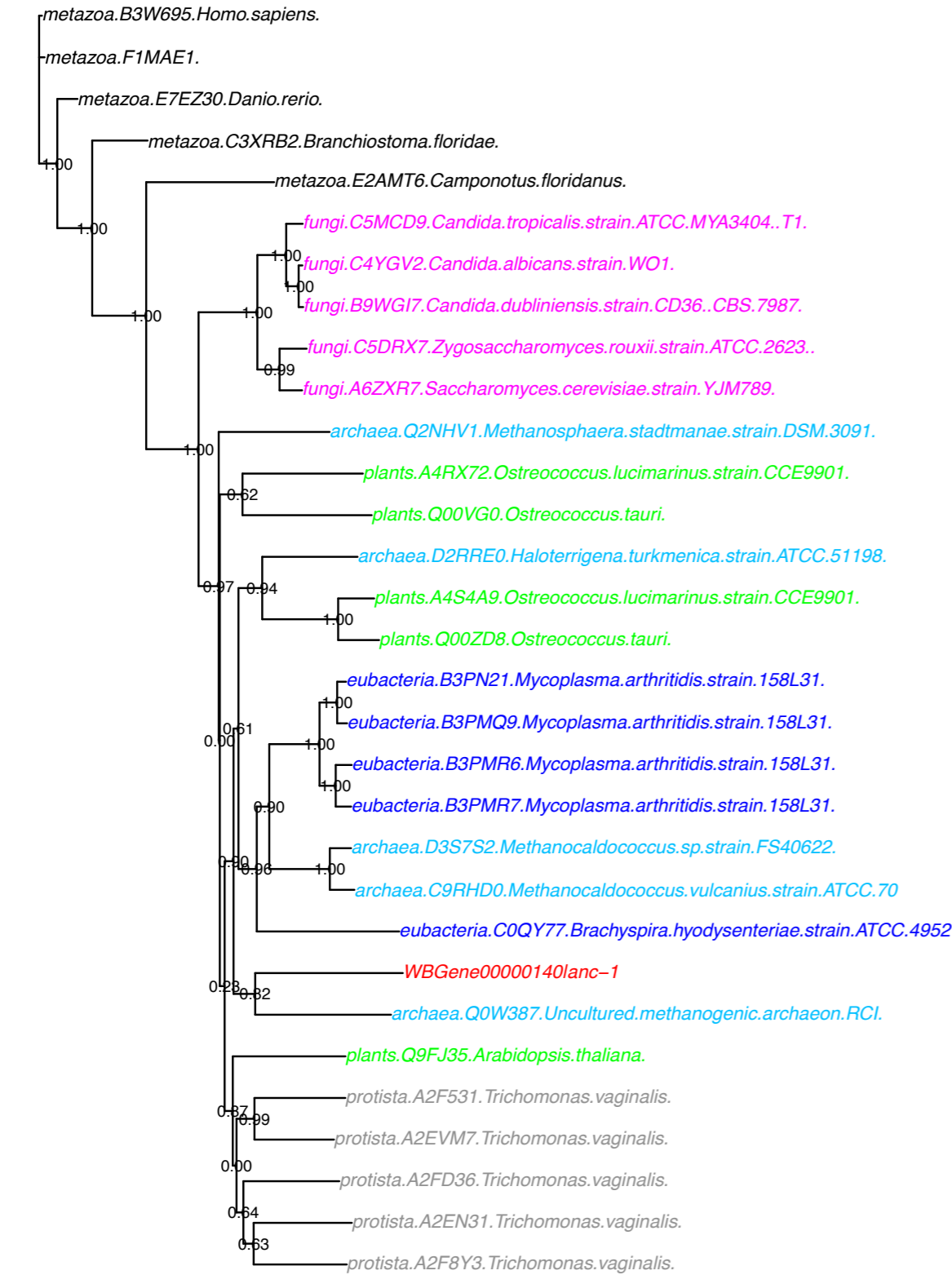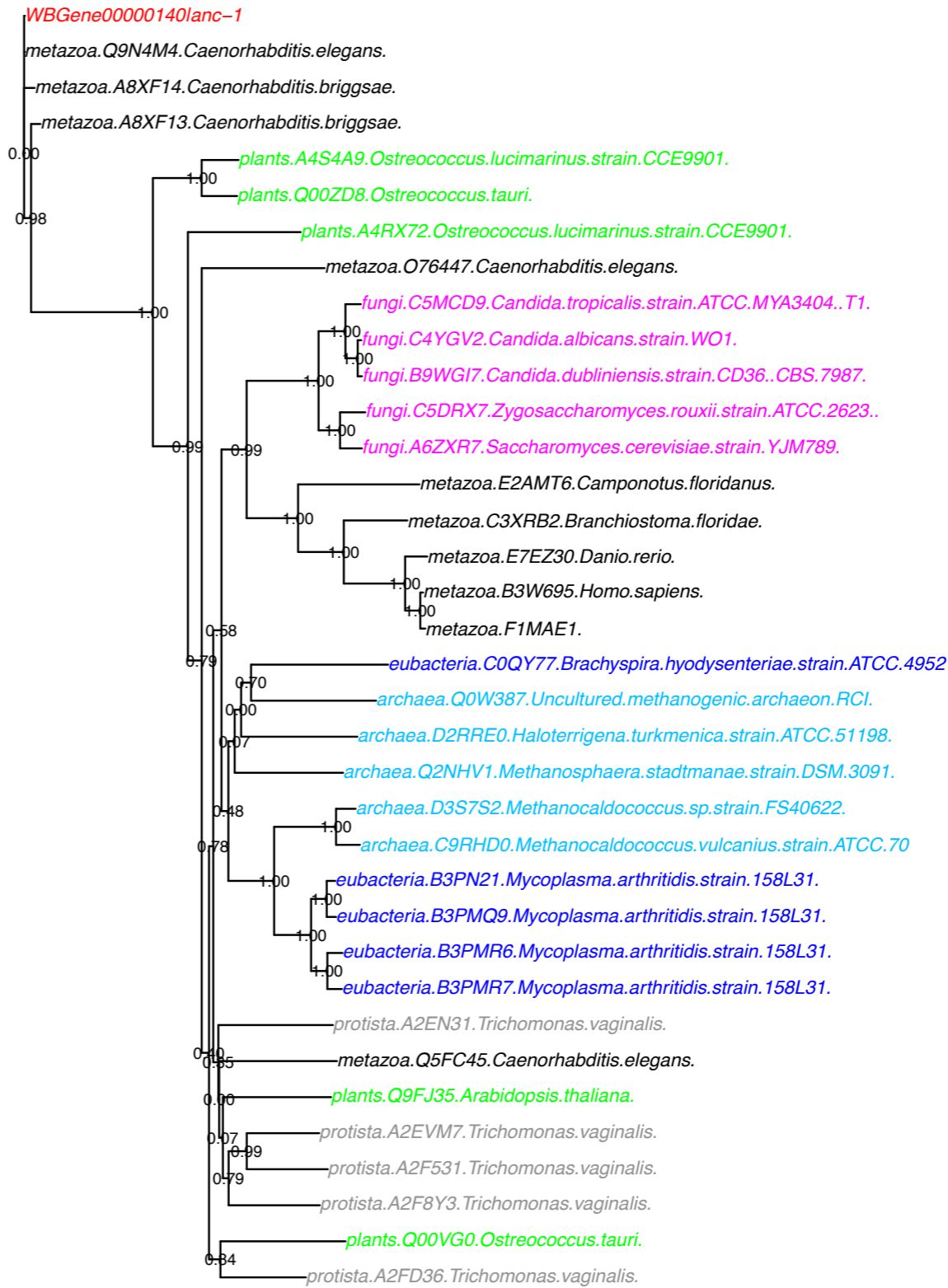

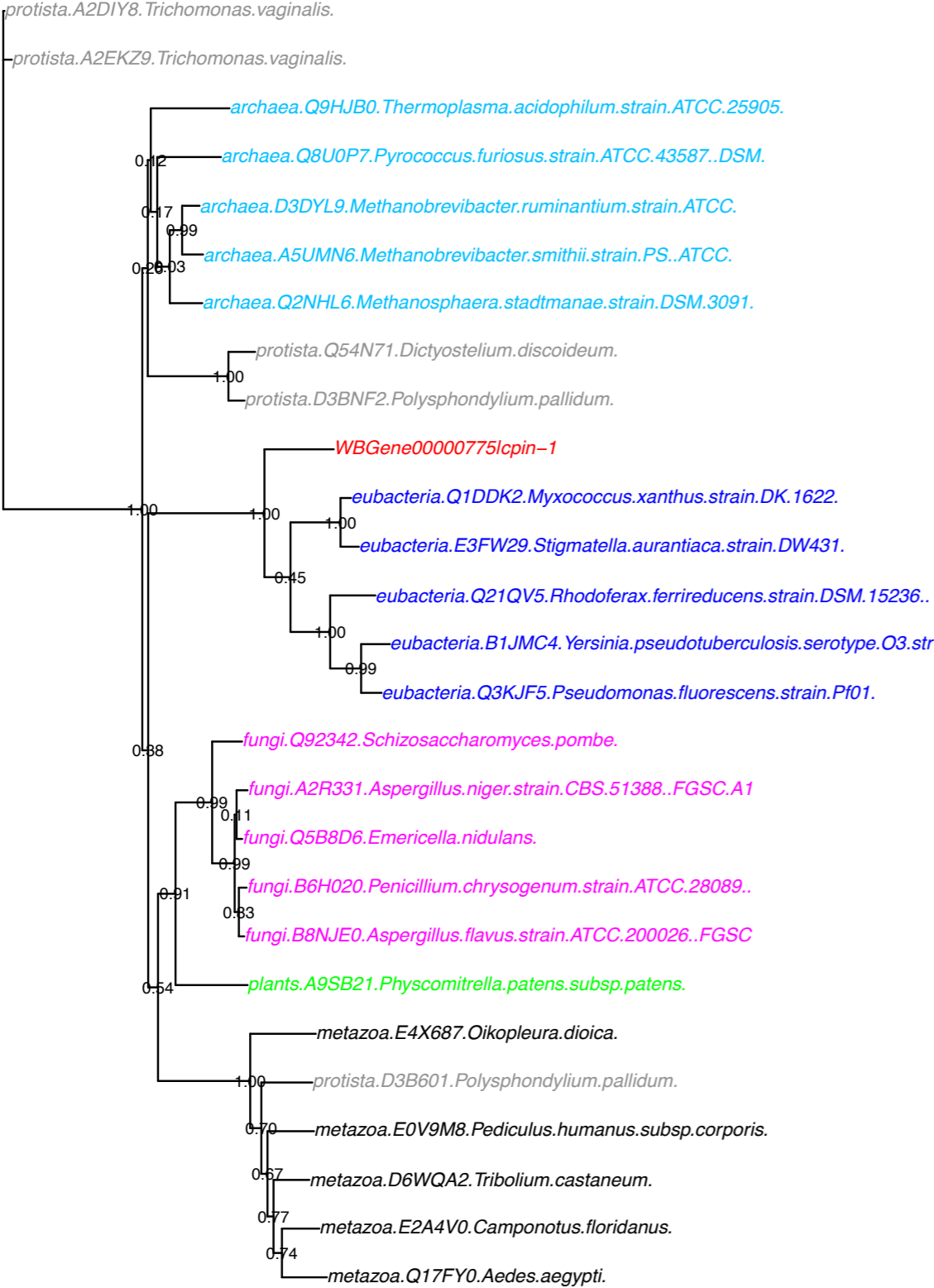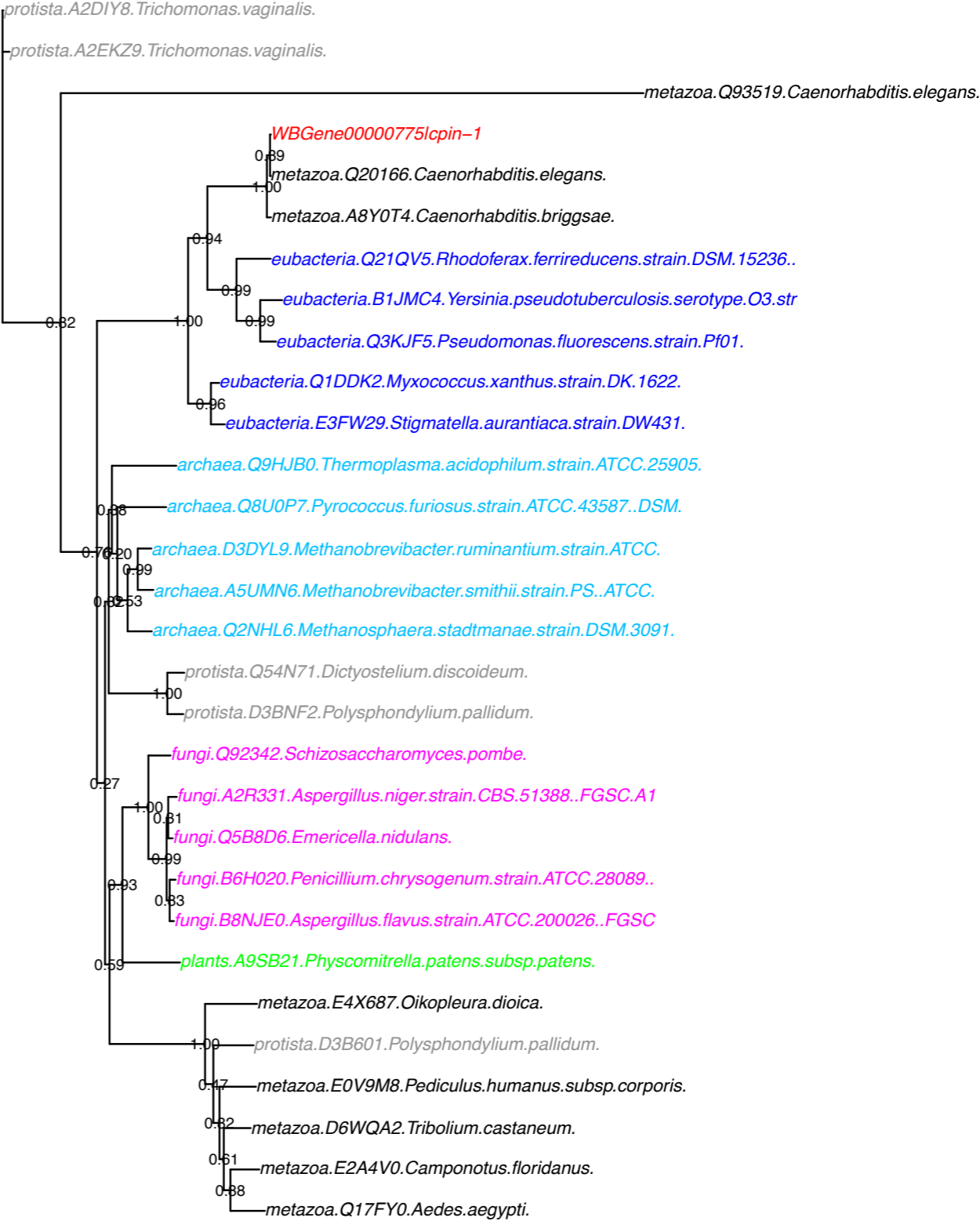

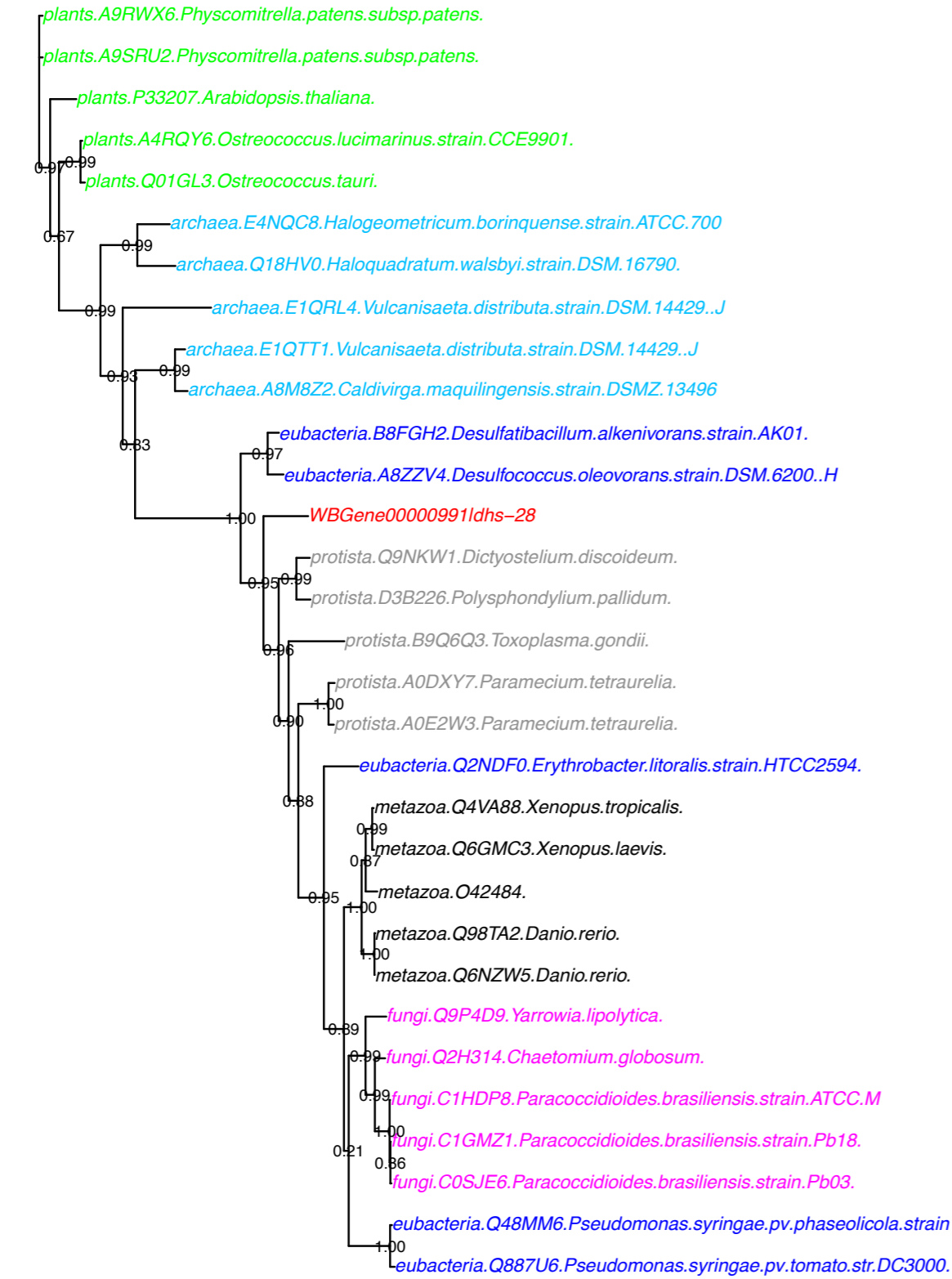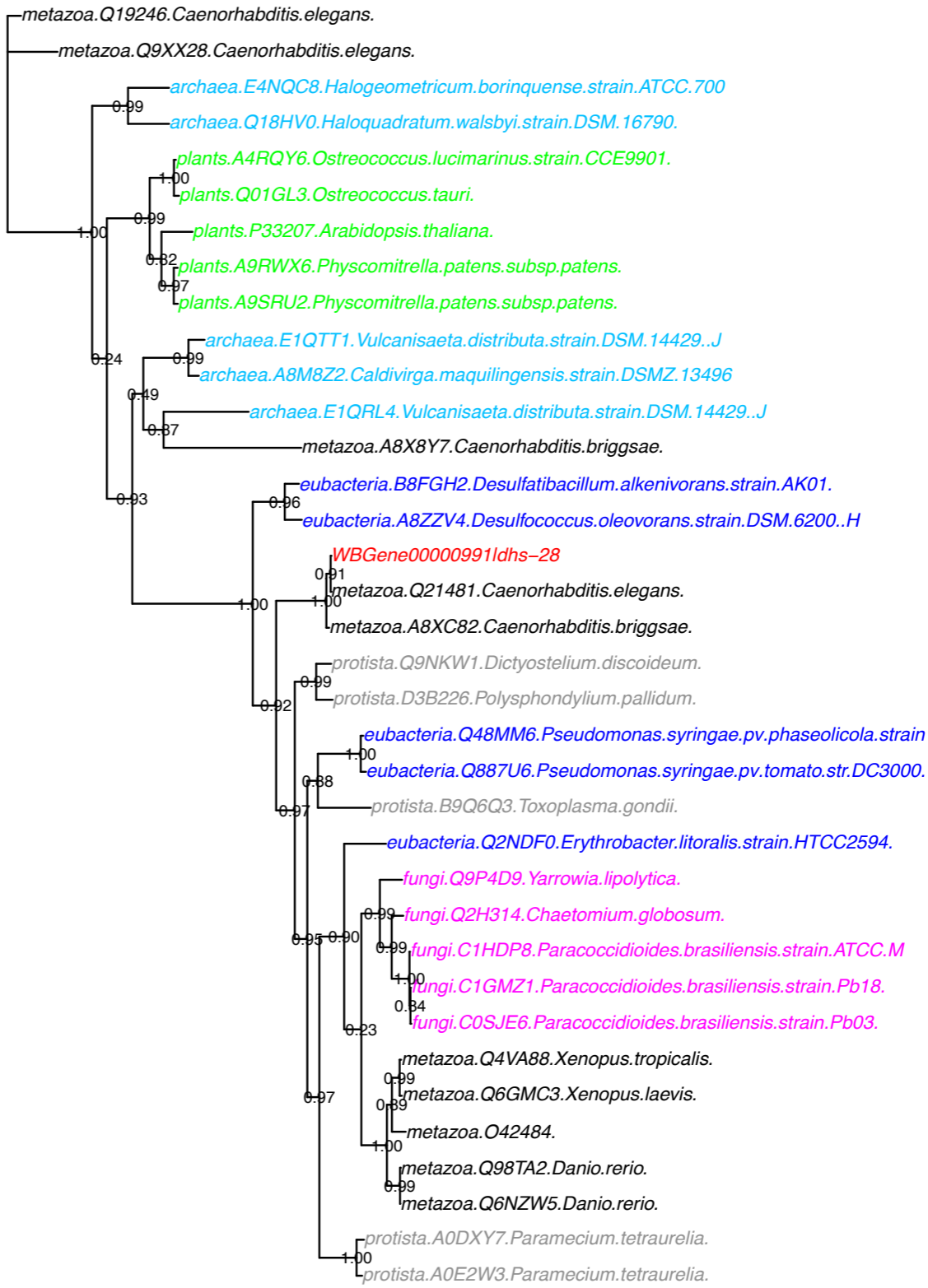

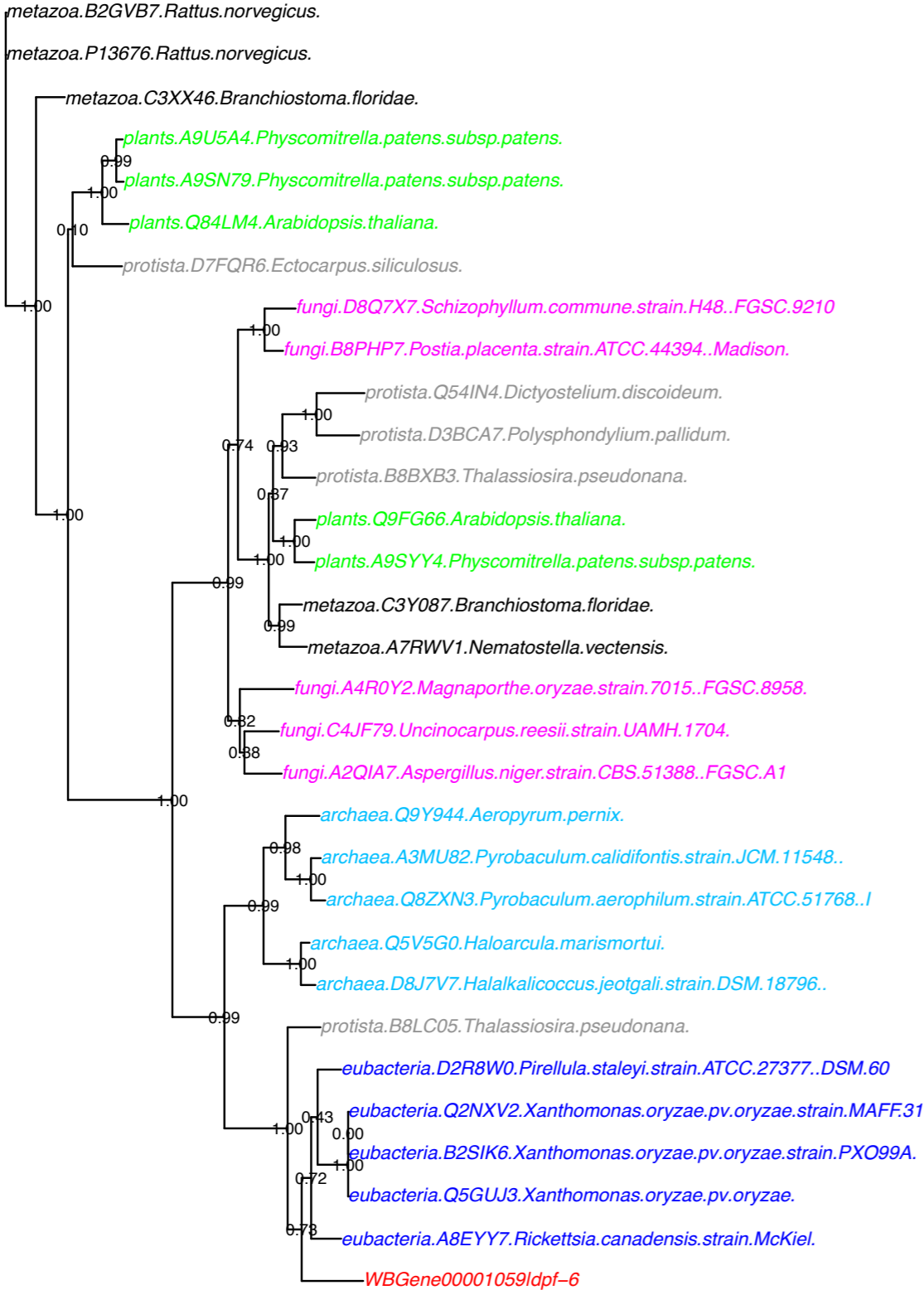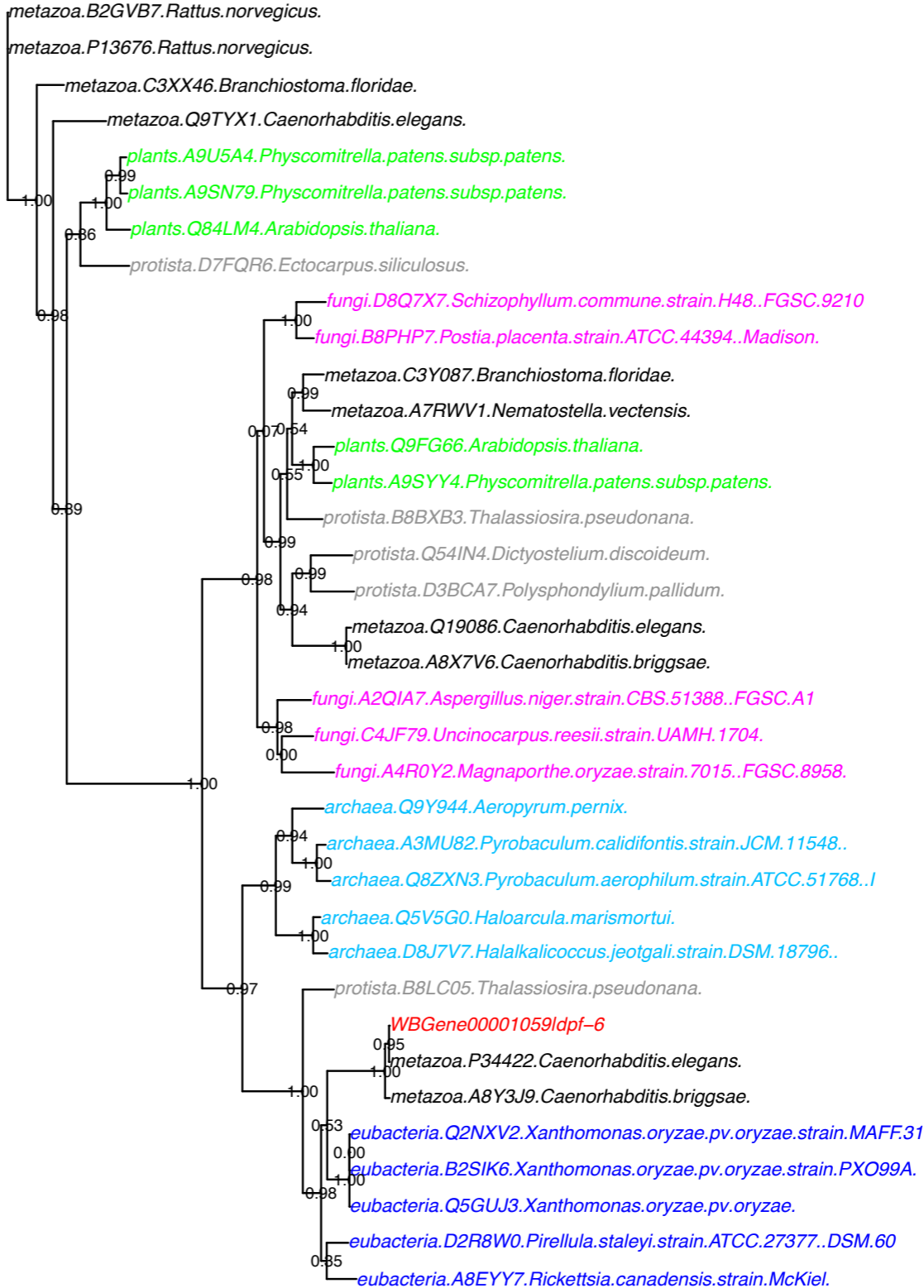

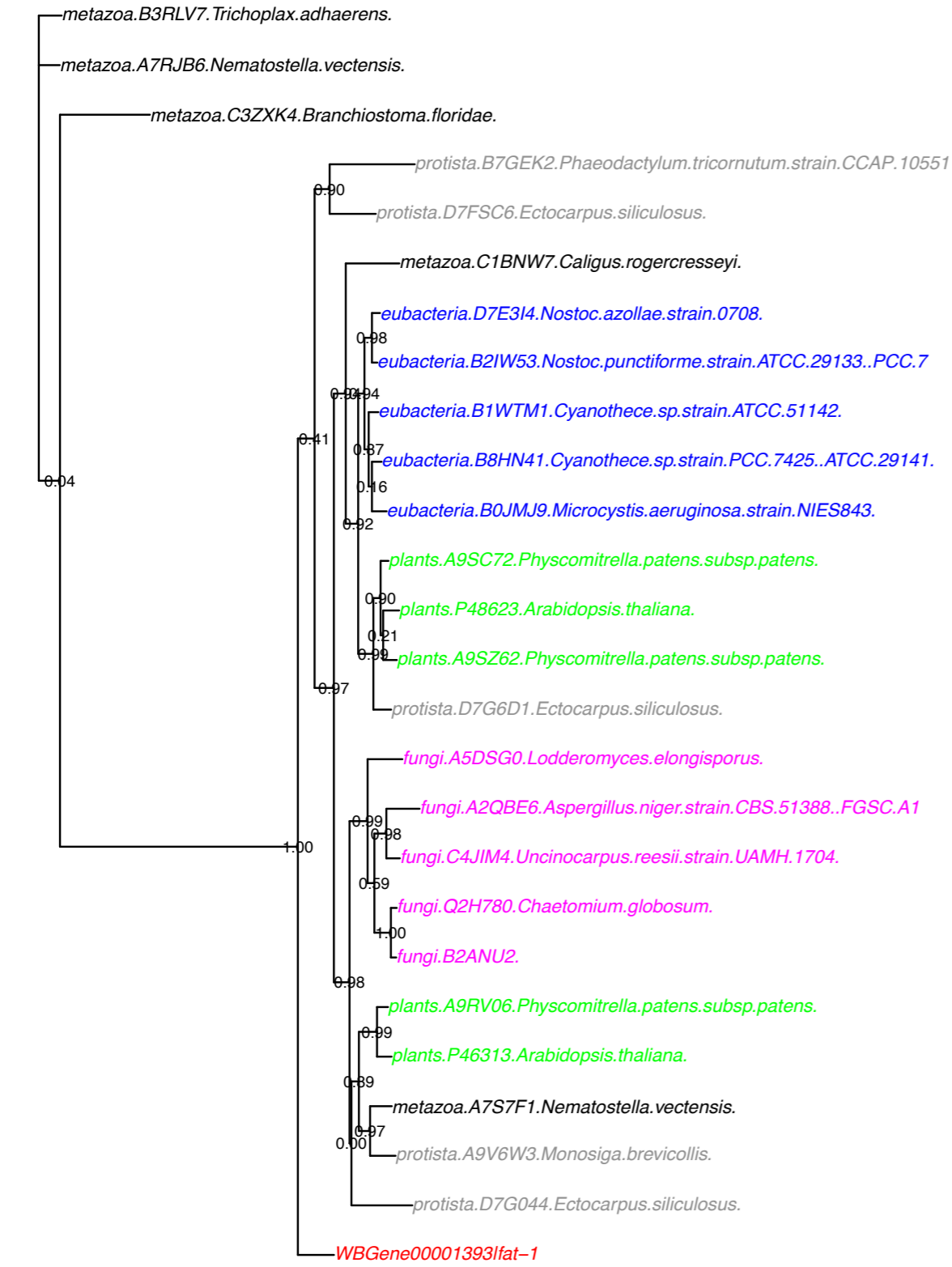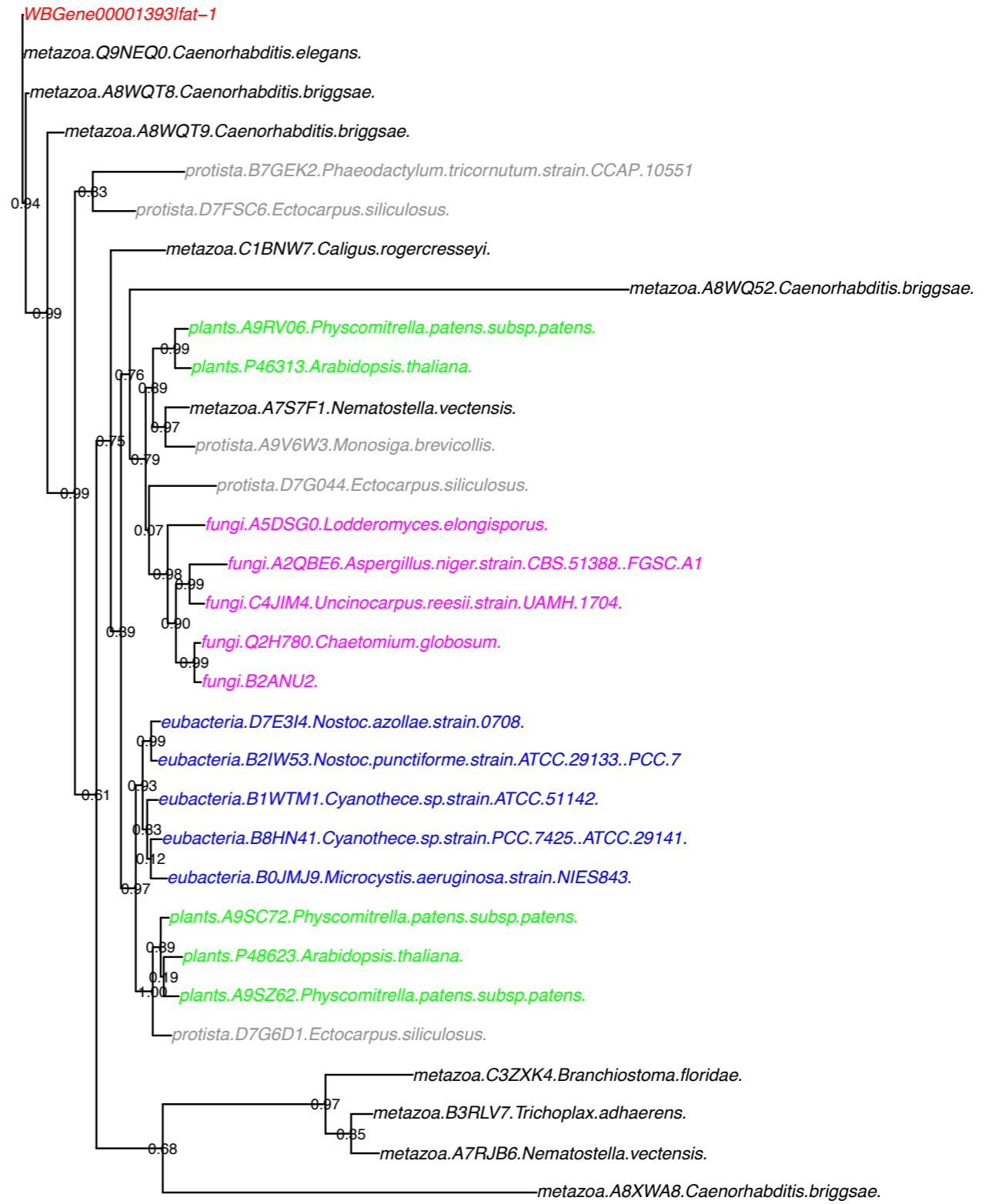

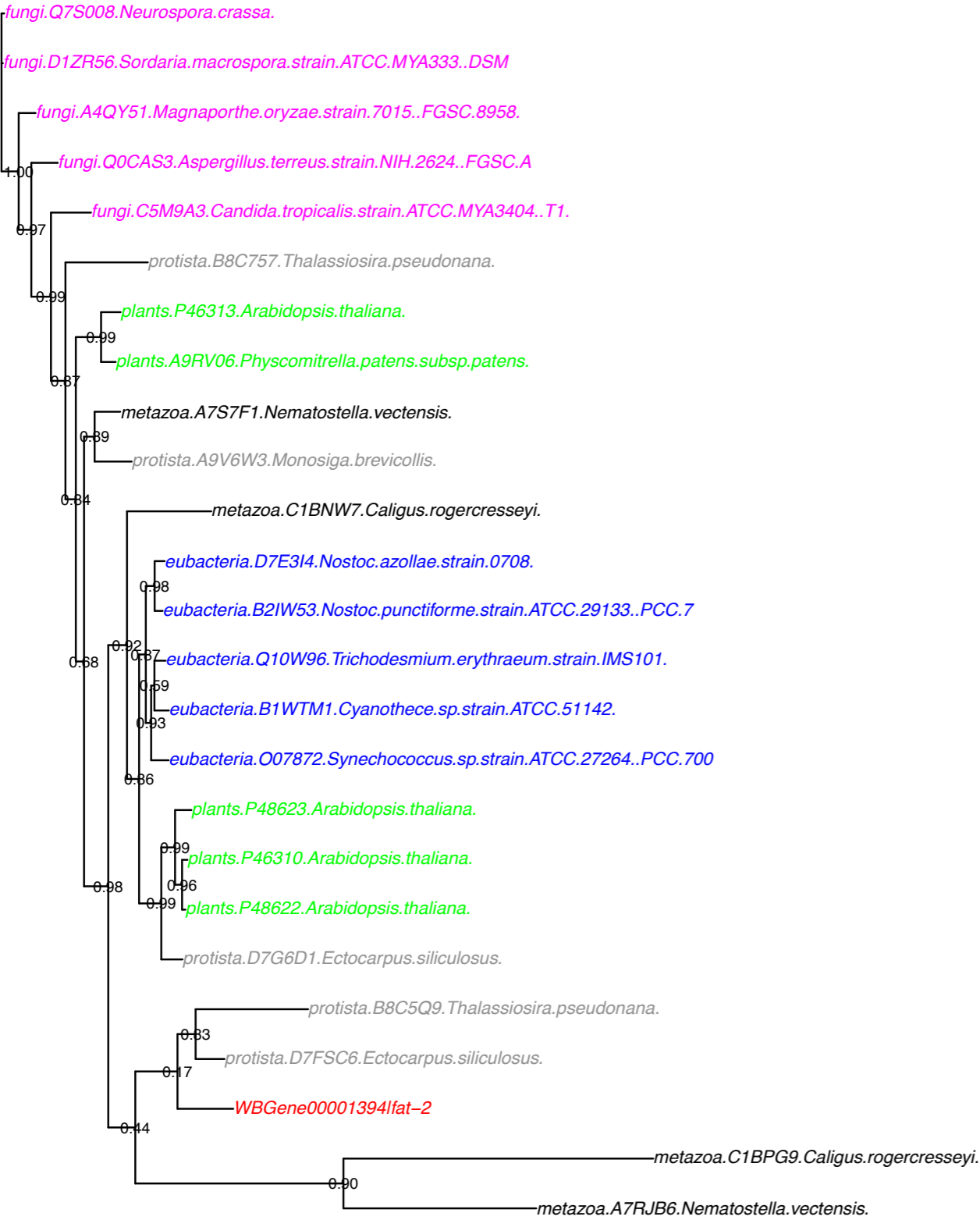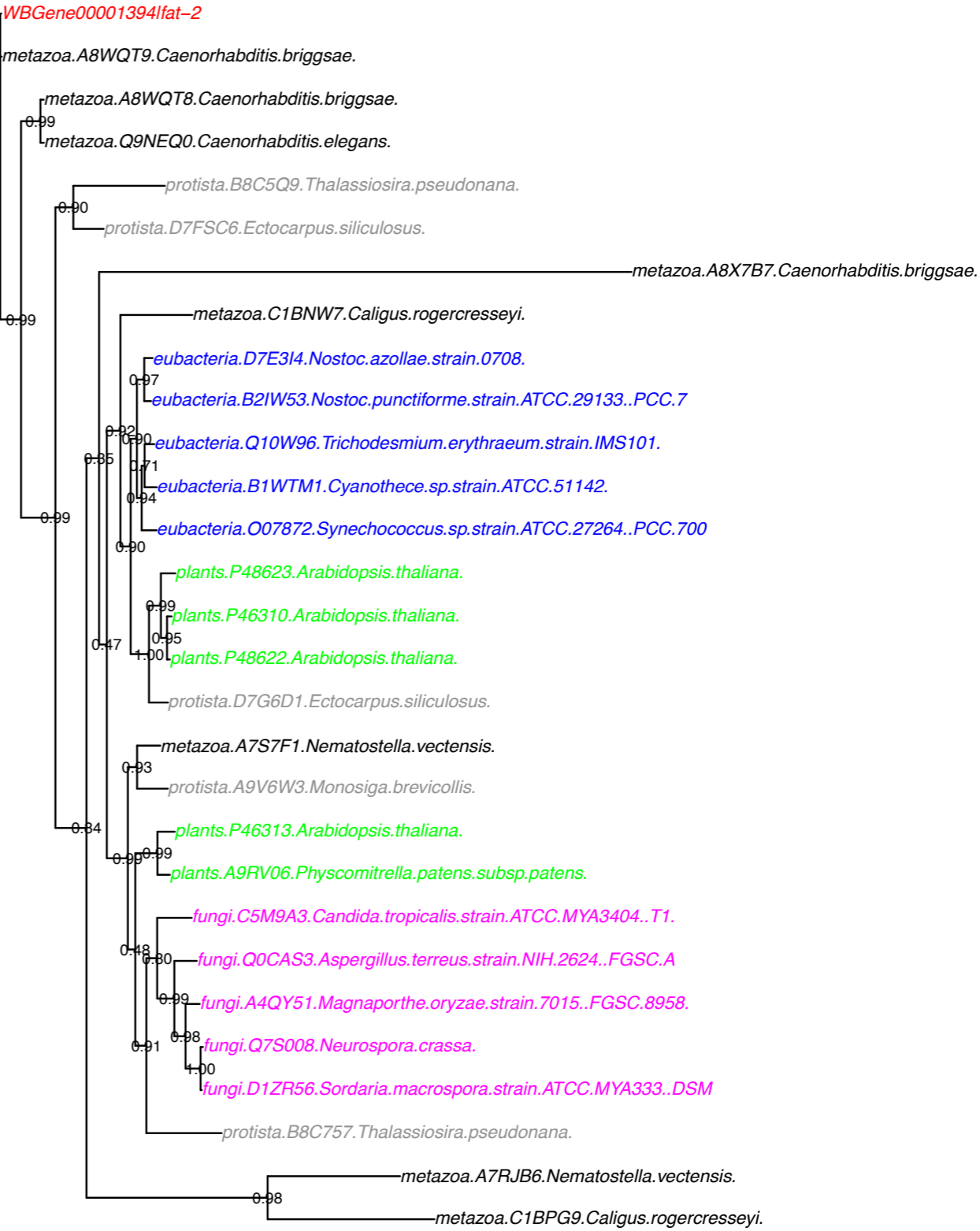

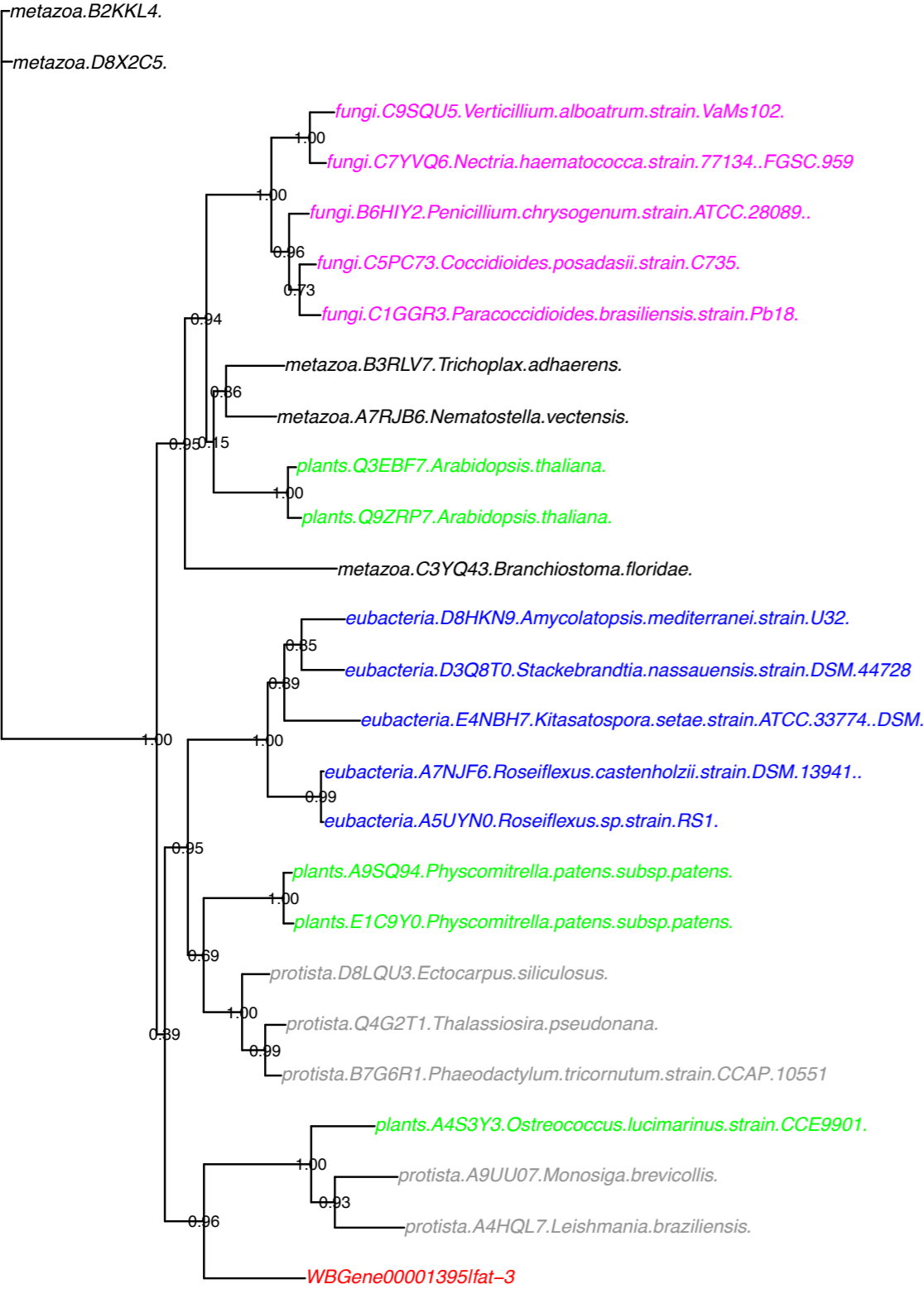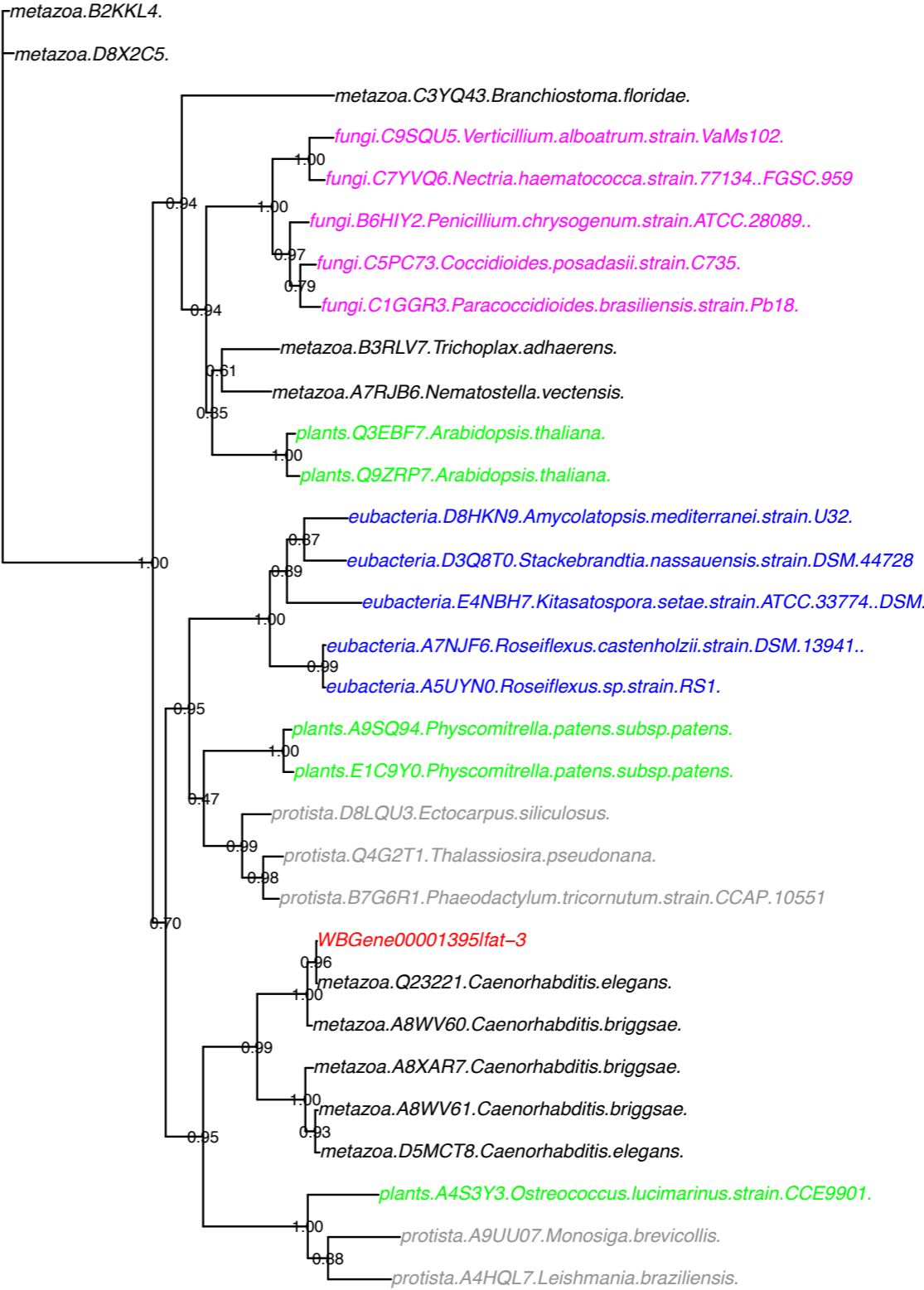

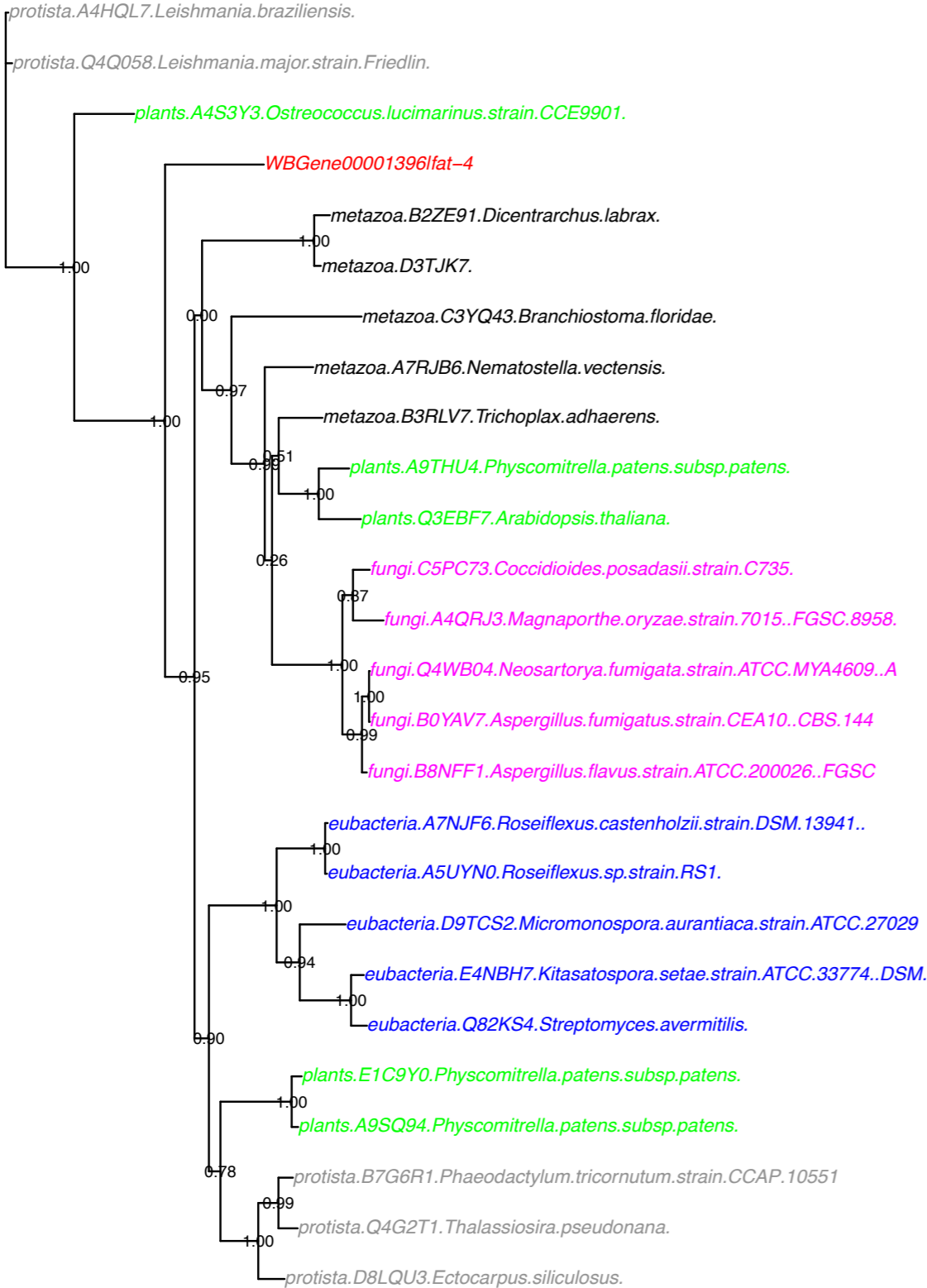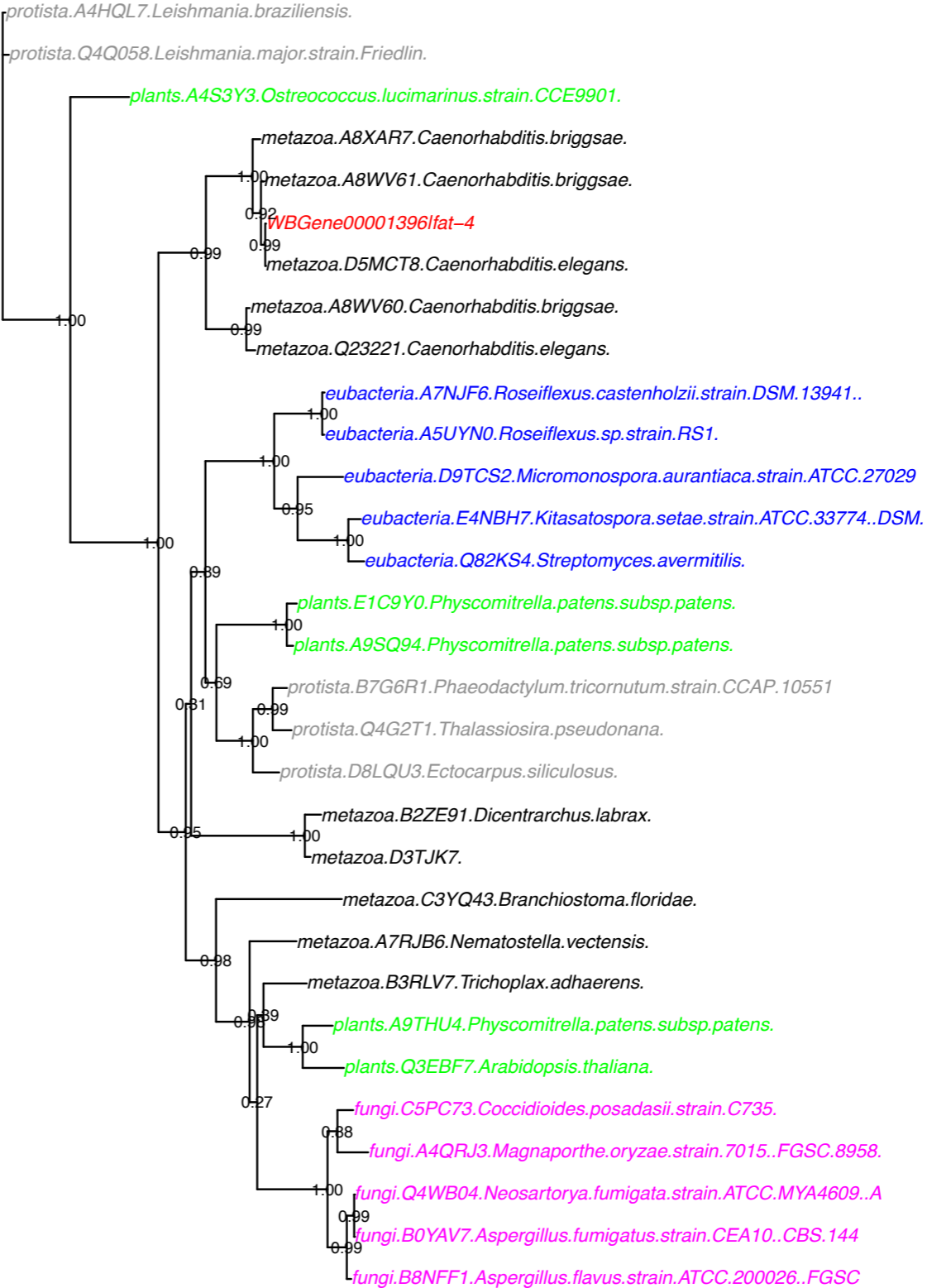

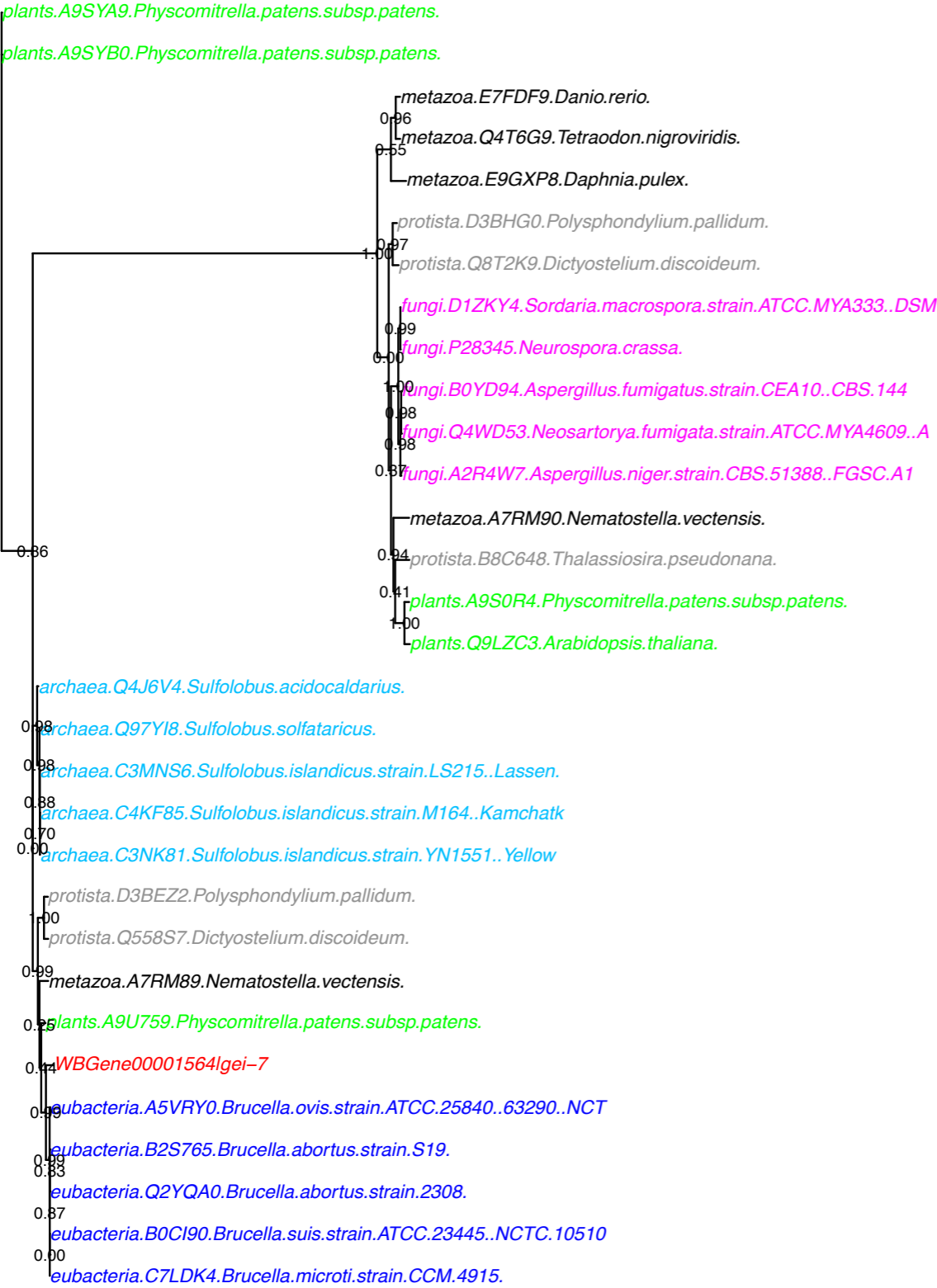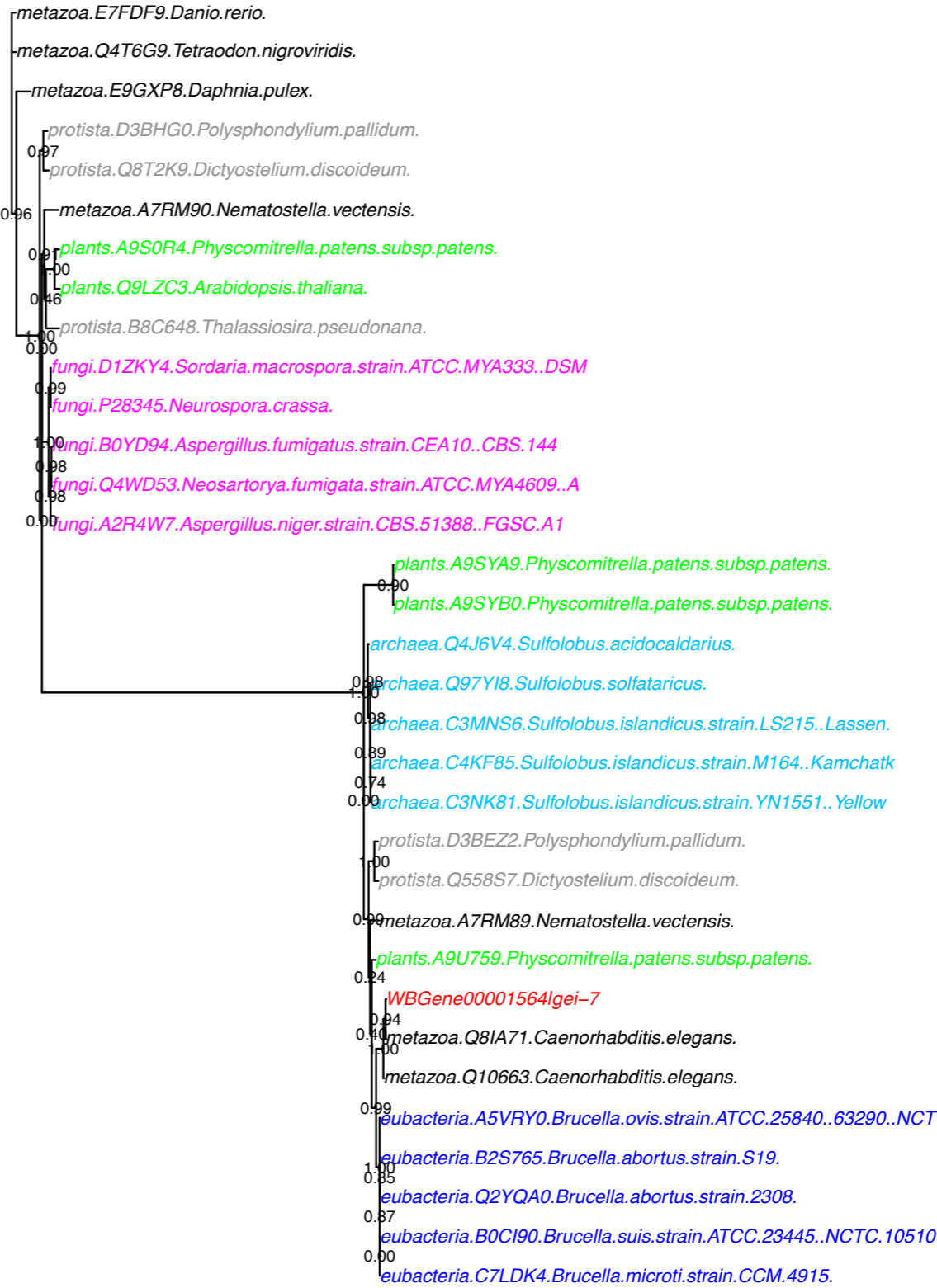

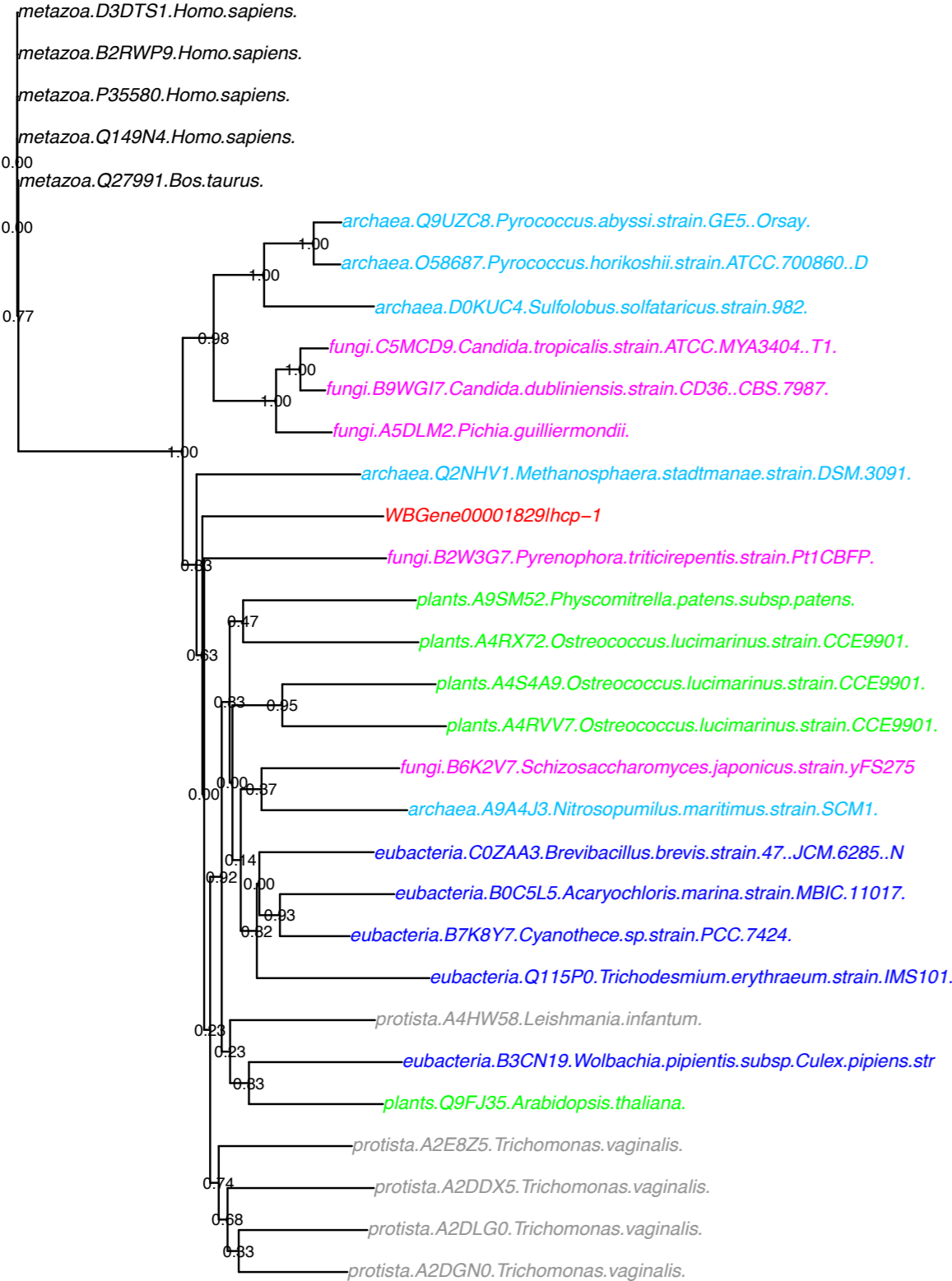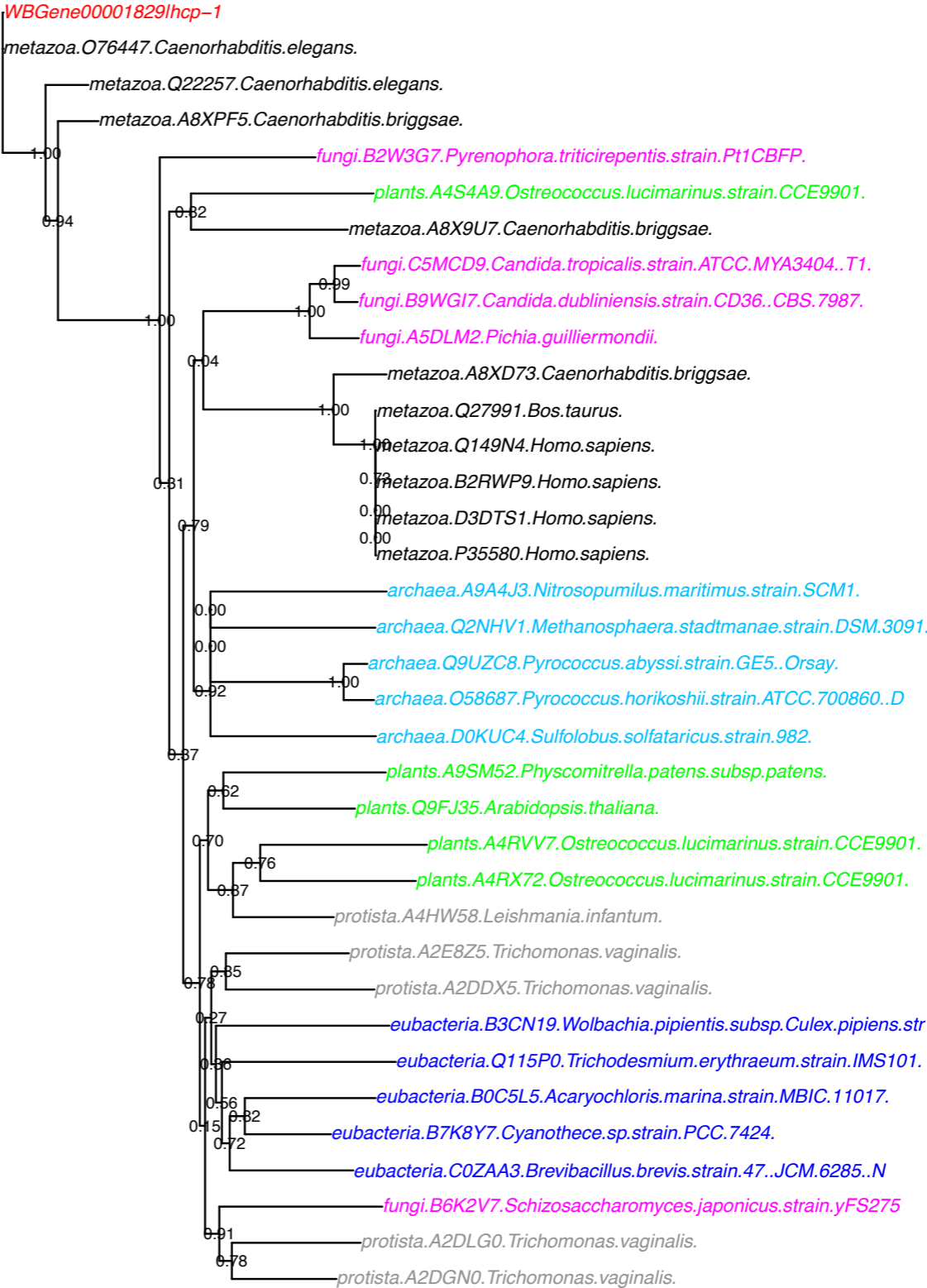

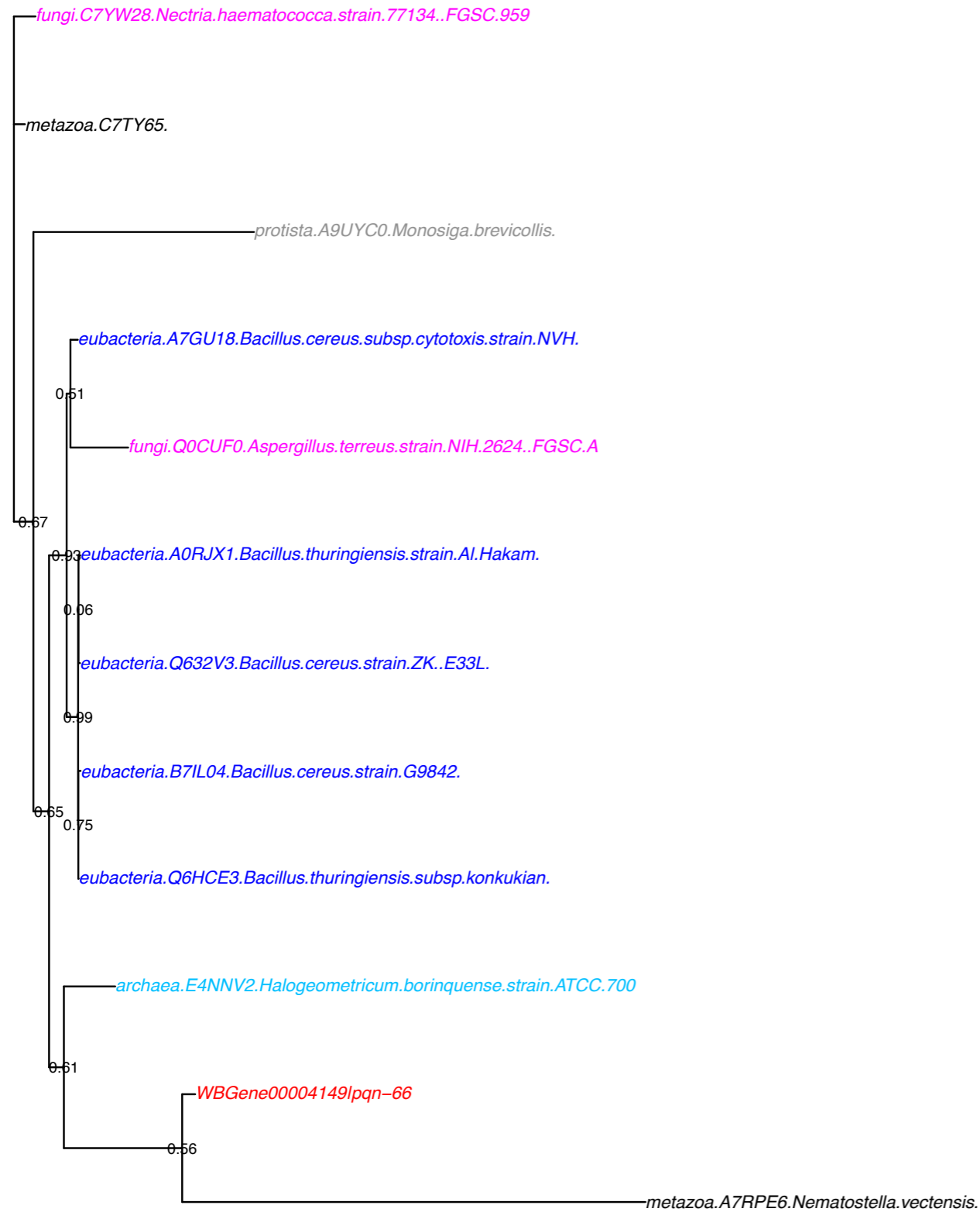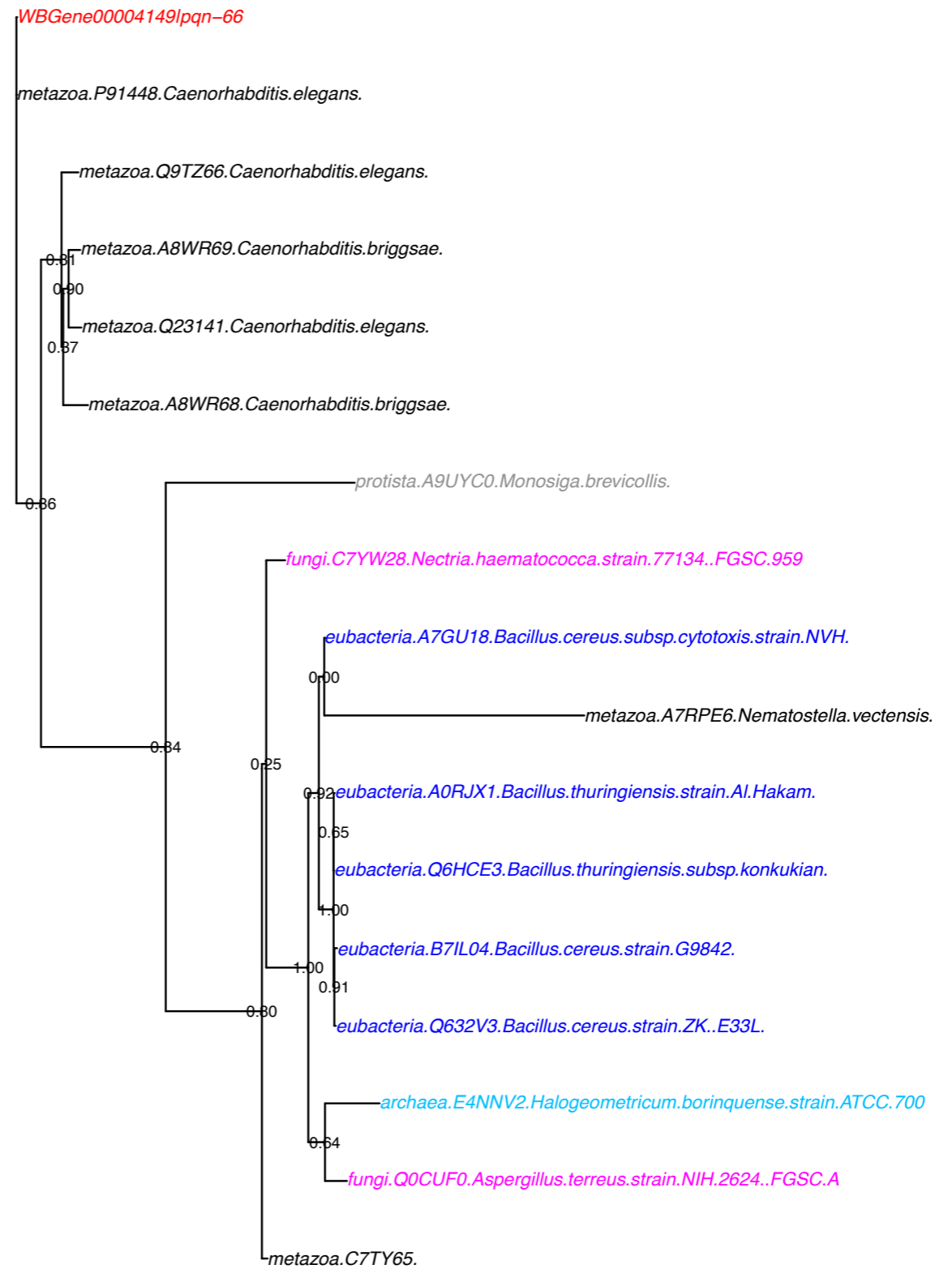

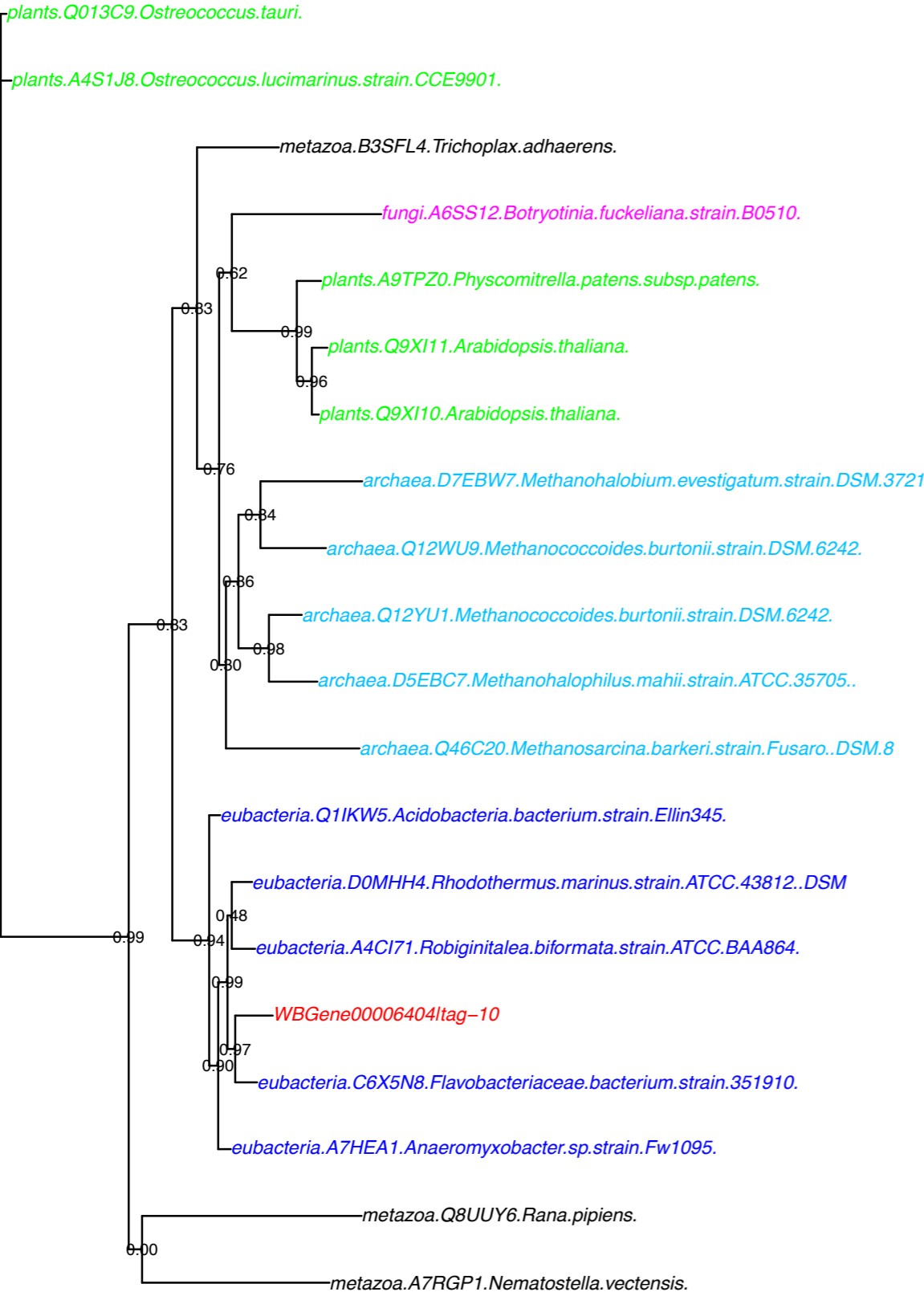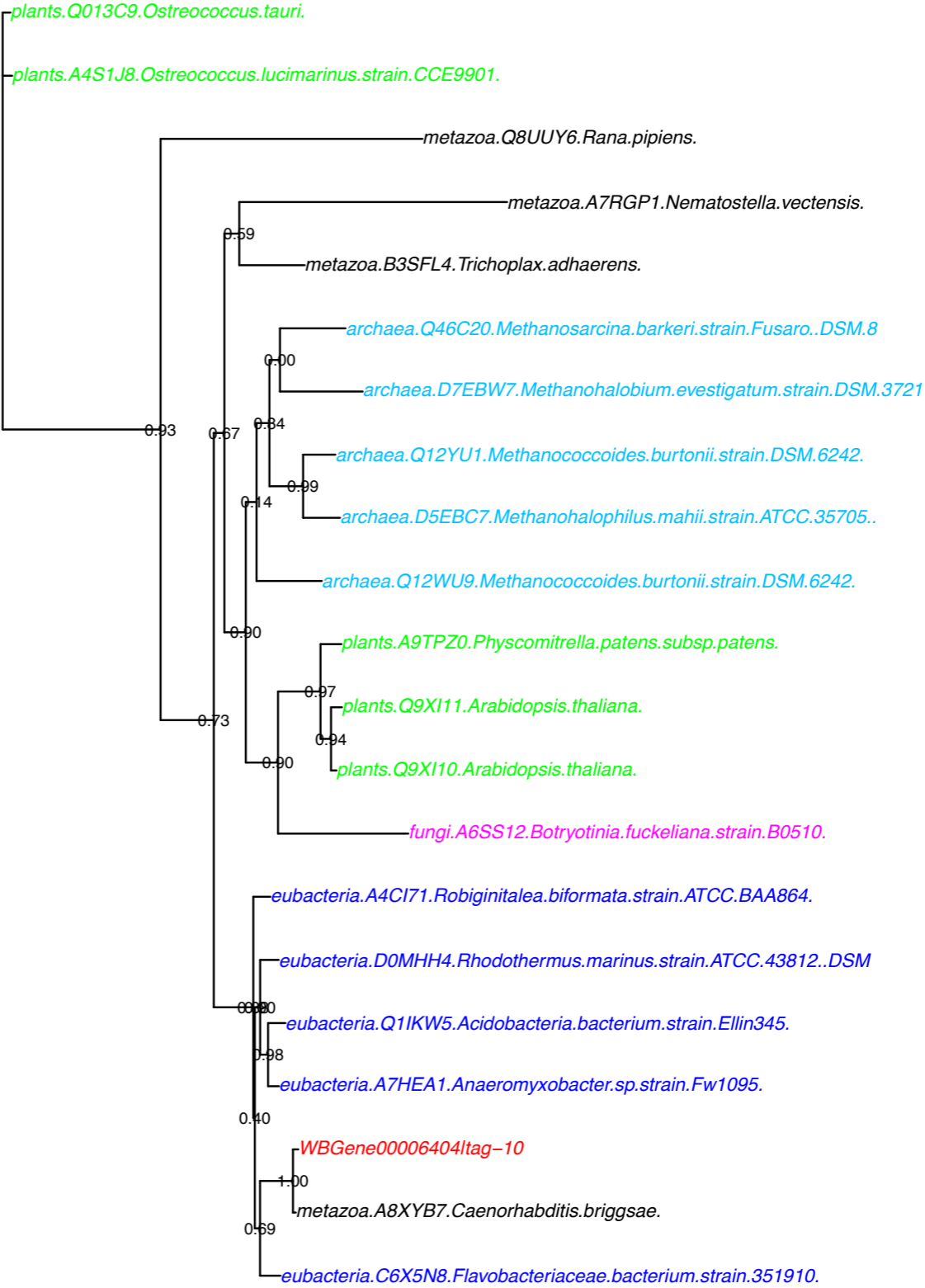

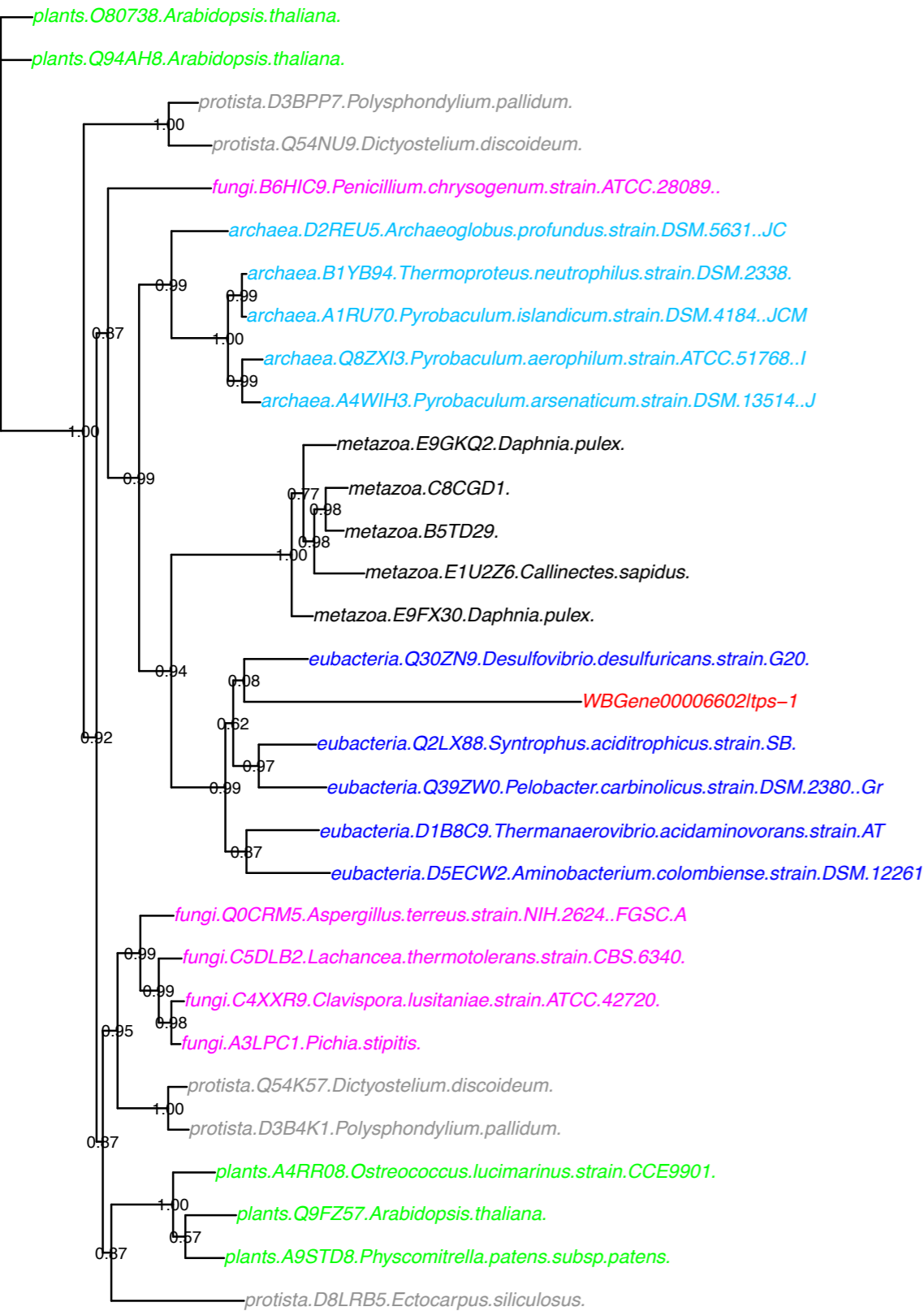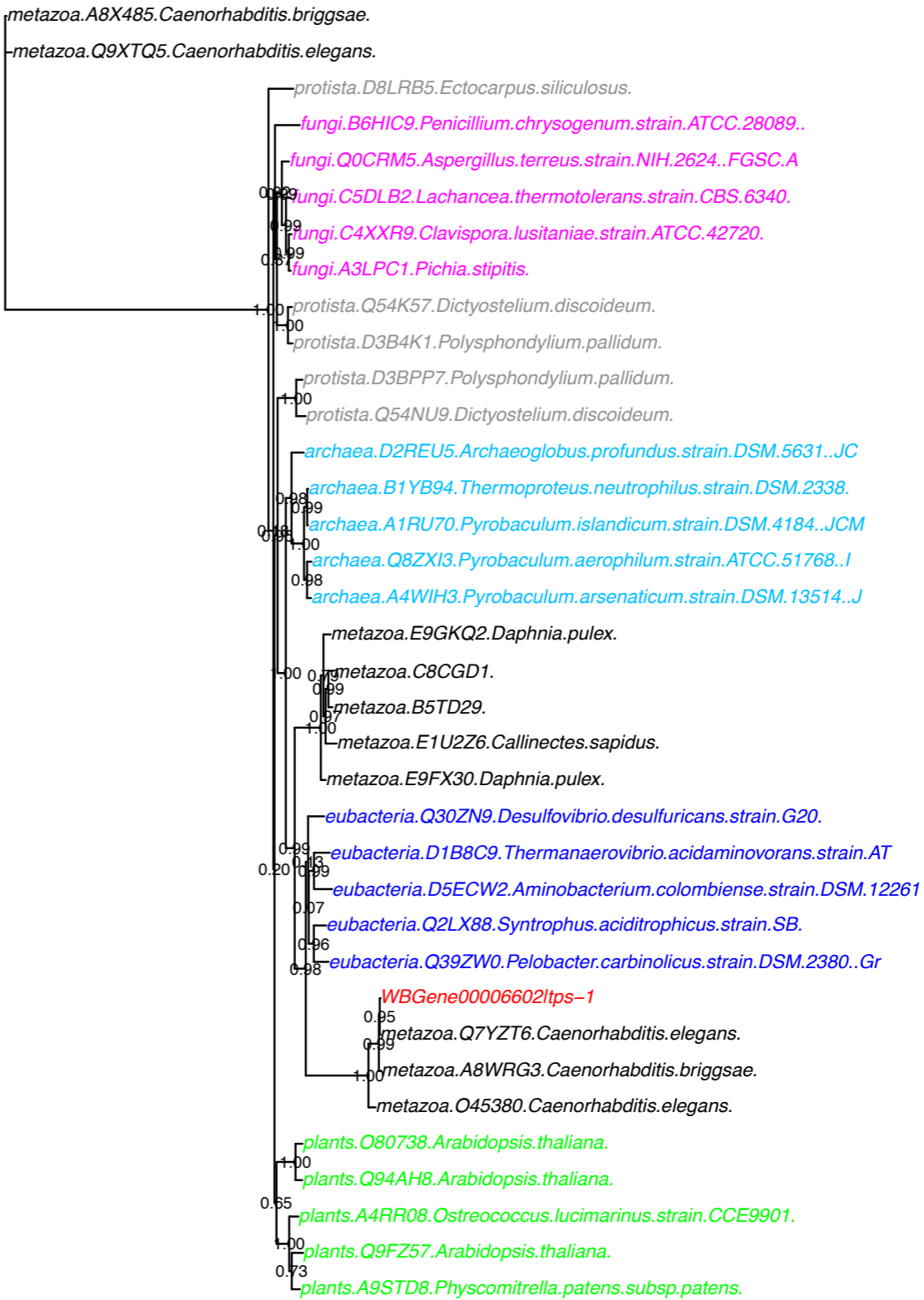

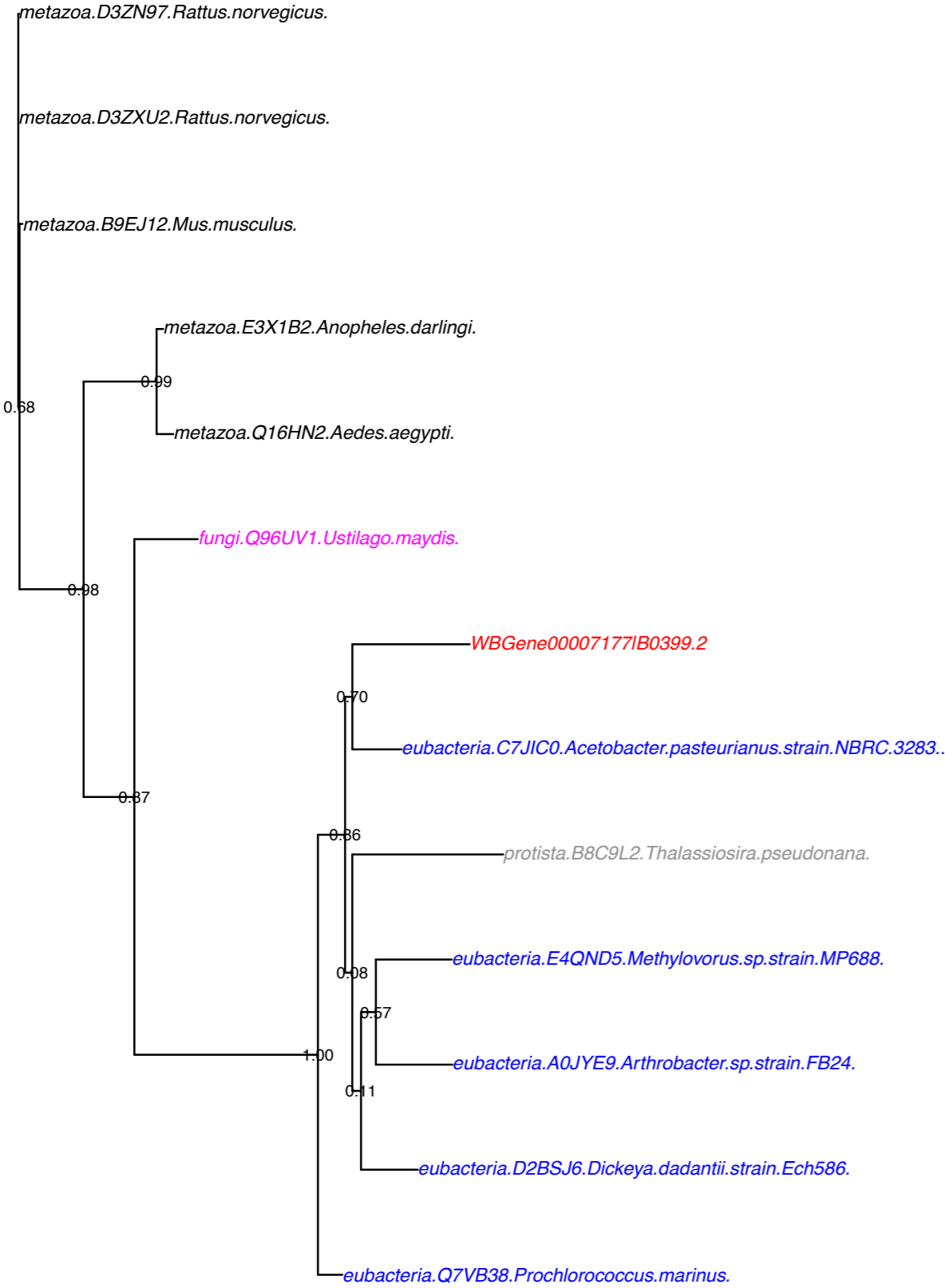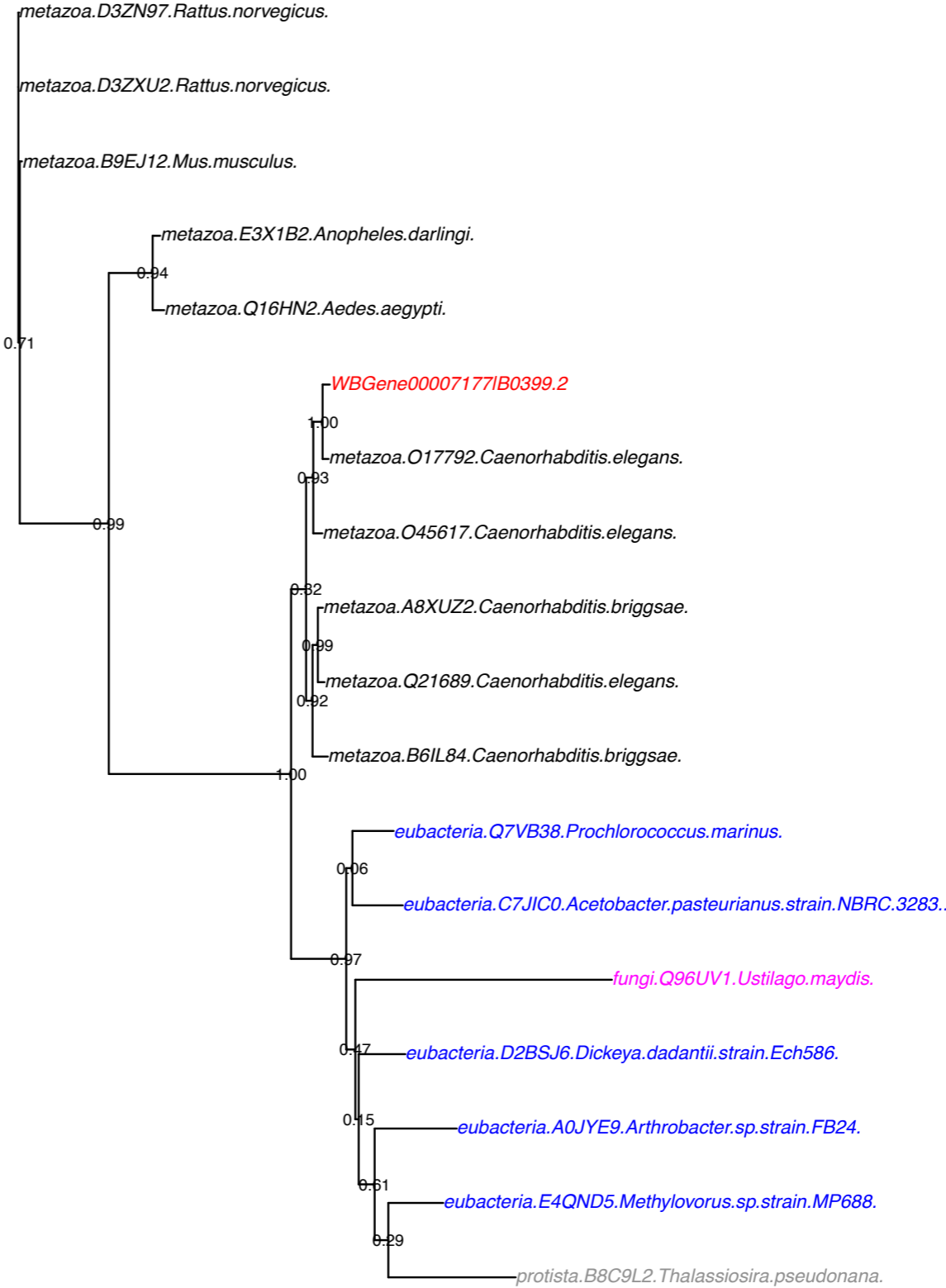

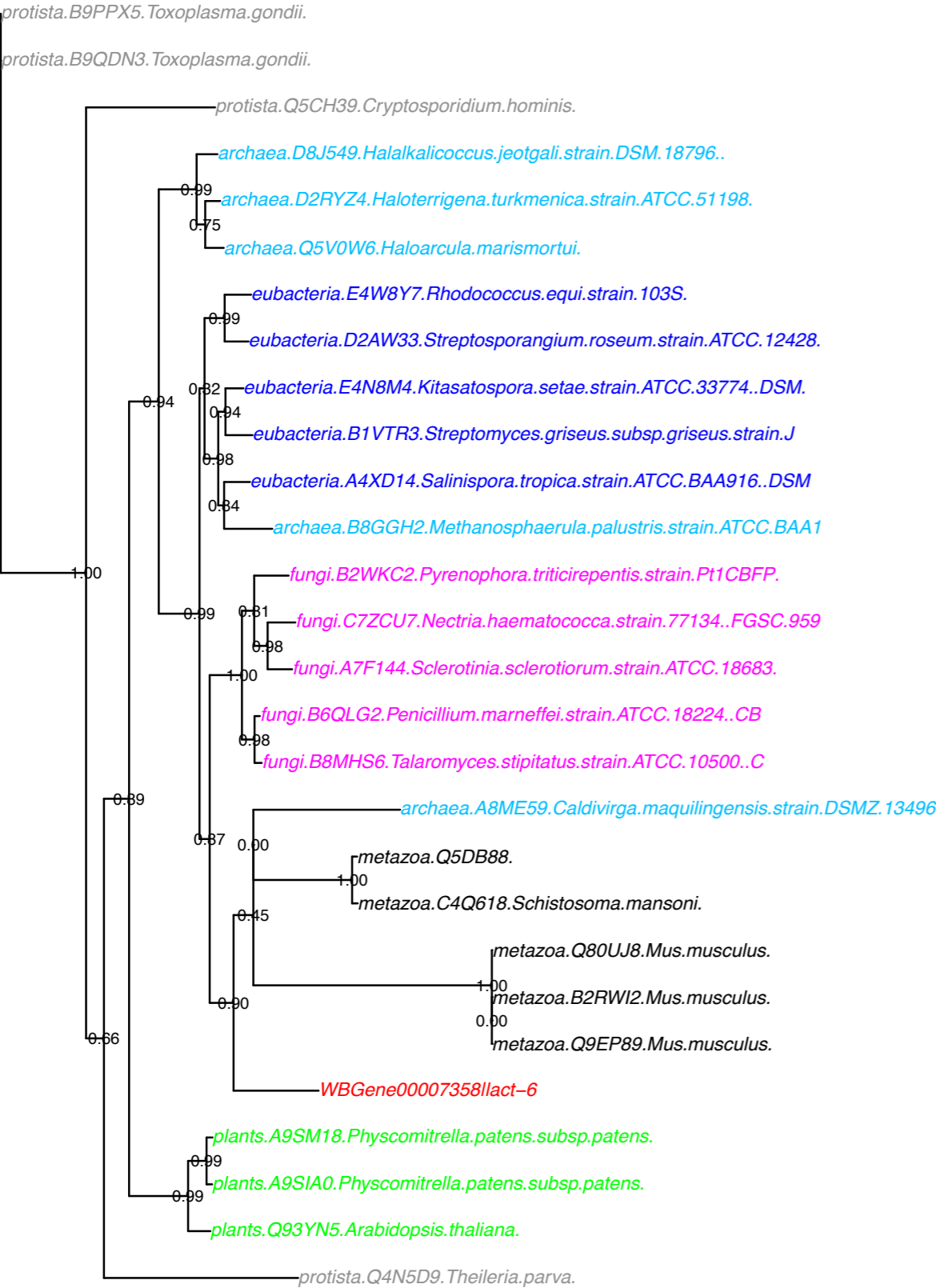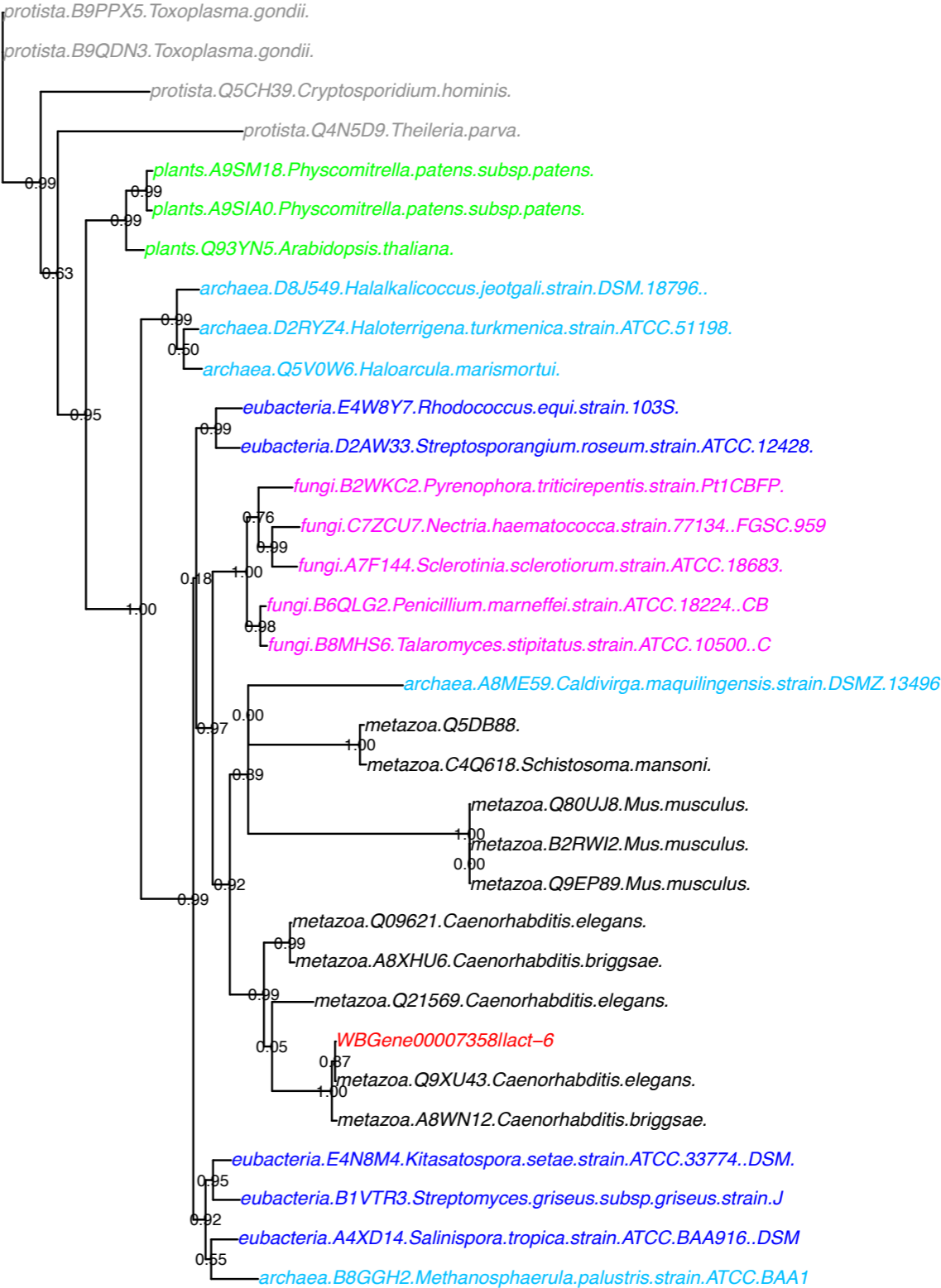

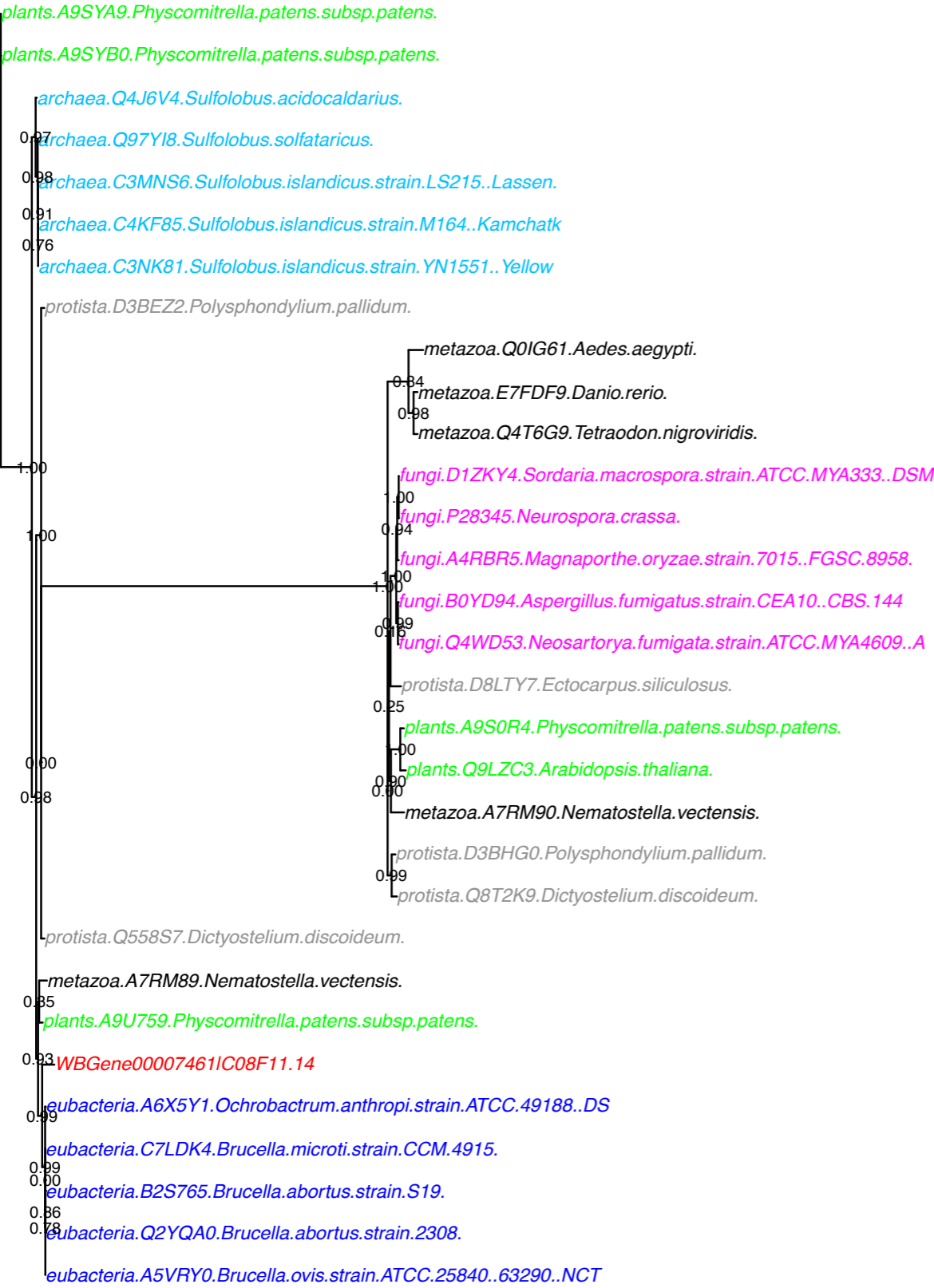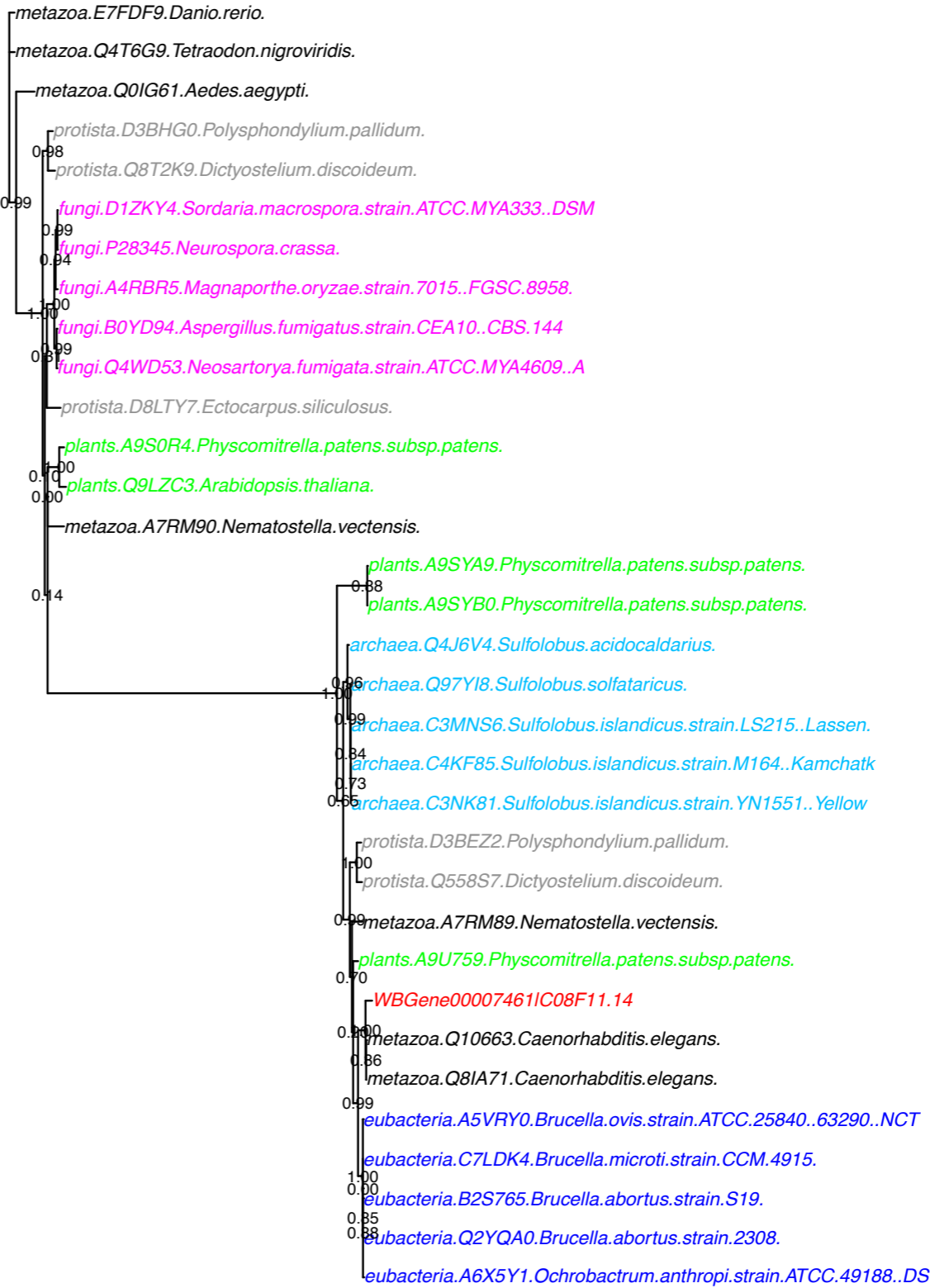

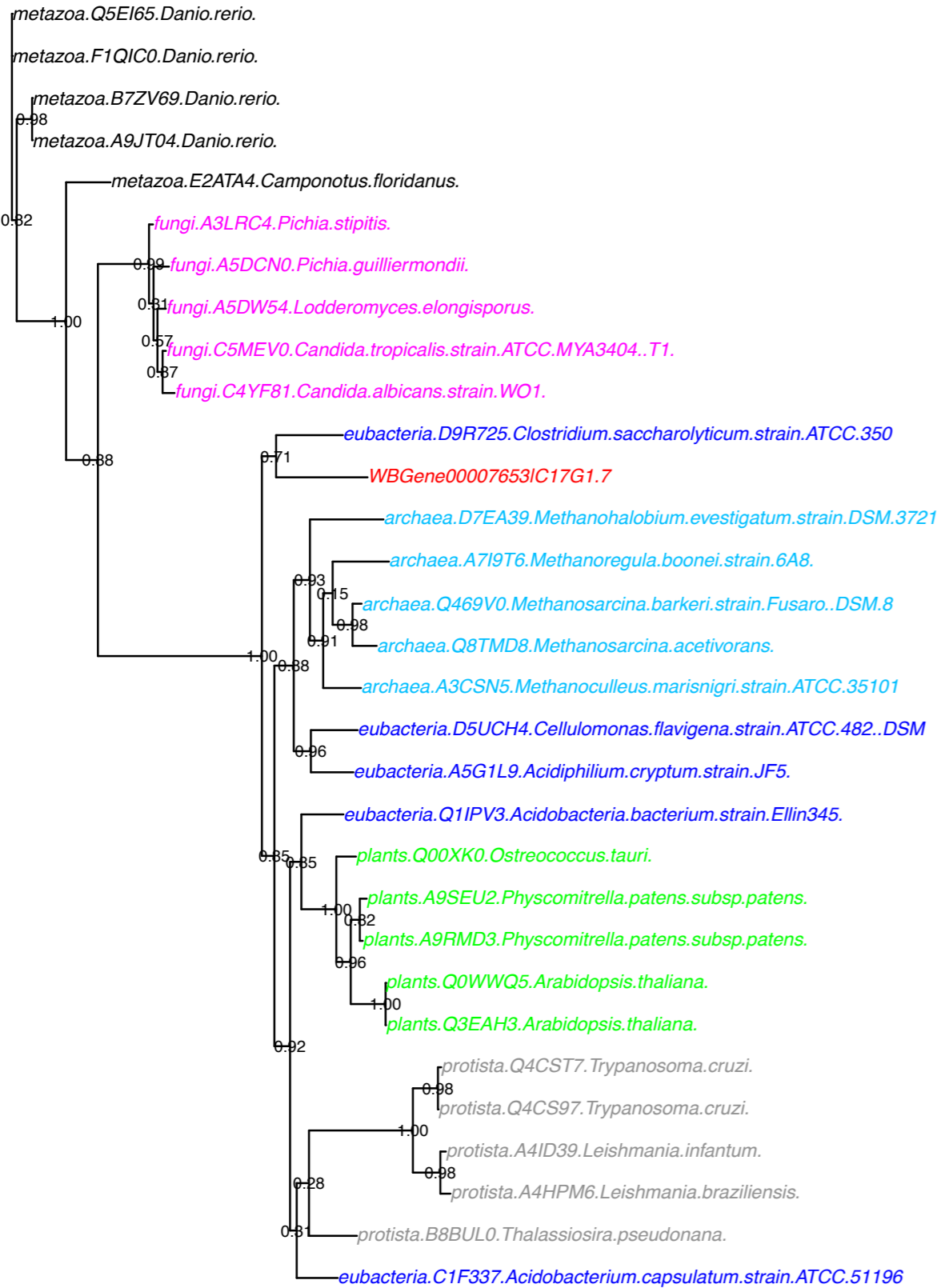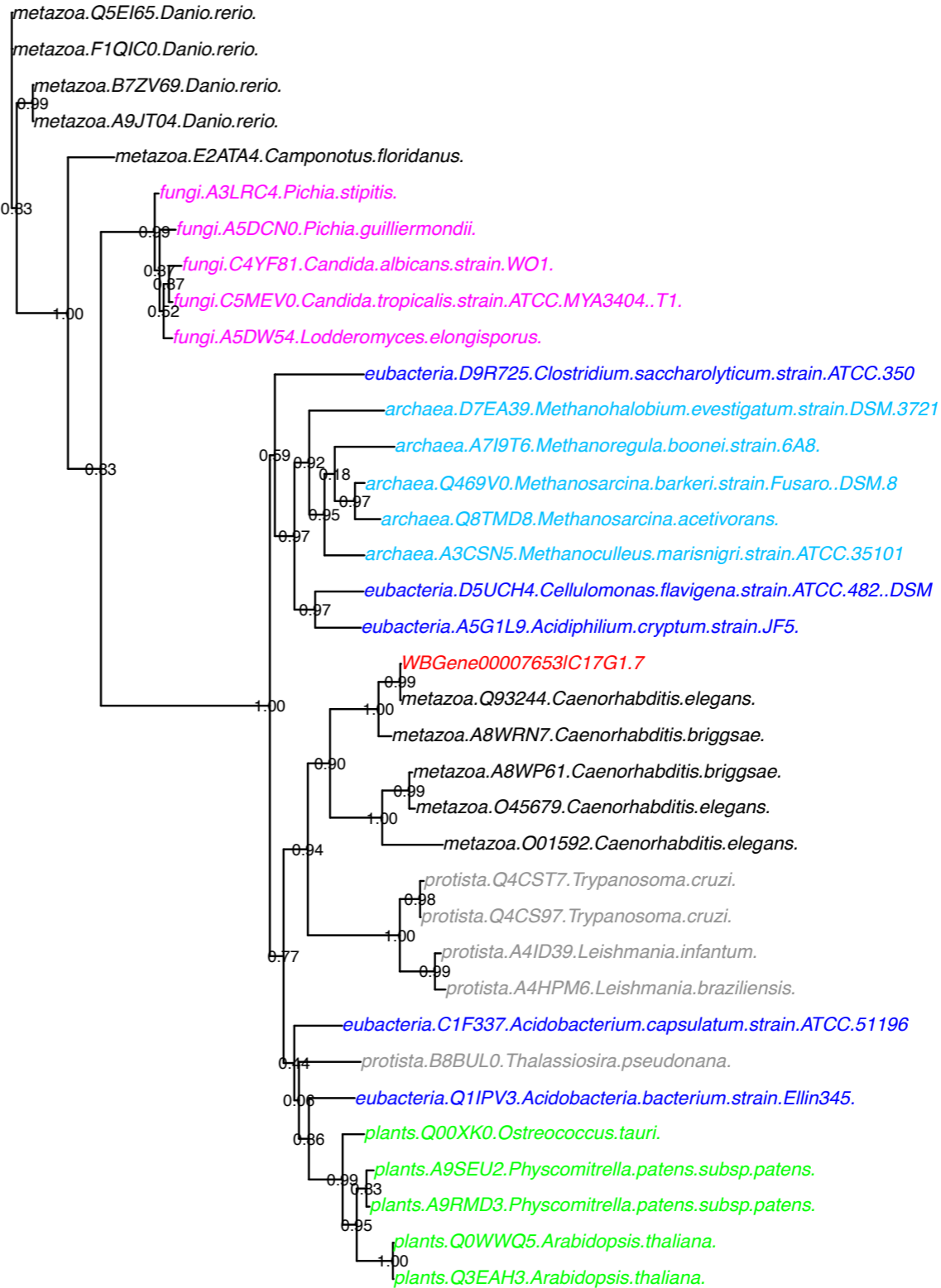

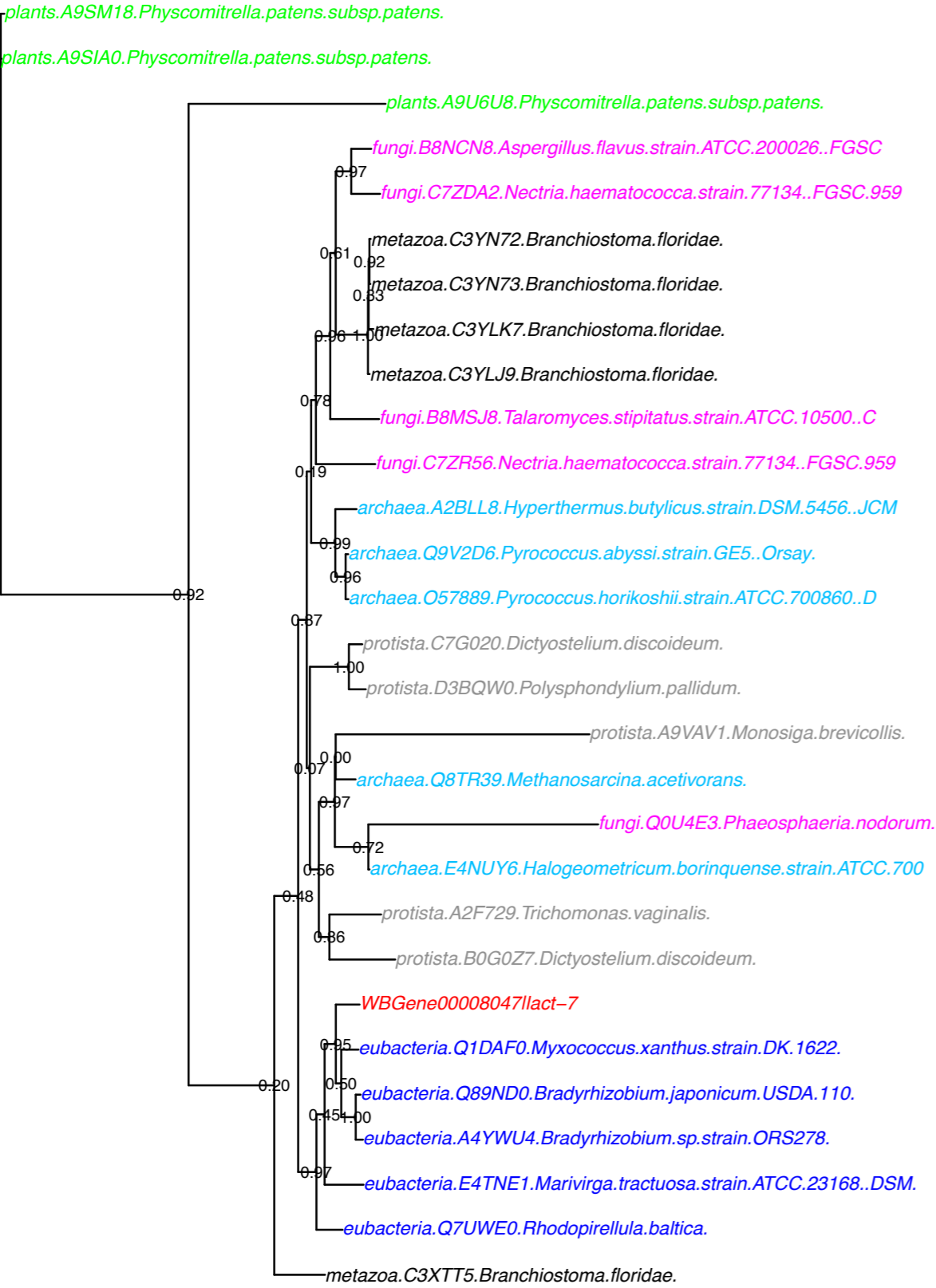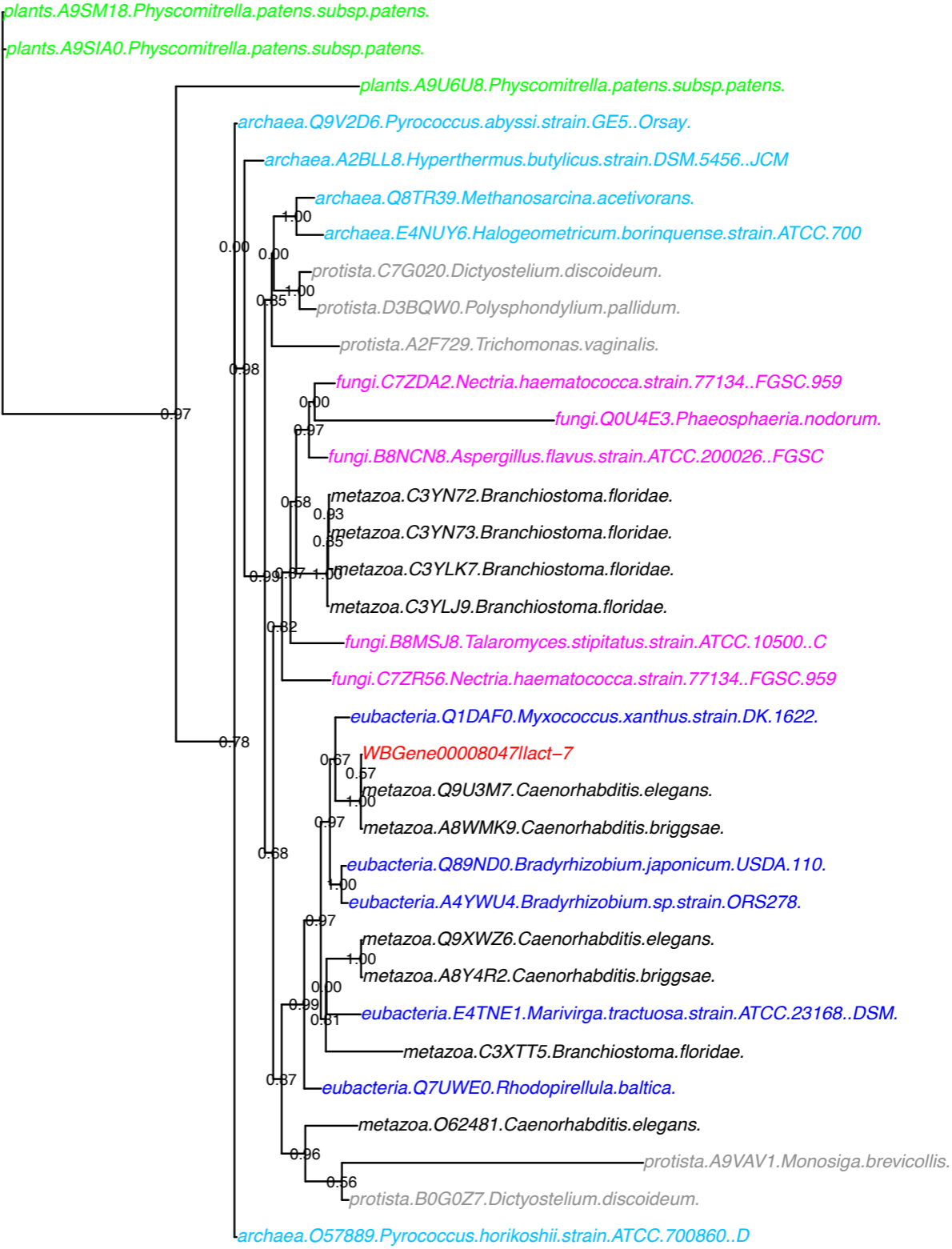

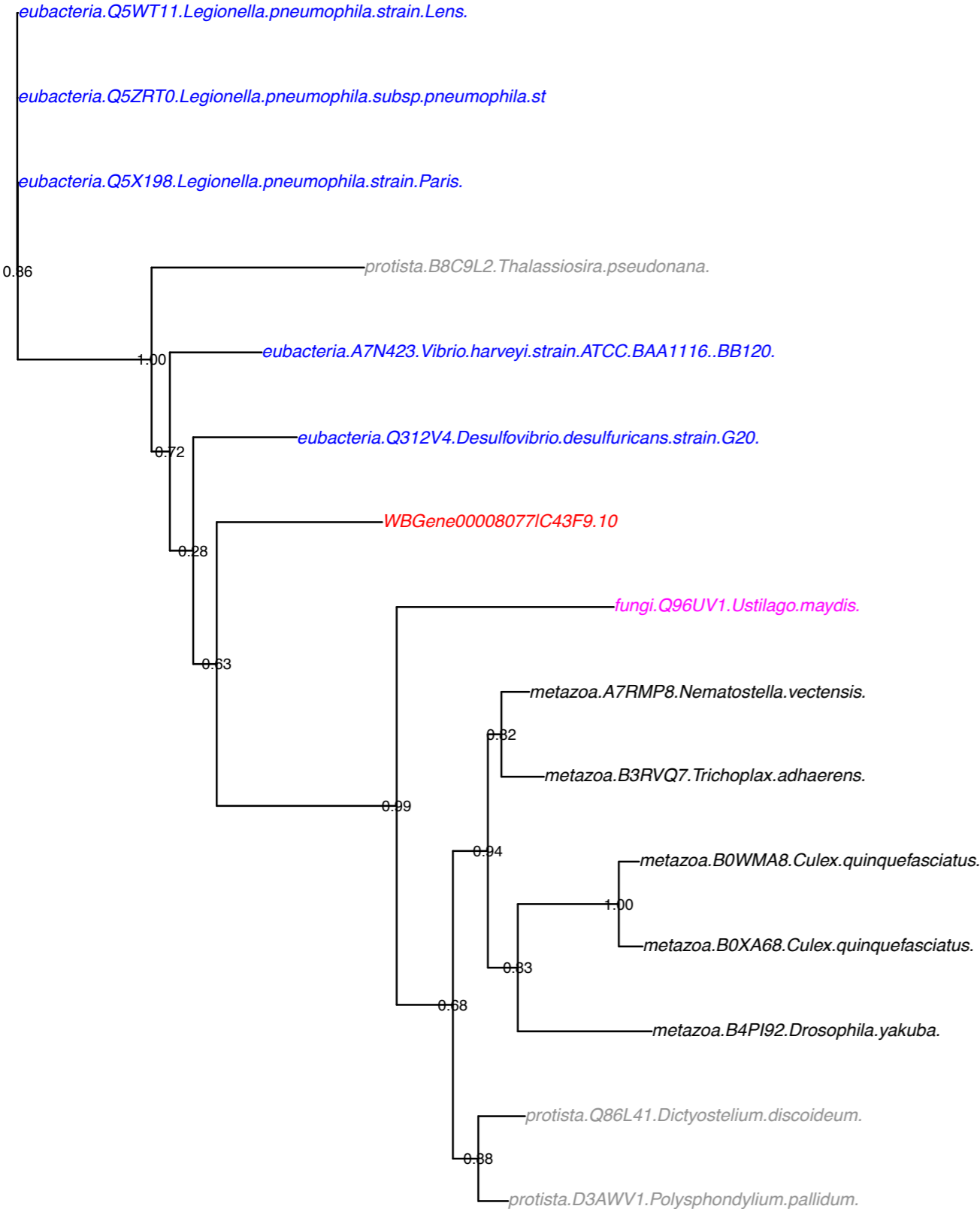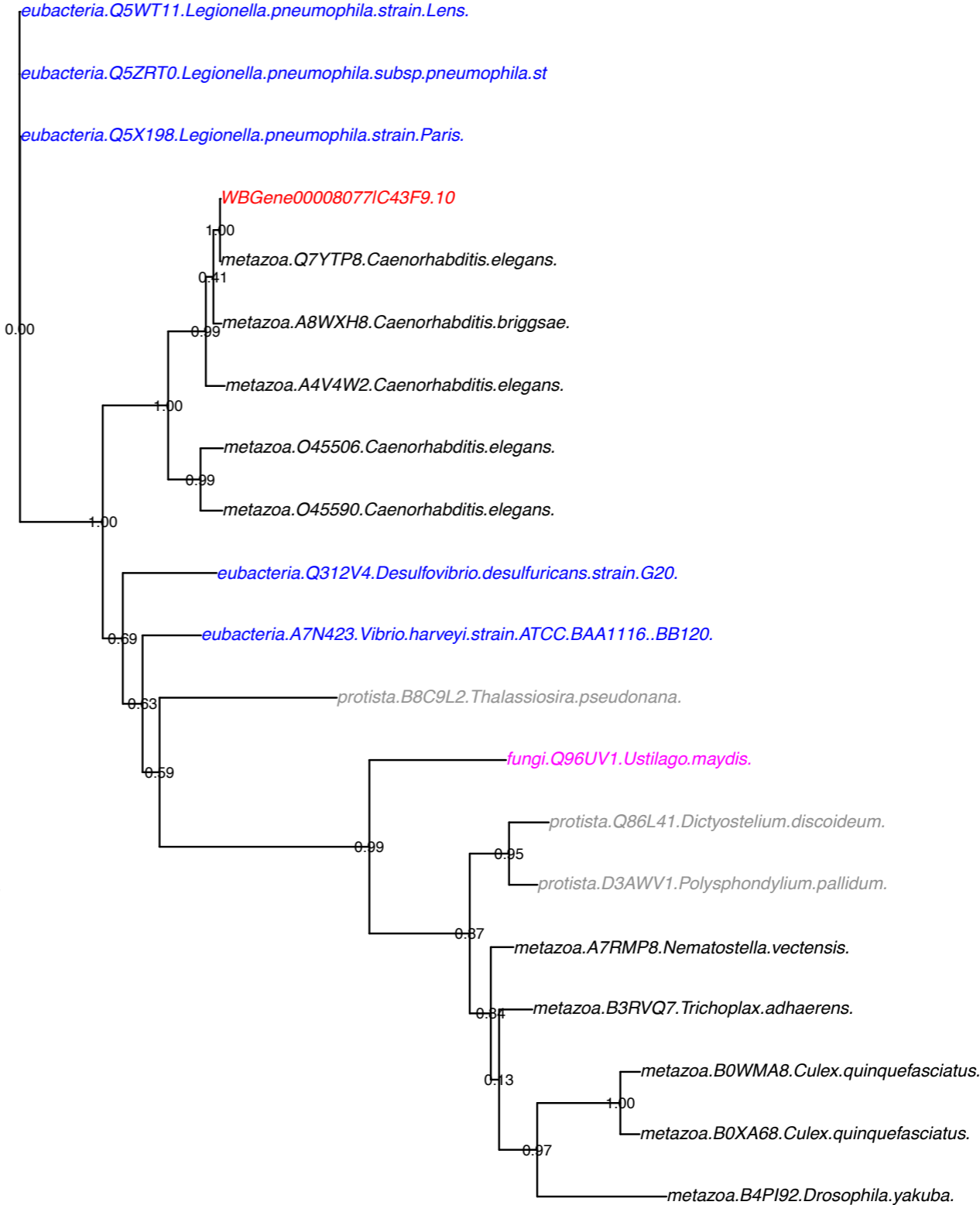

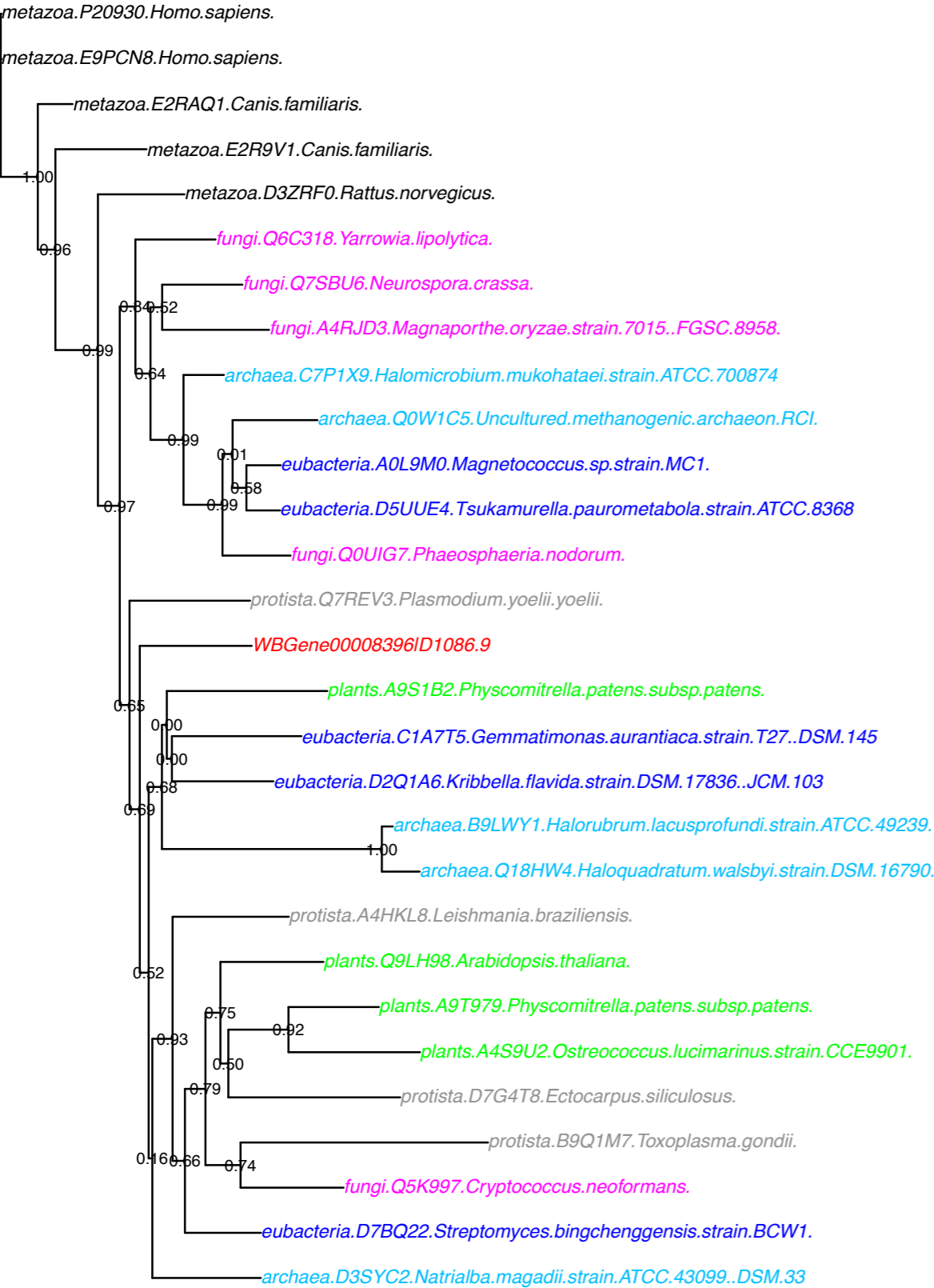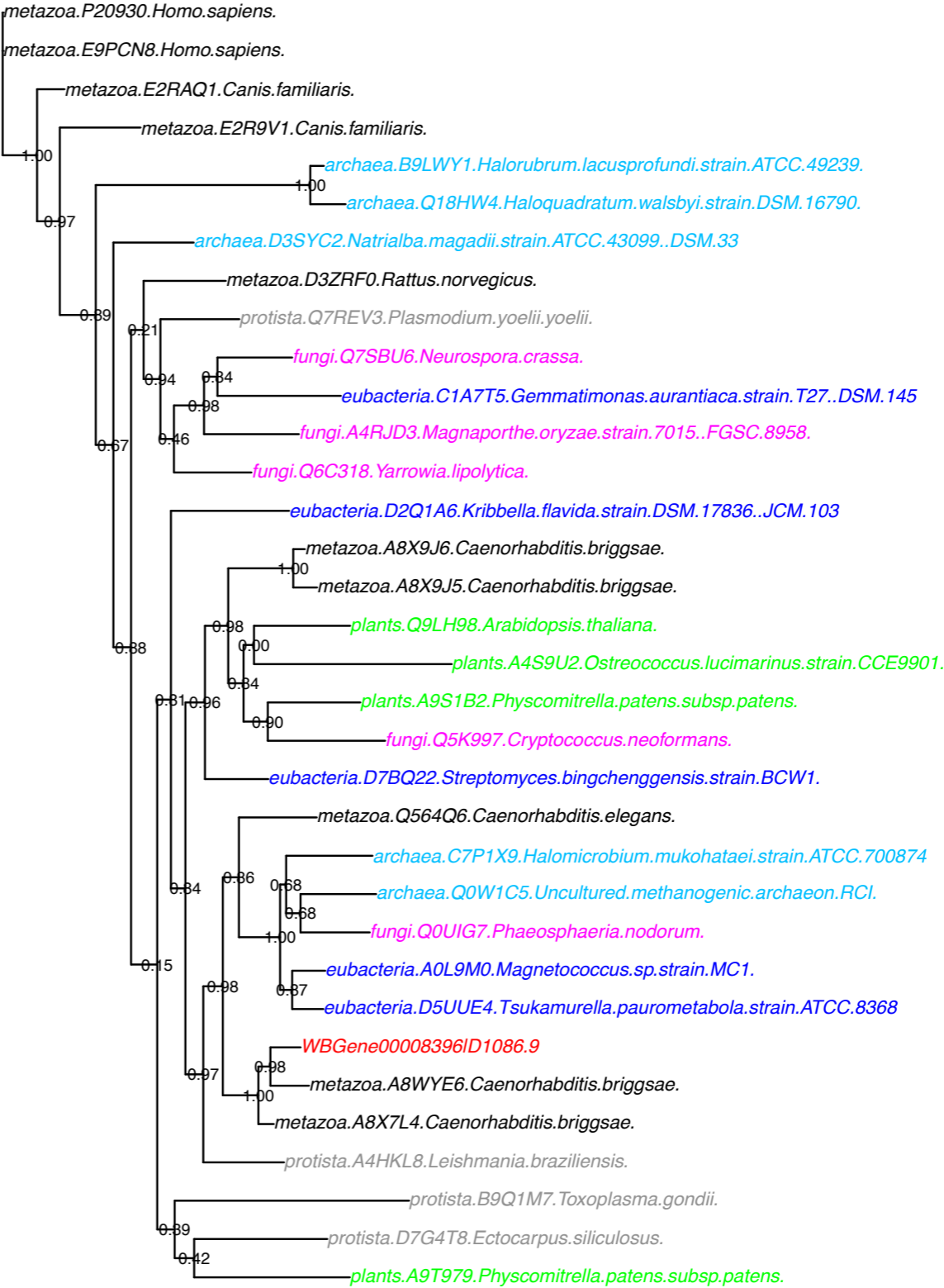

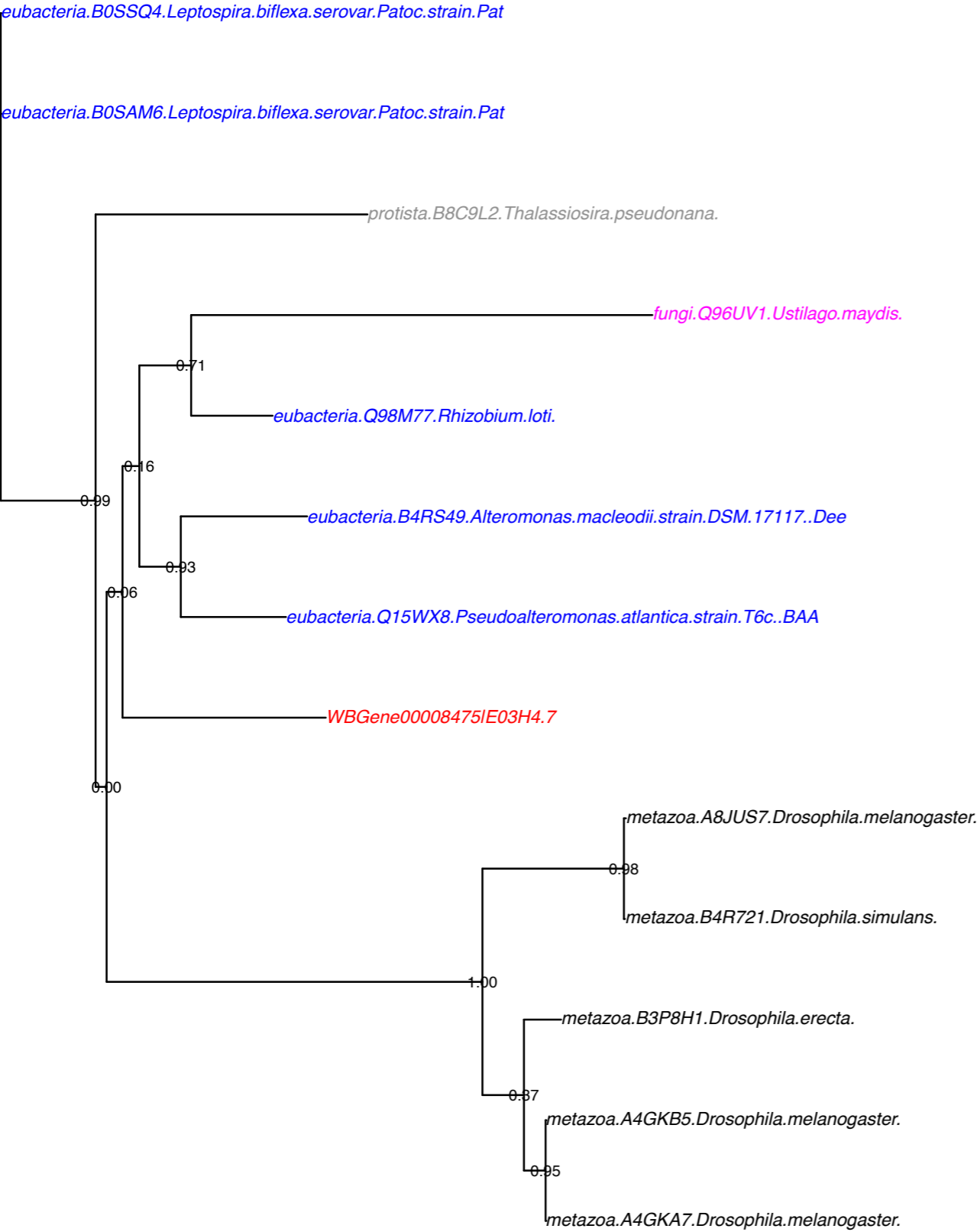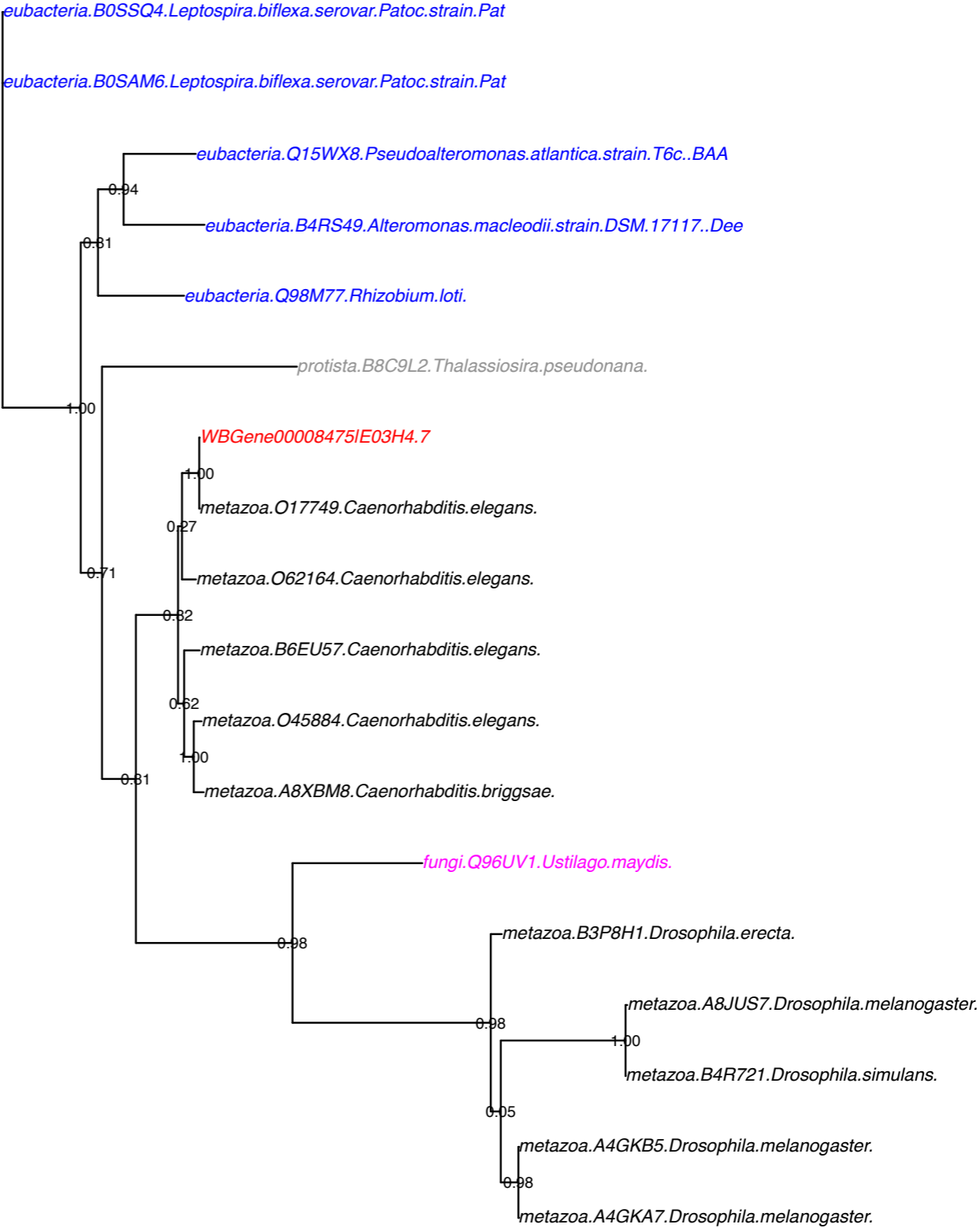

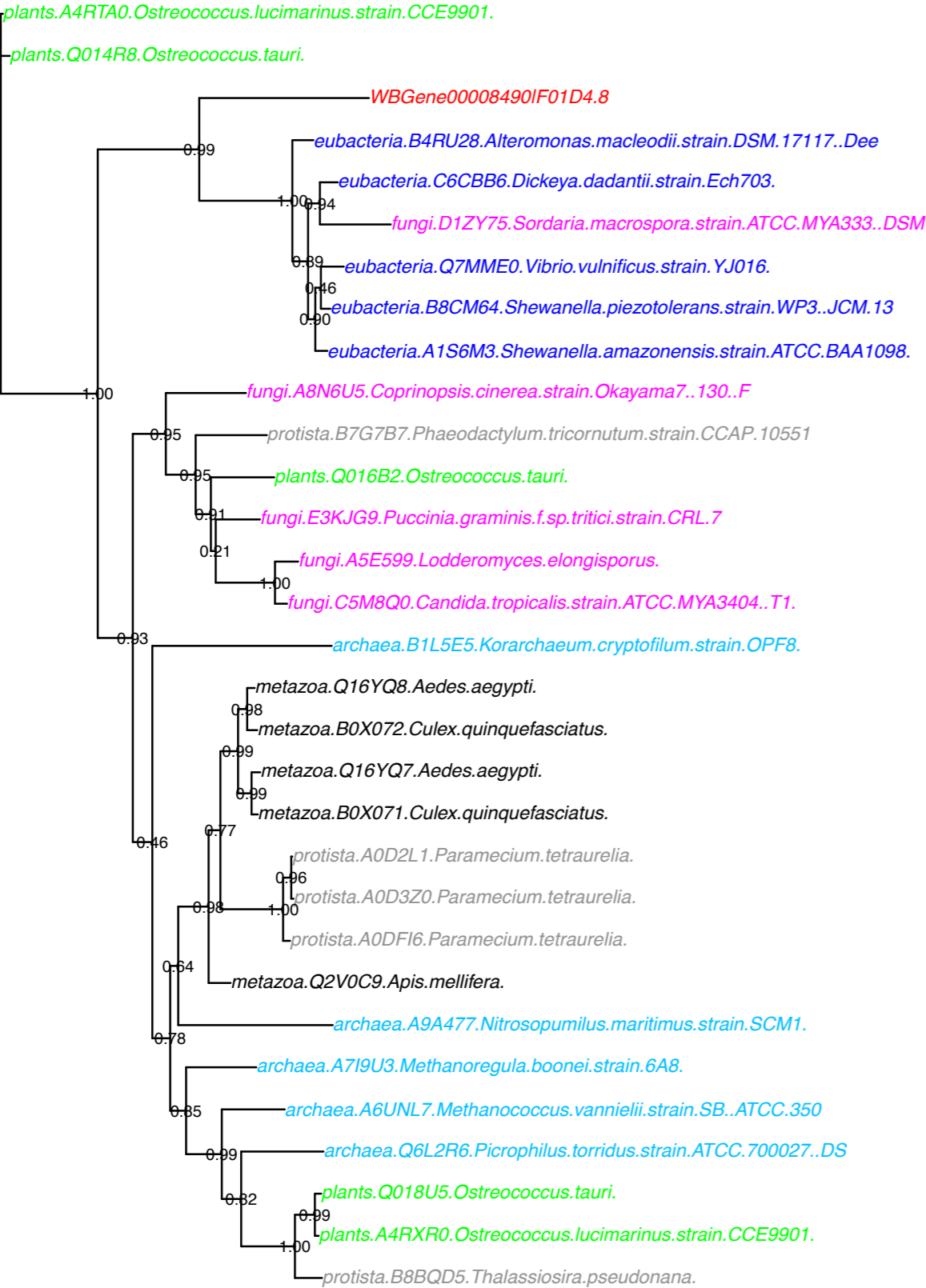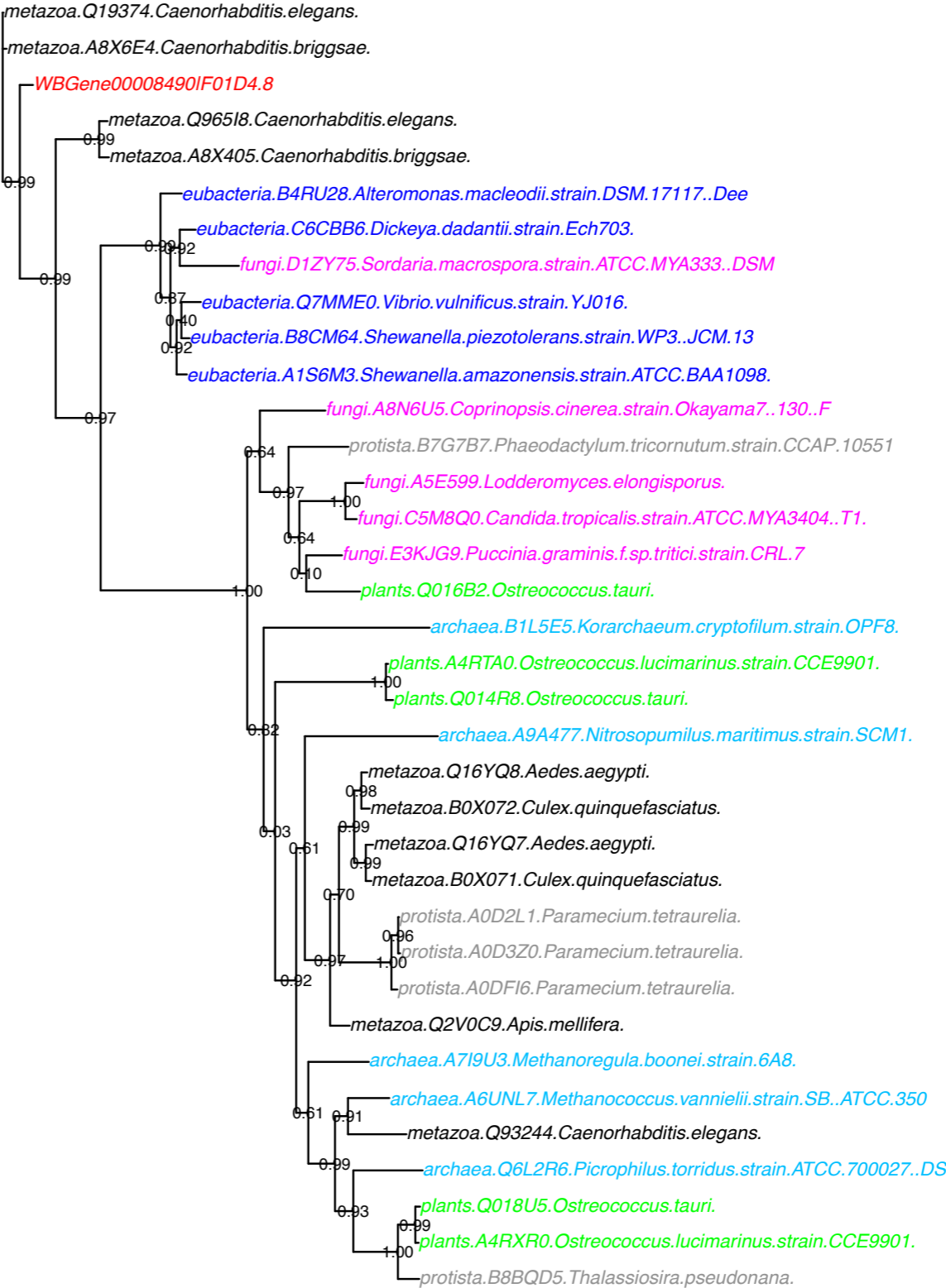

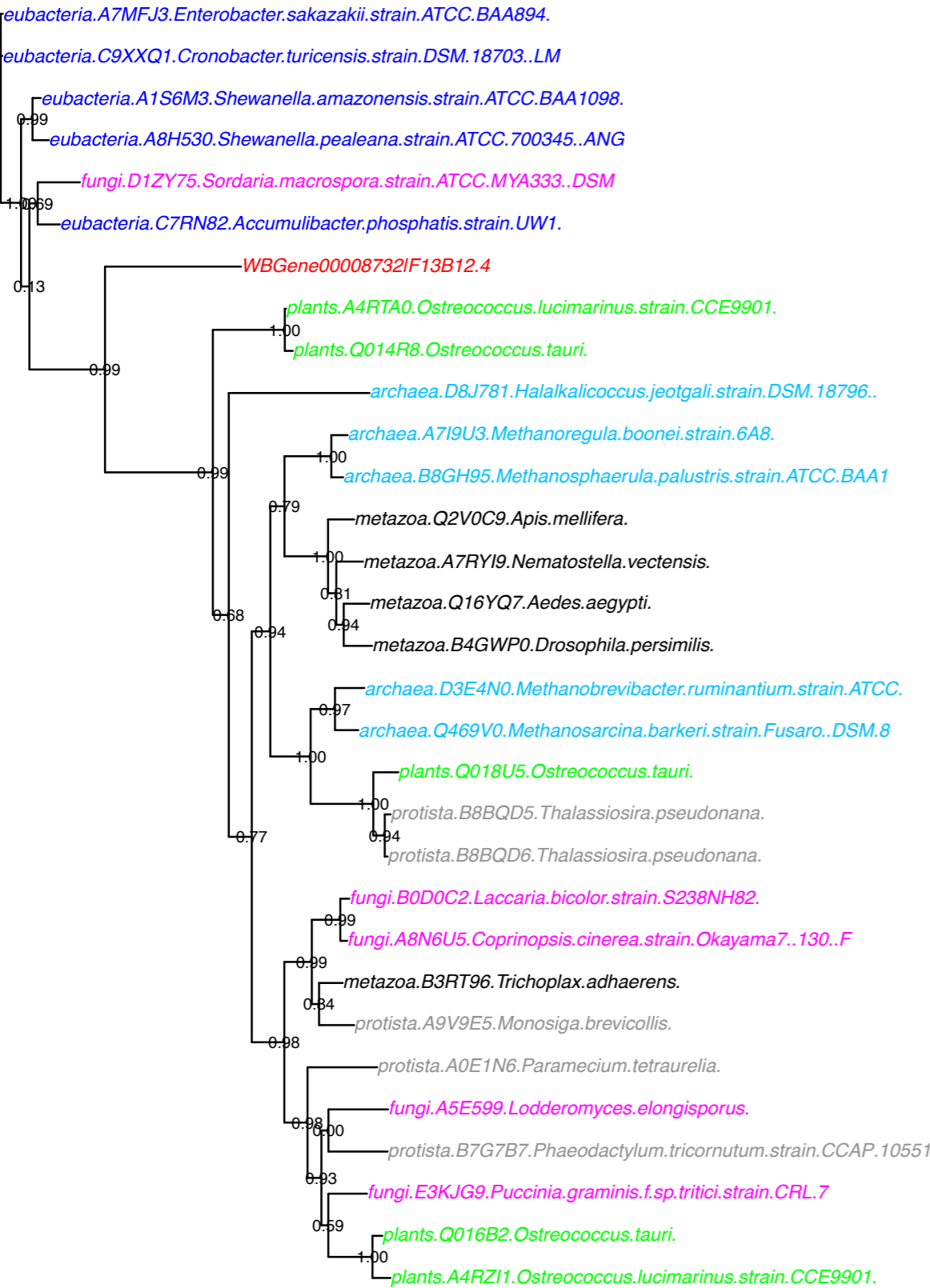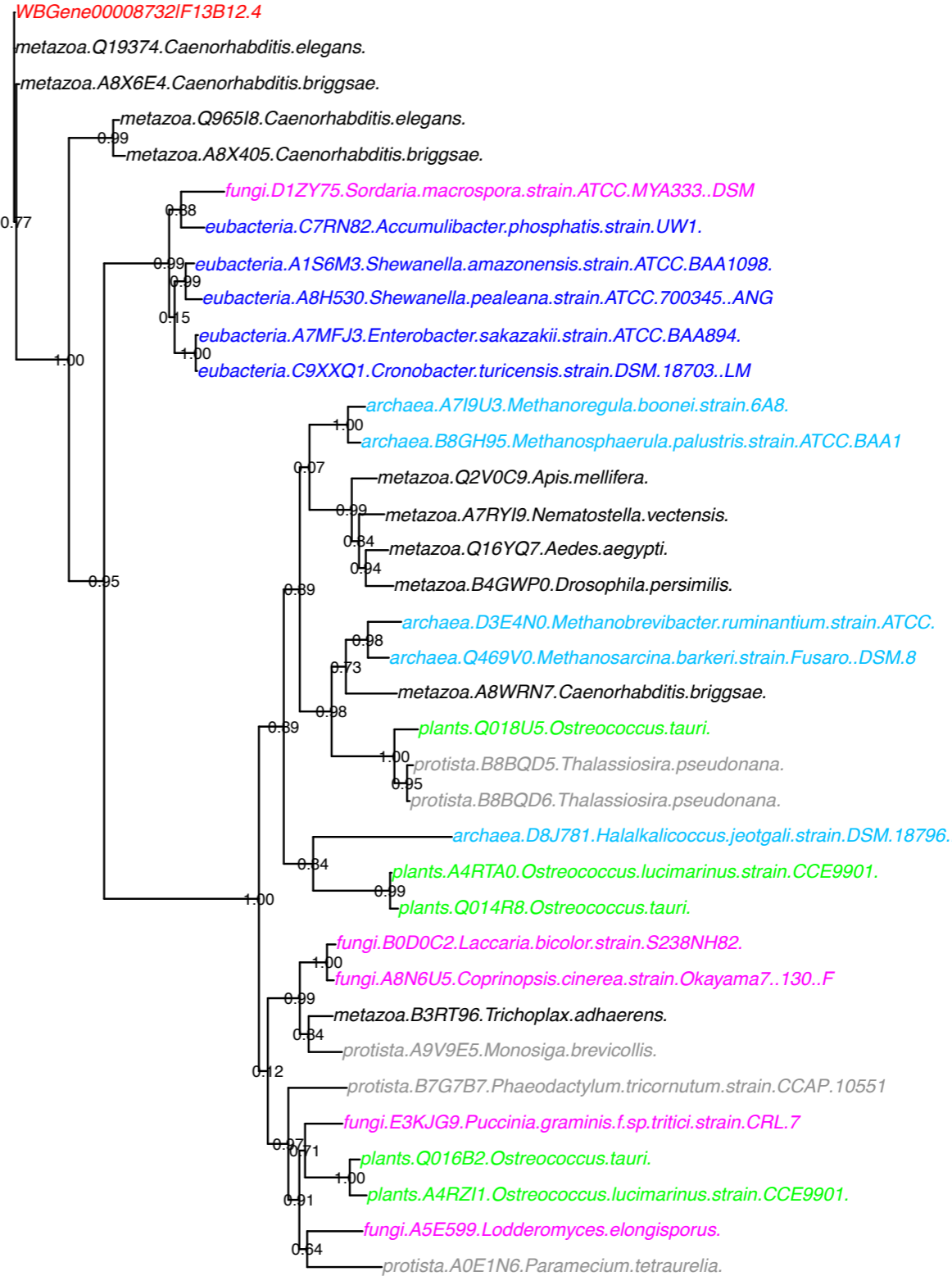

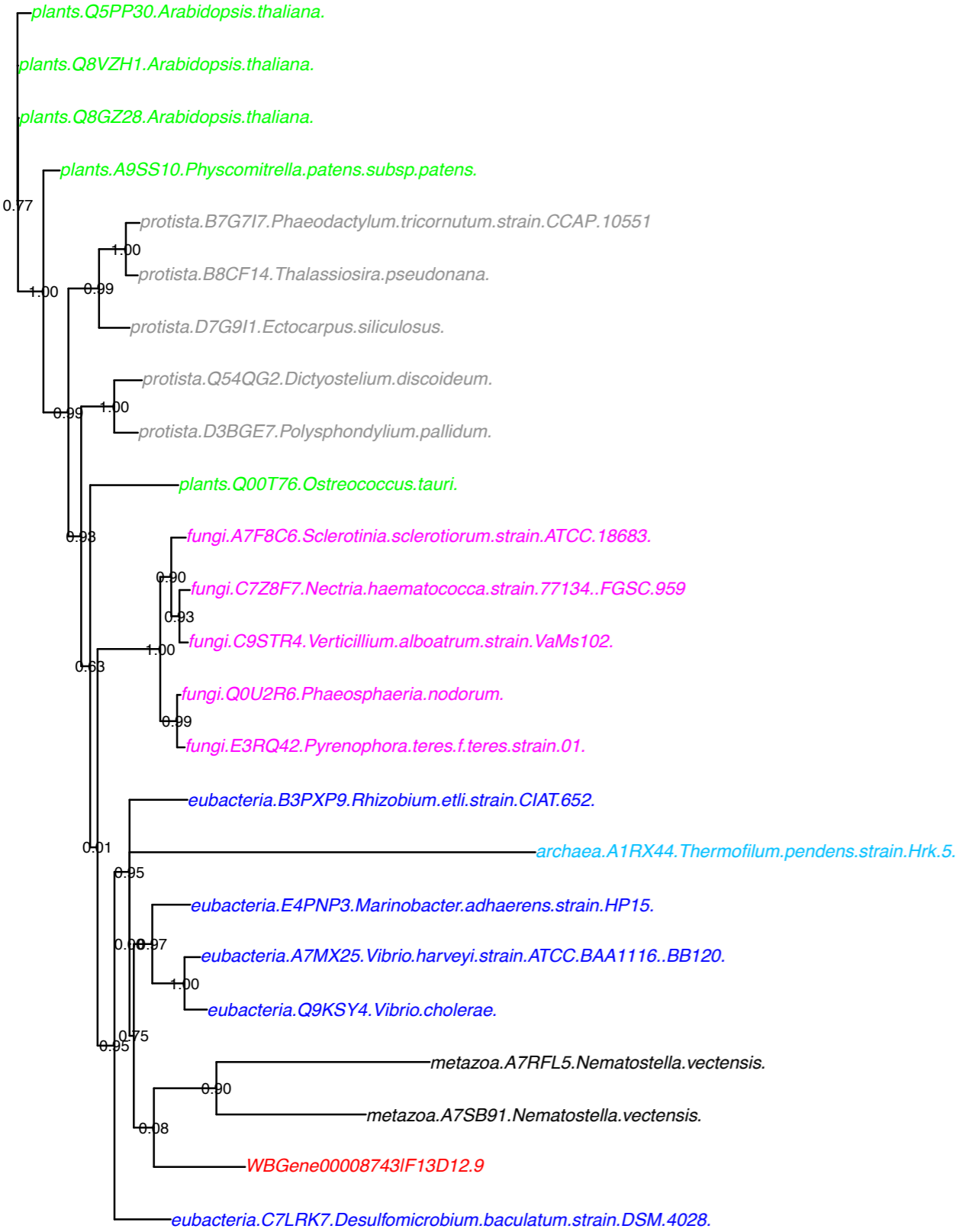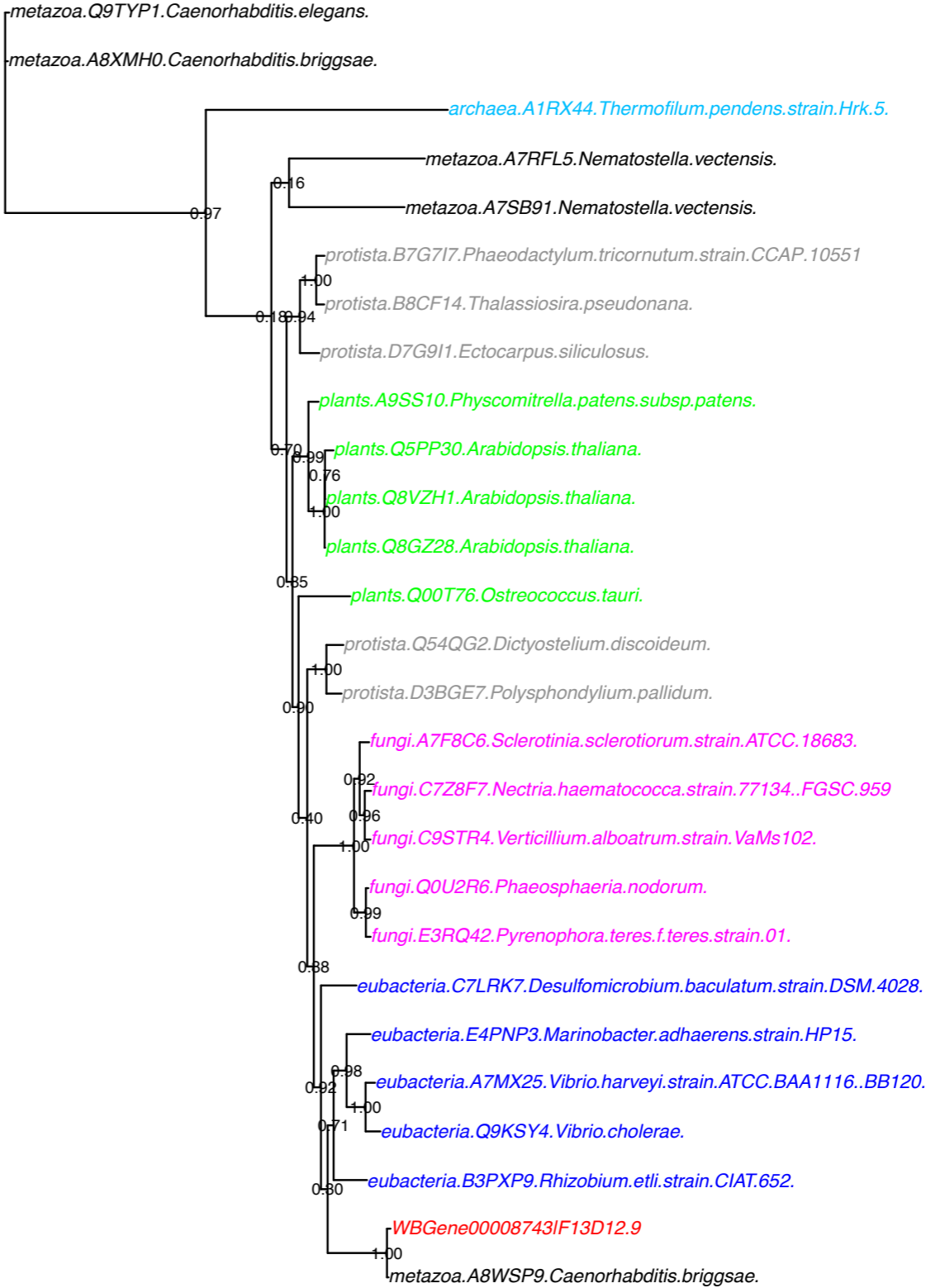

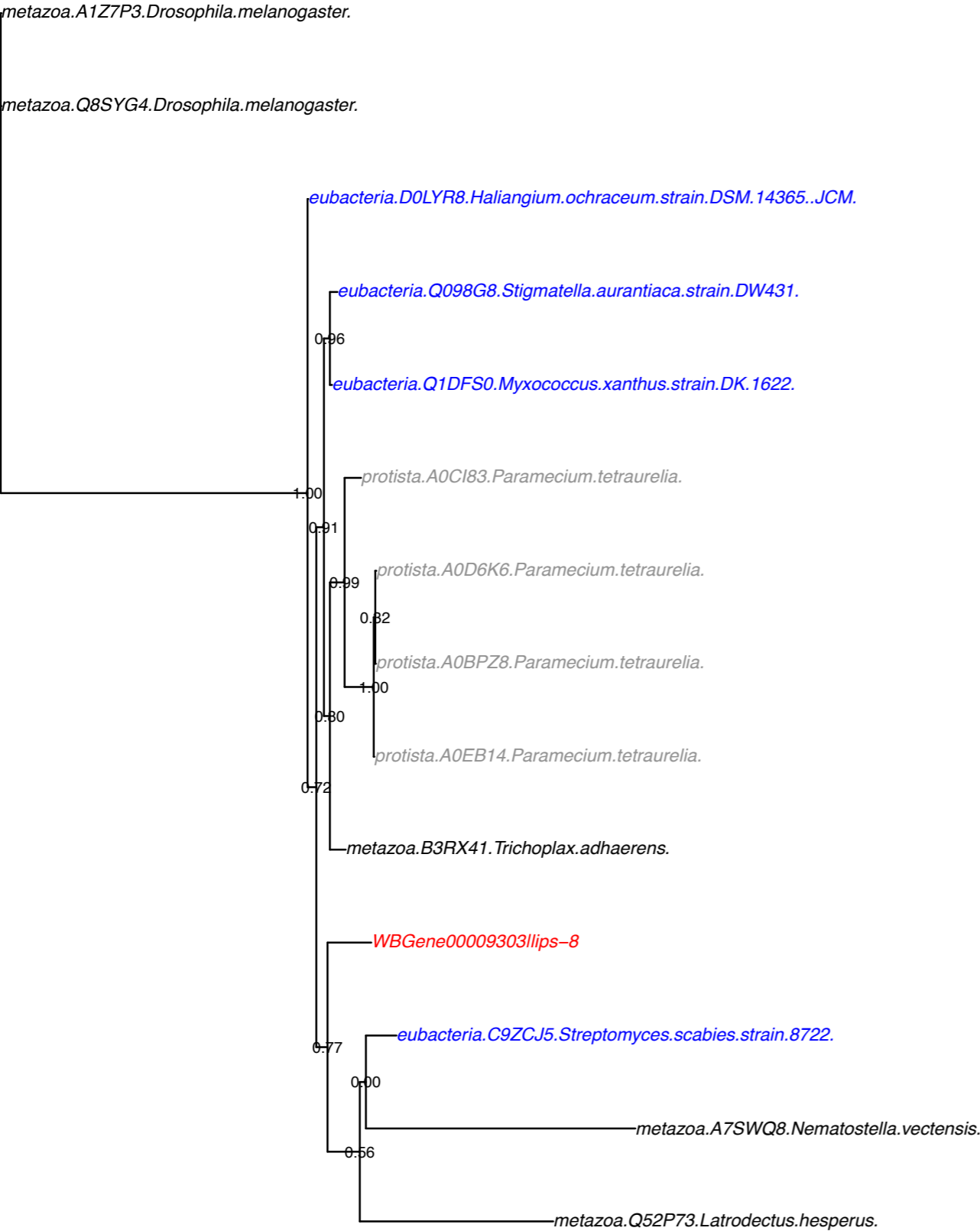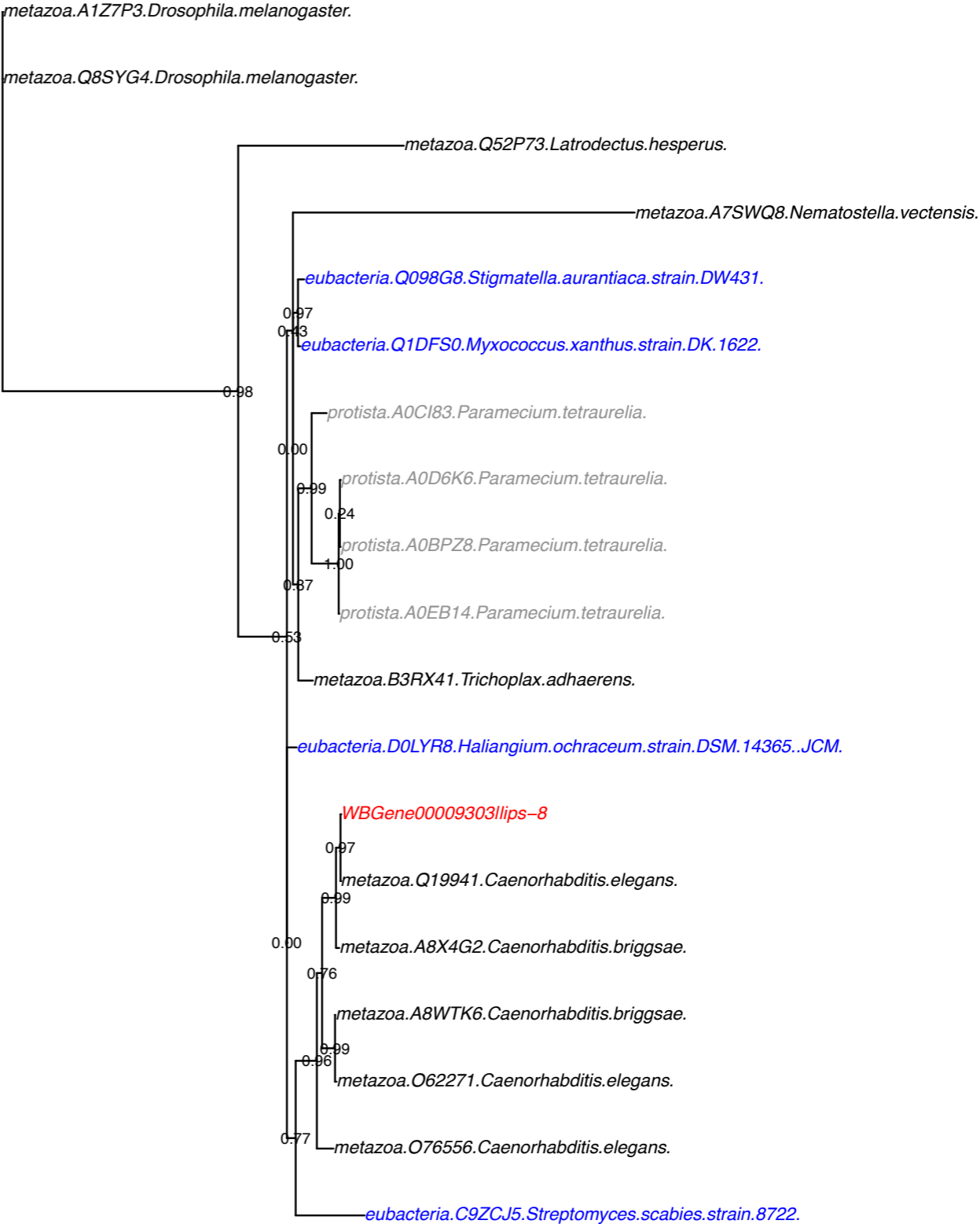

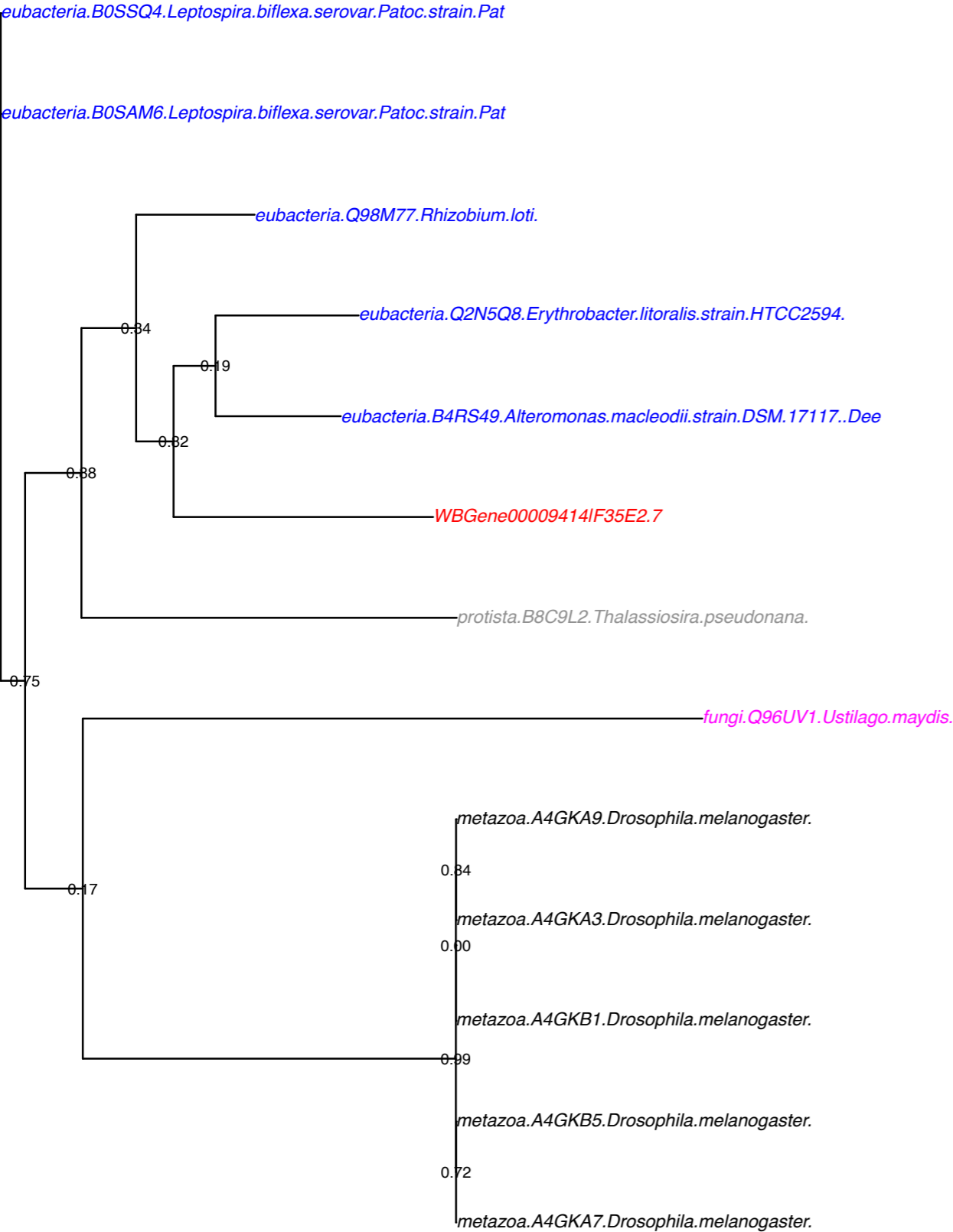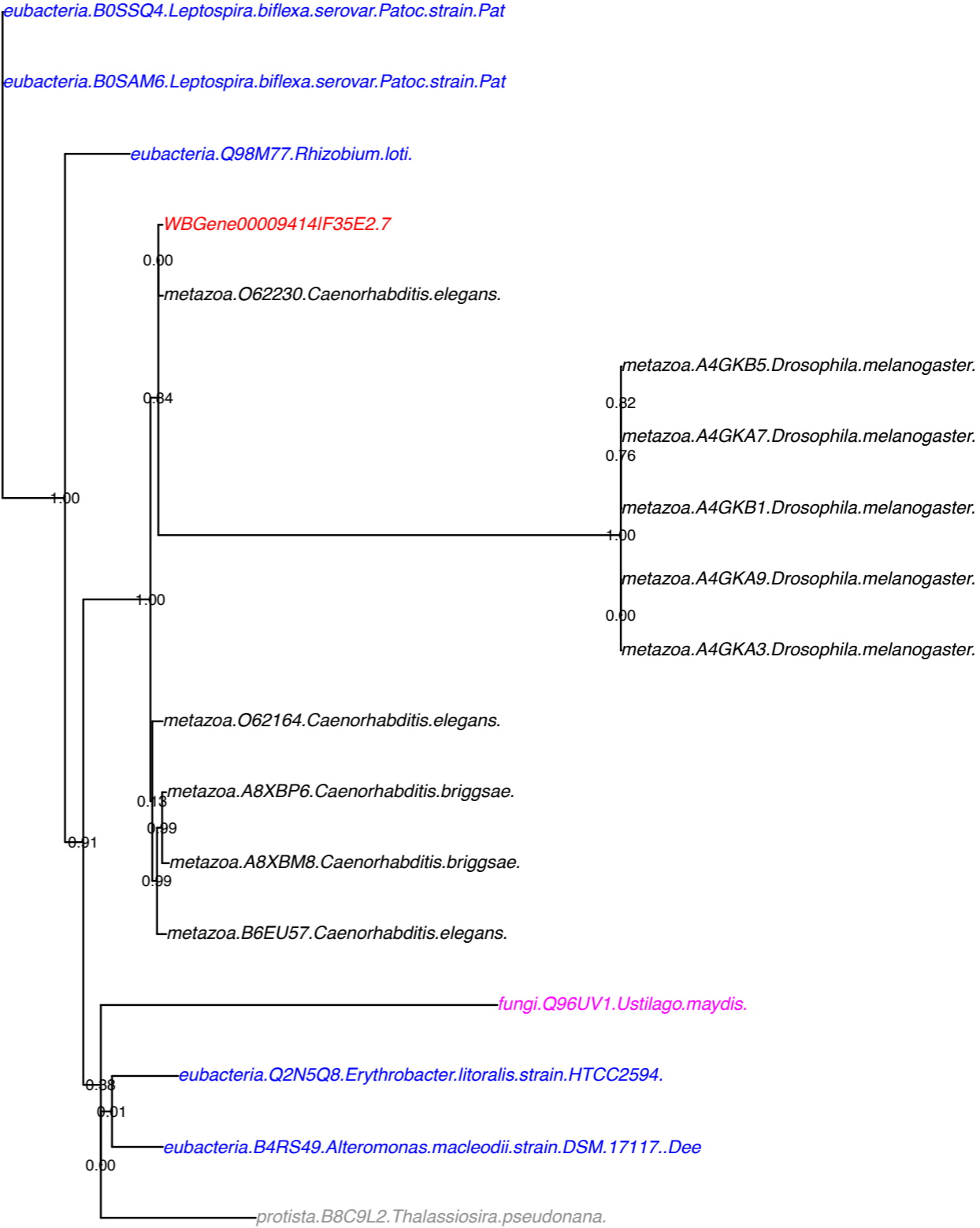

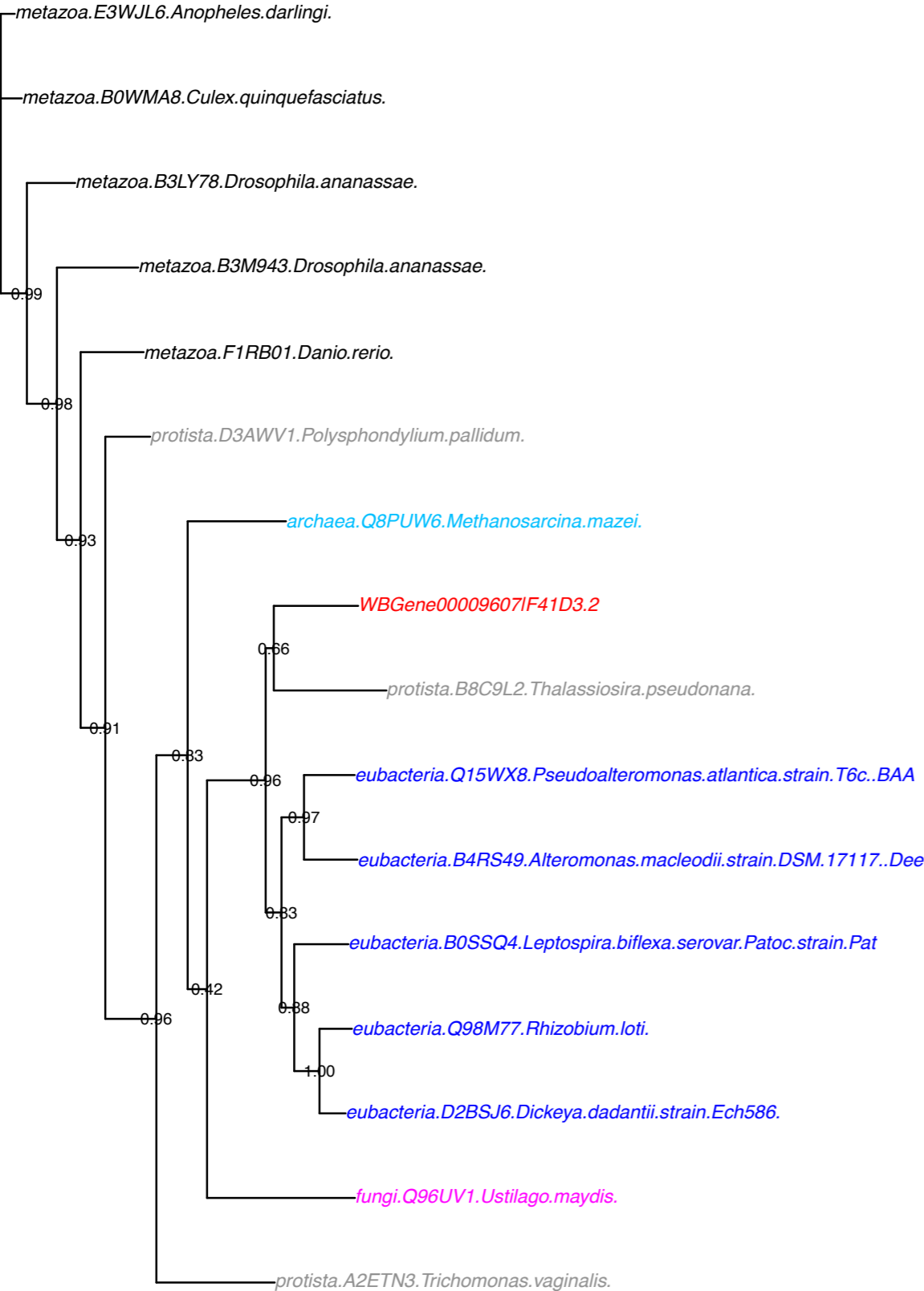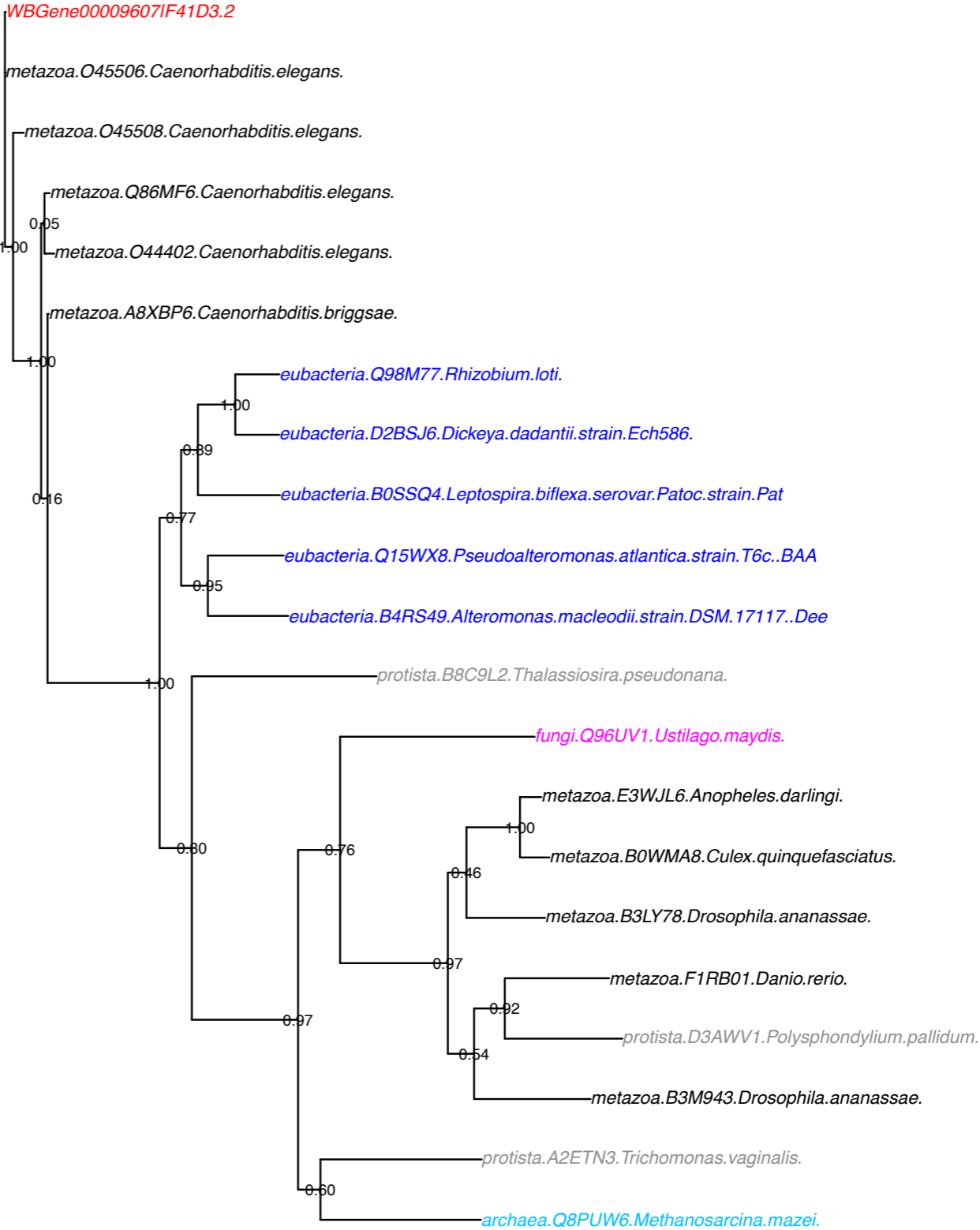

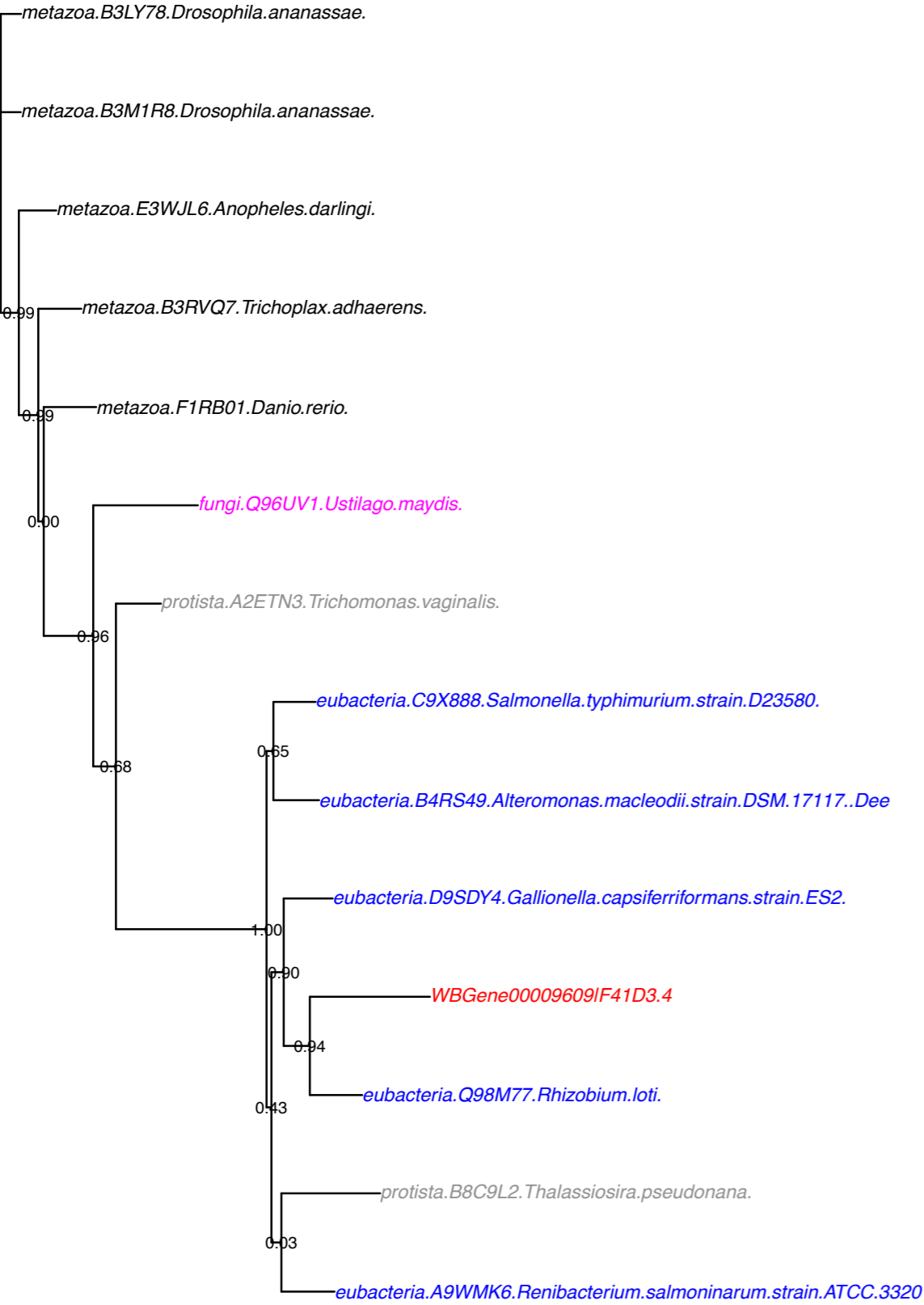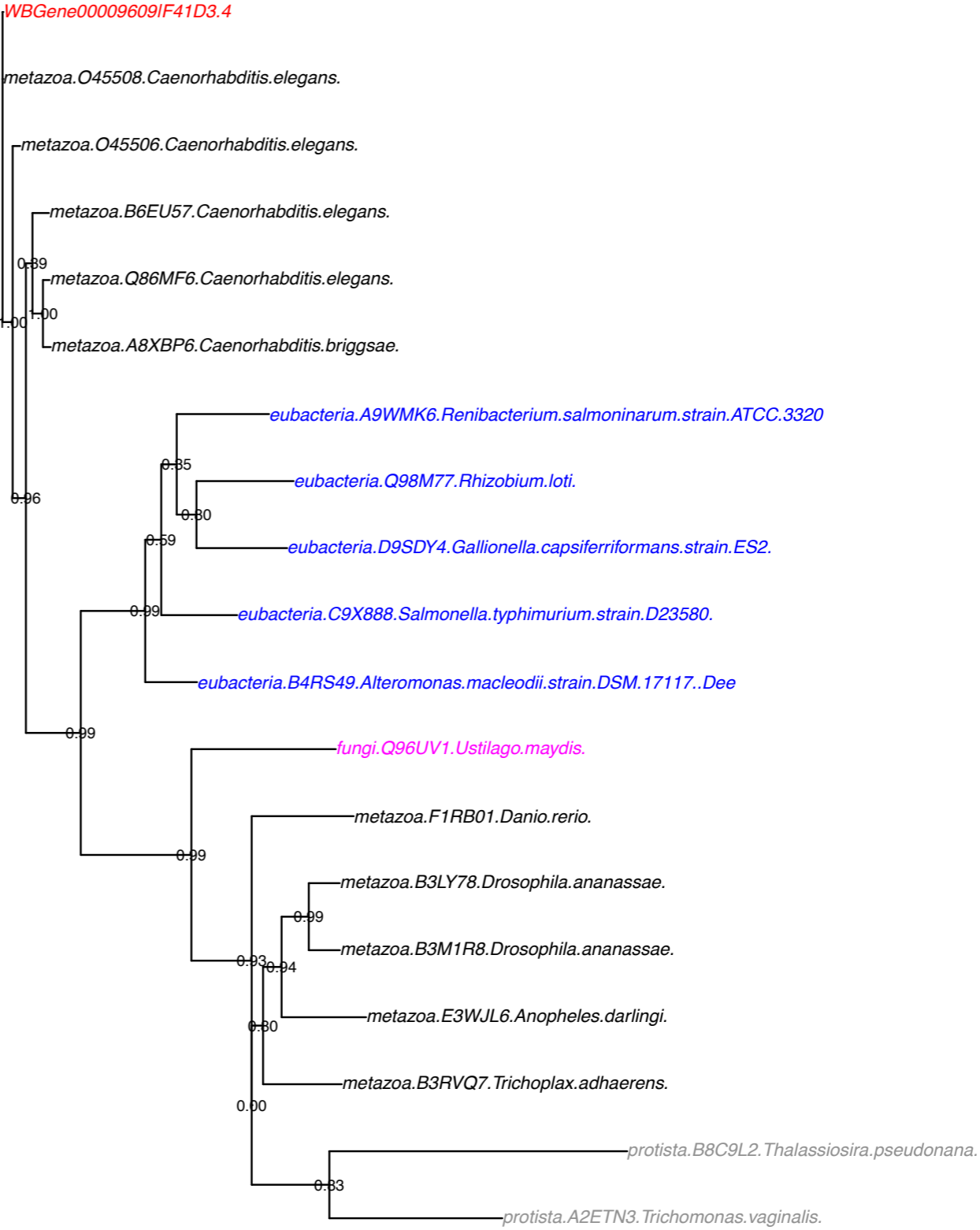

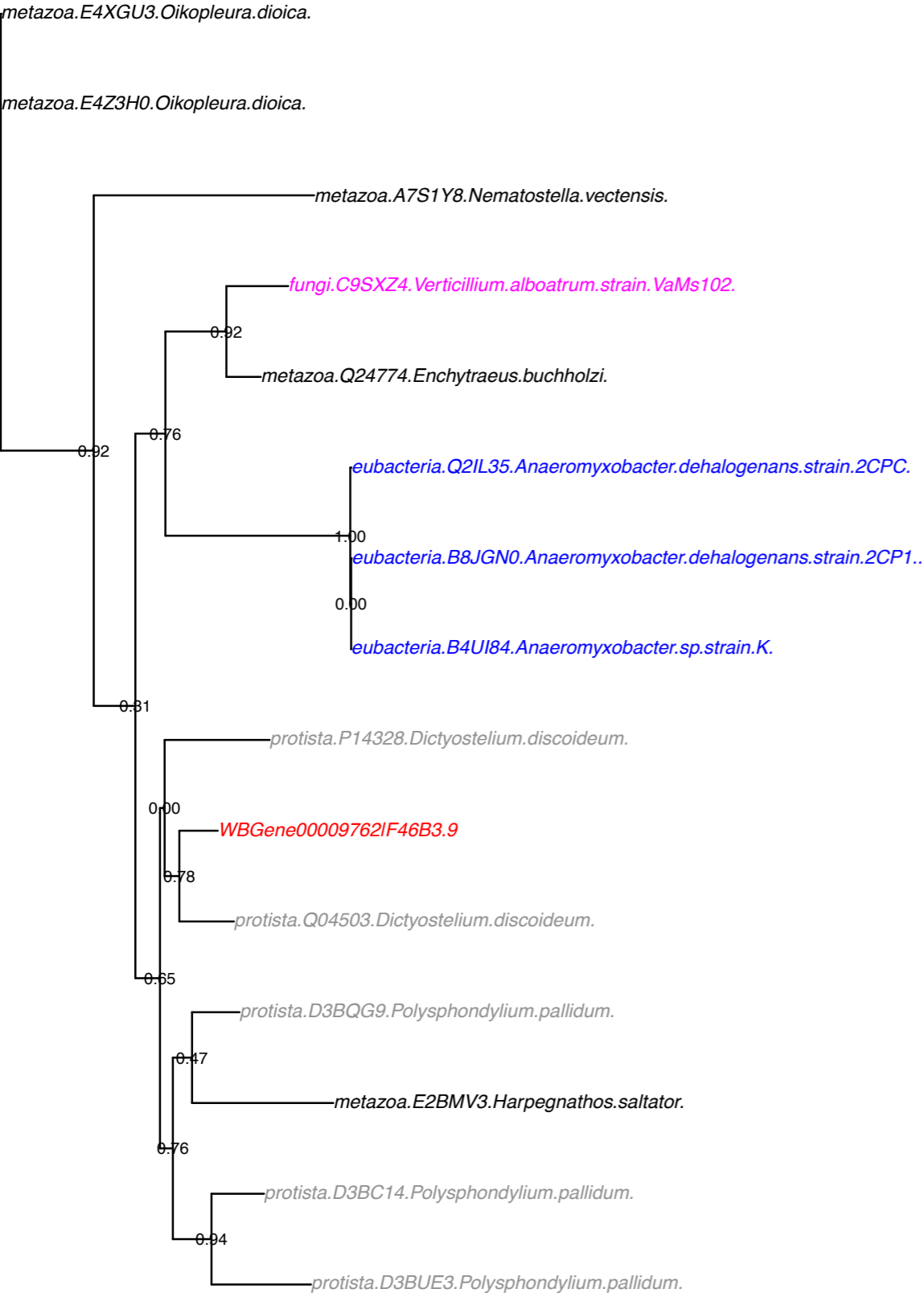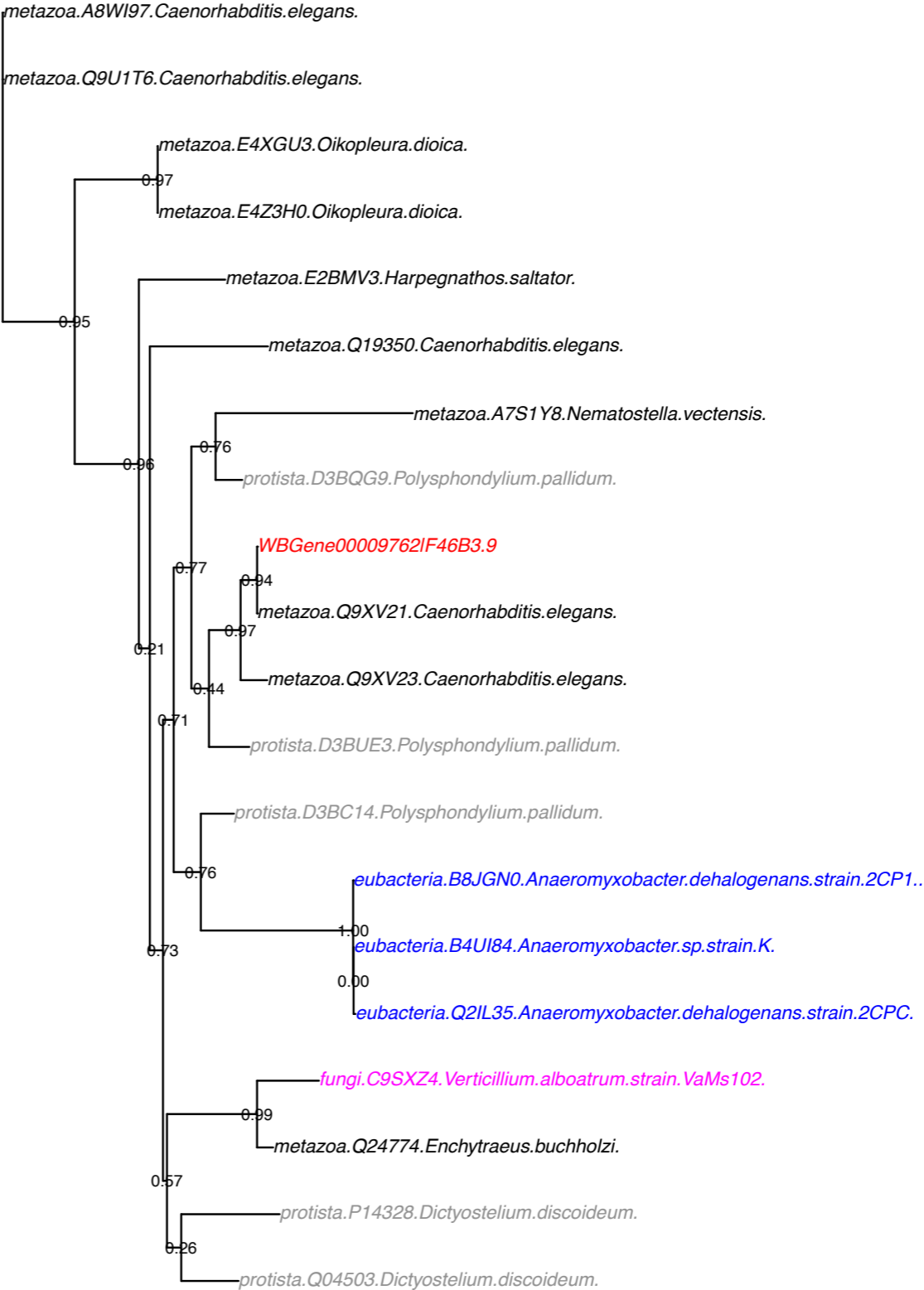

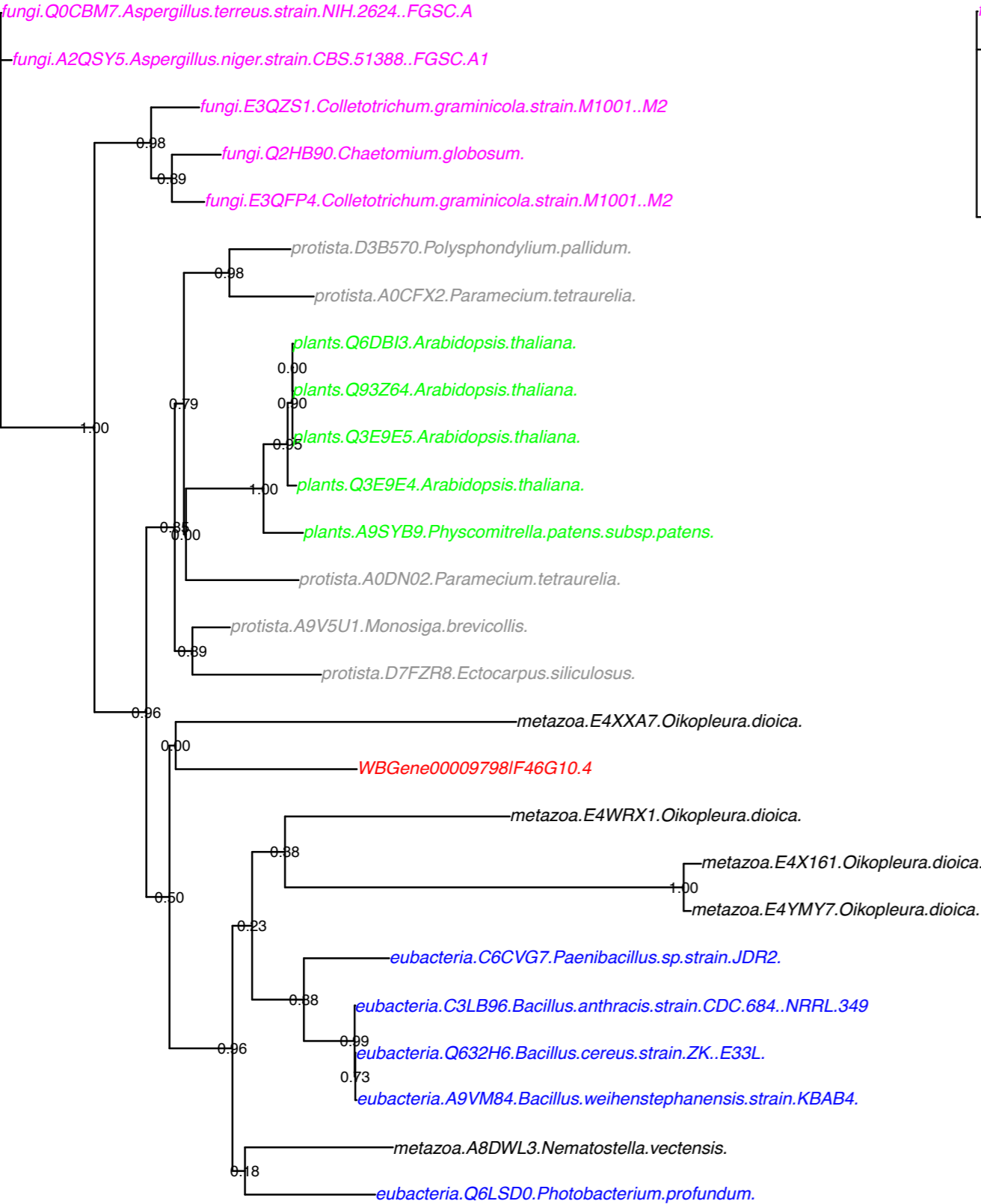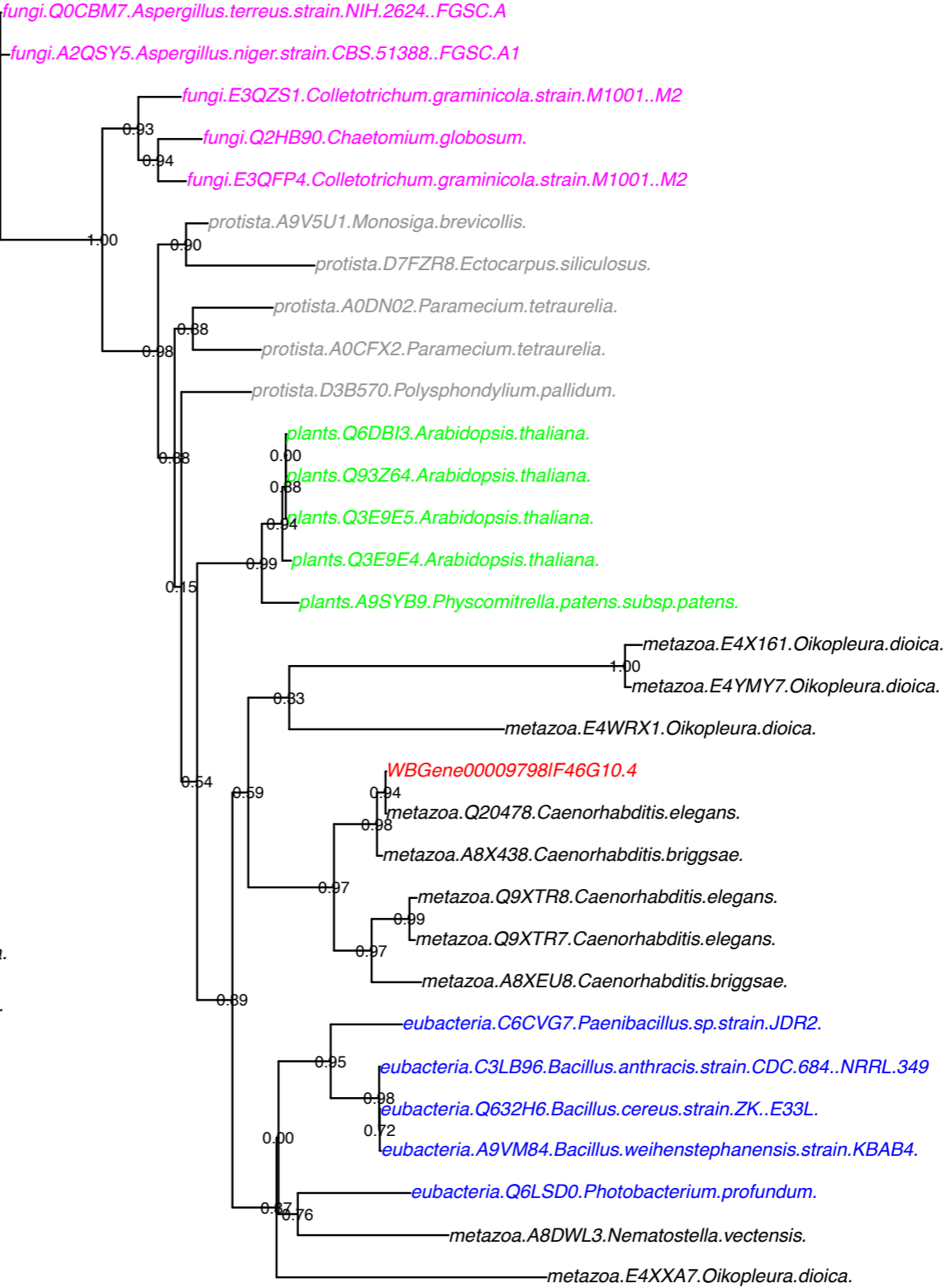

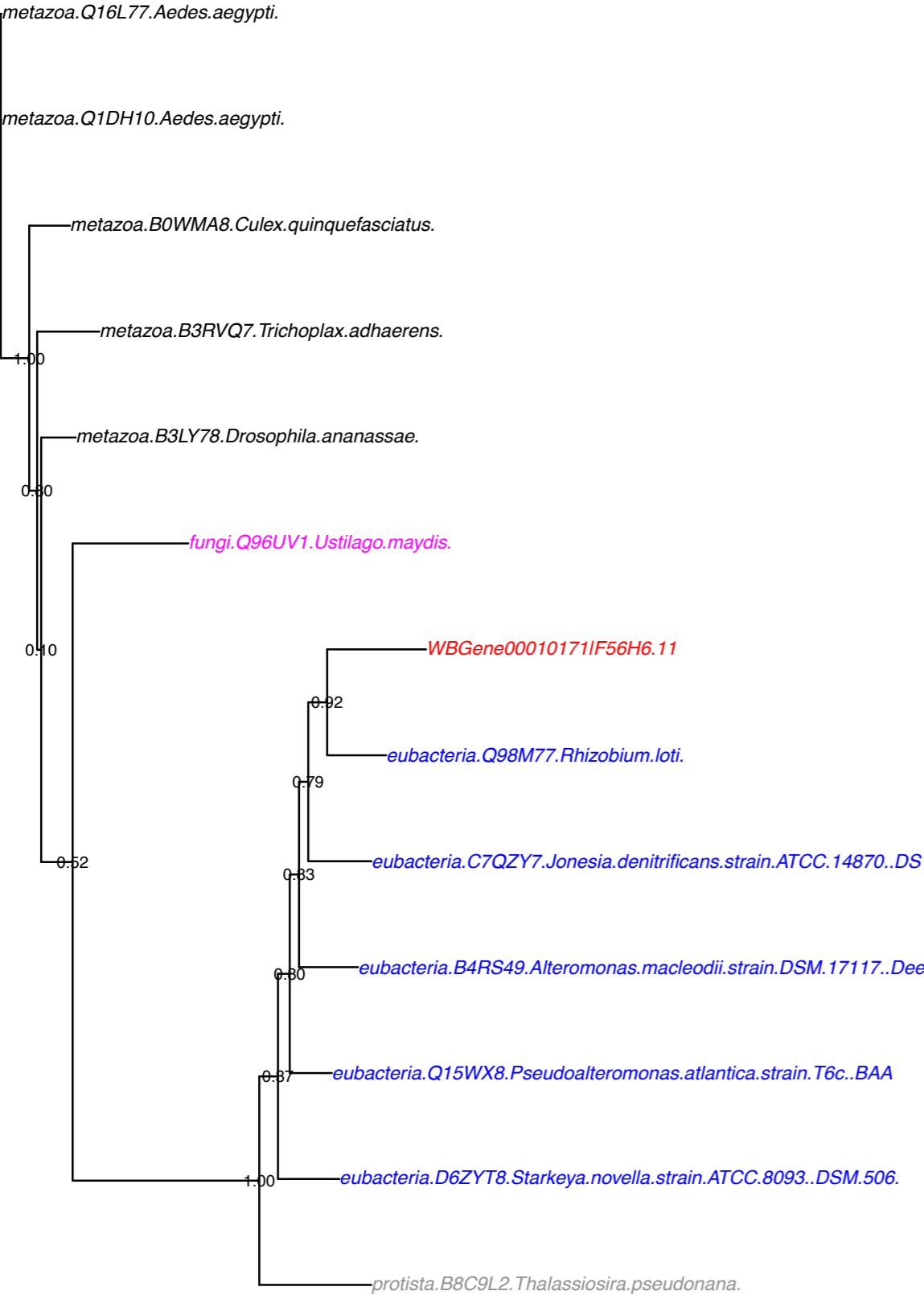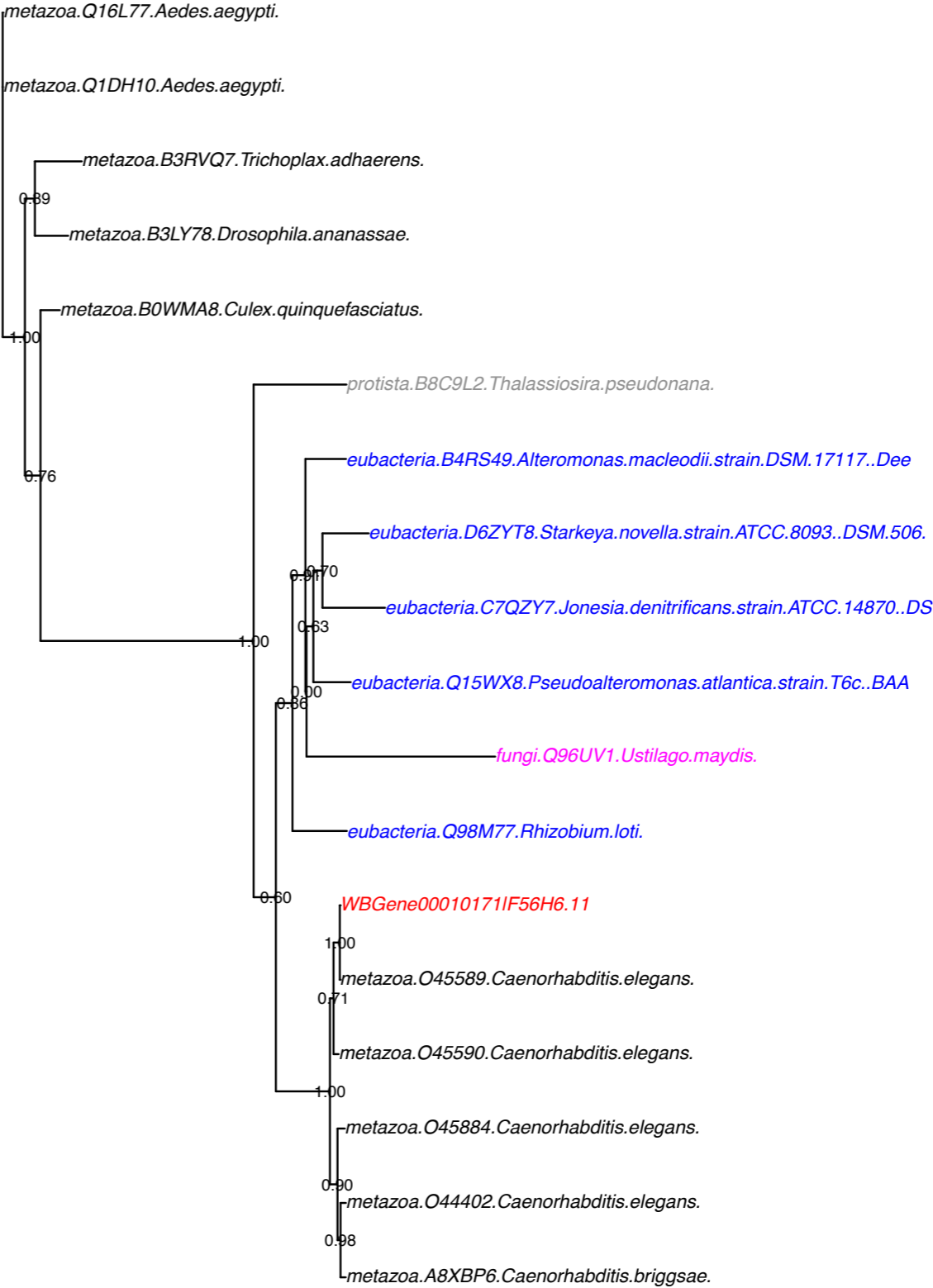

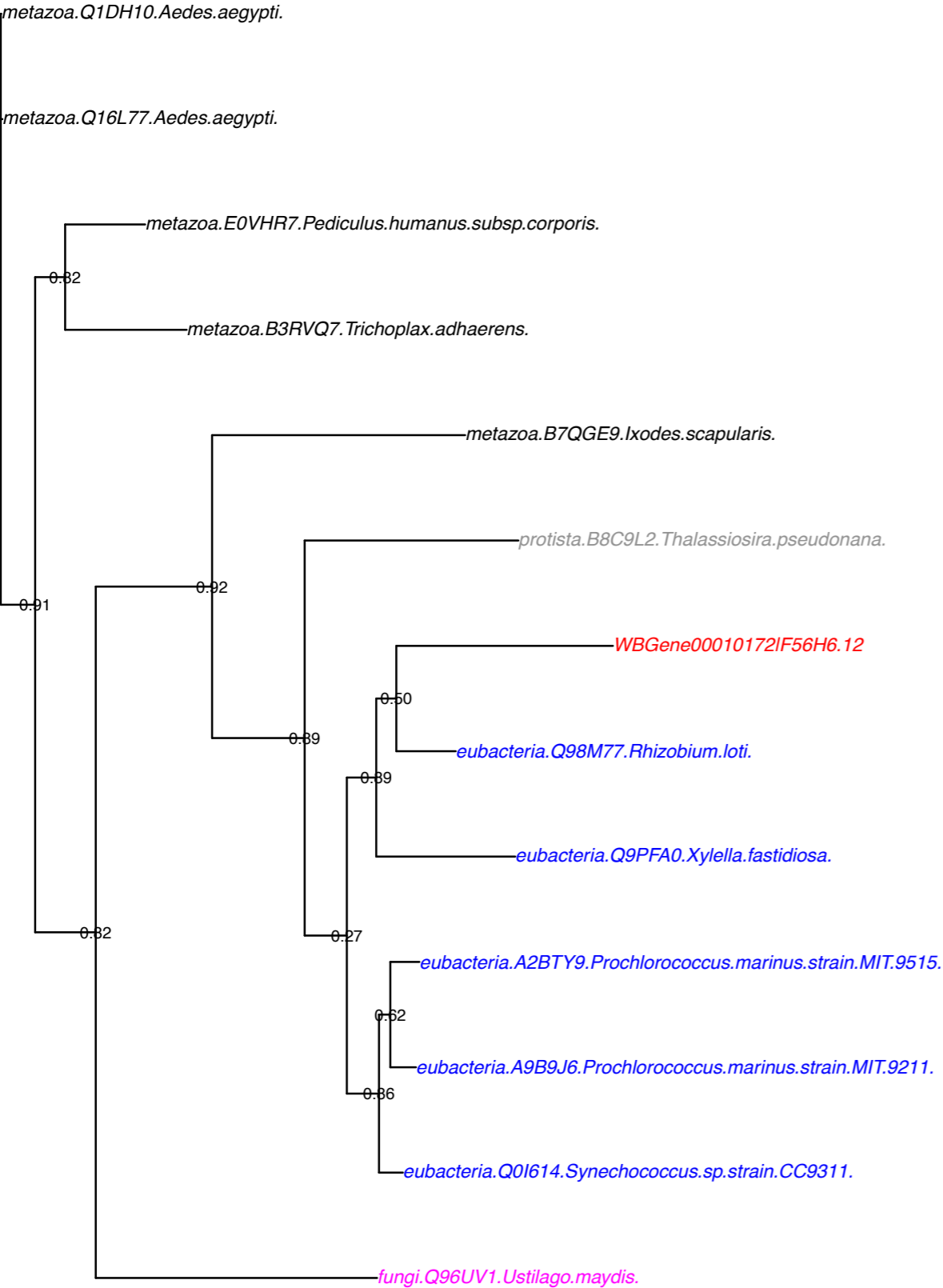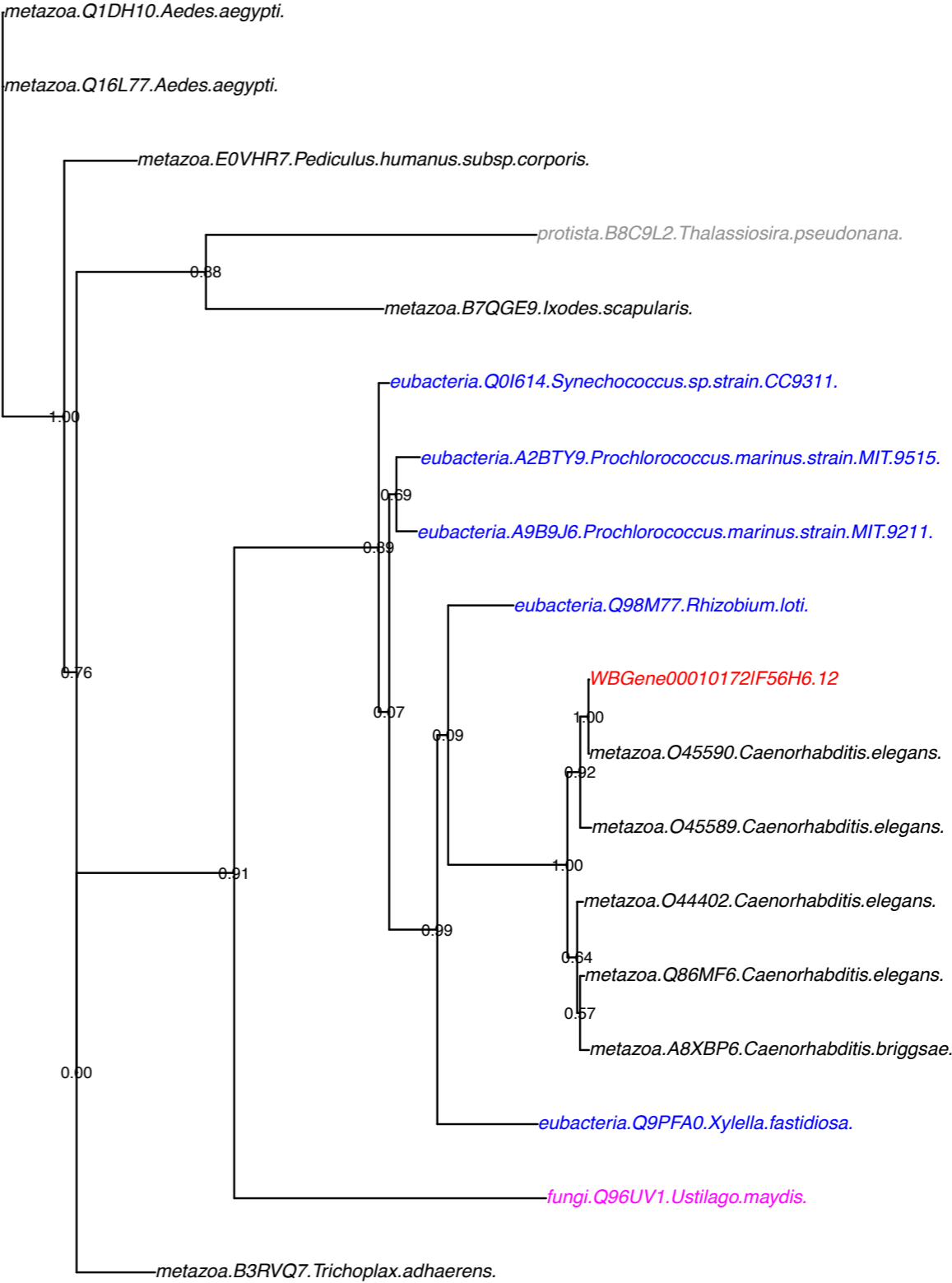

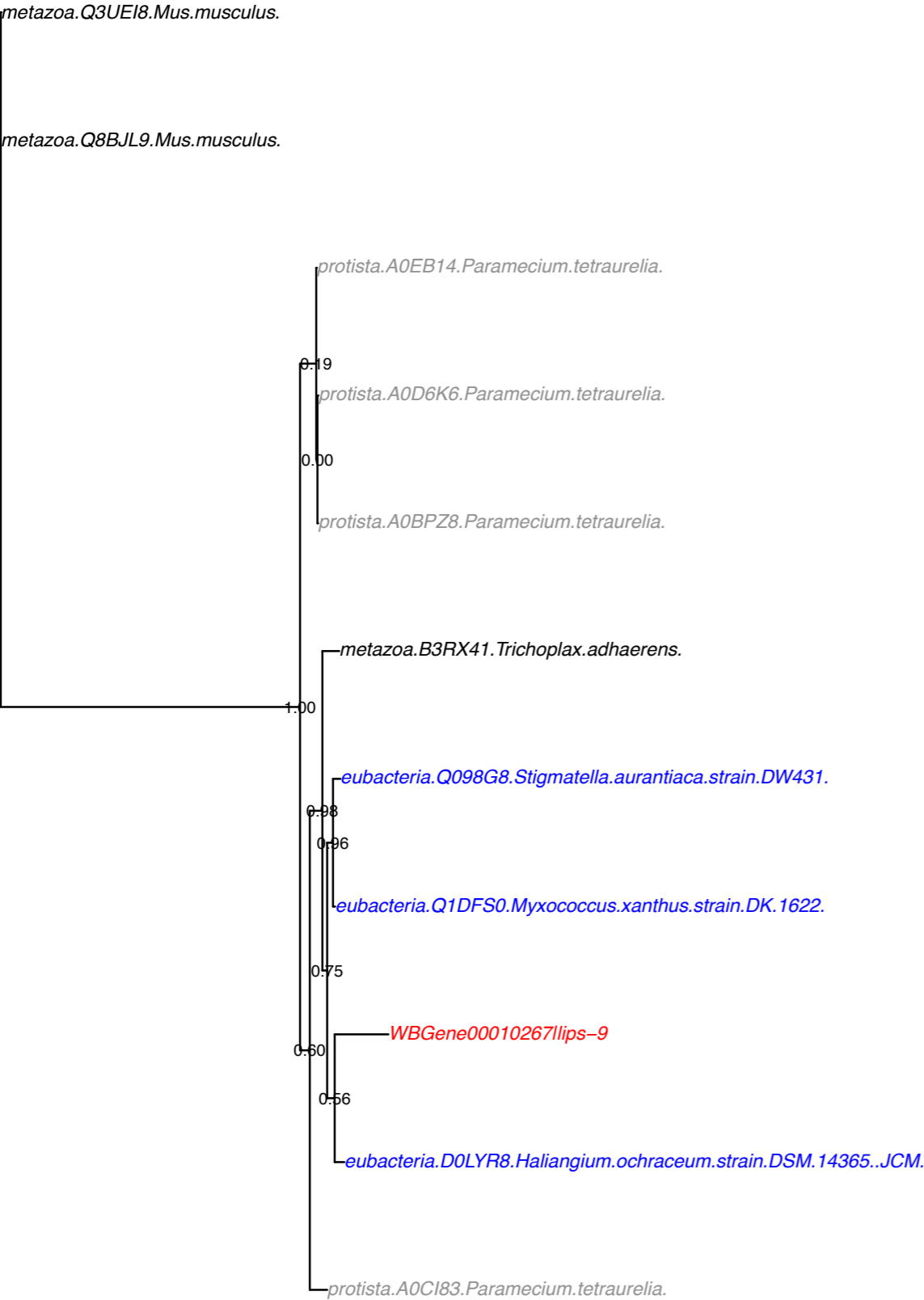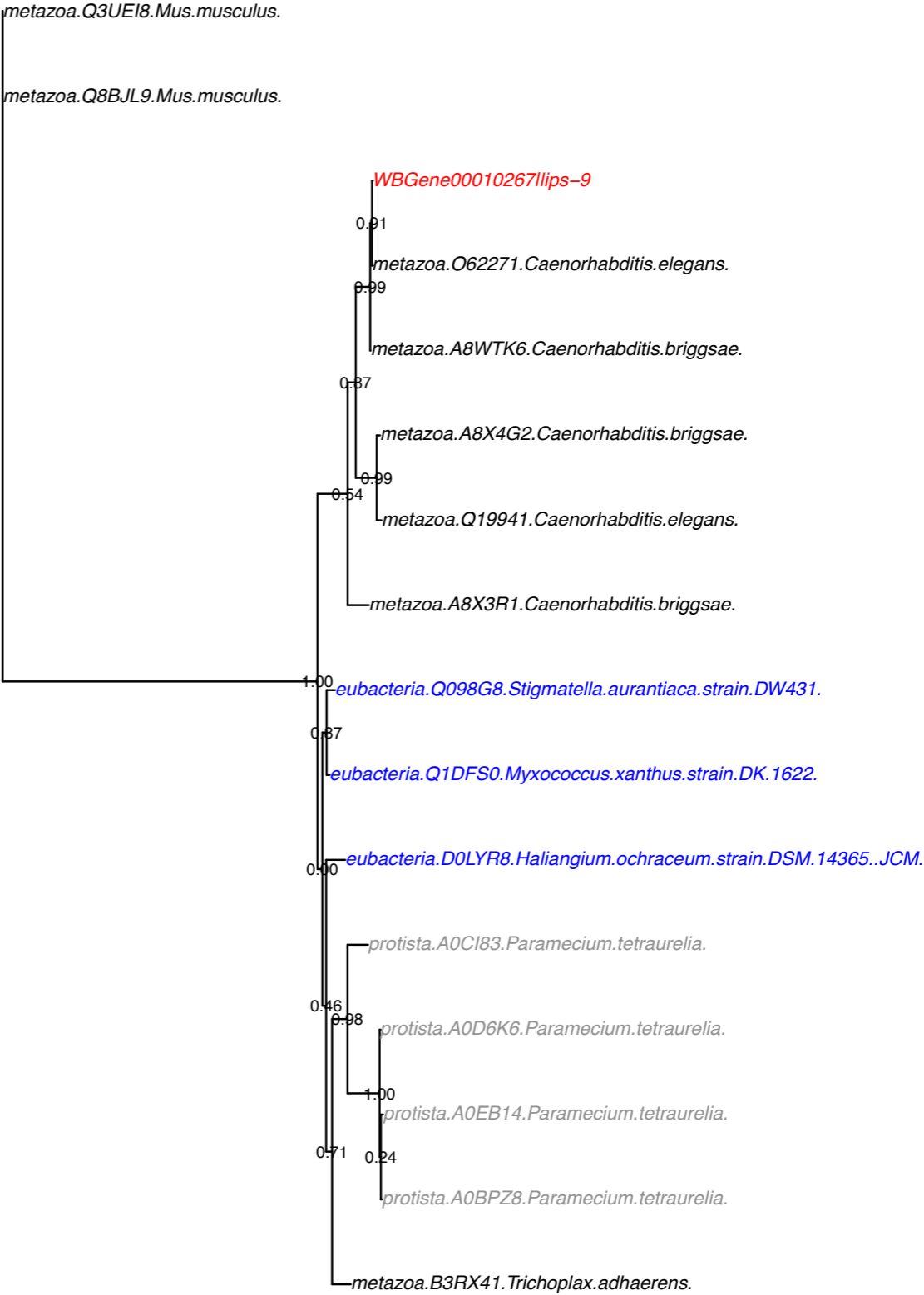

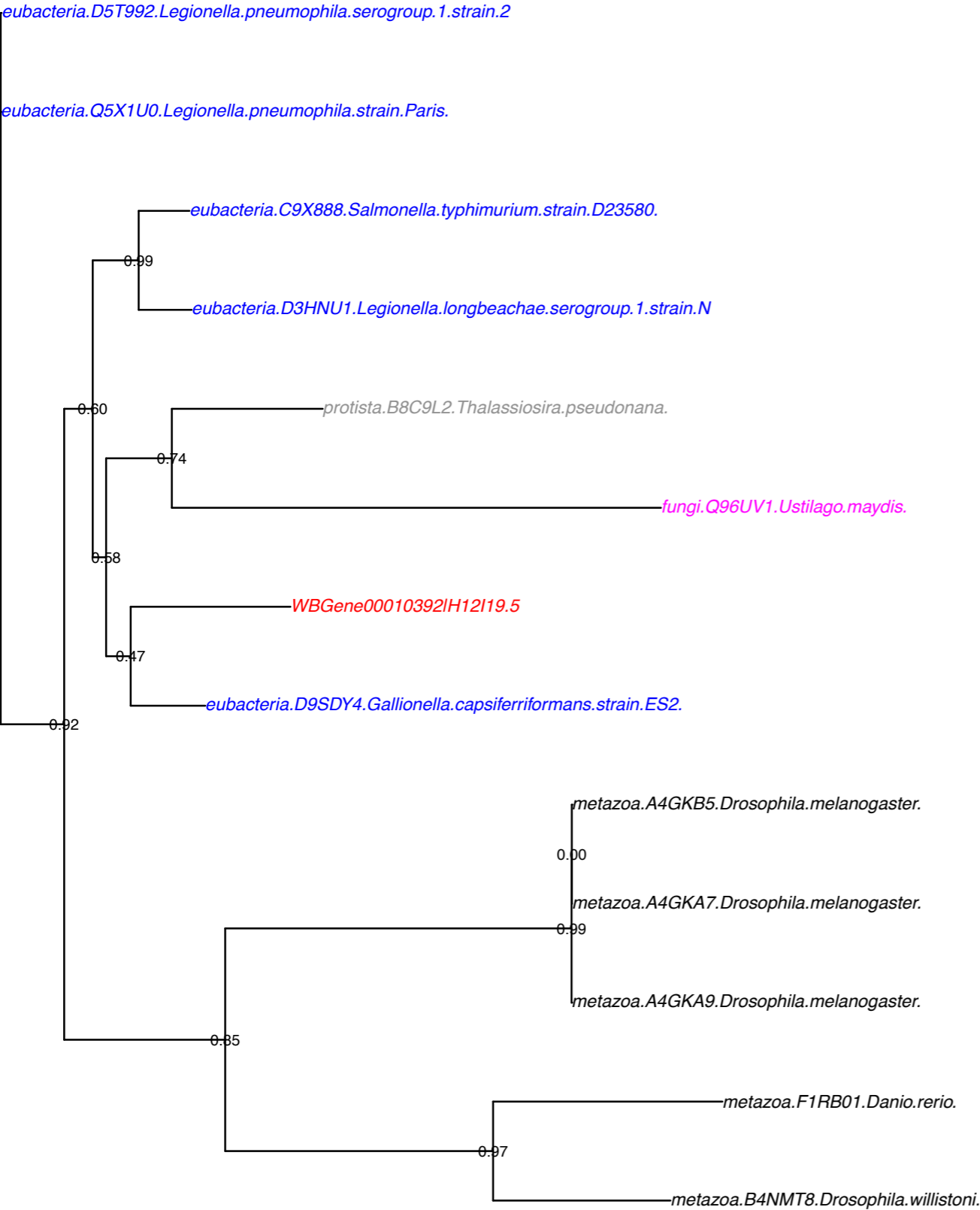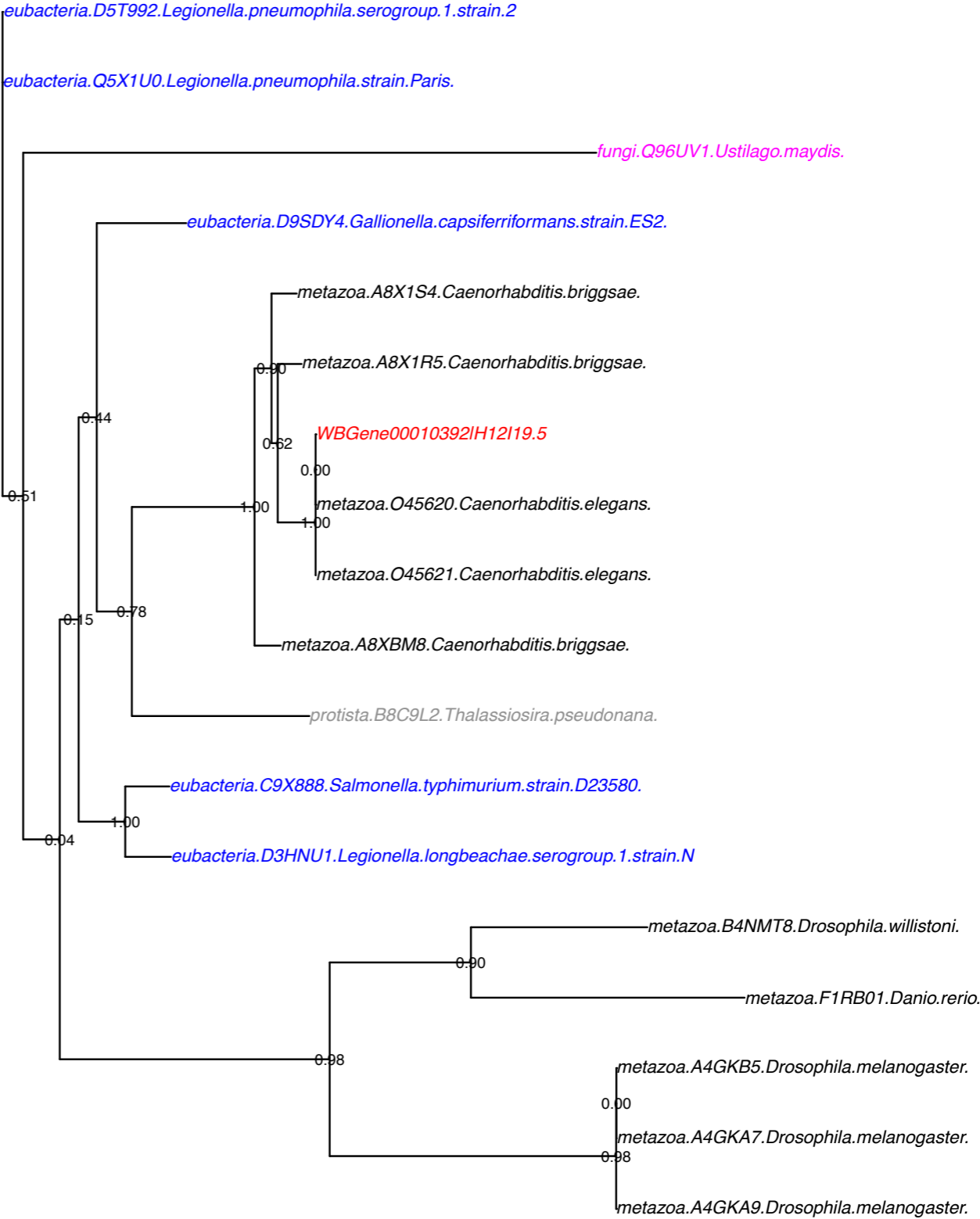

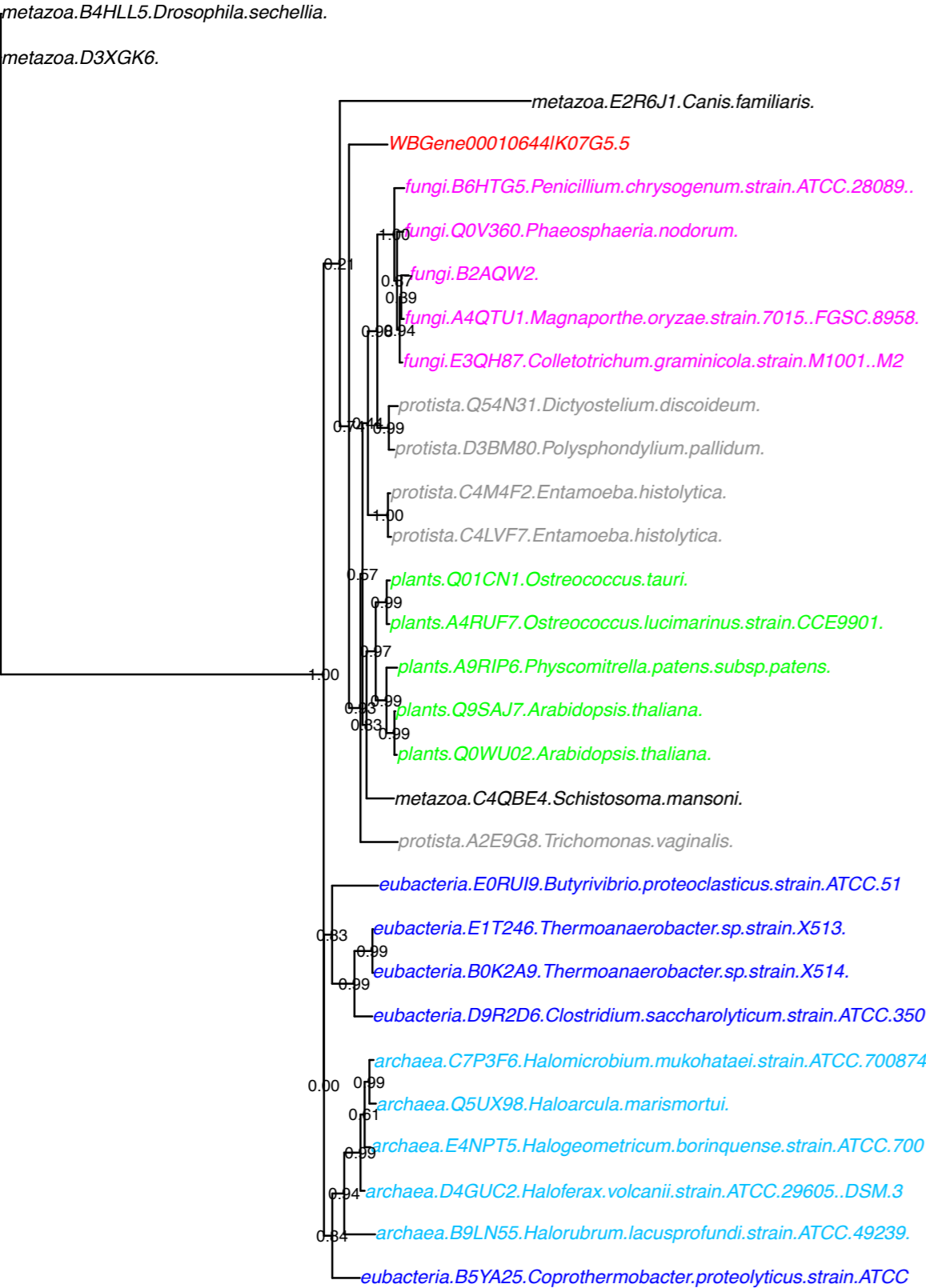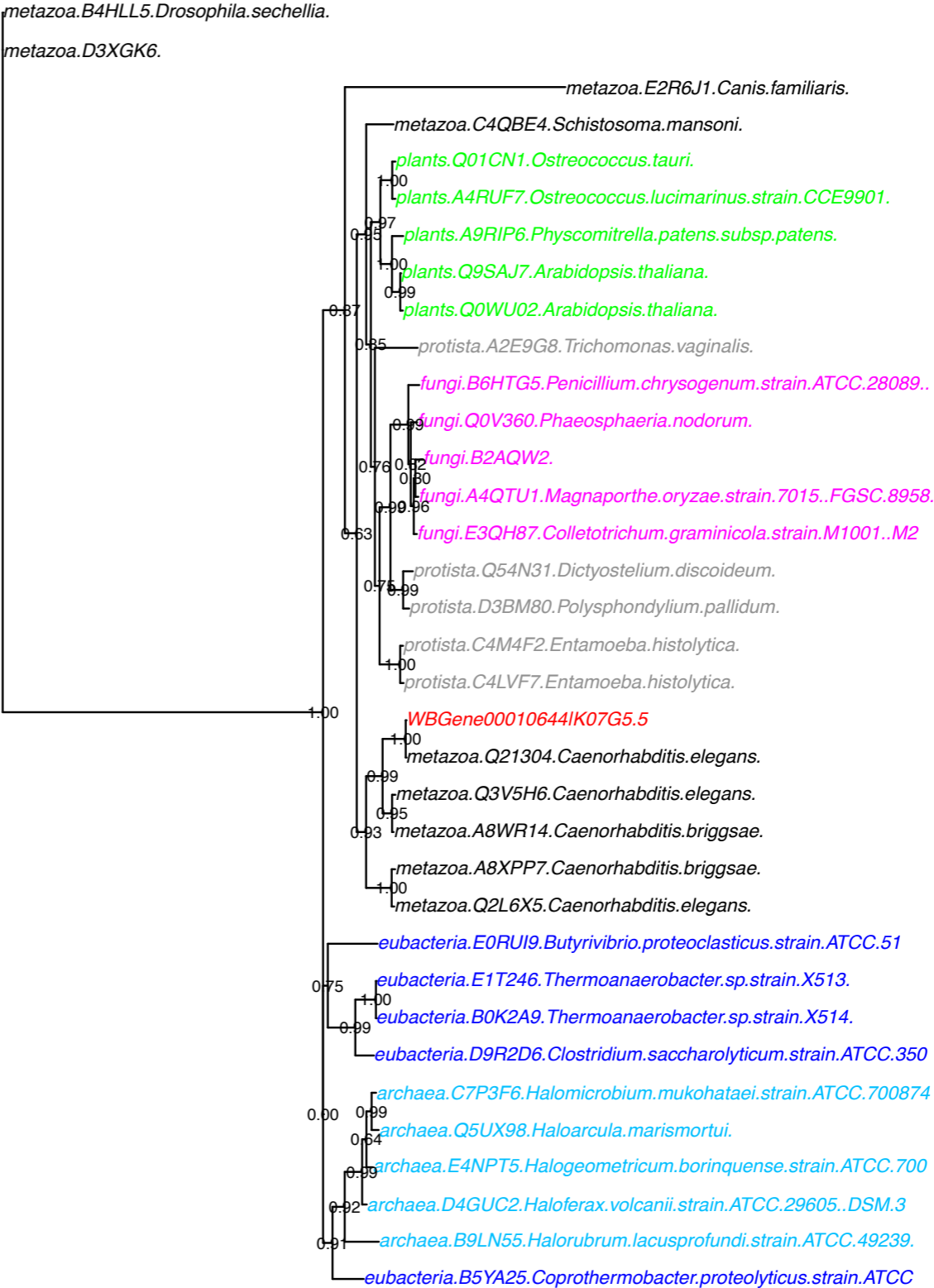

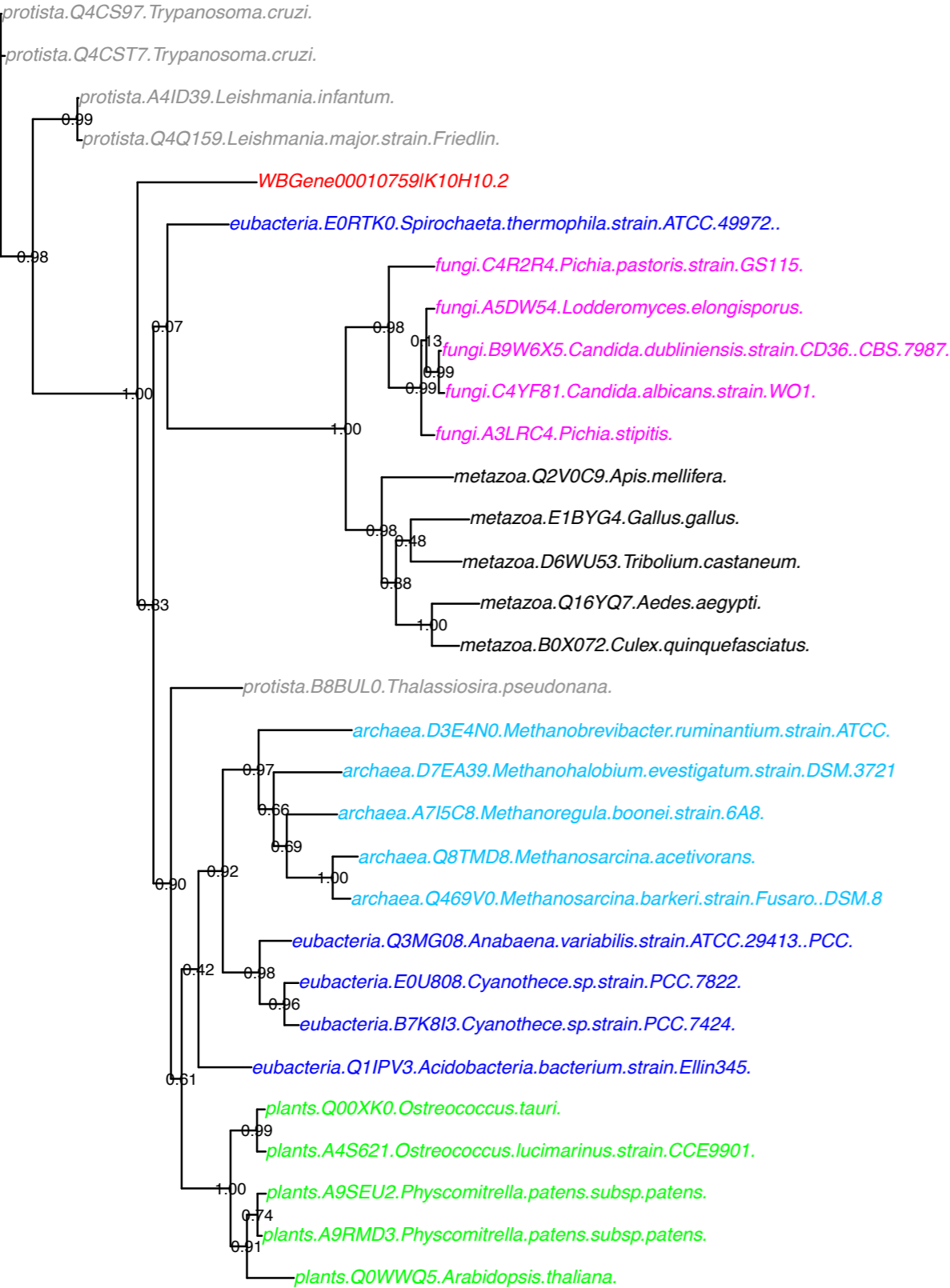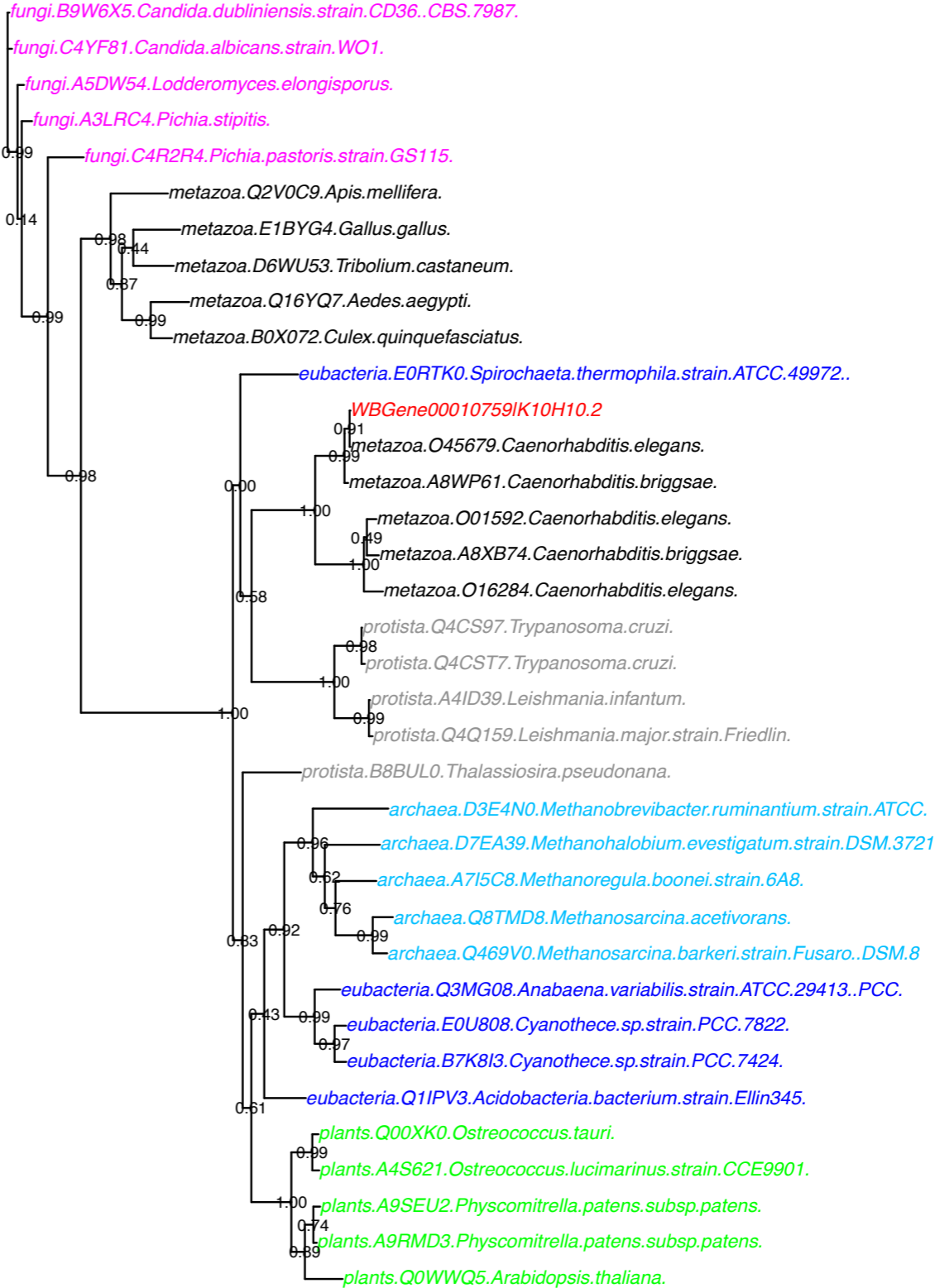

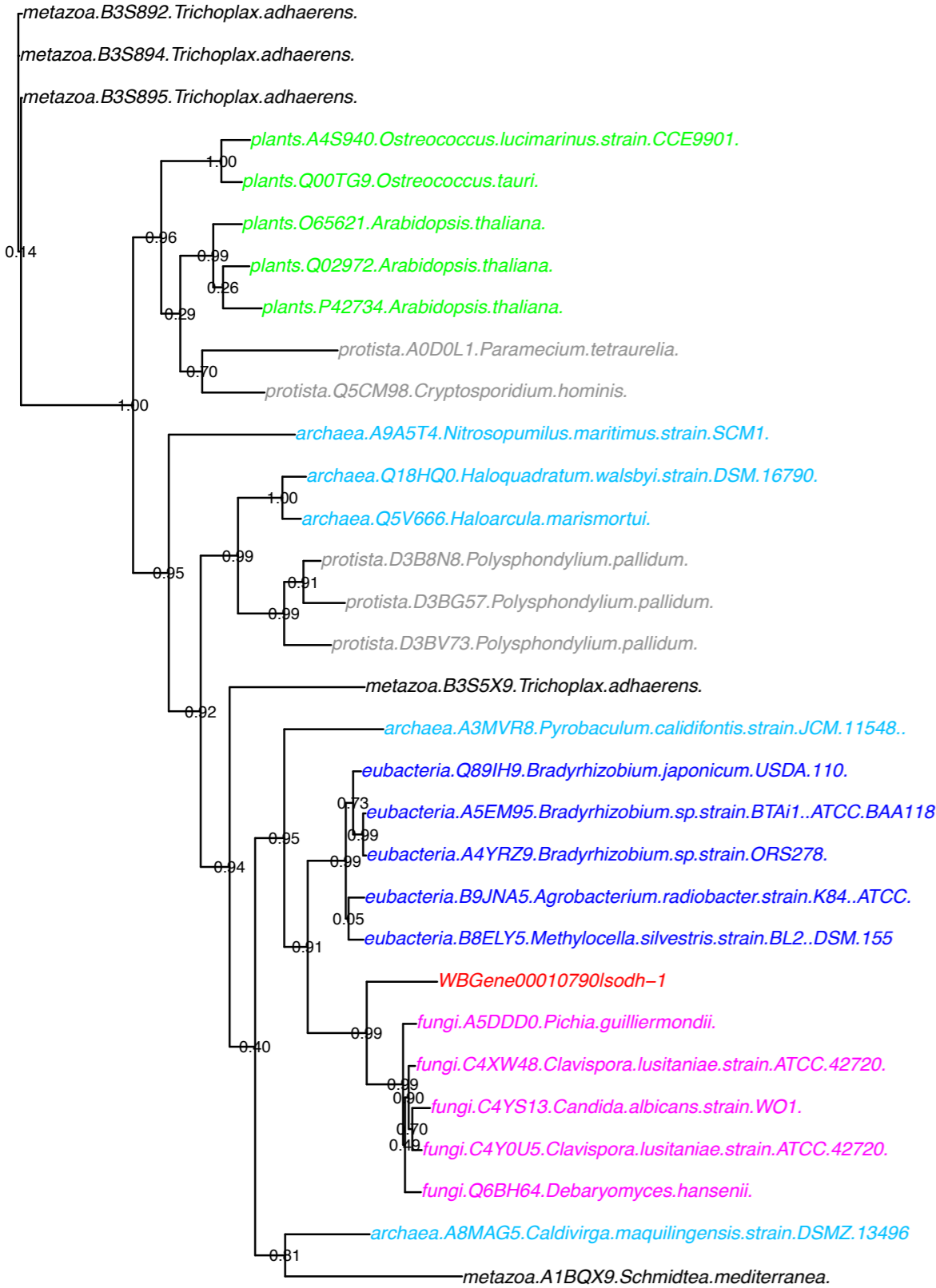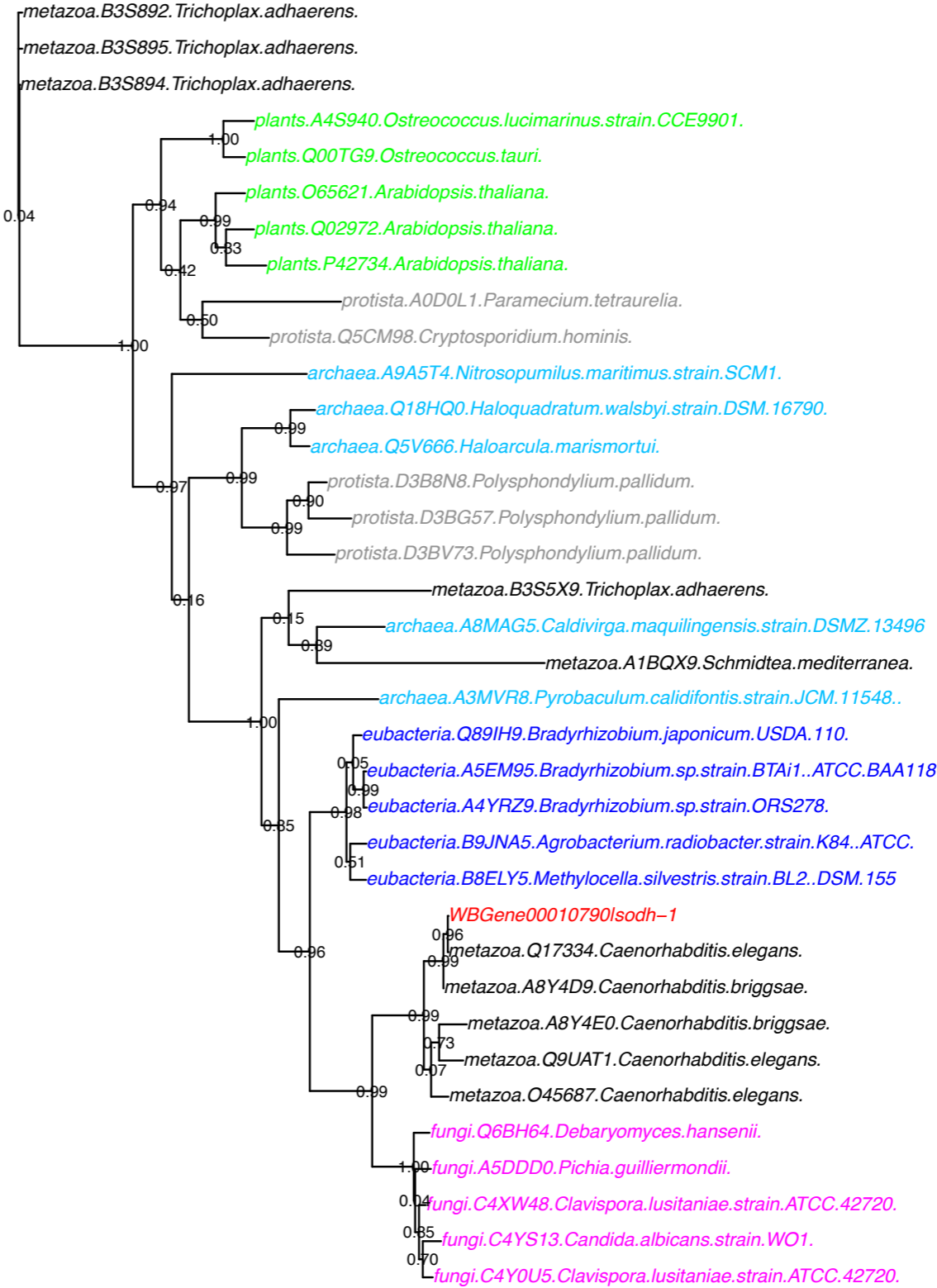

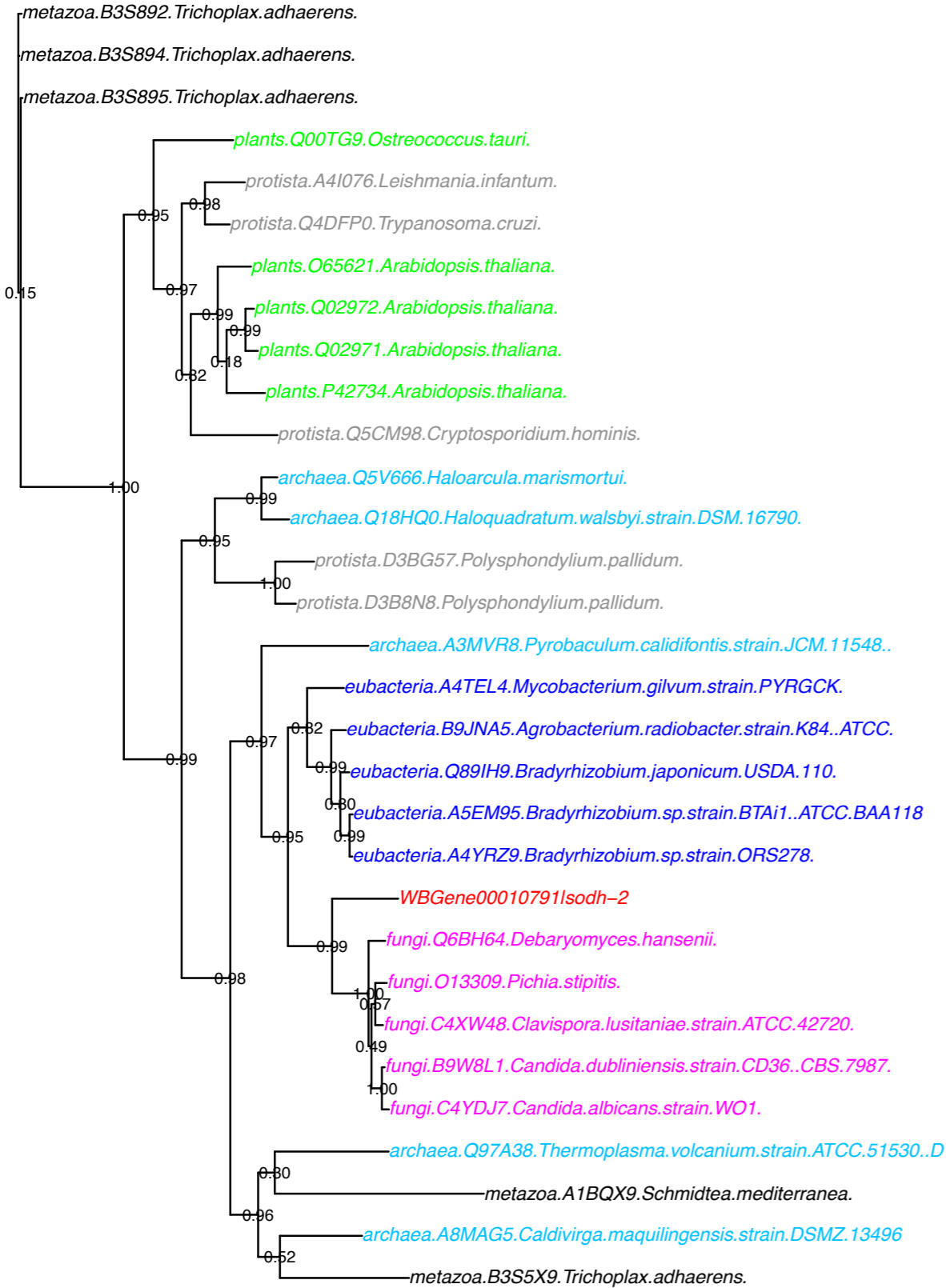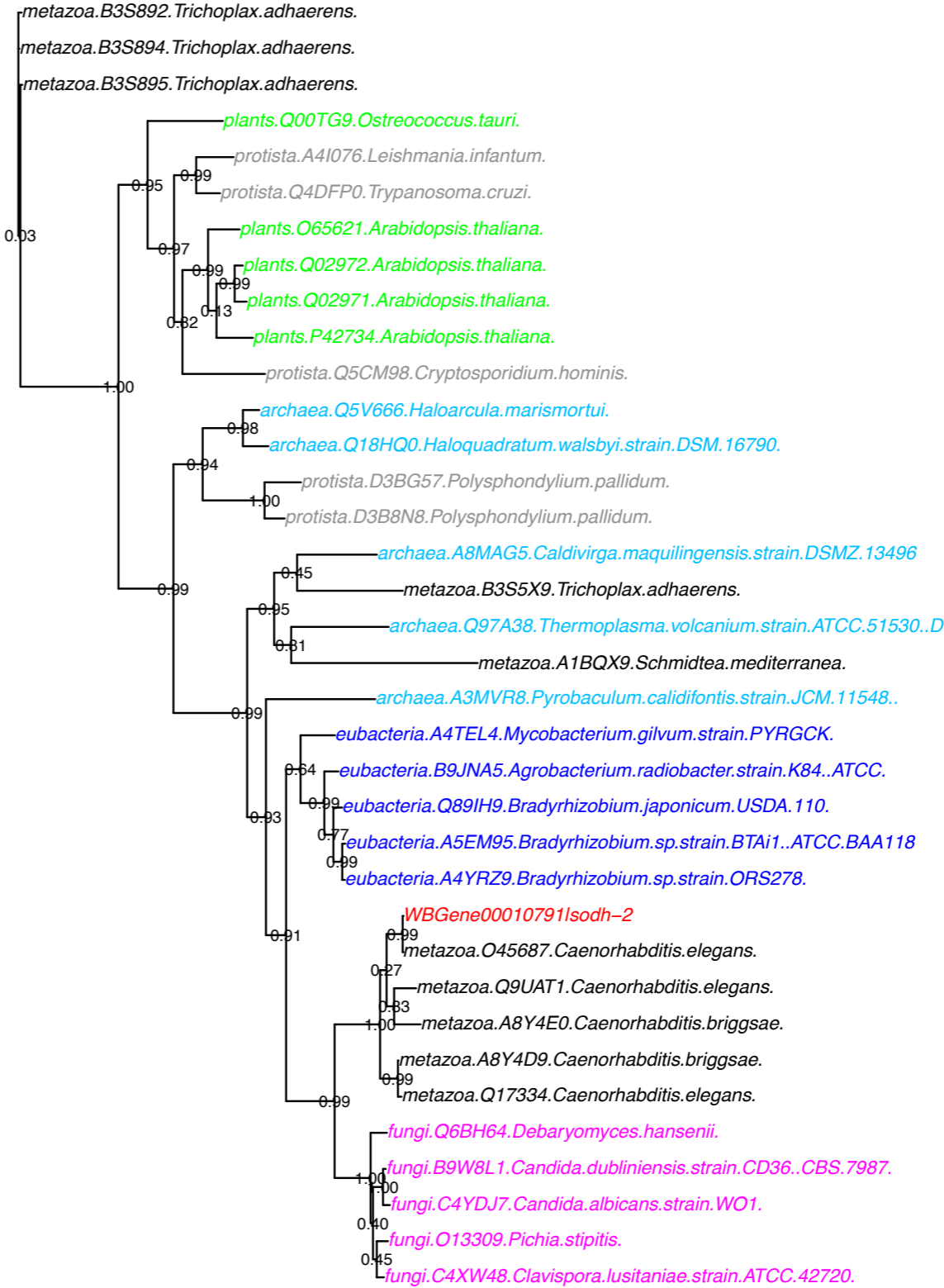

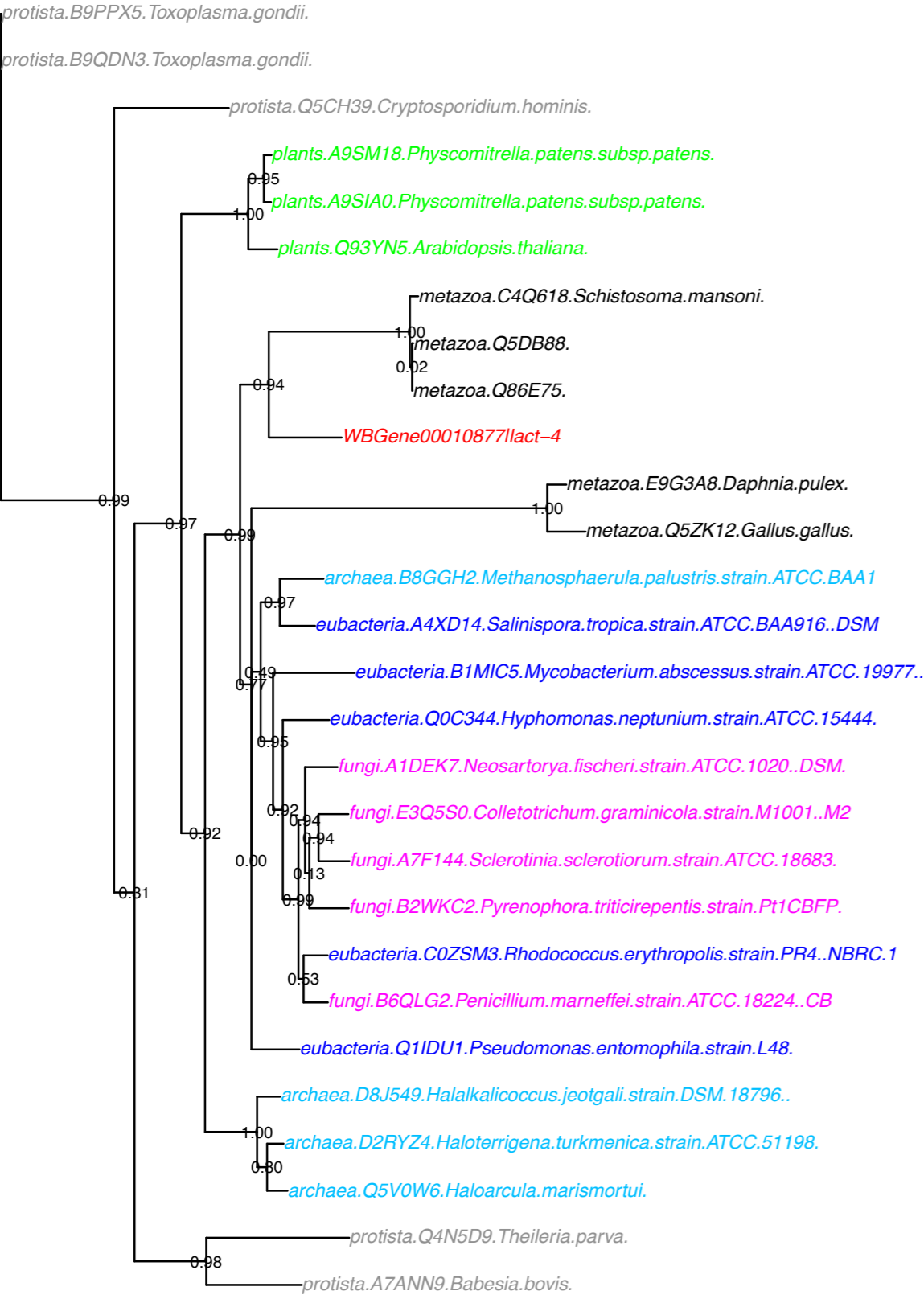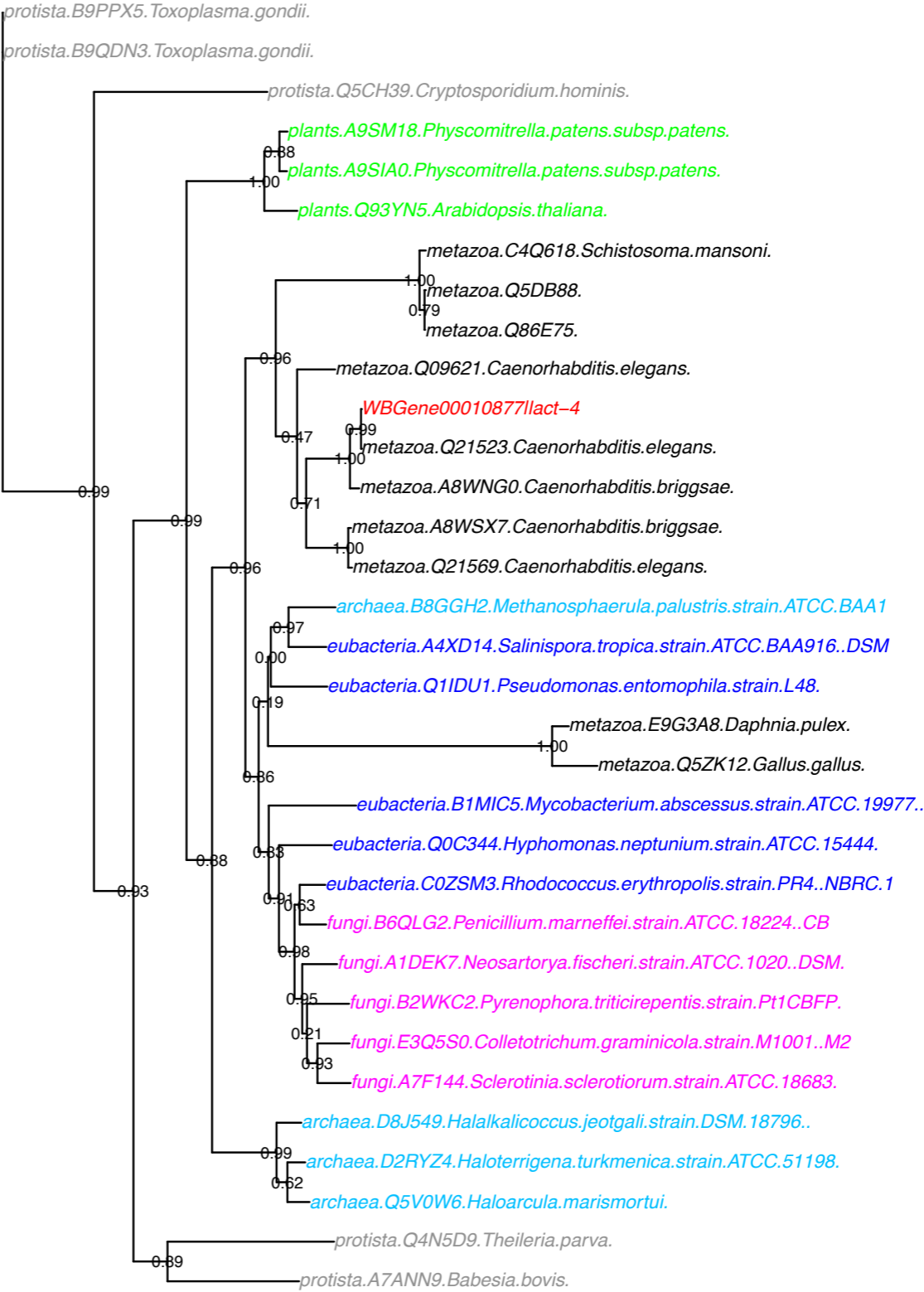

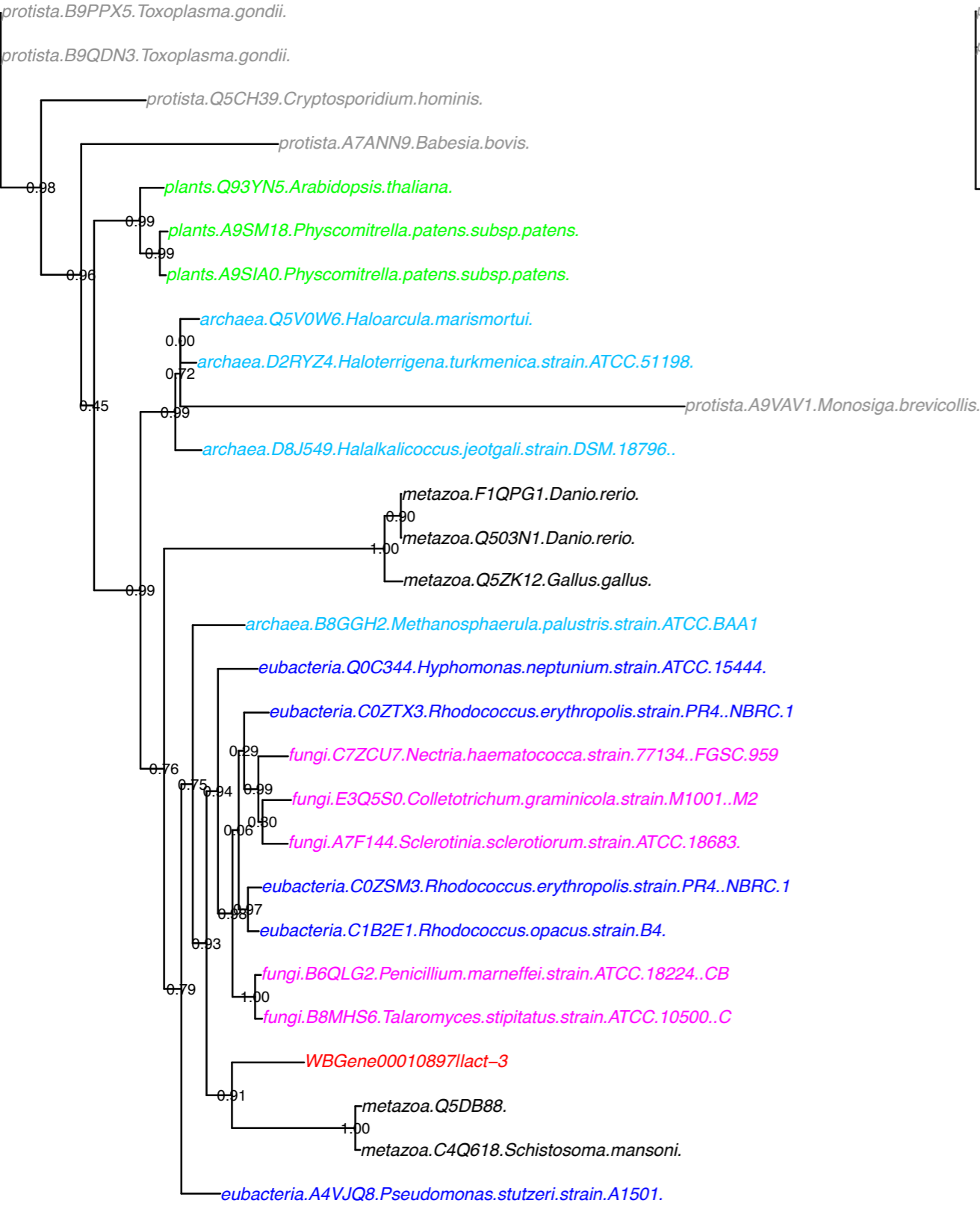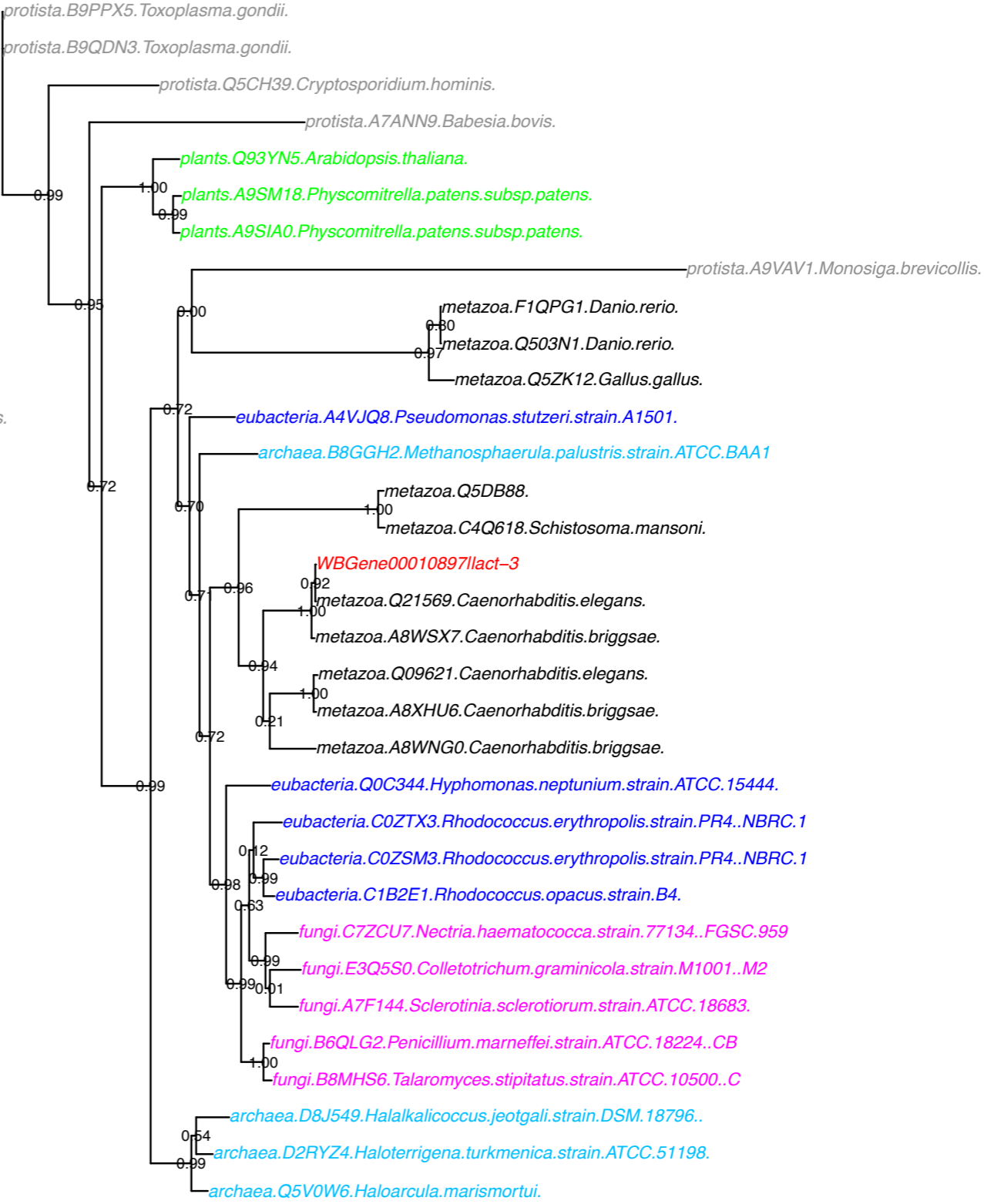

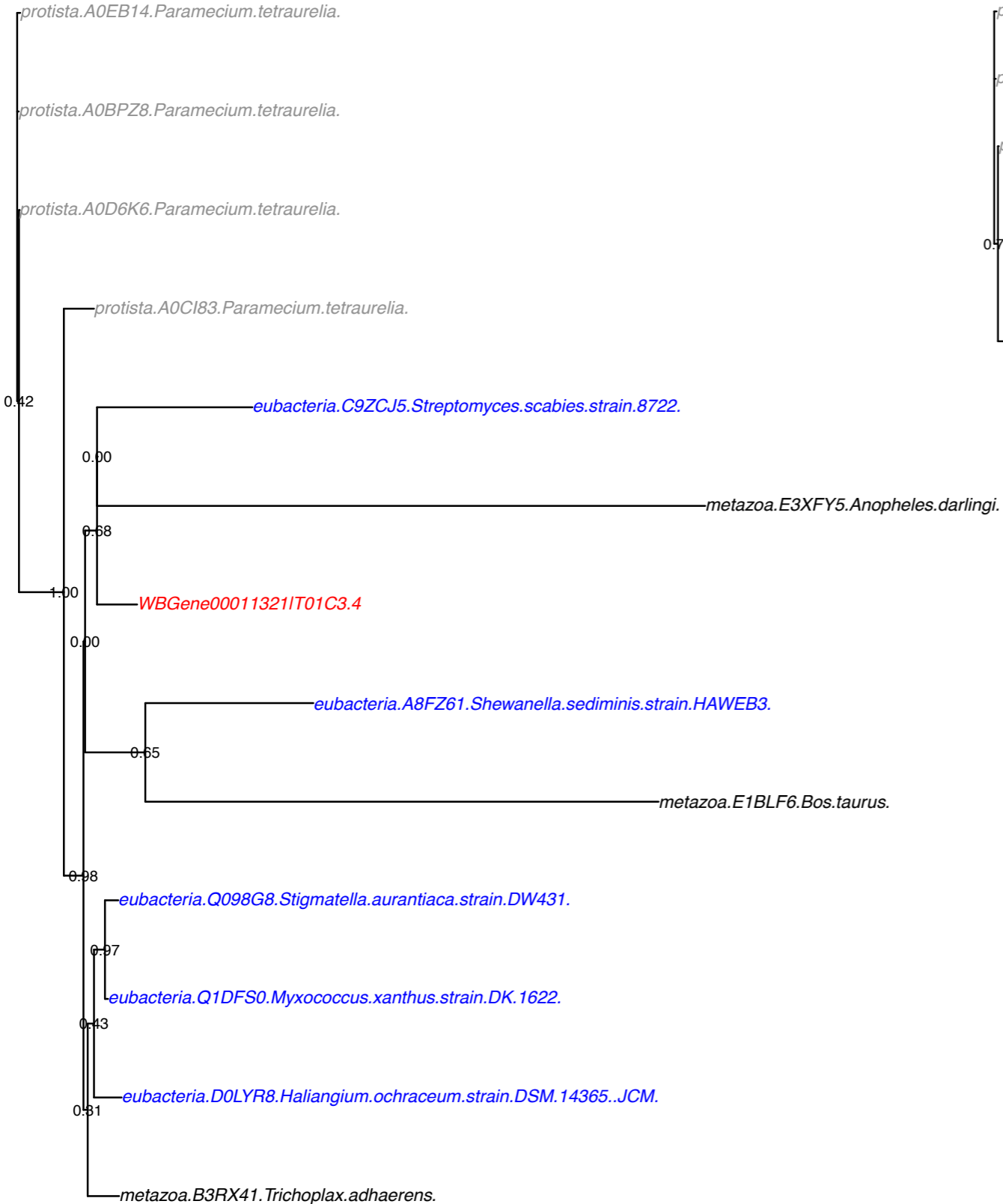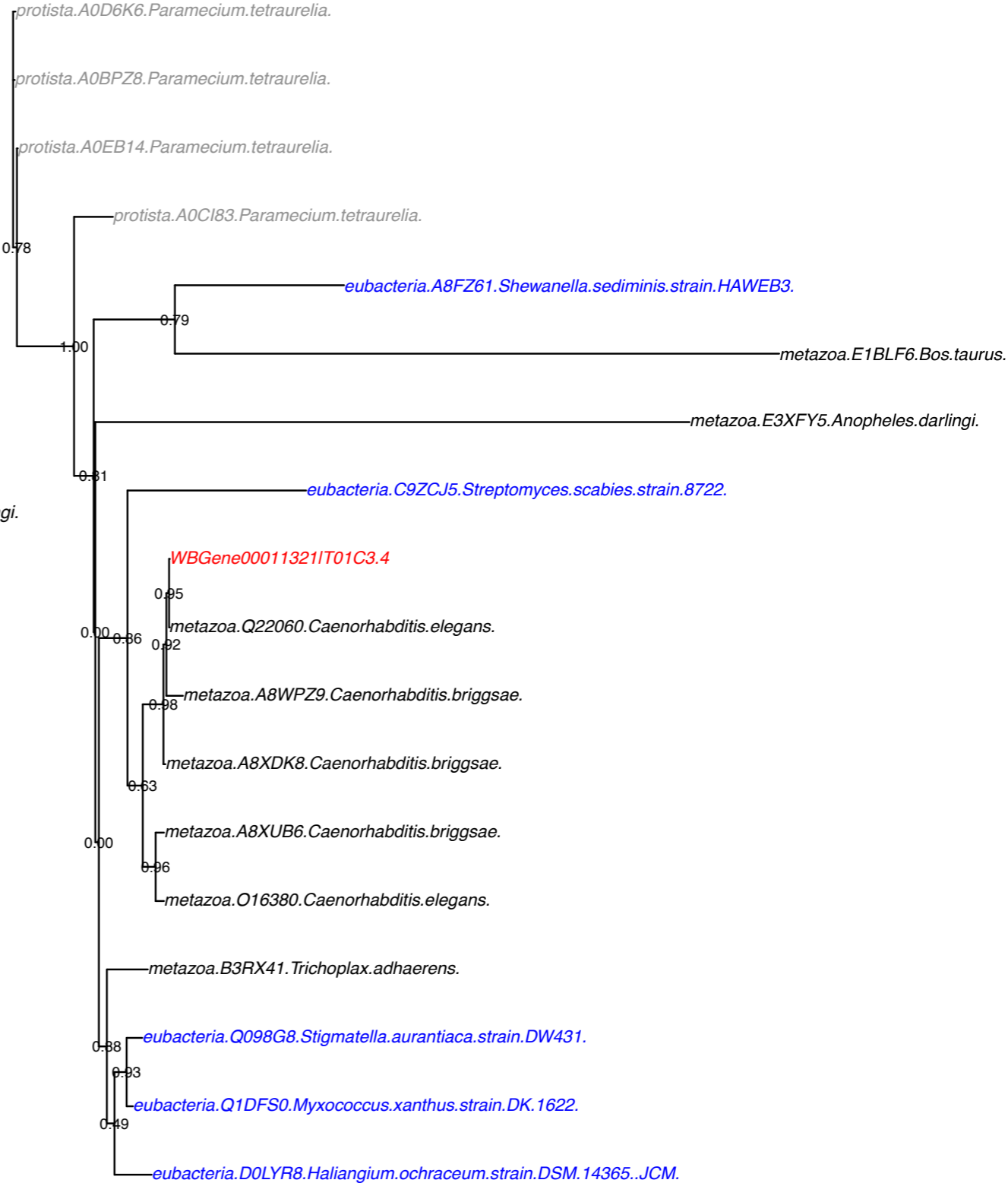

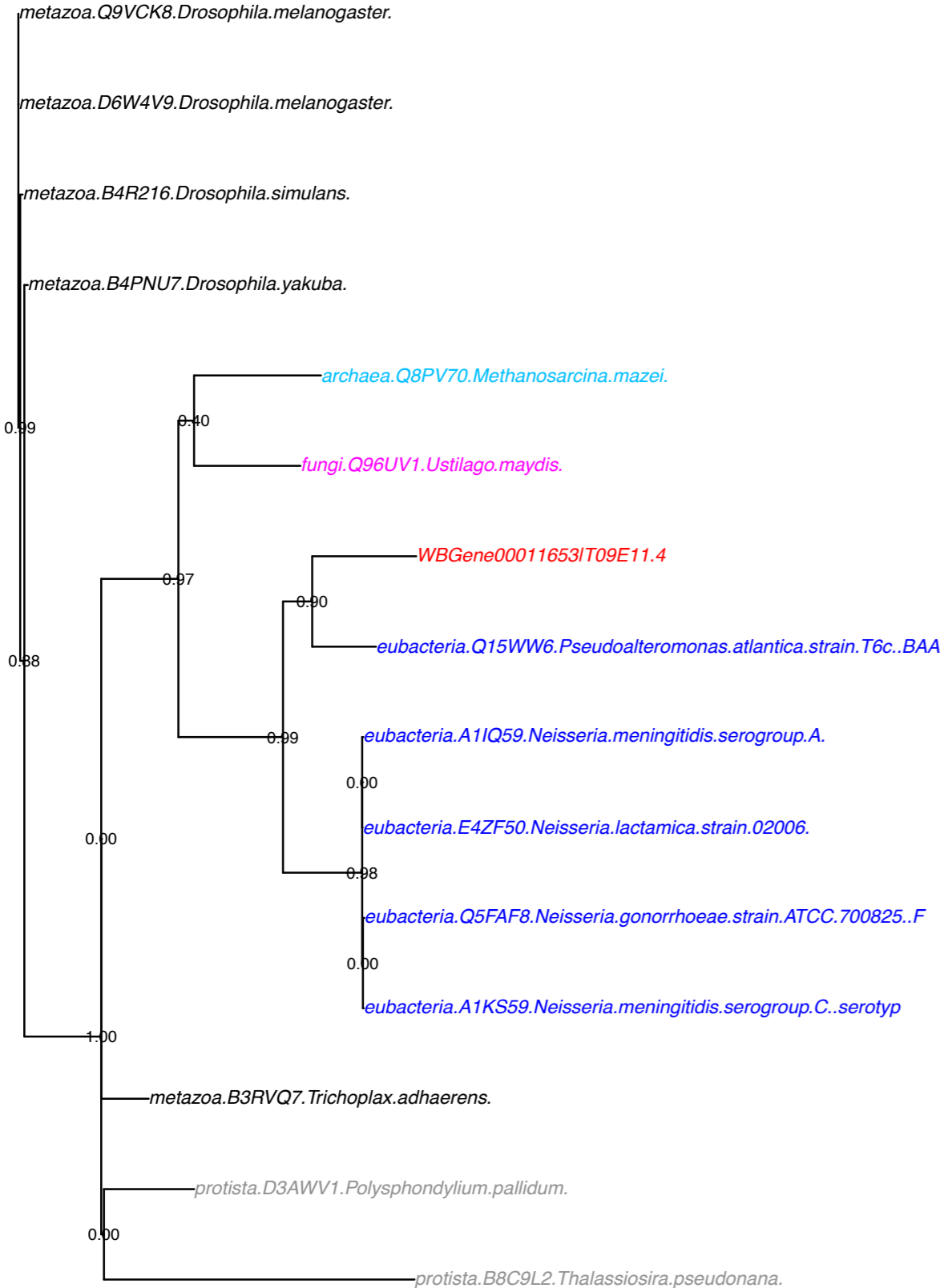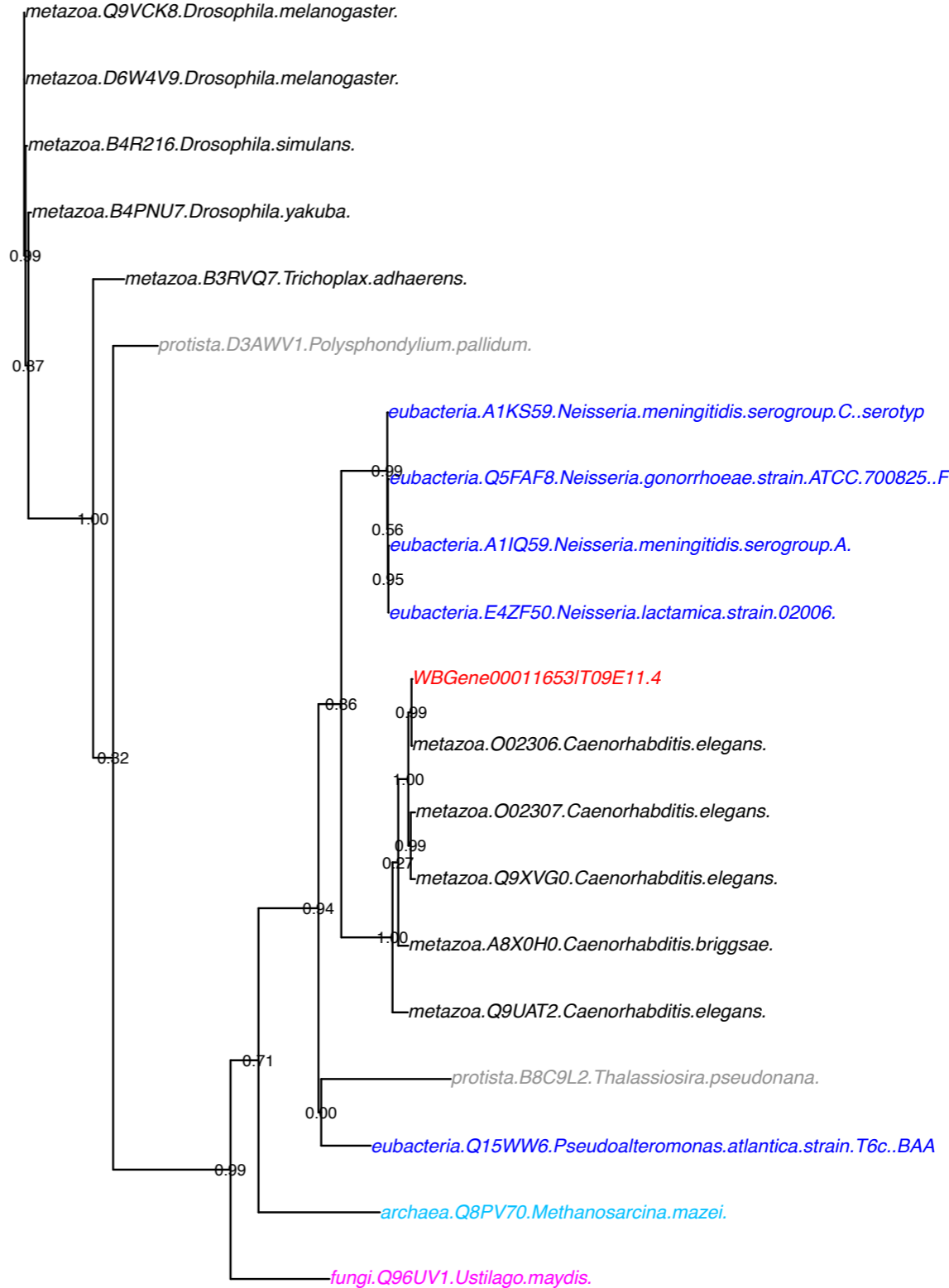

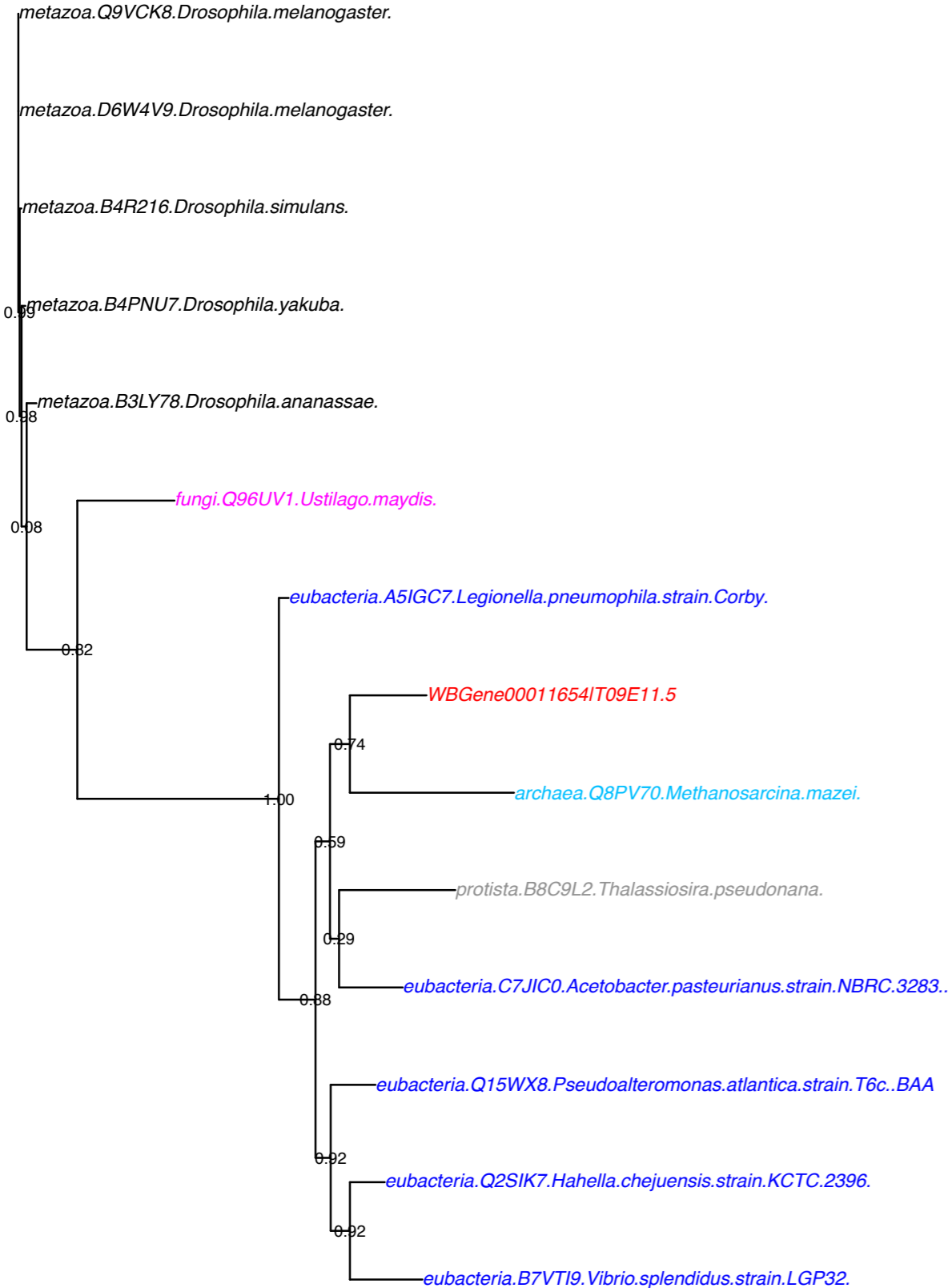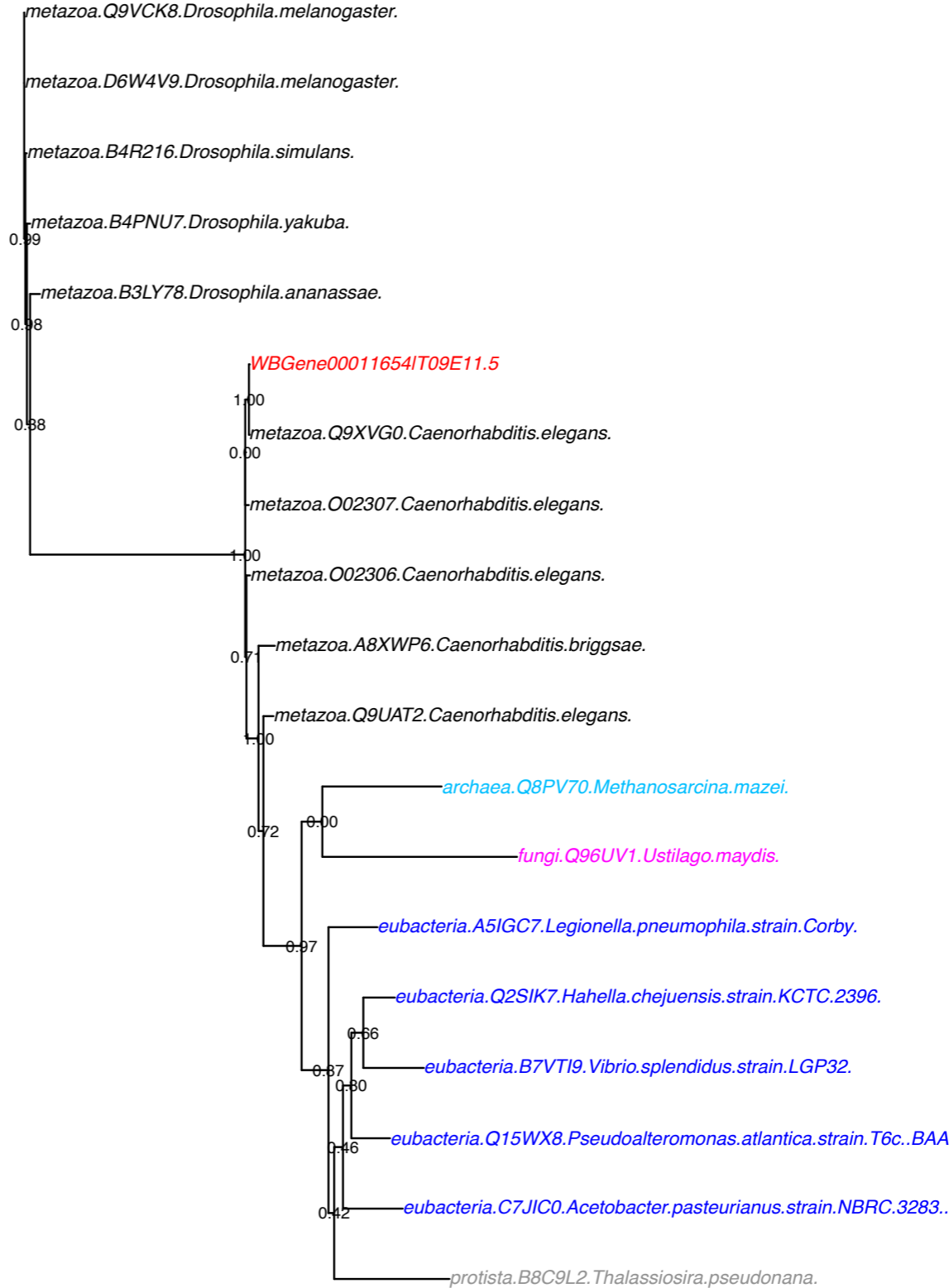

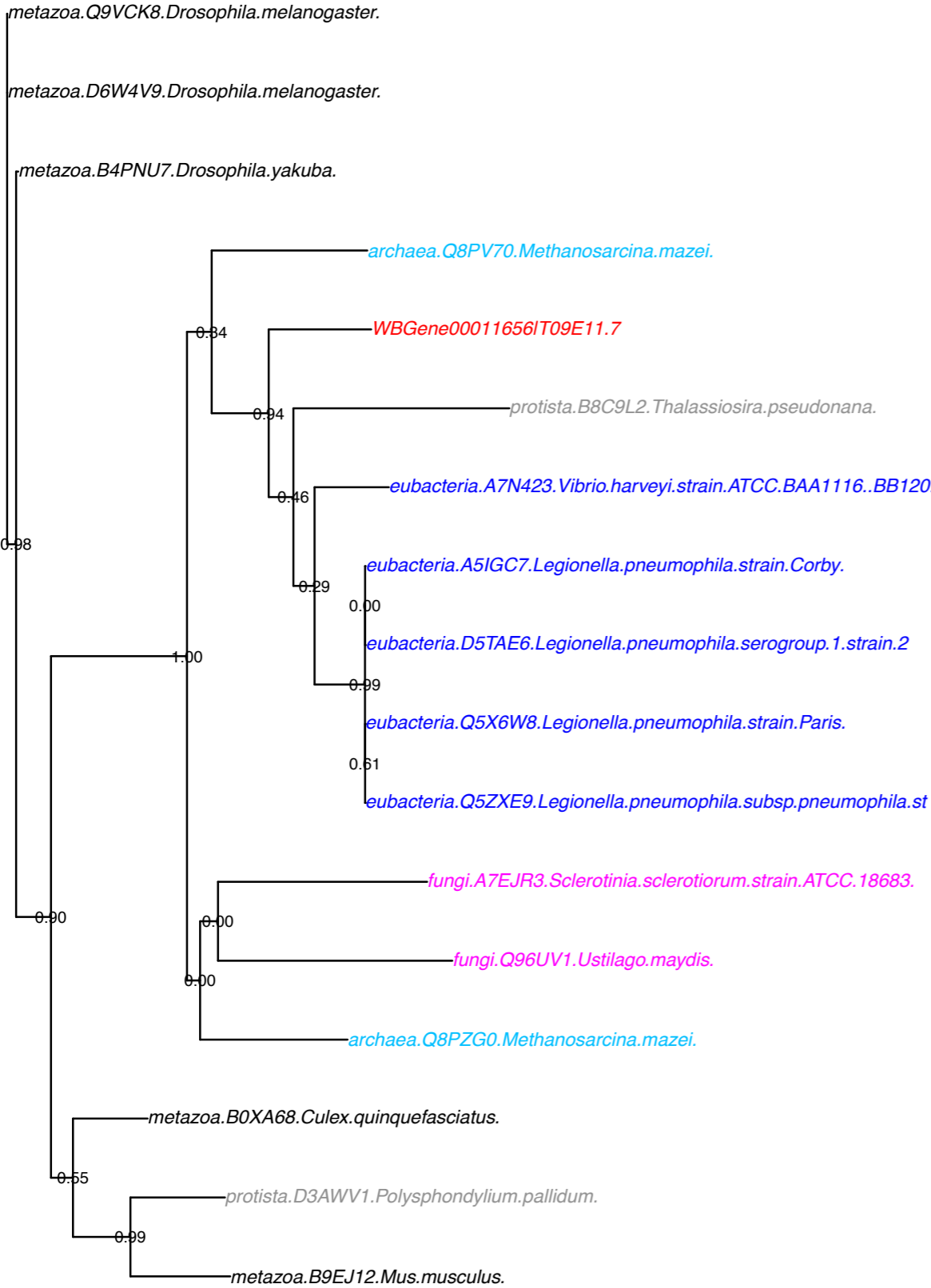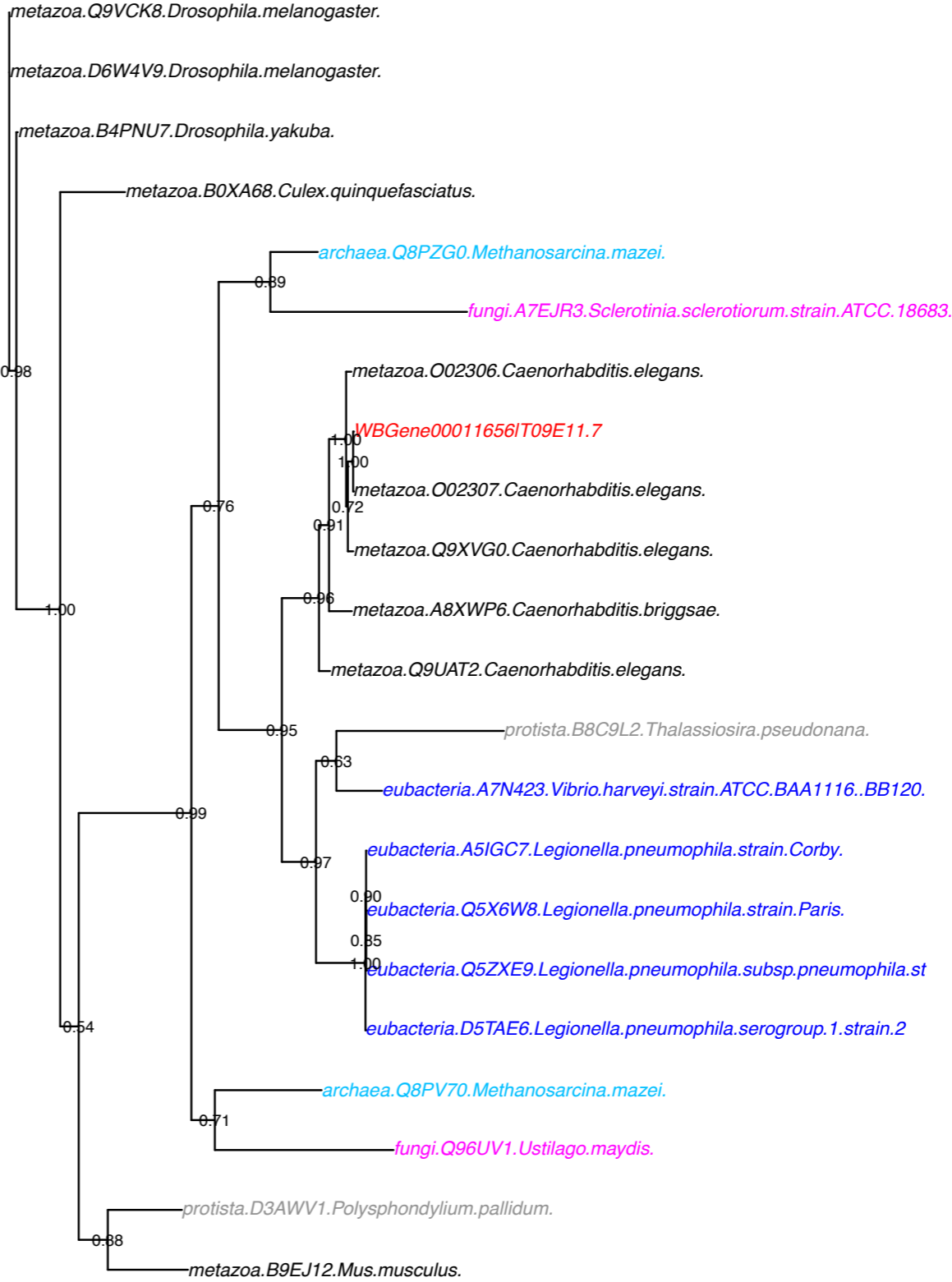

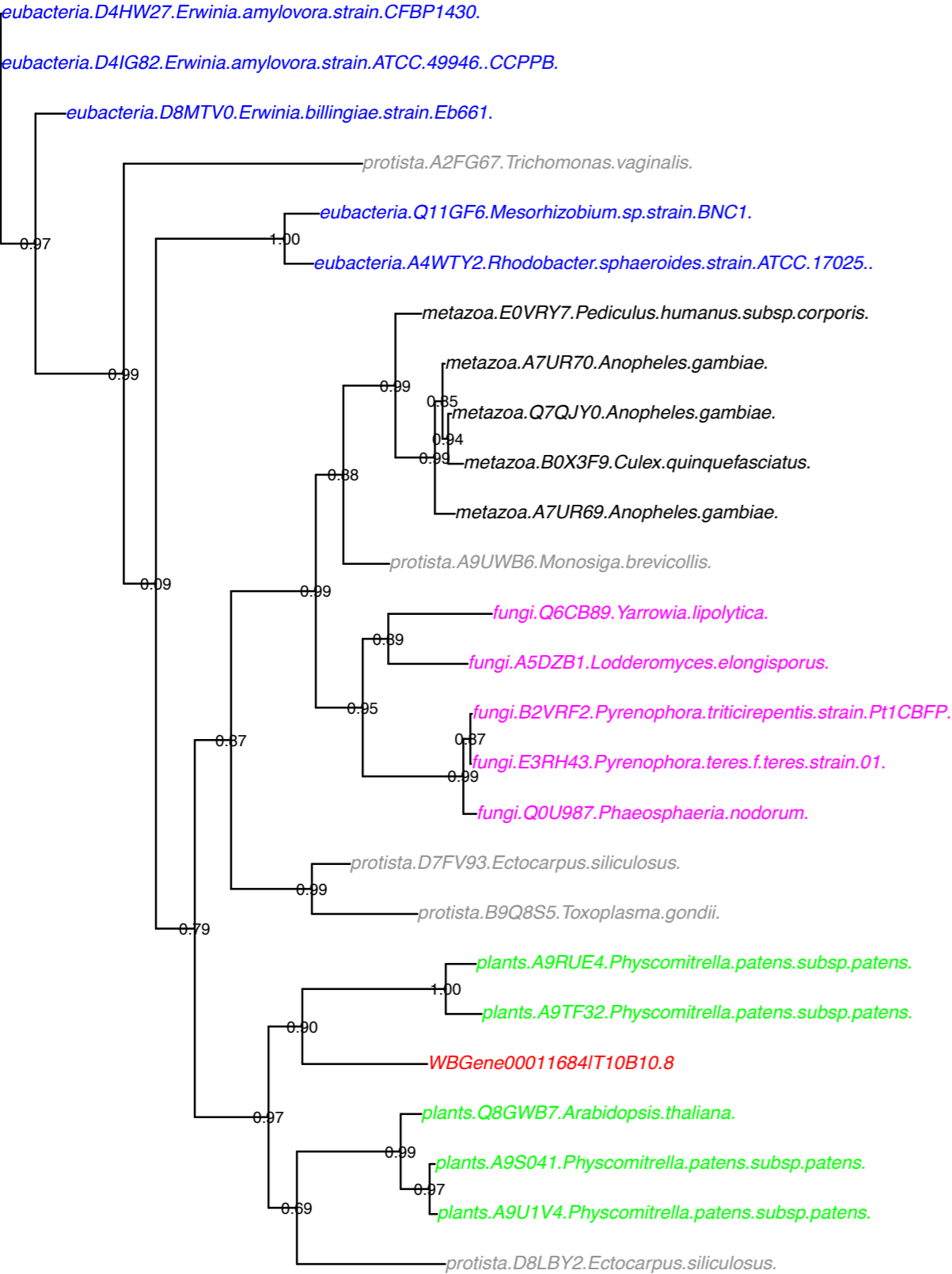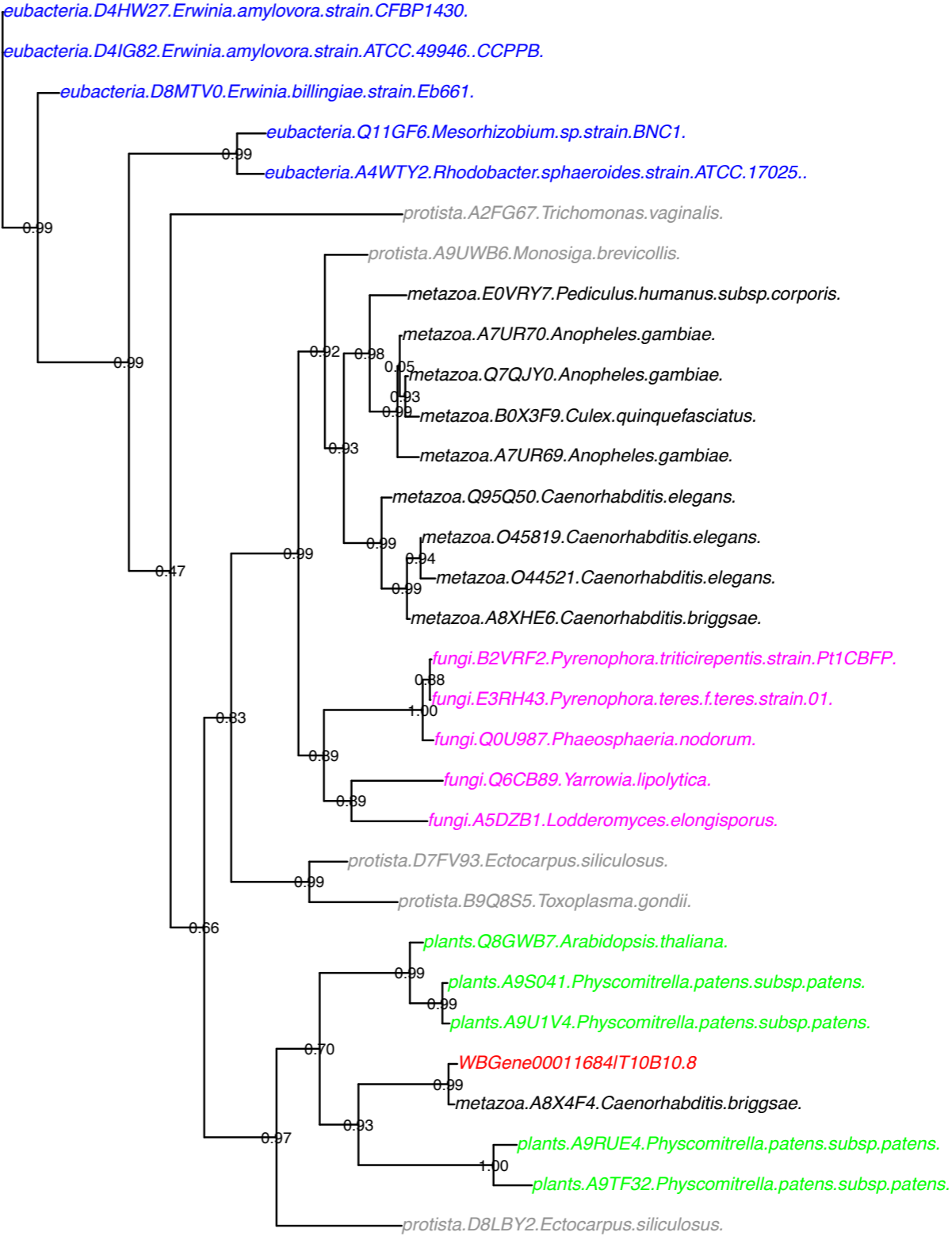

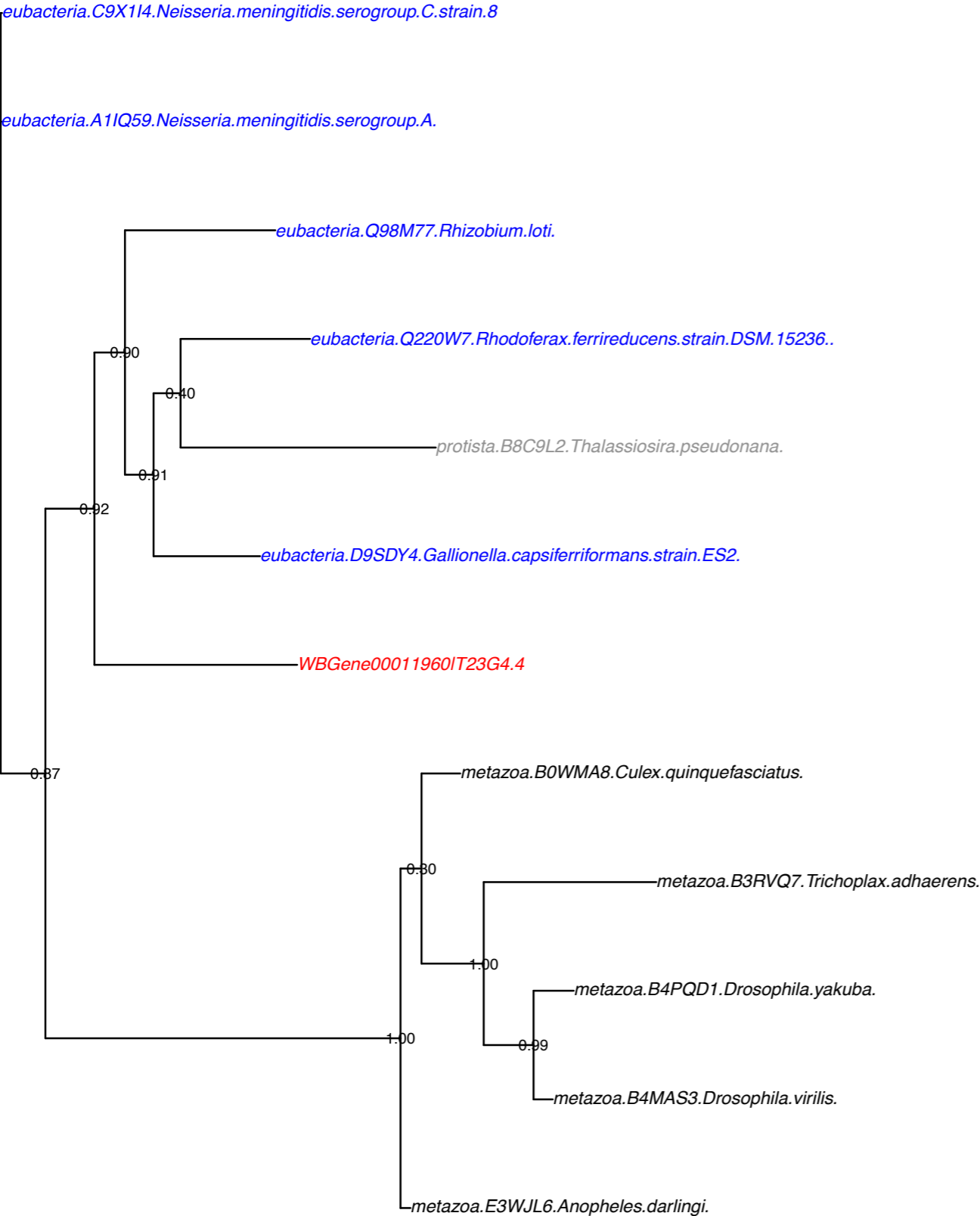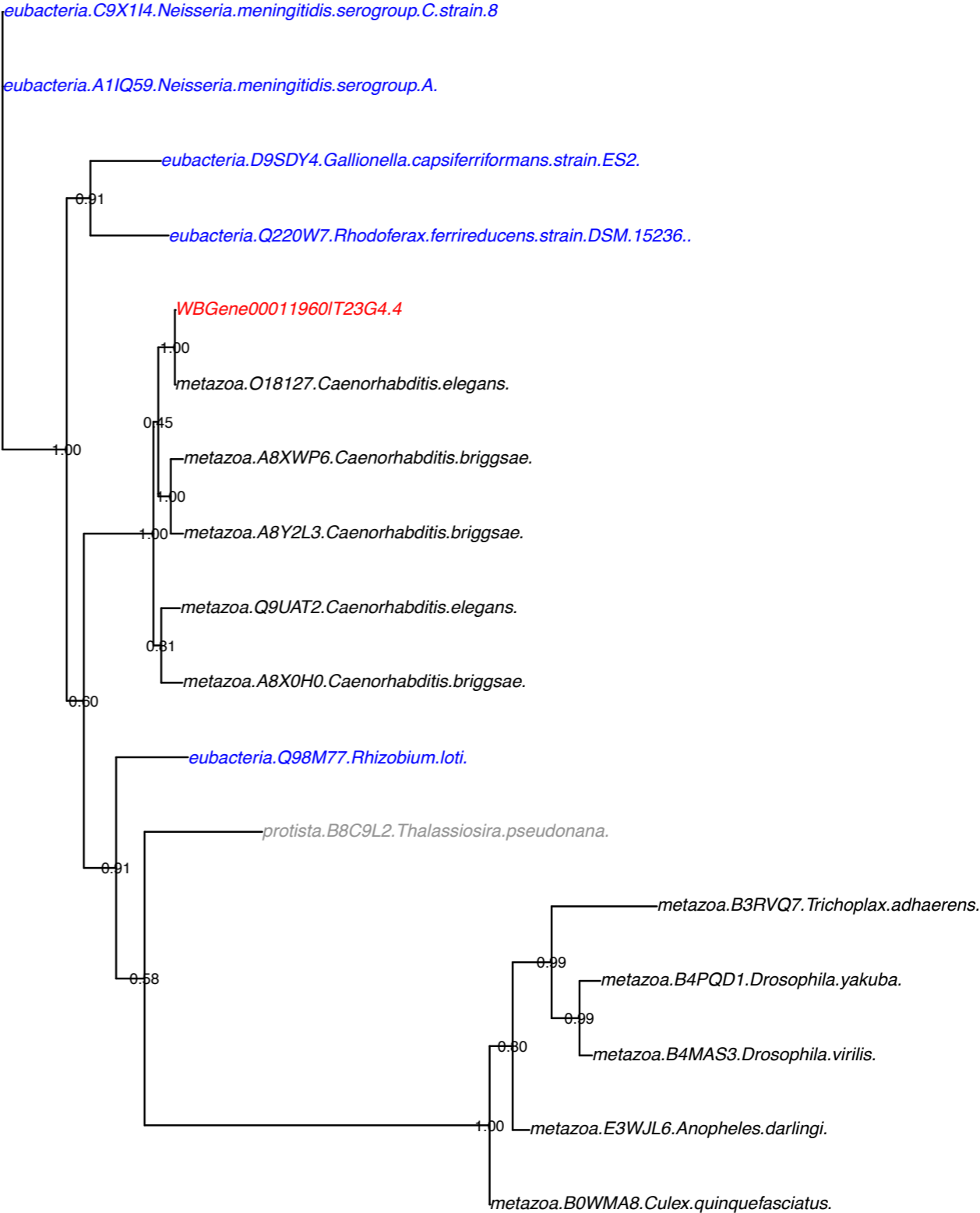

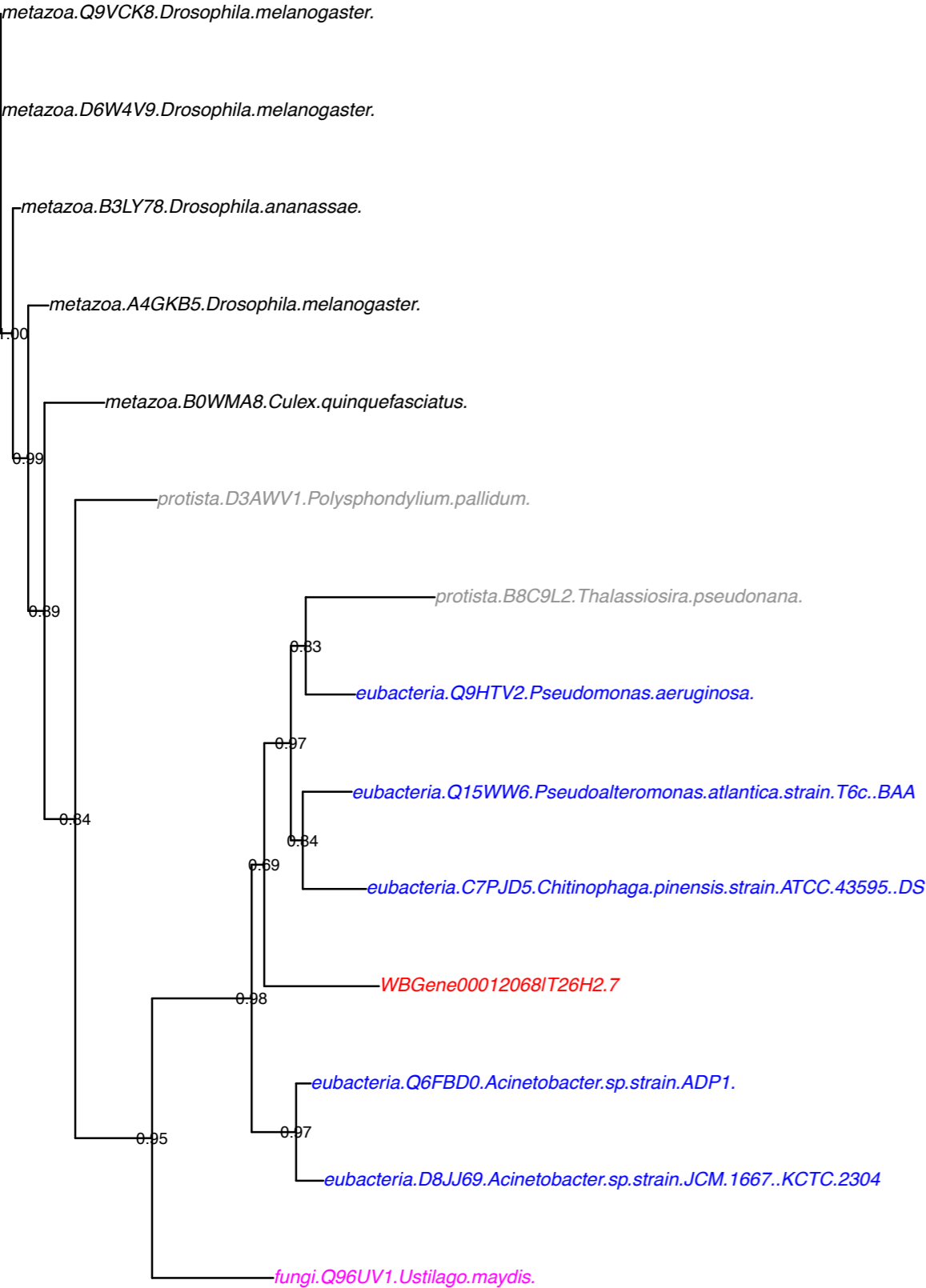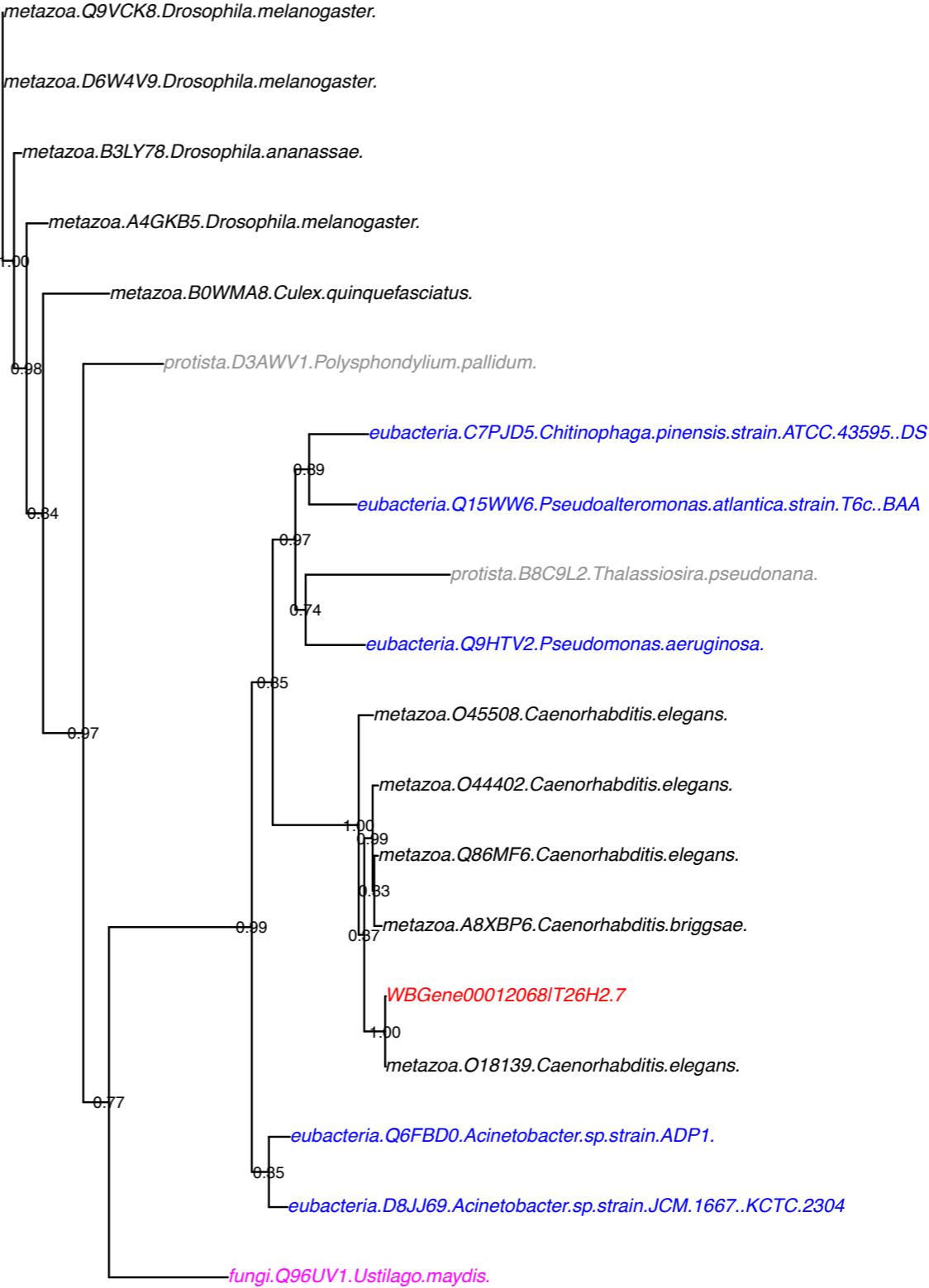

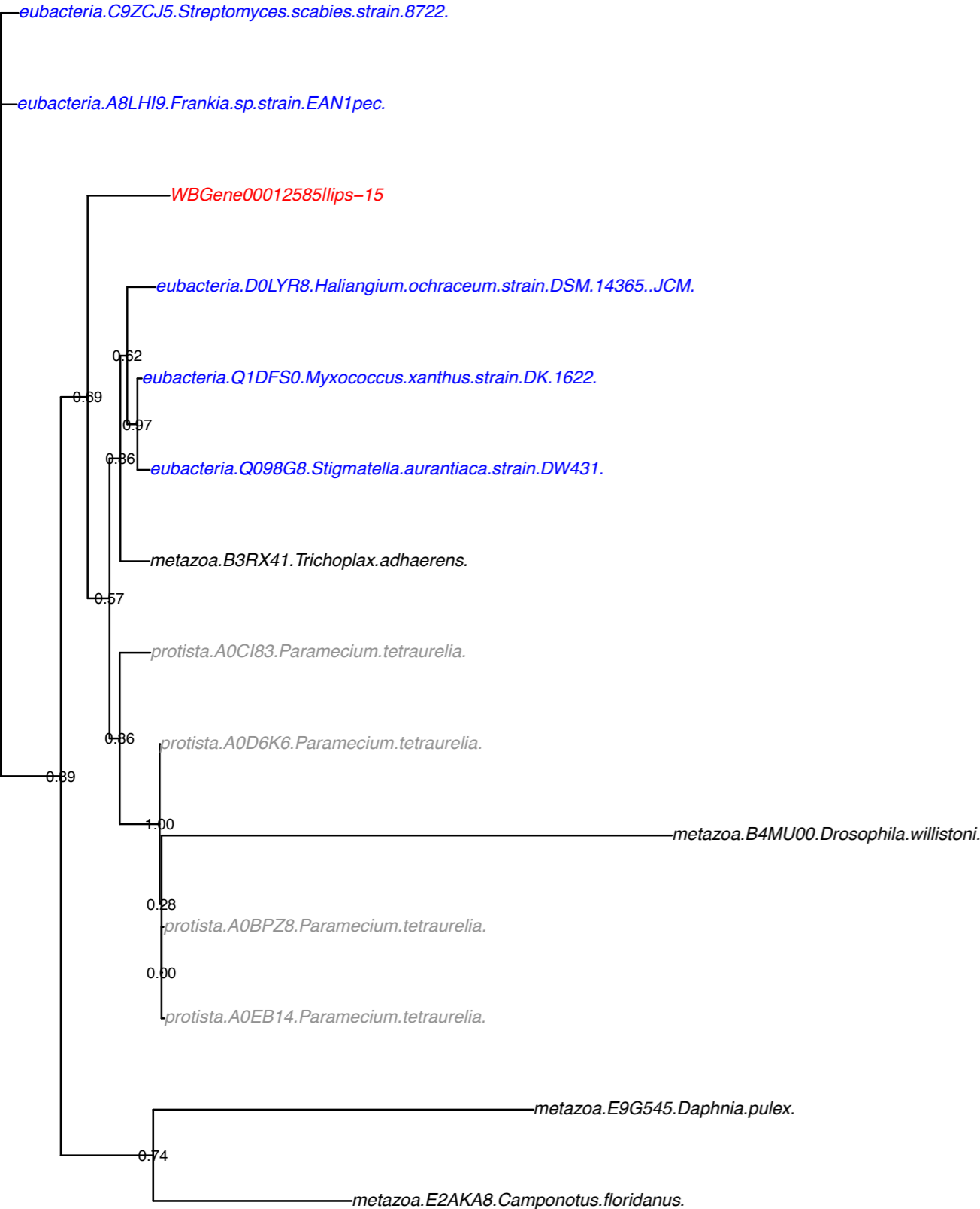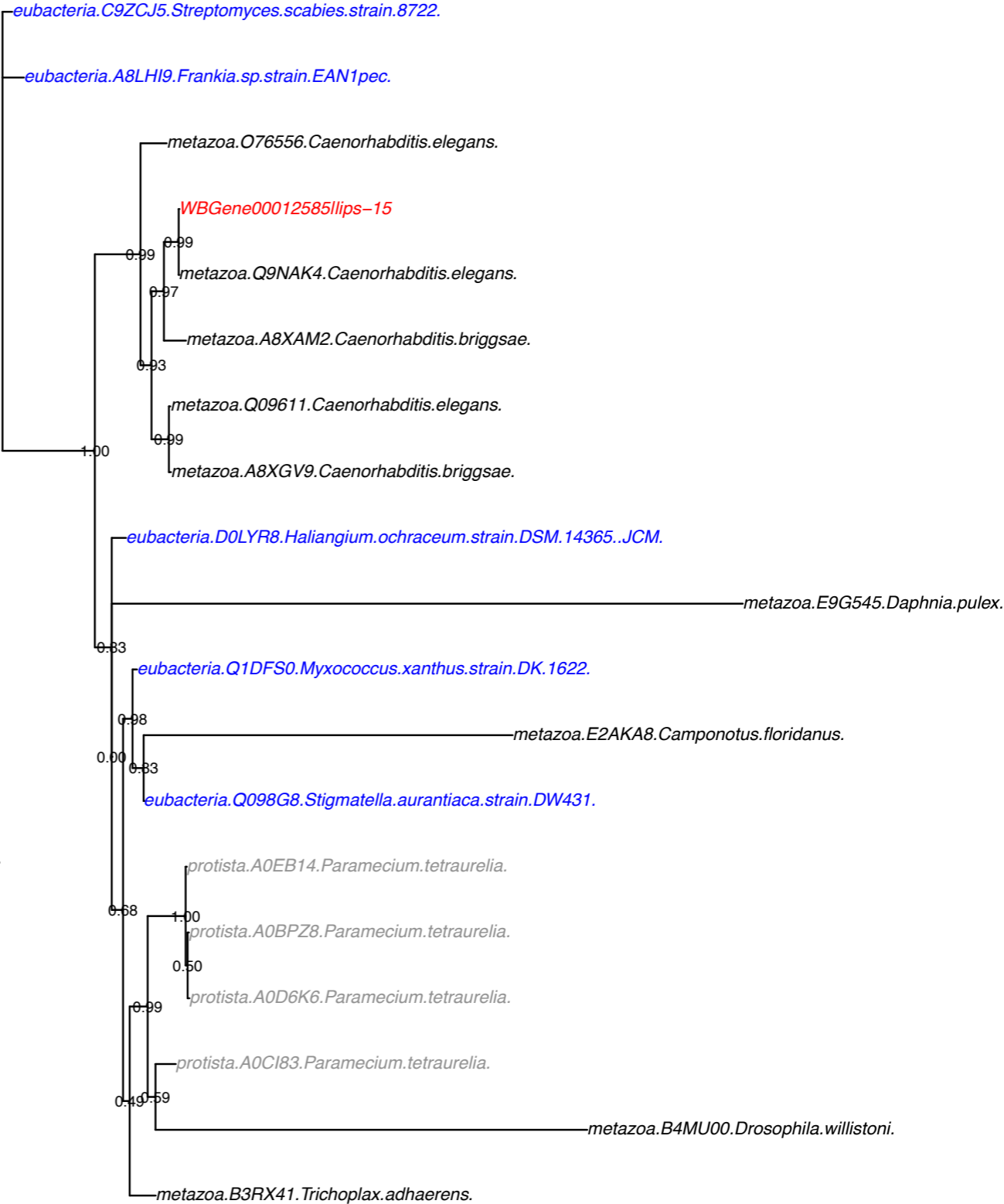

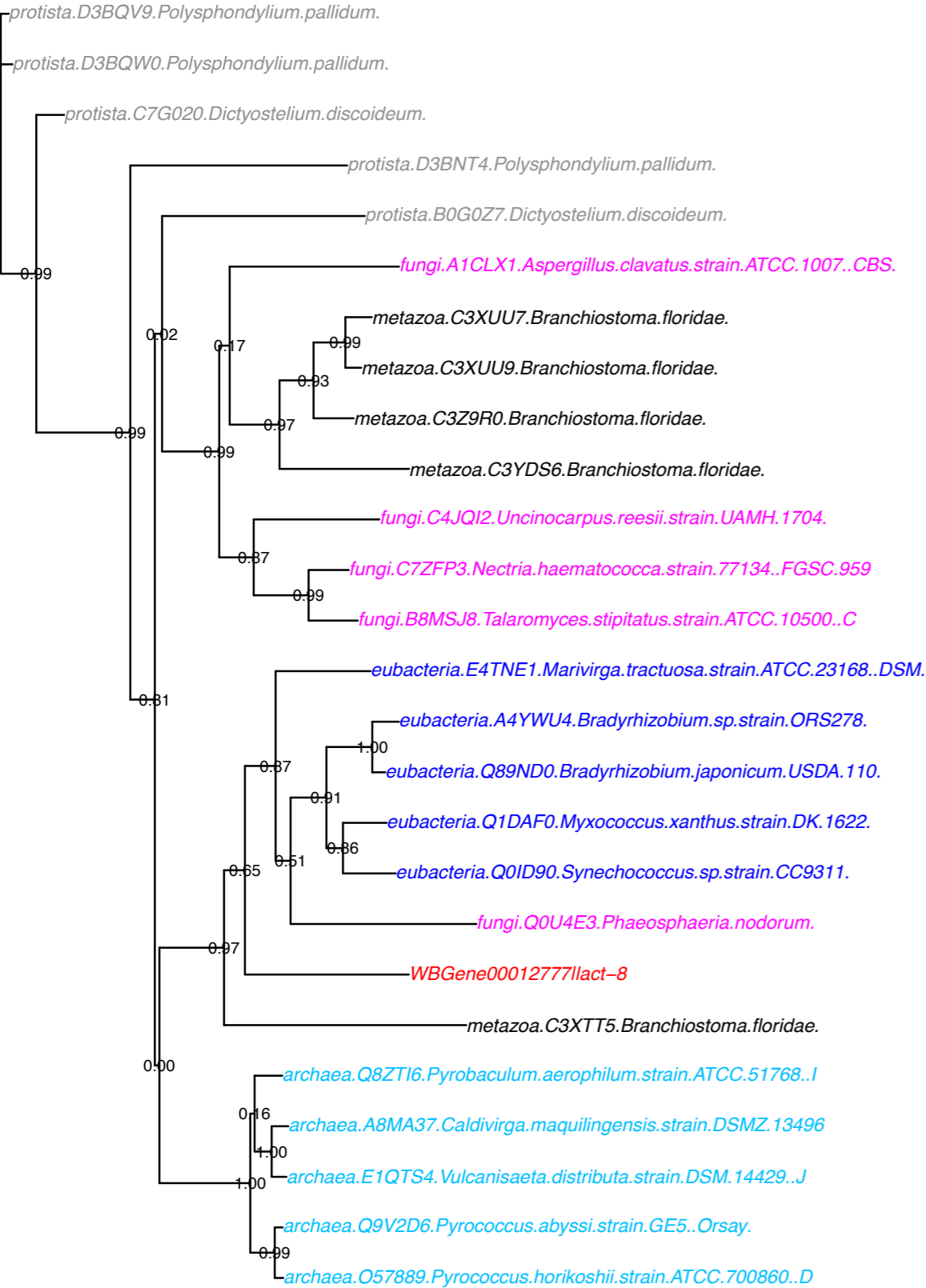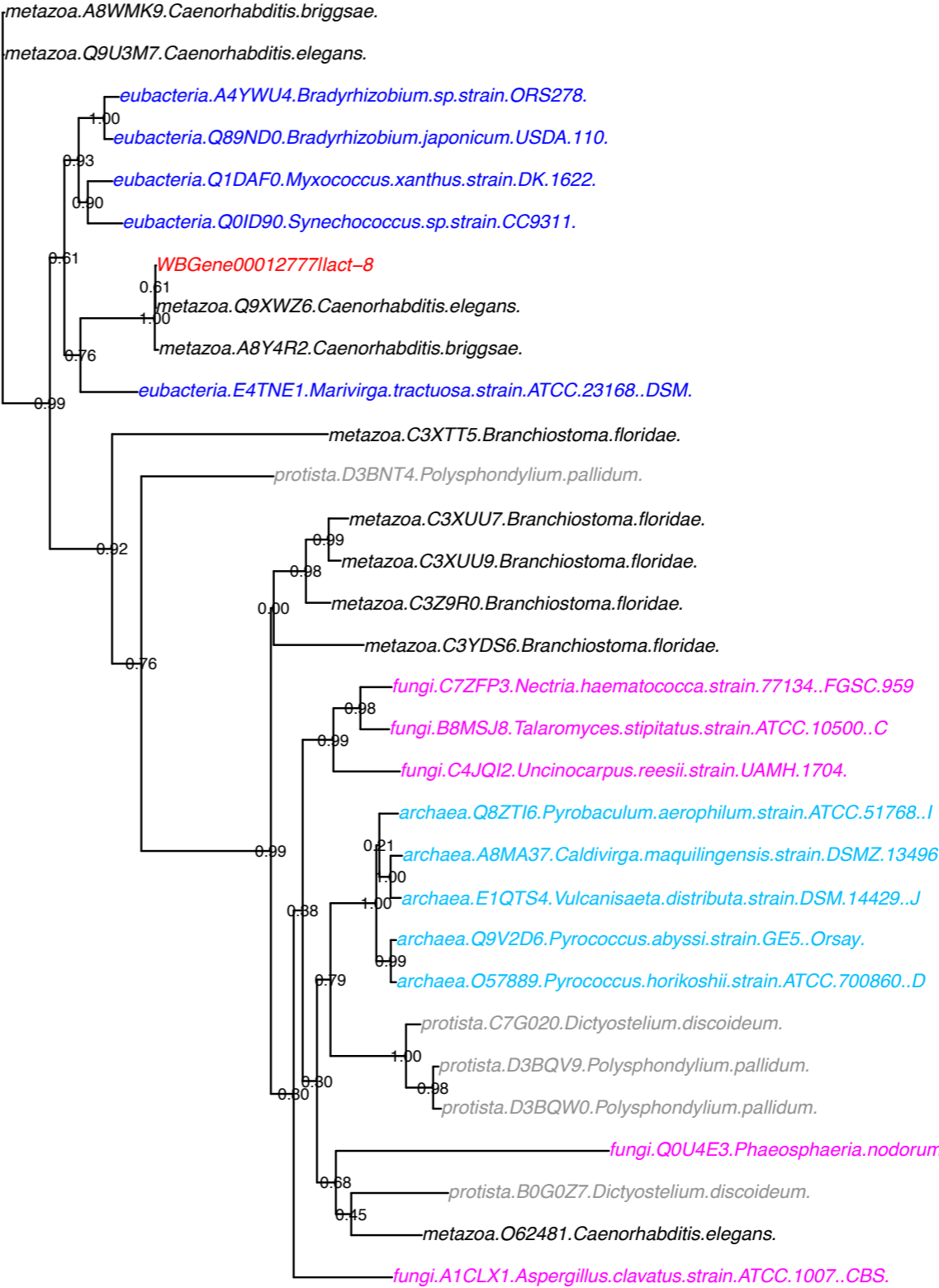

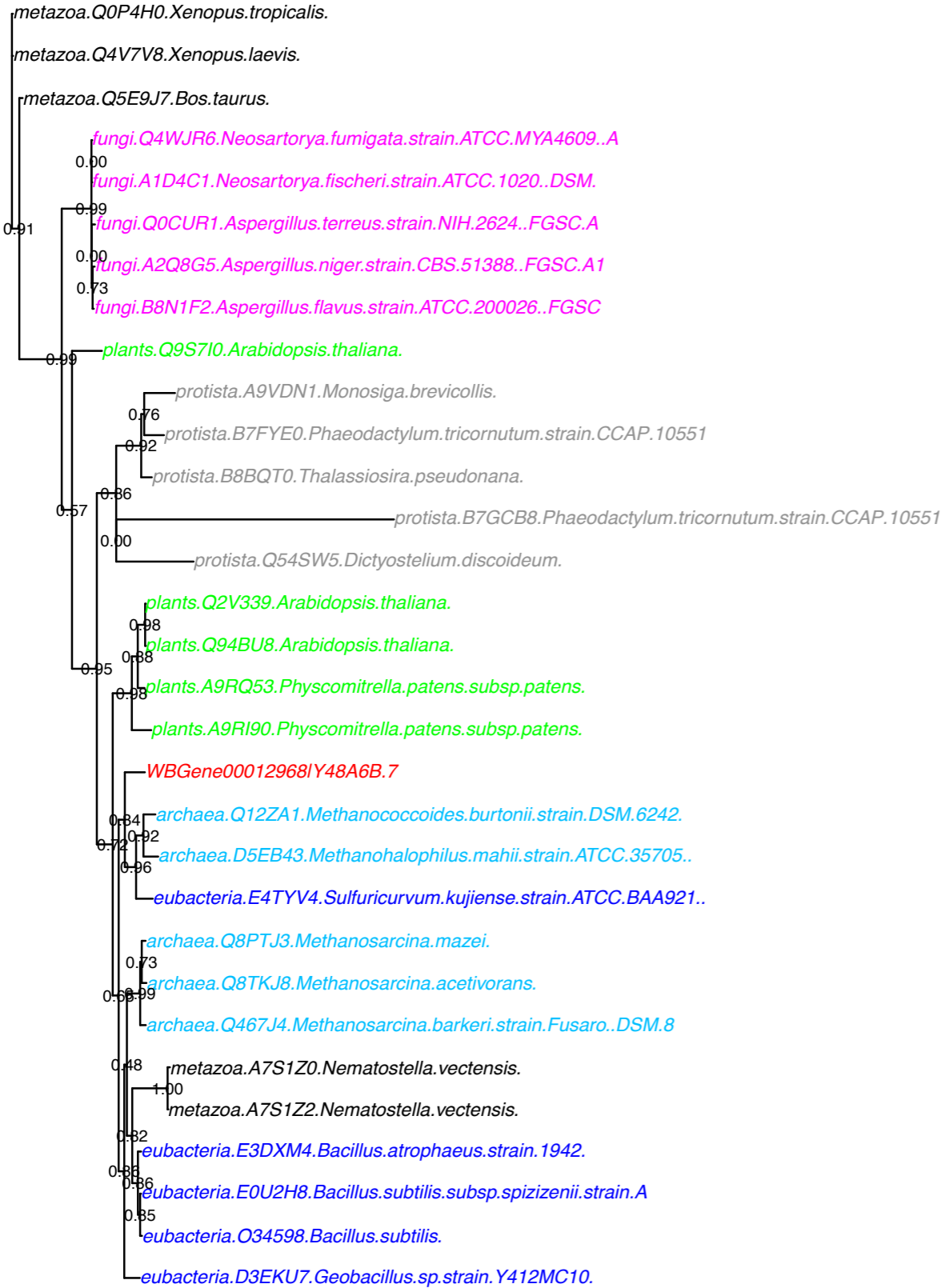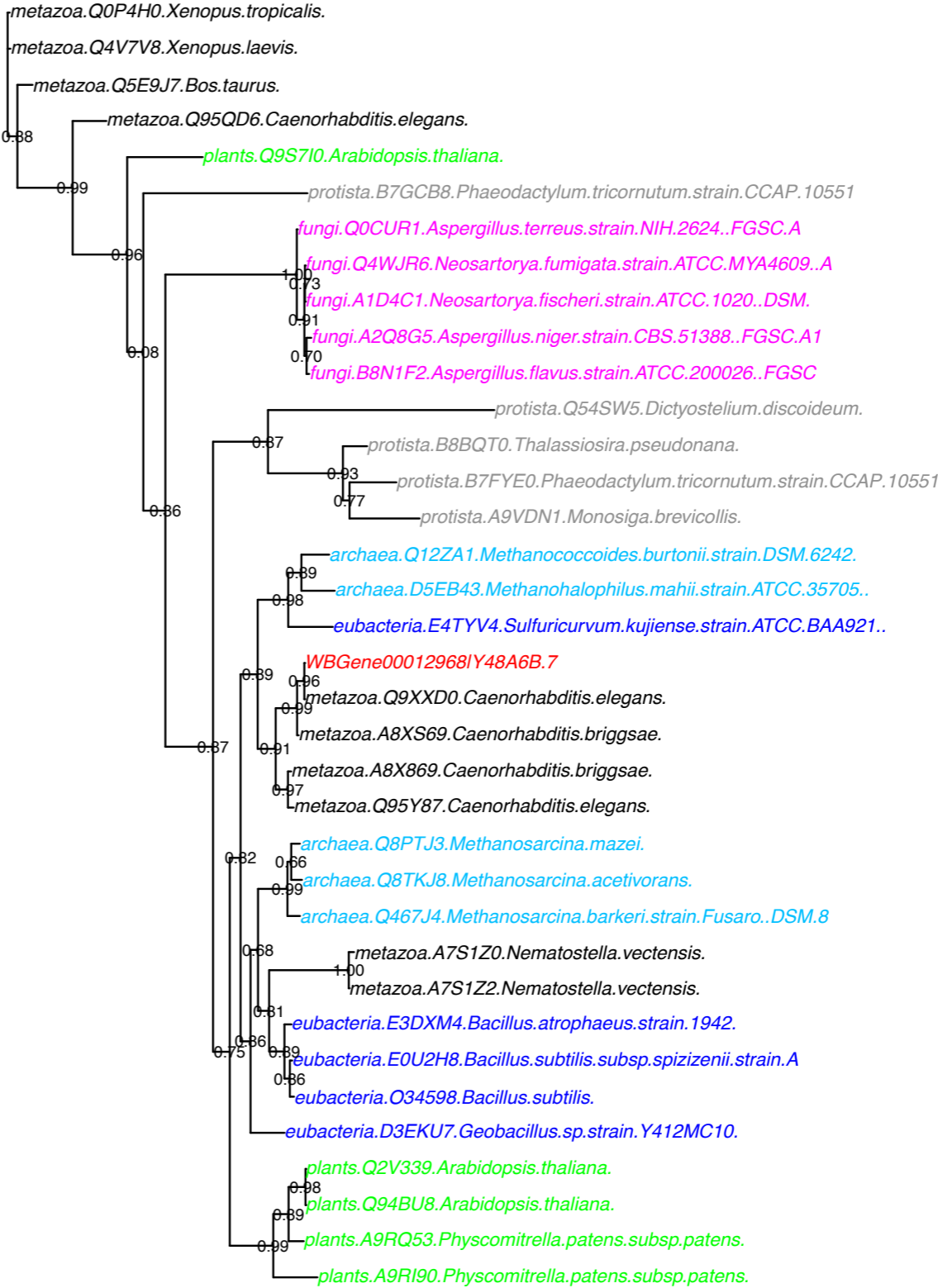

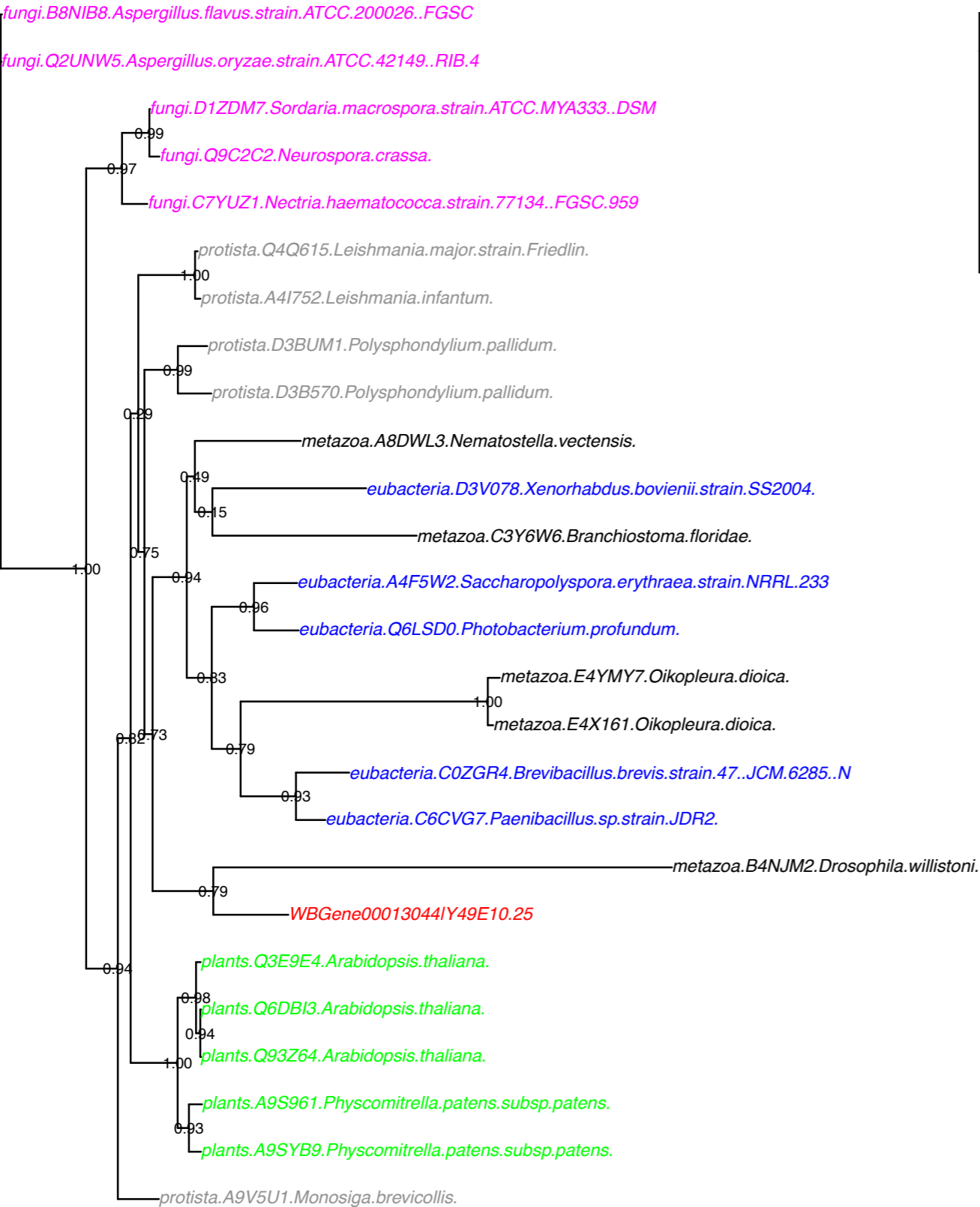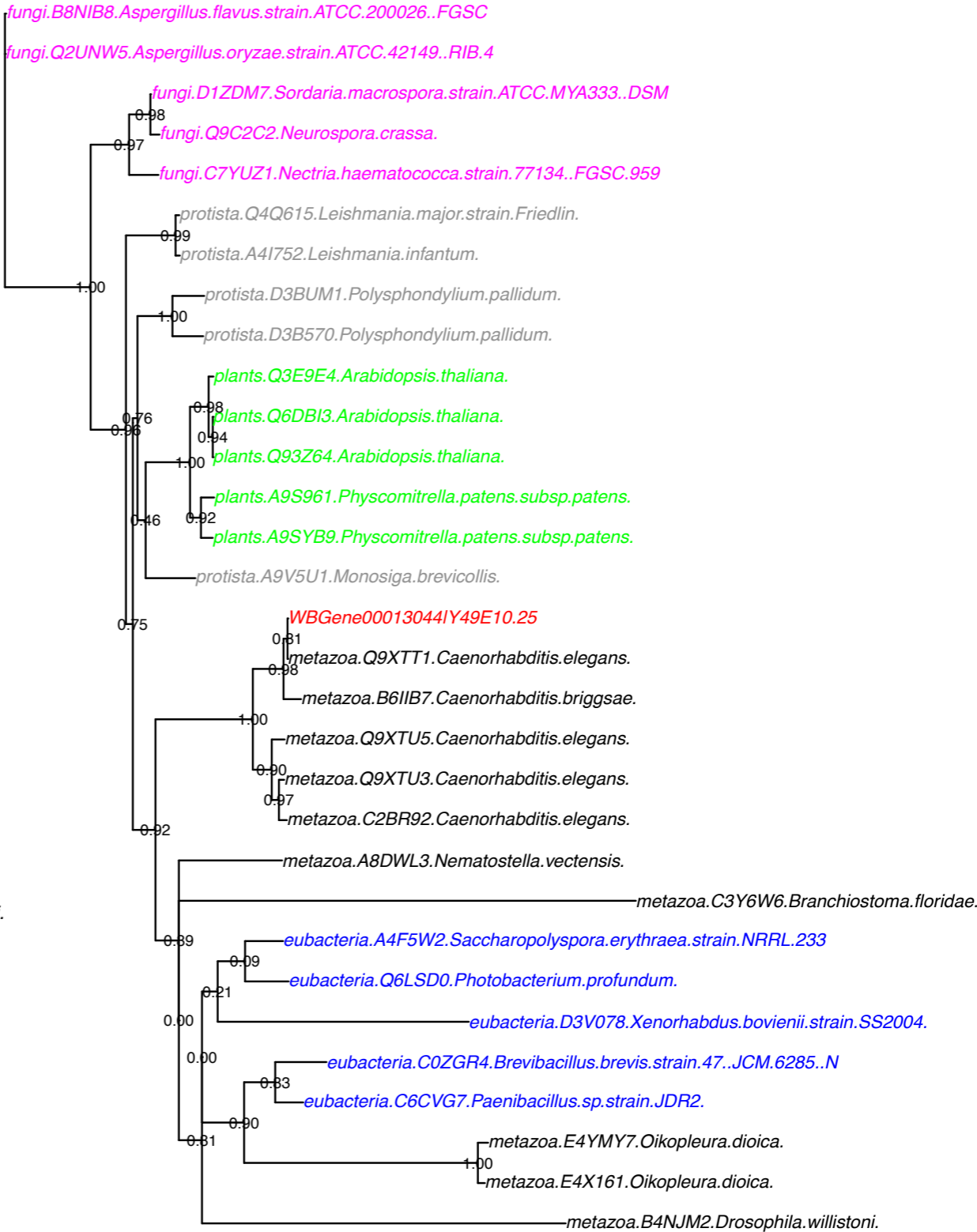

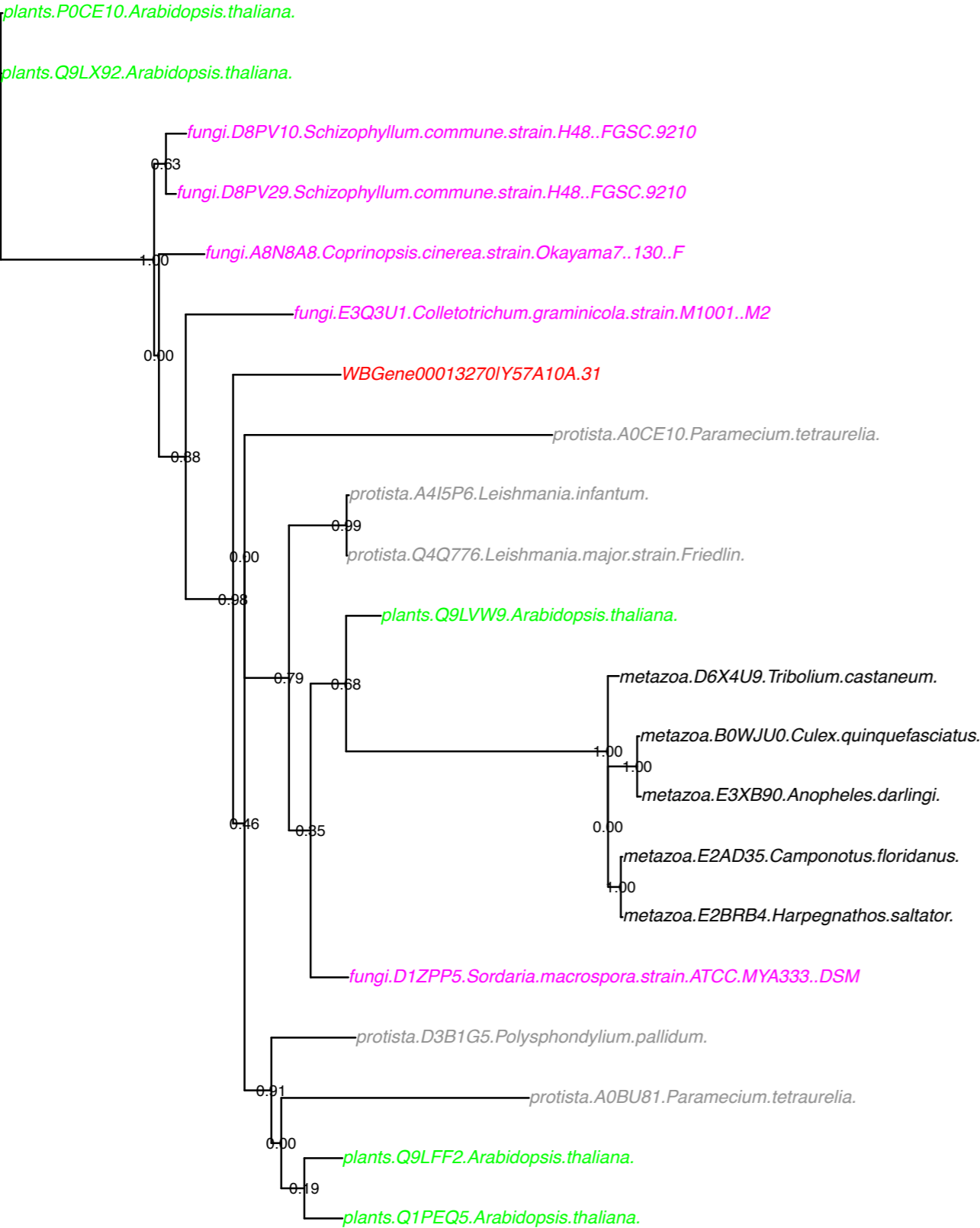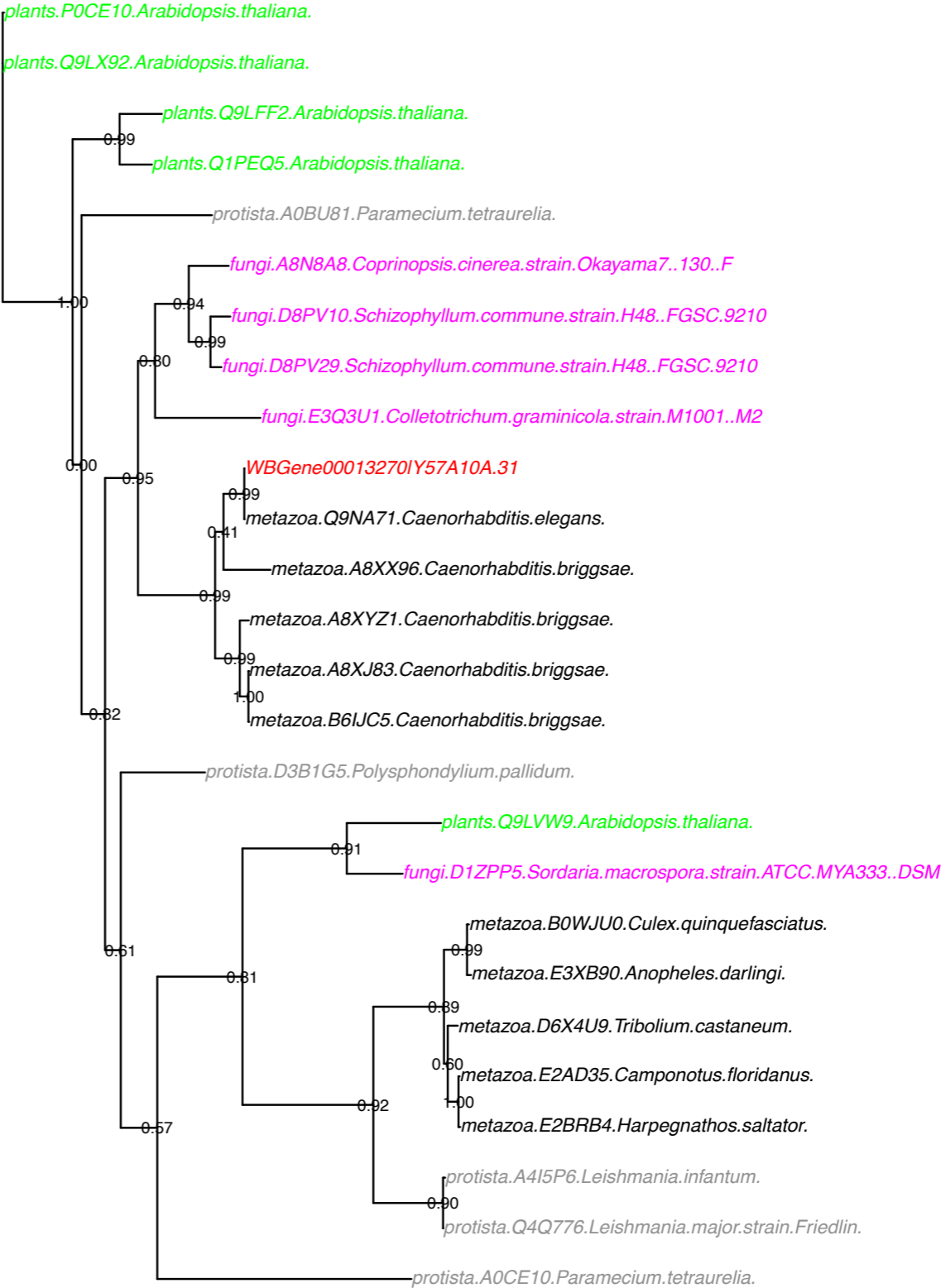

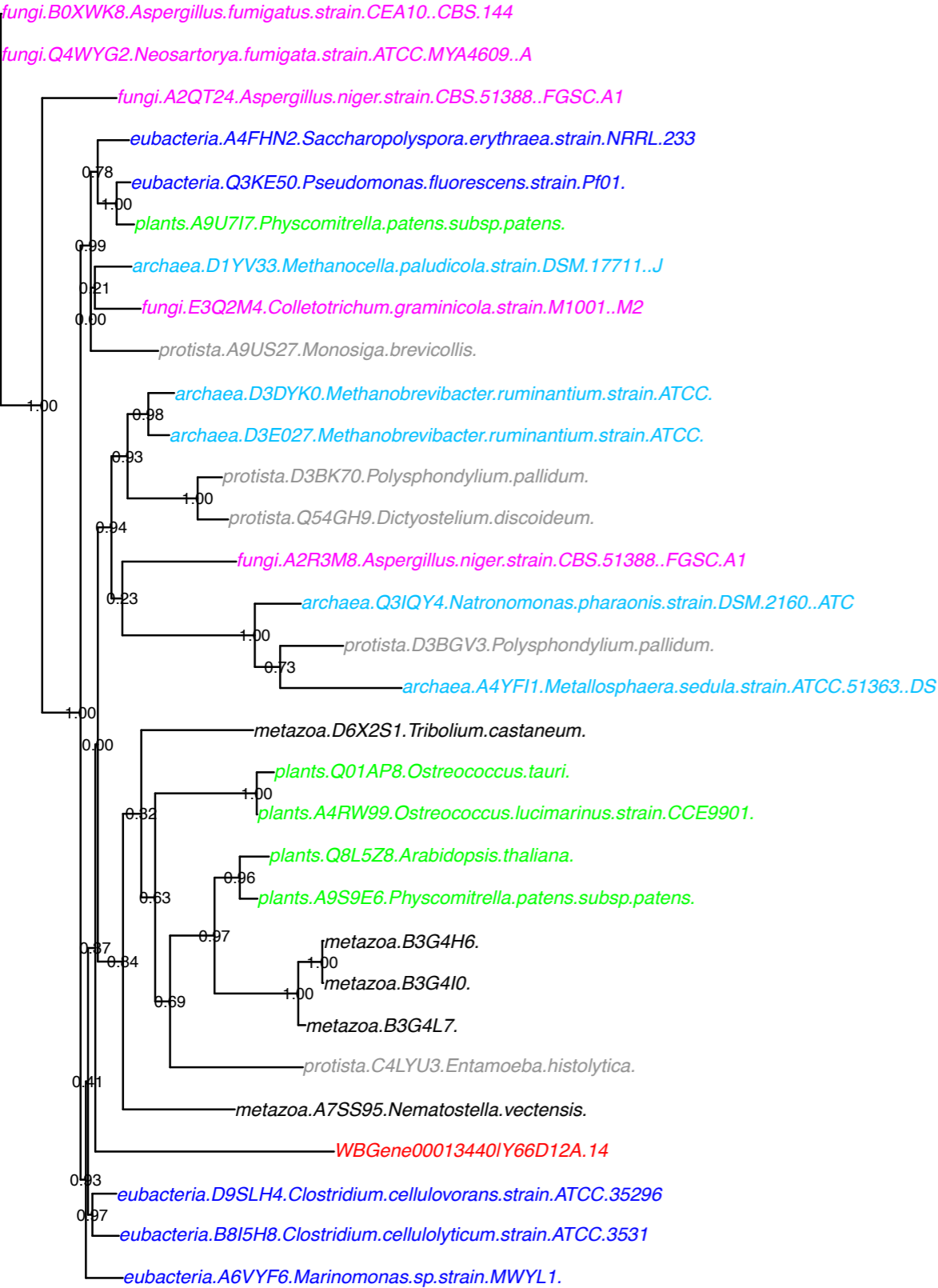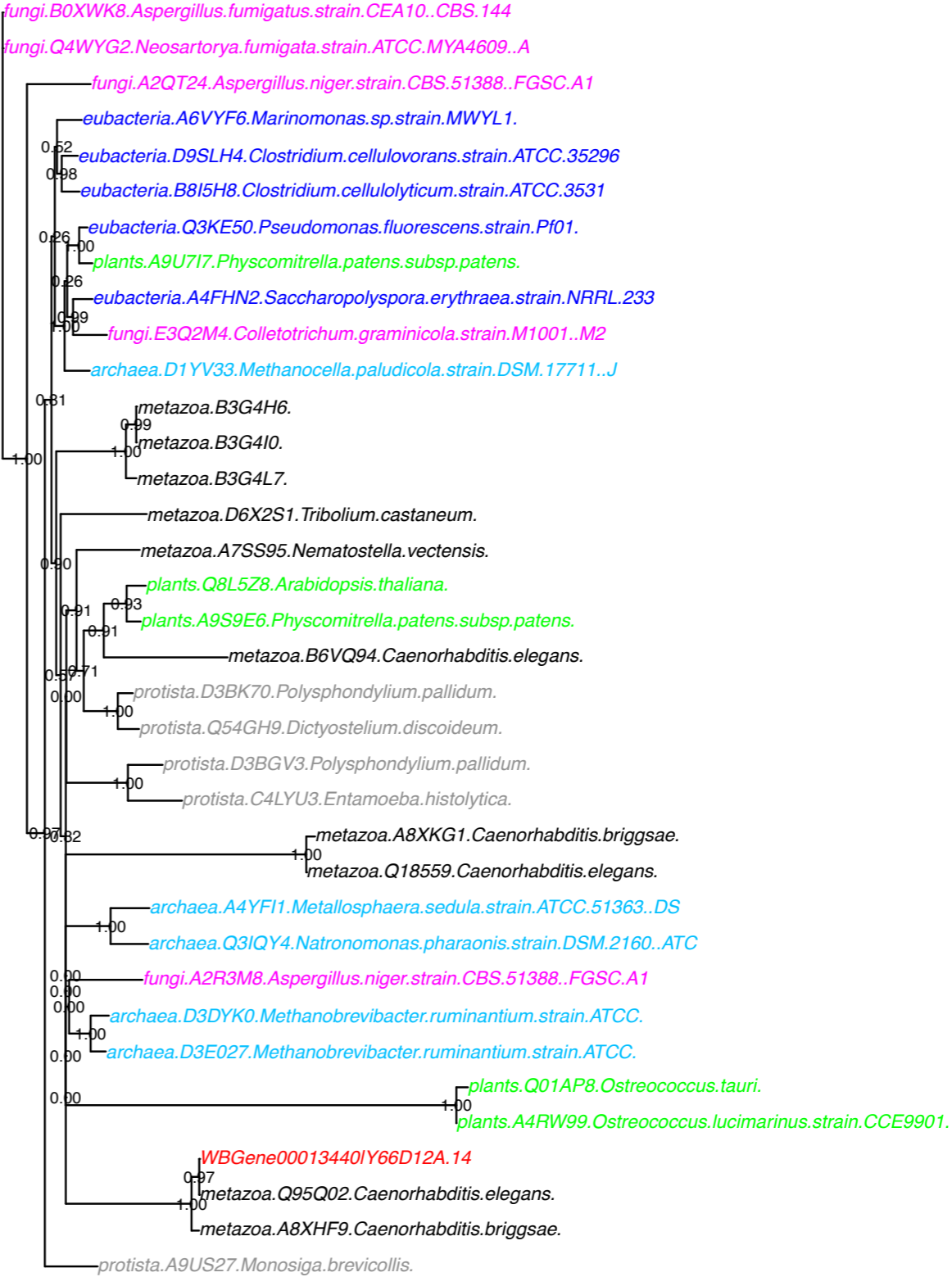

transcript  
100

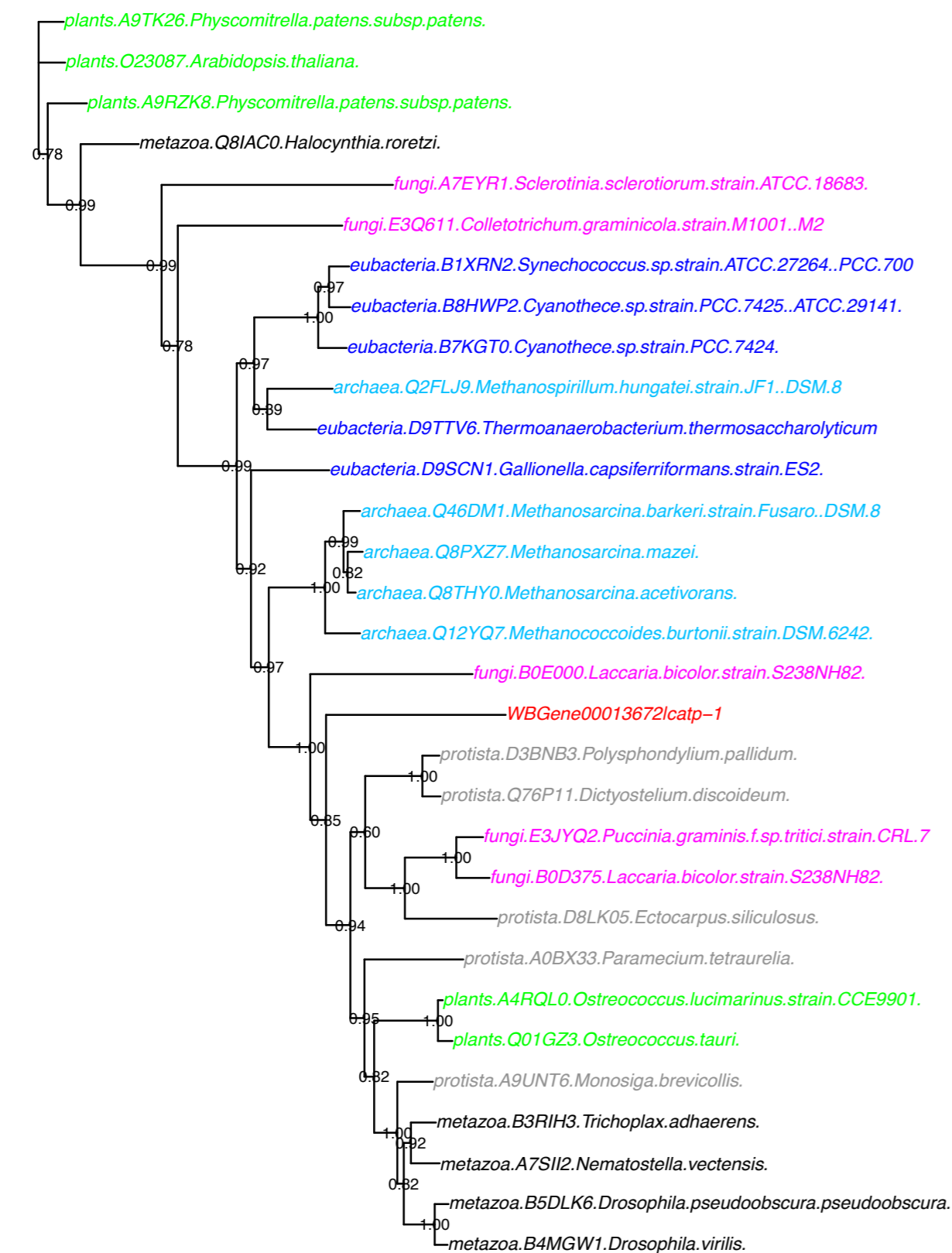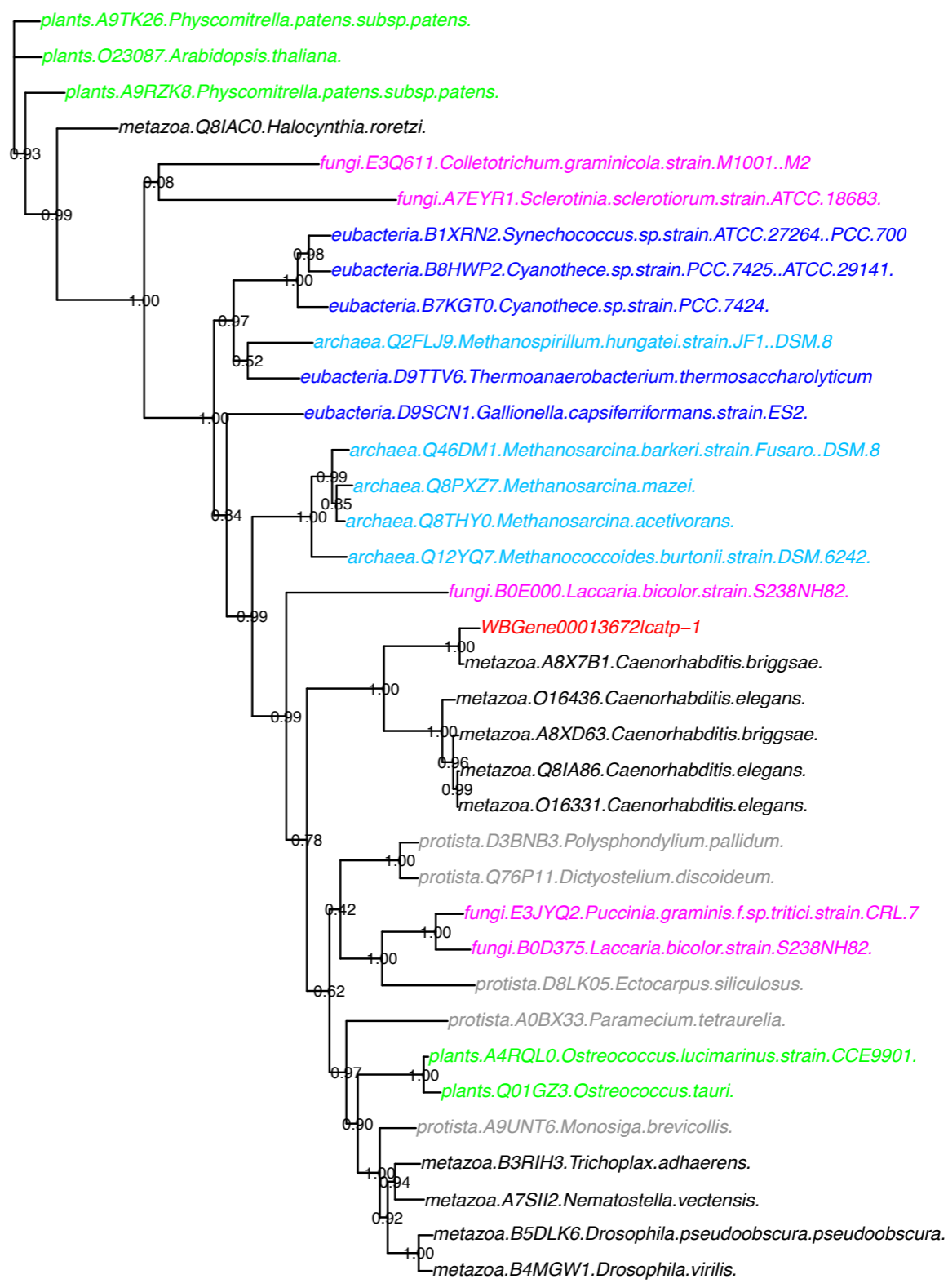

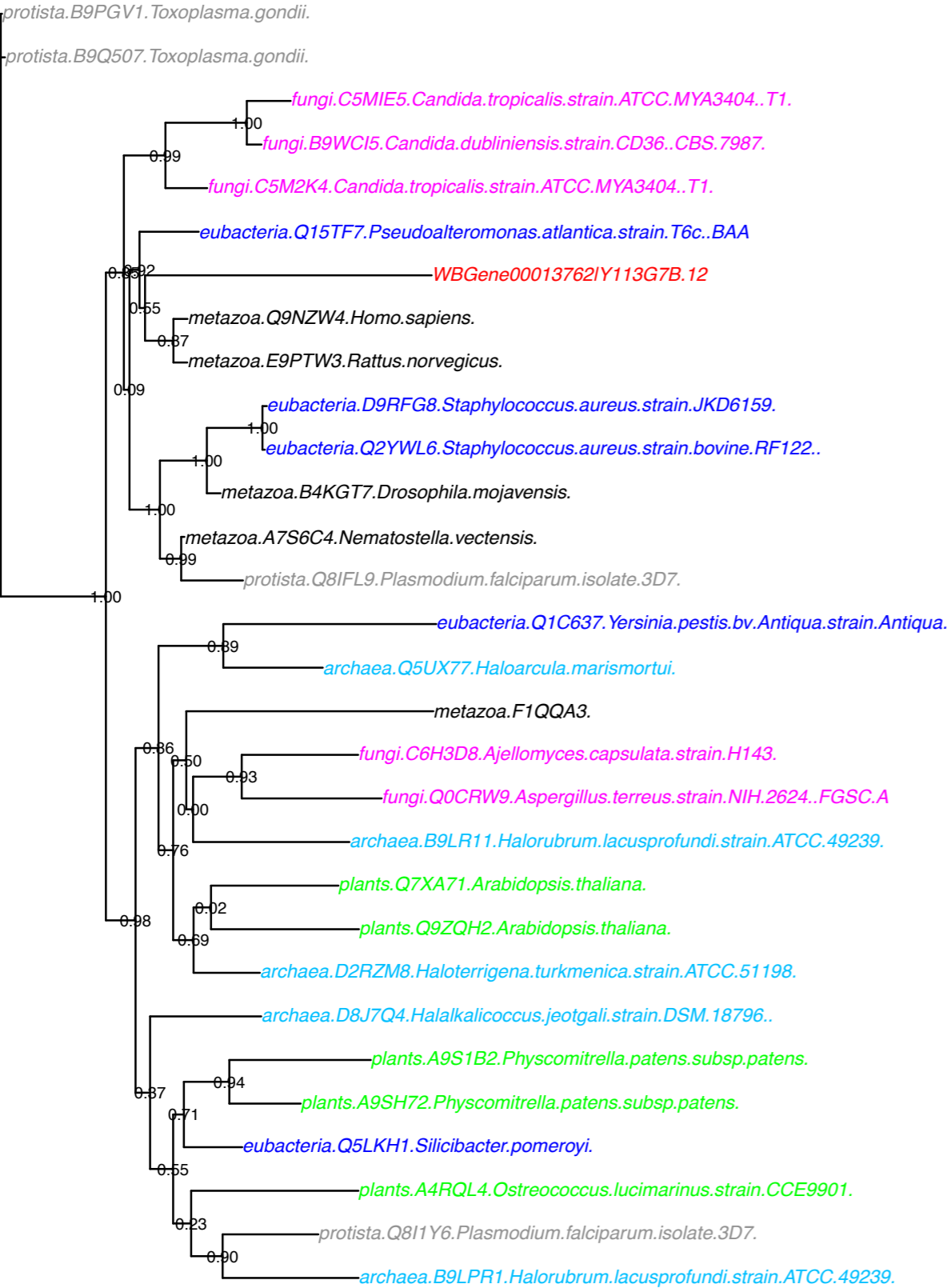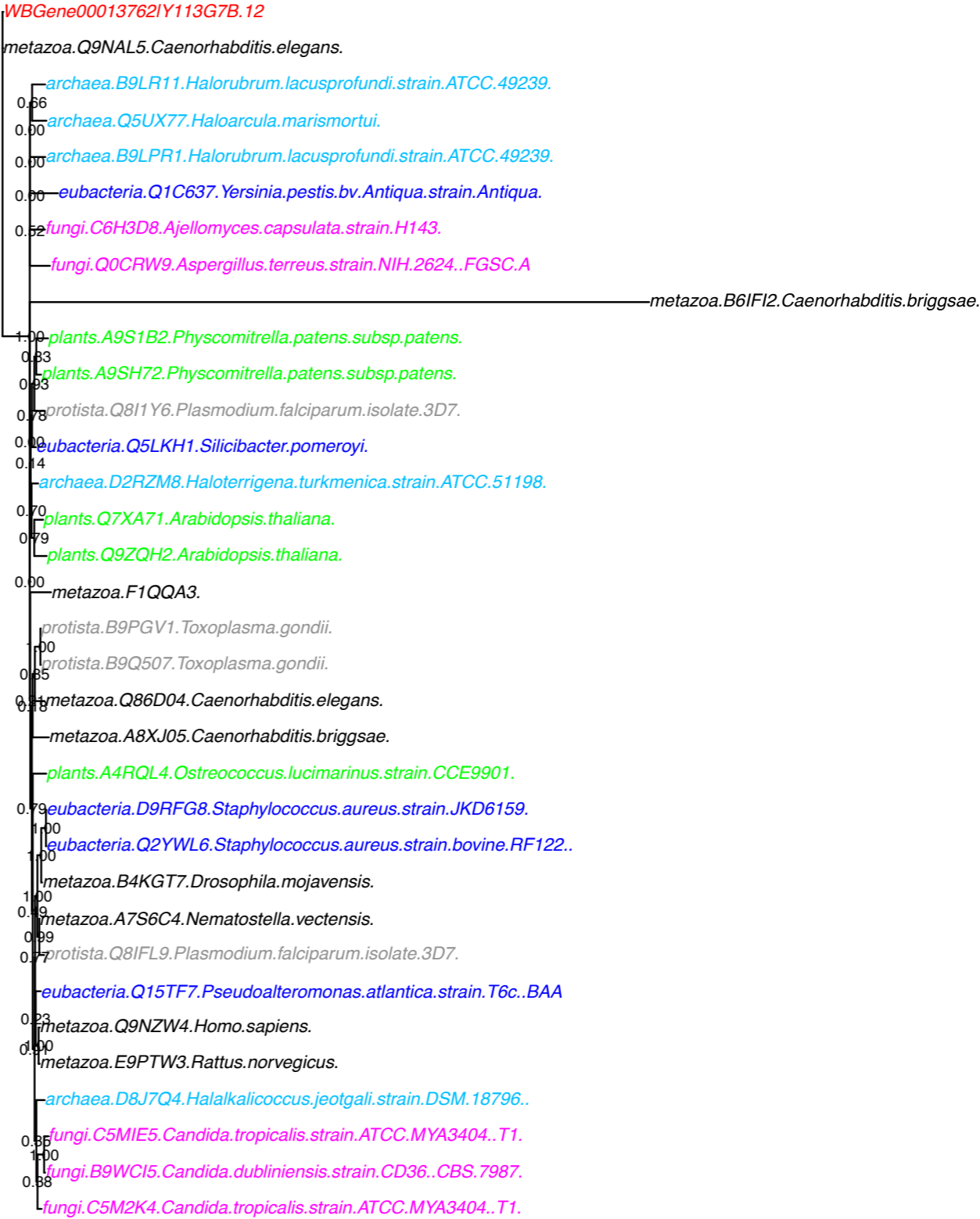

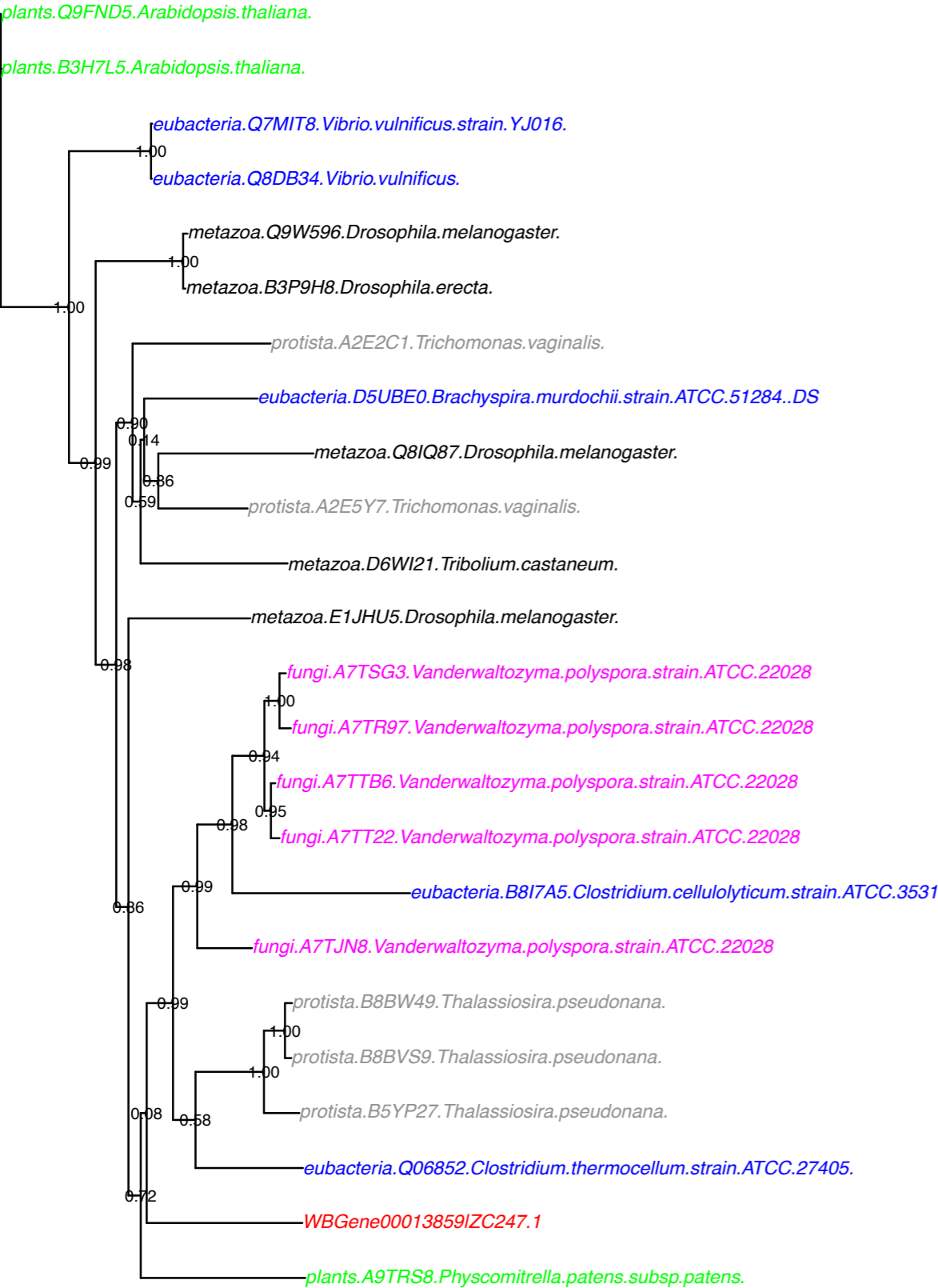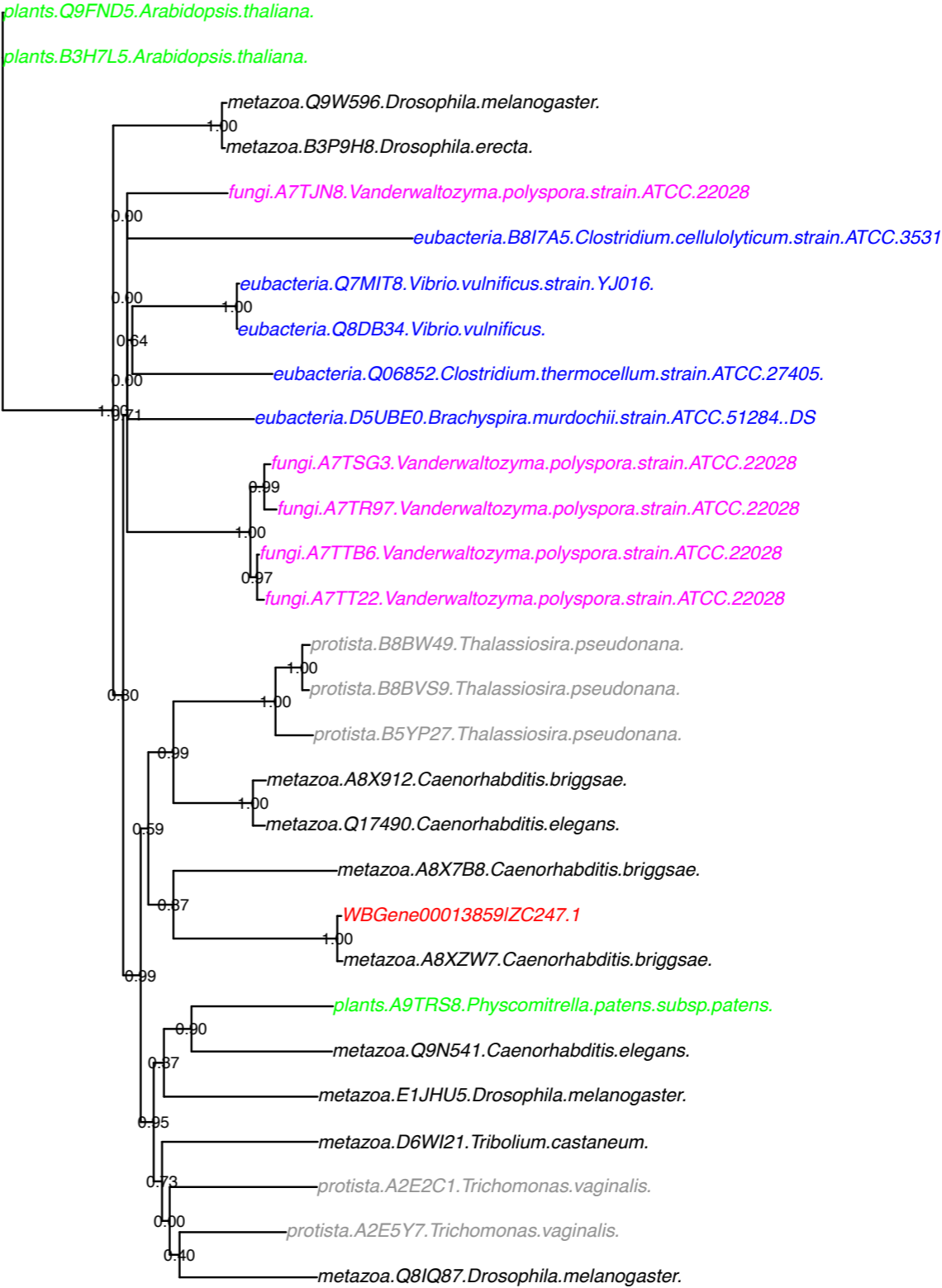

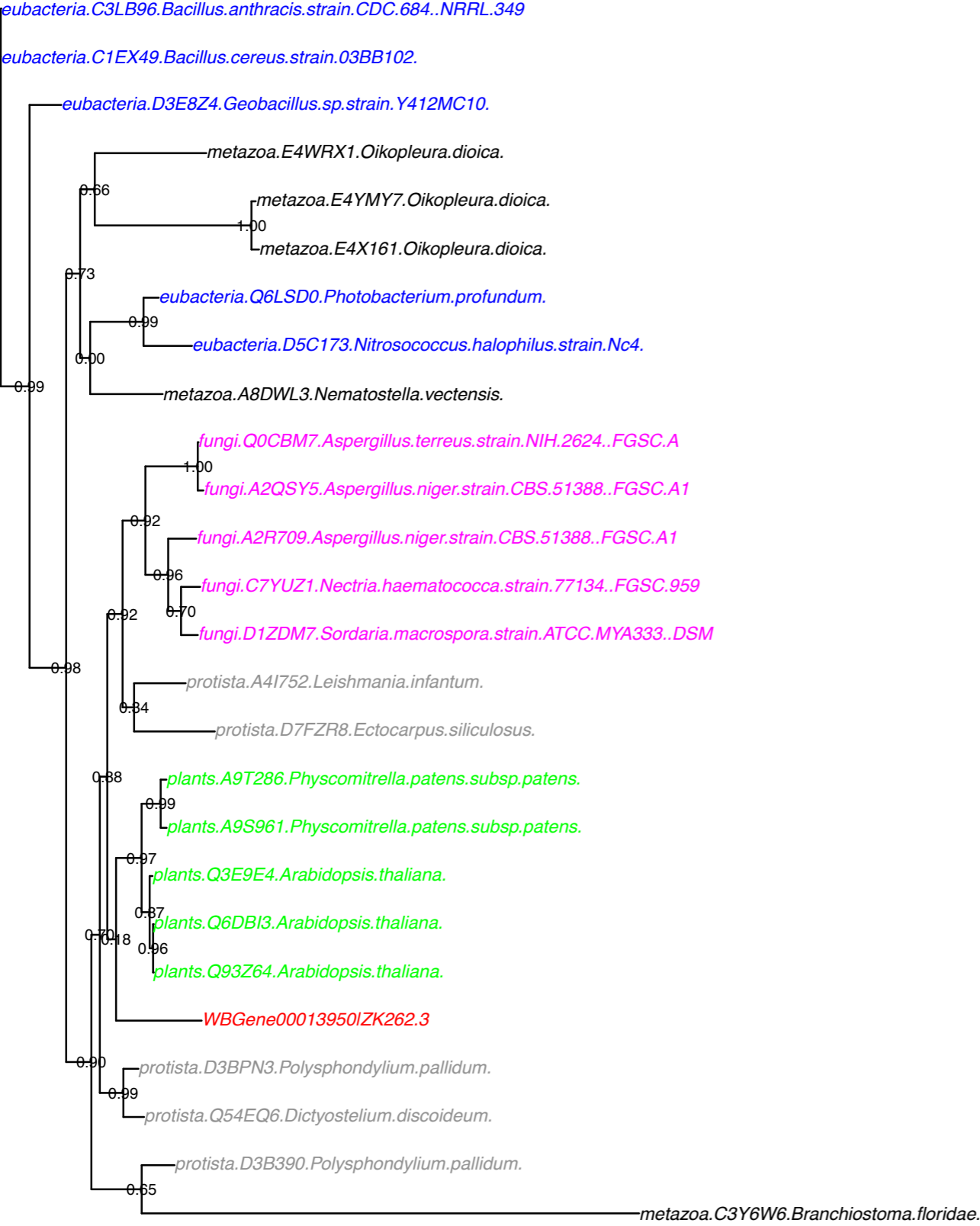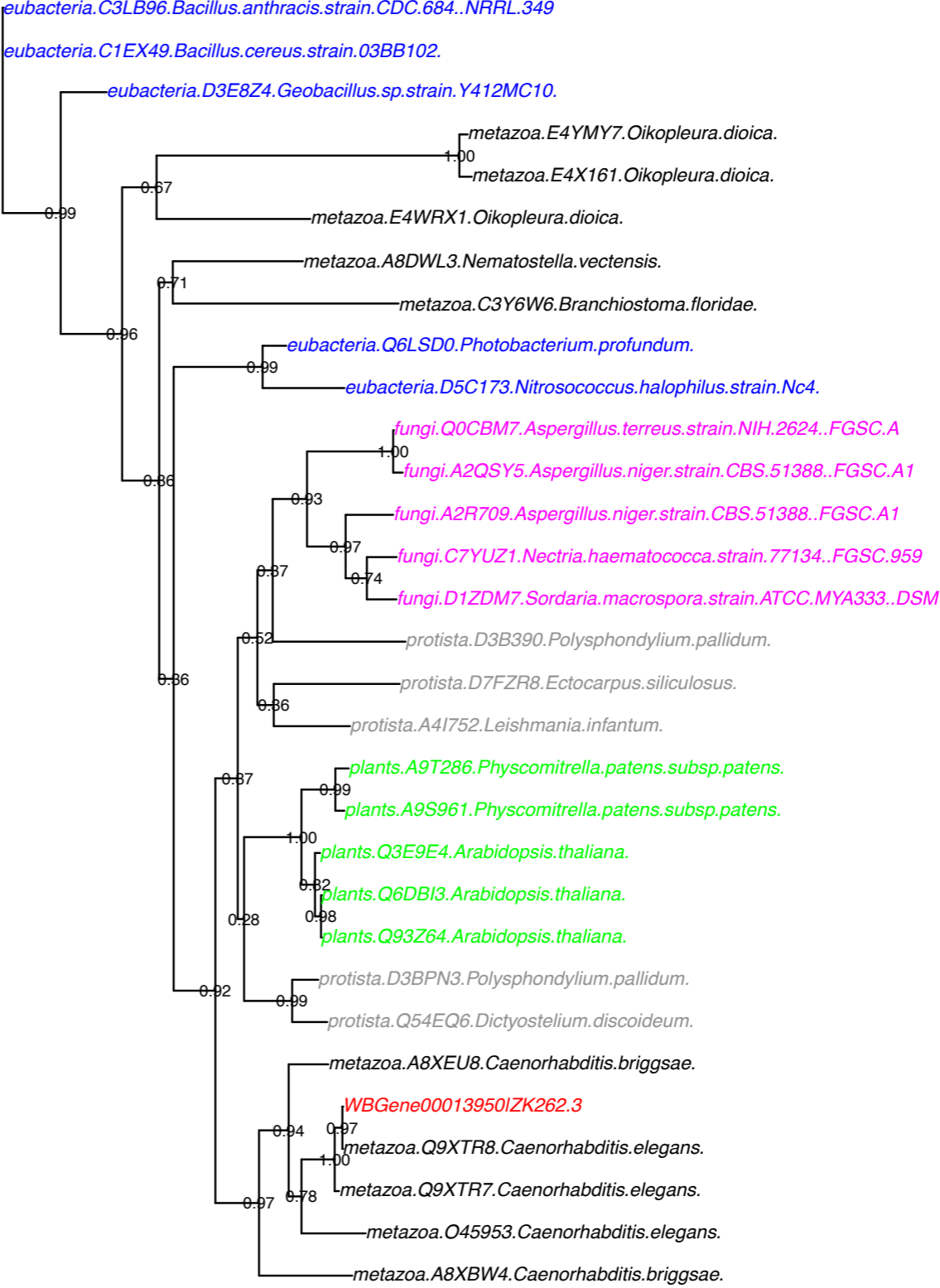

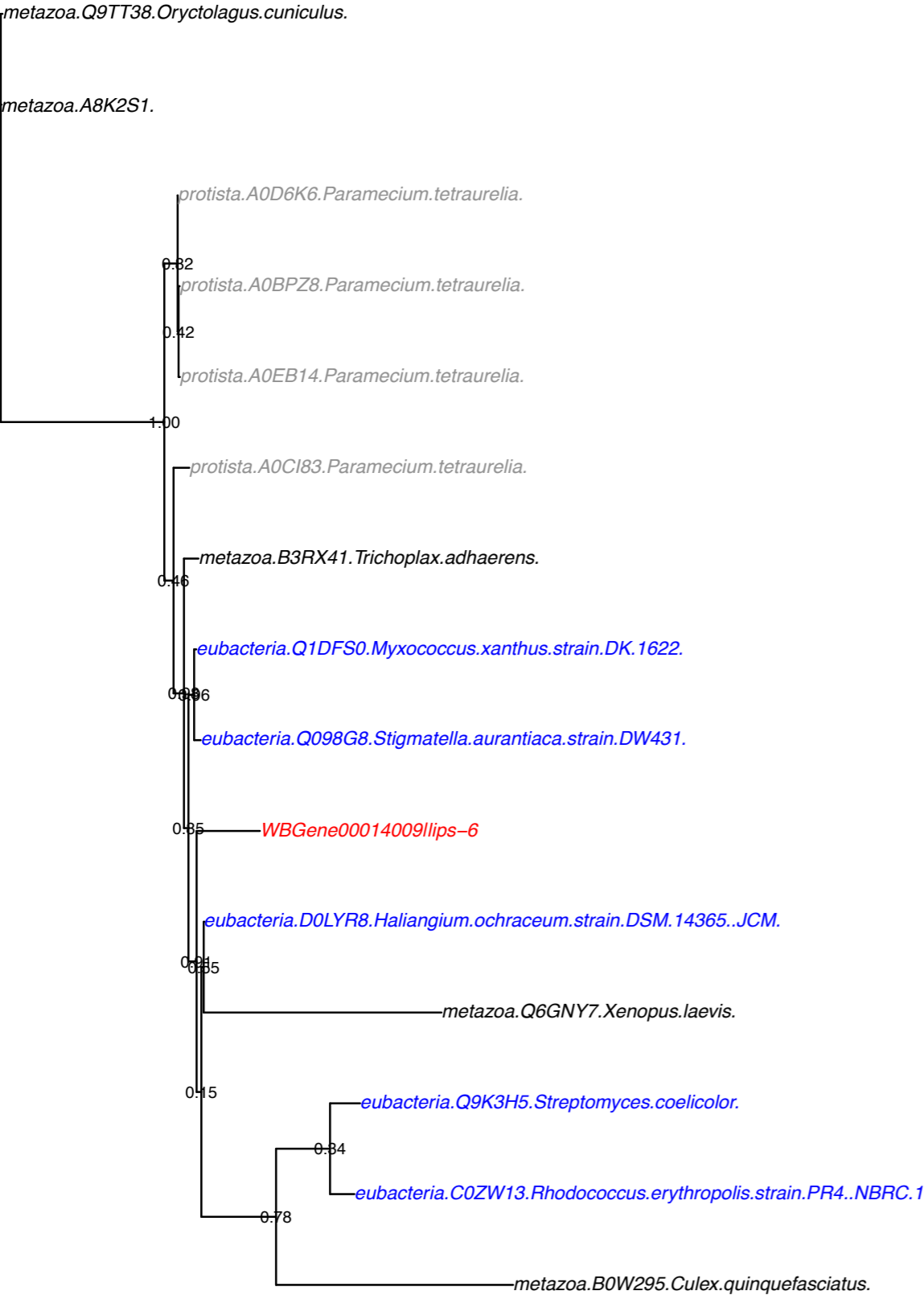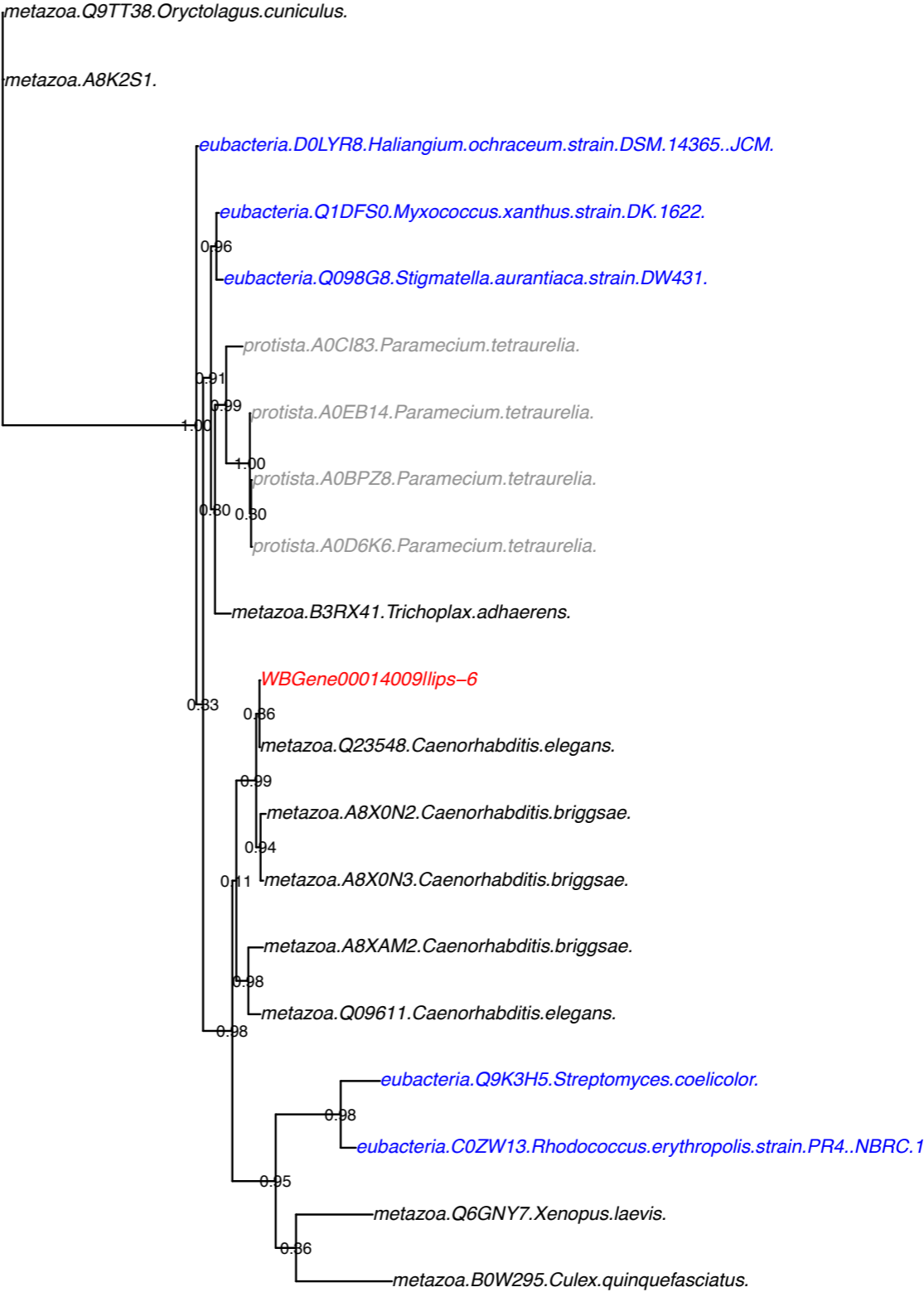

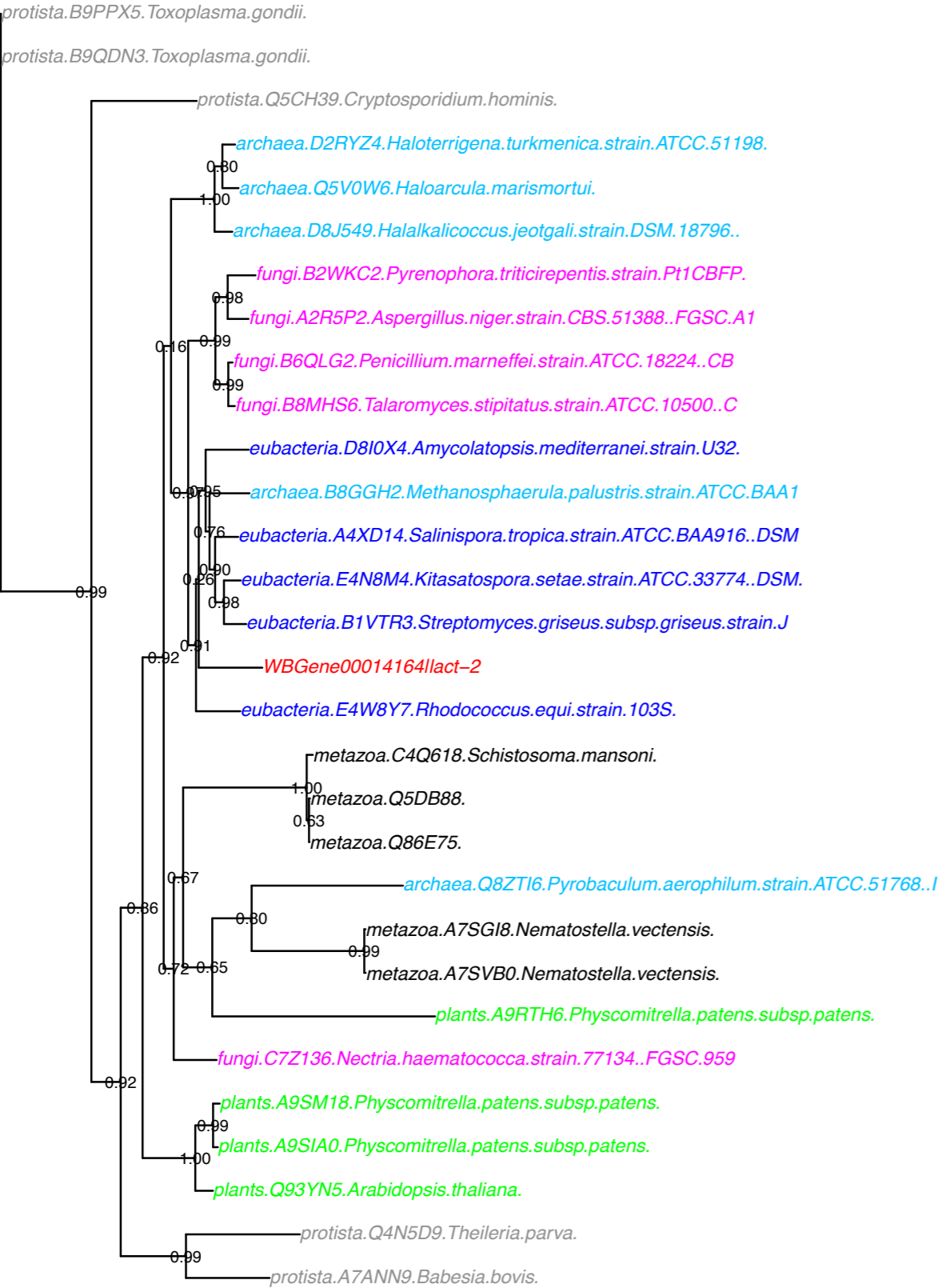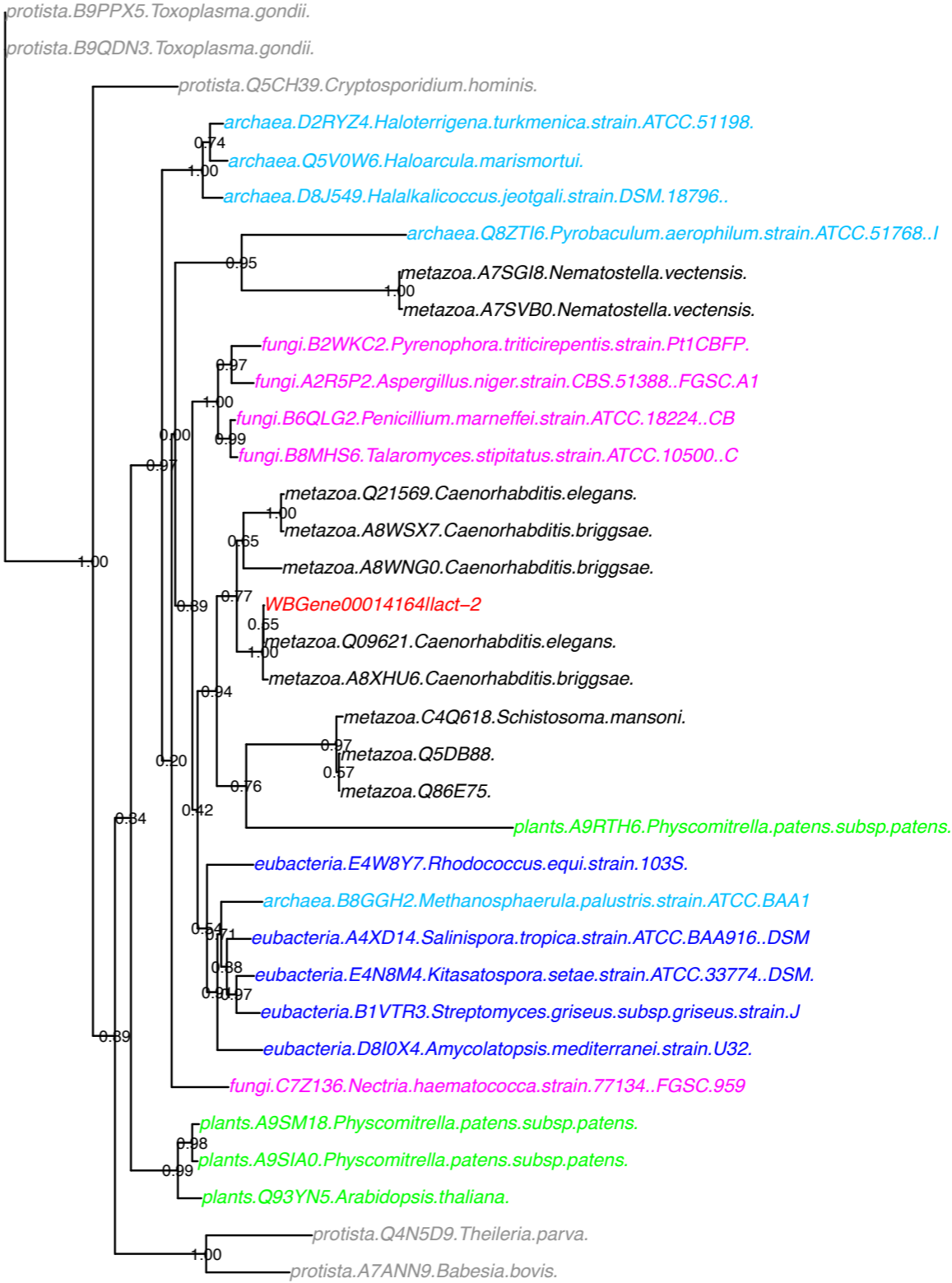

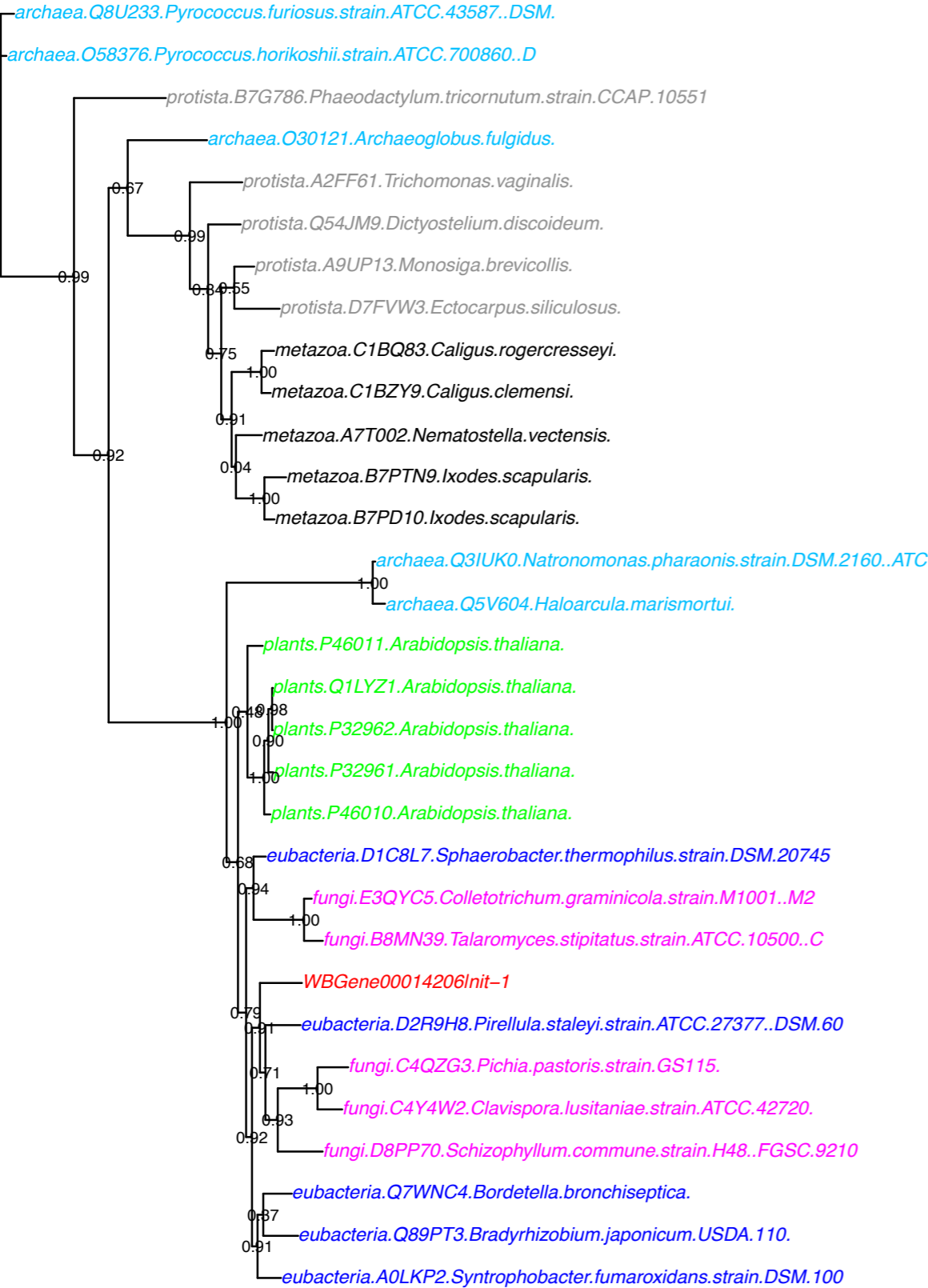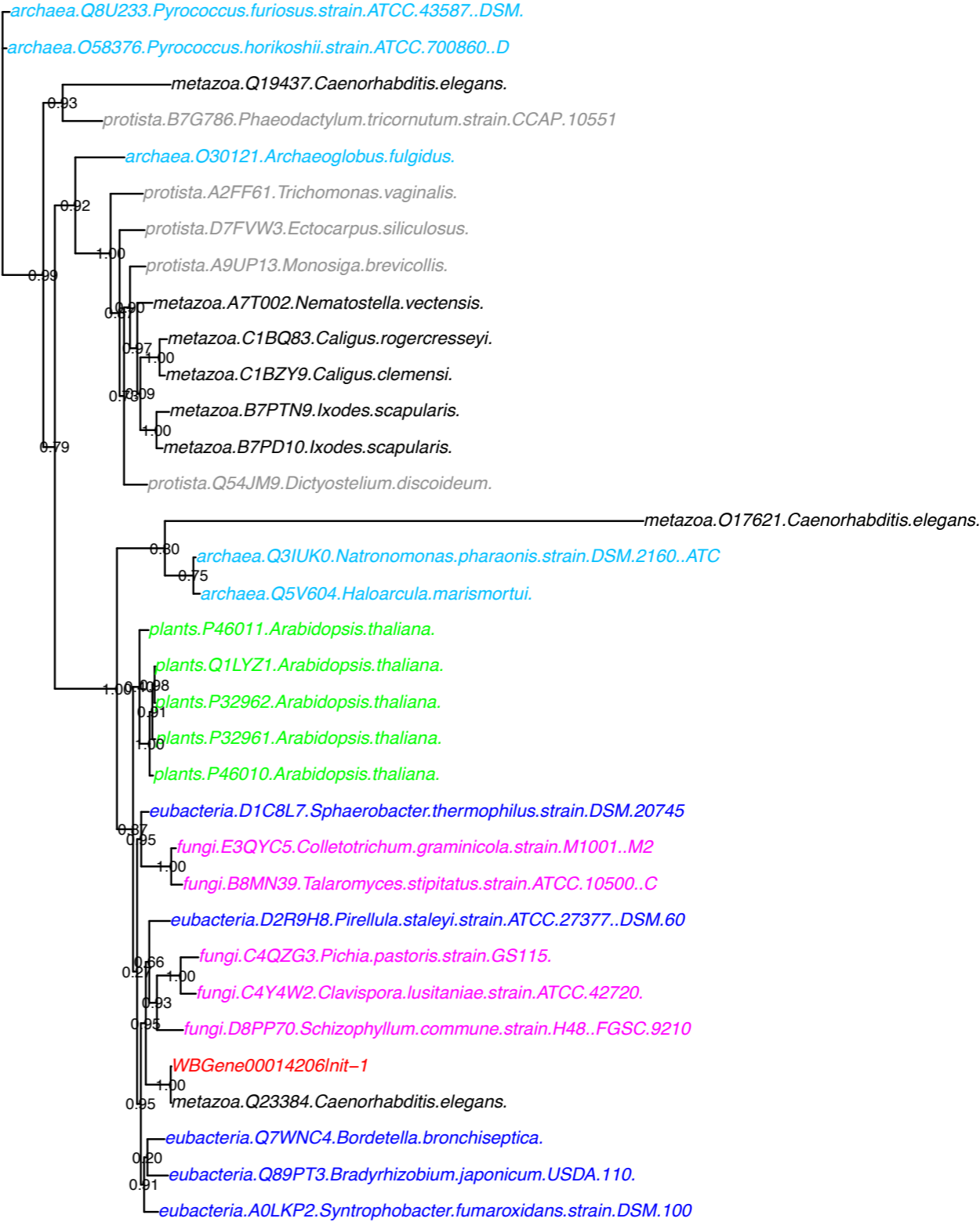

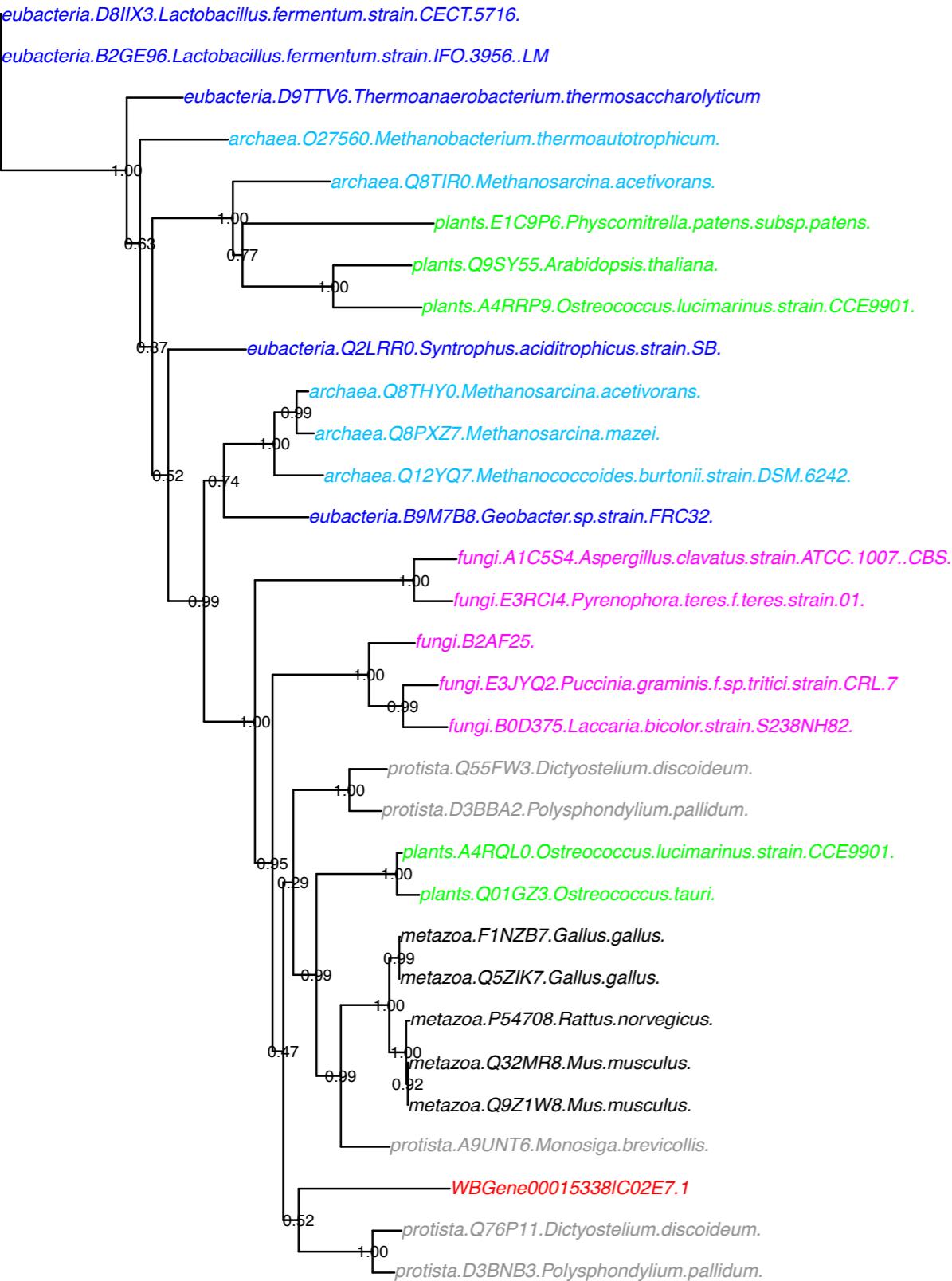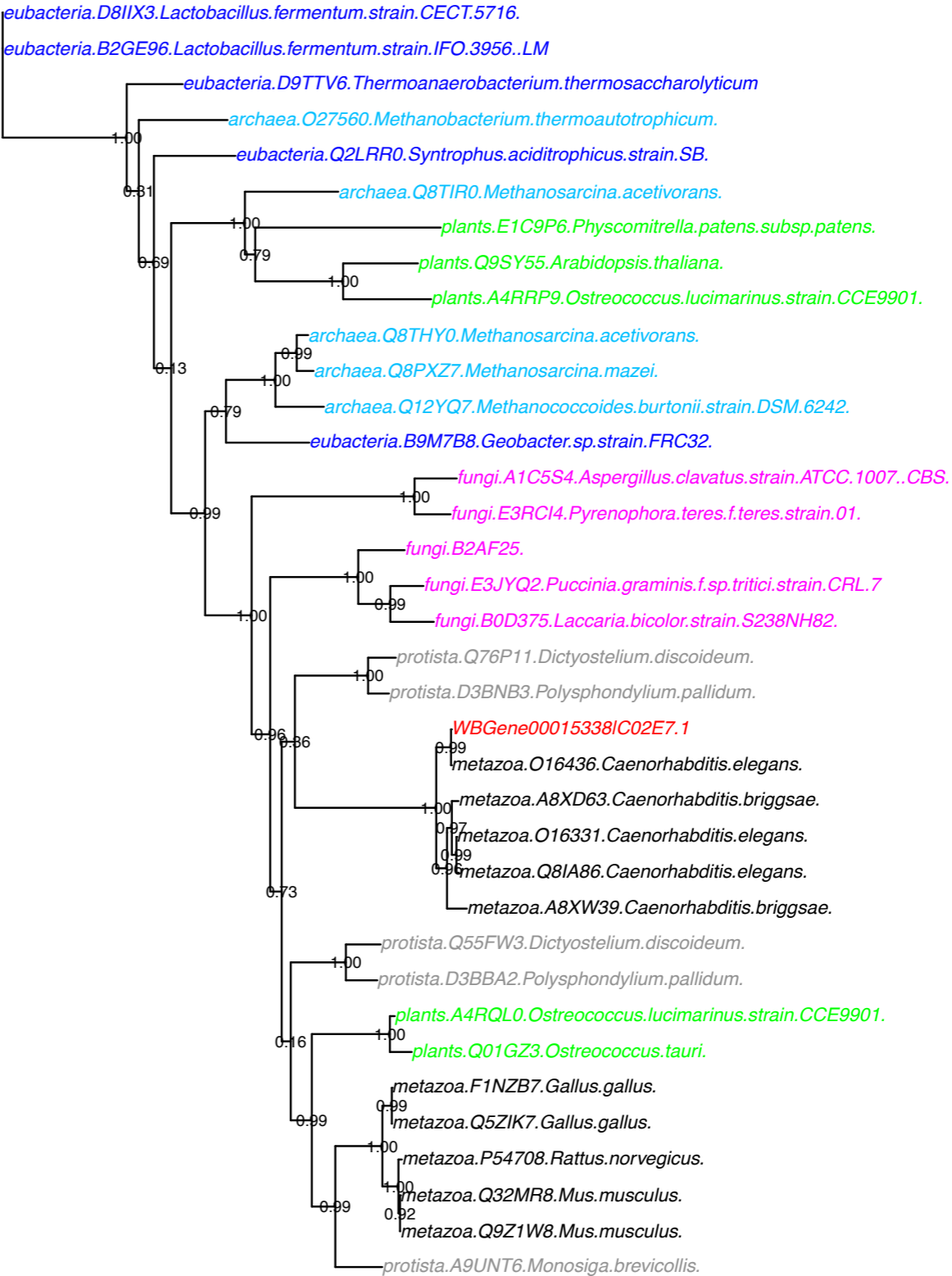

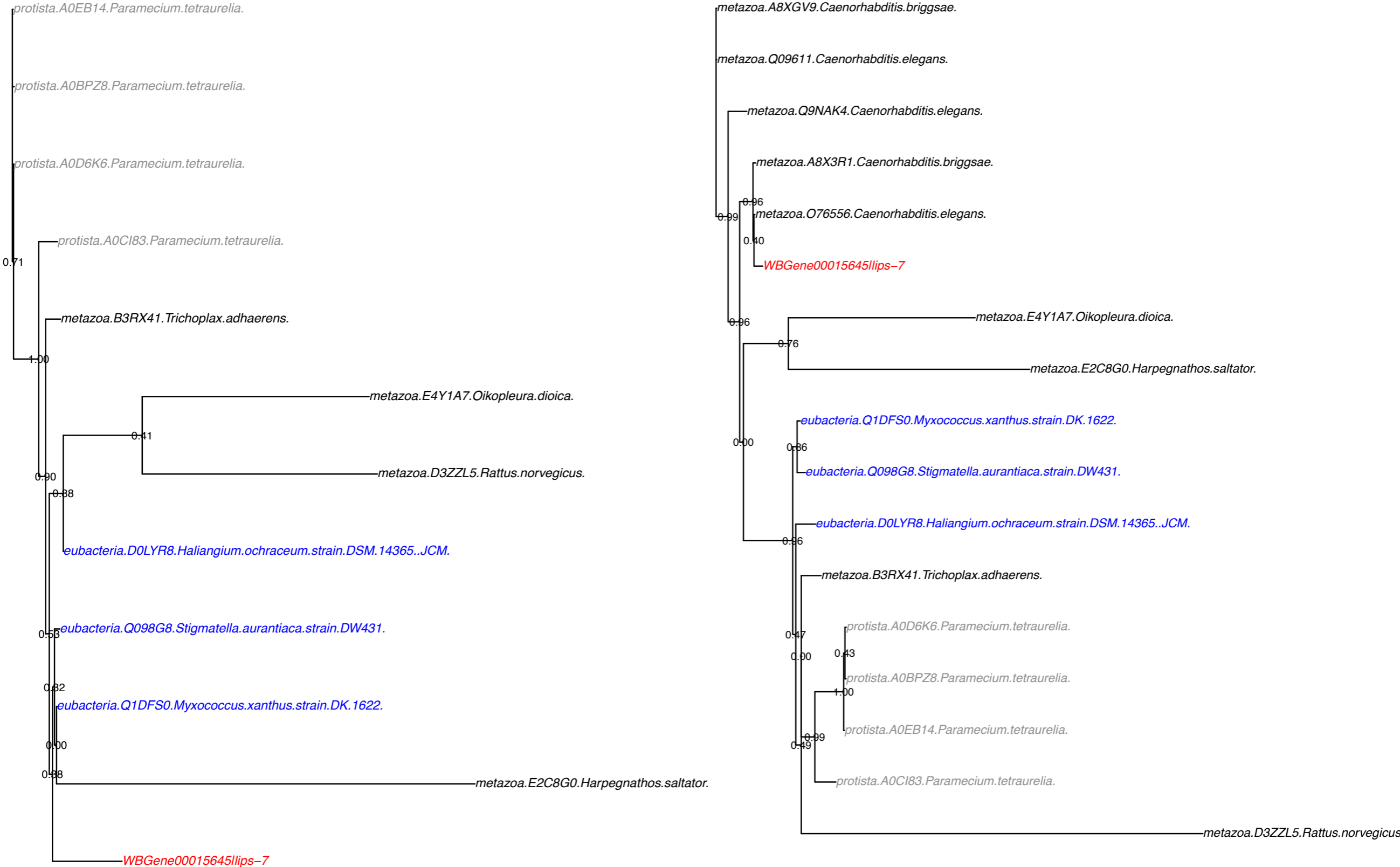

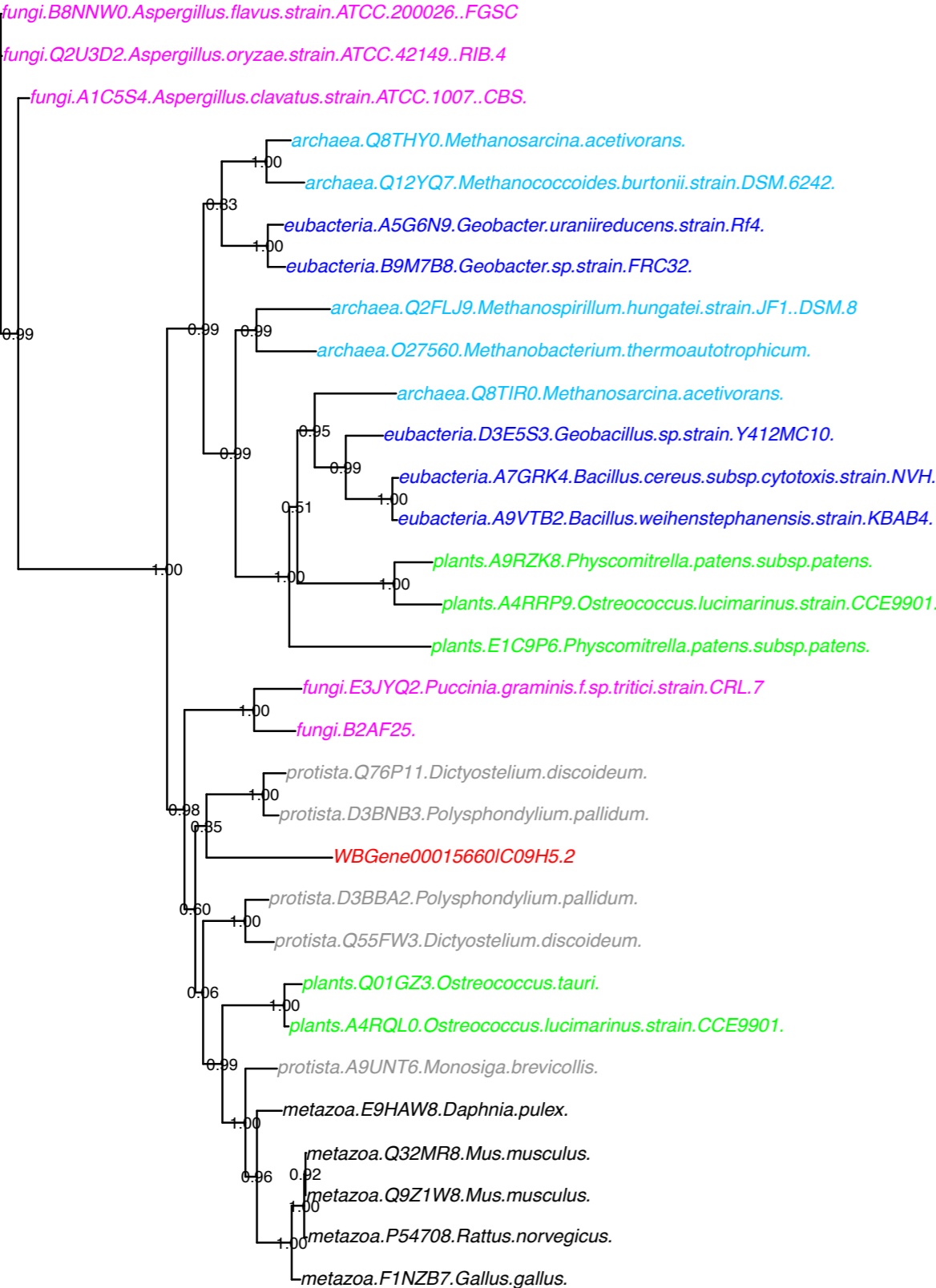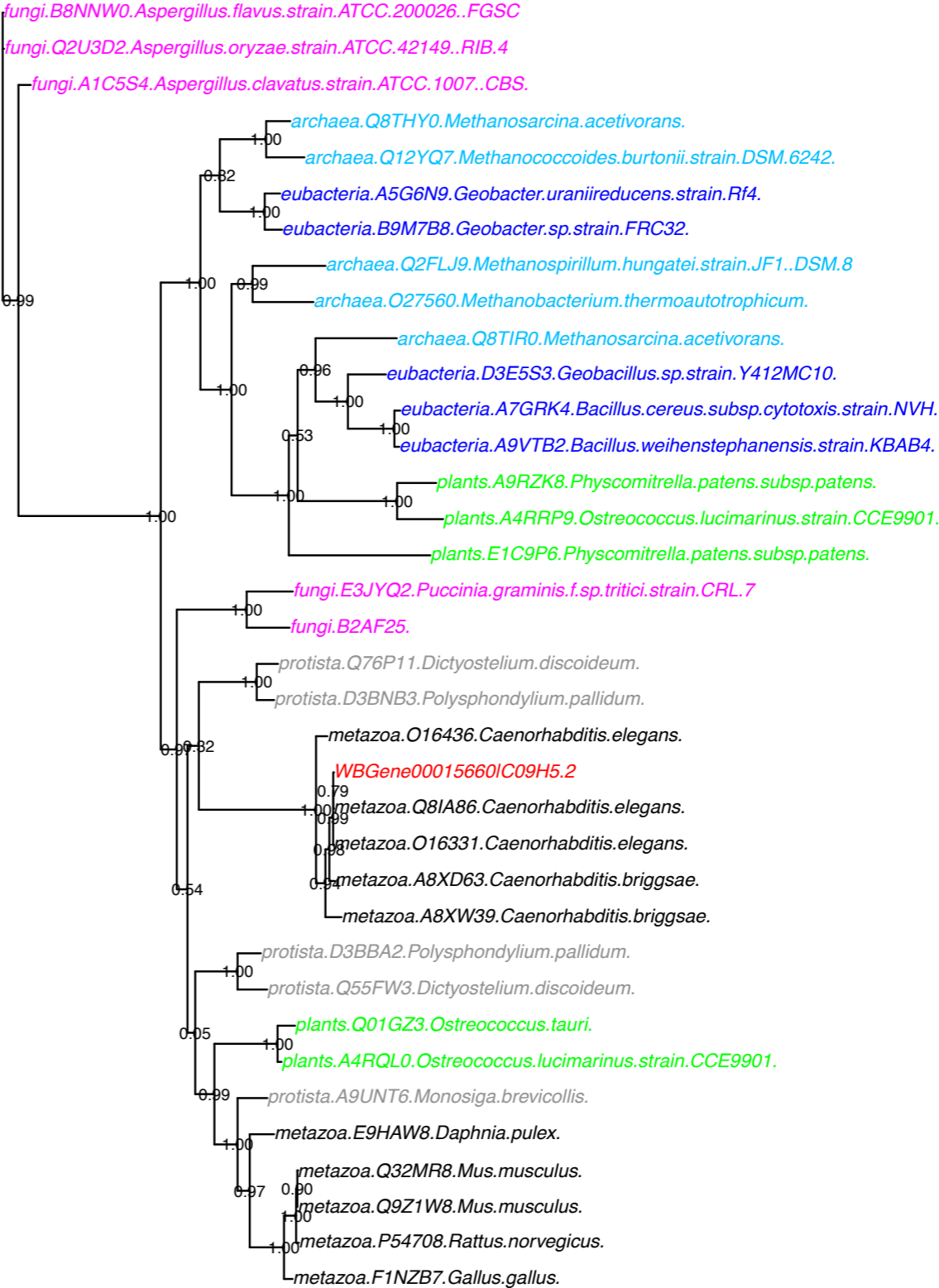

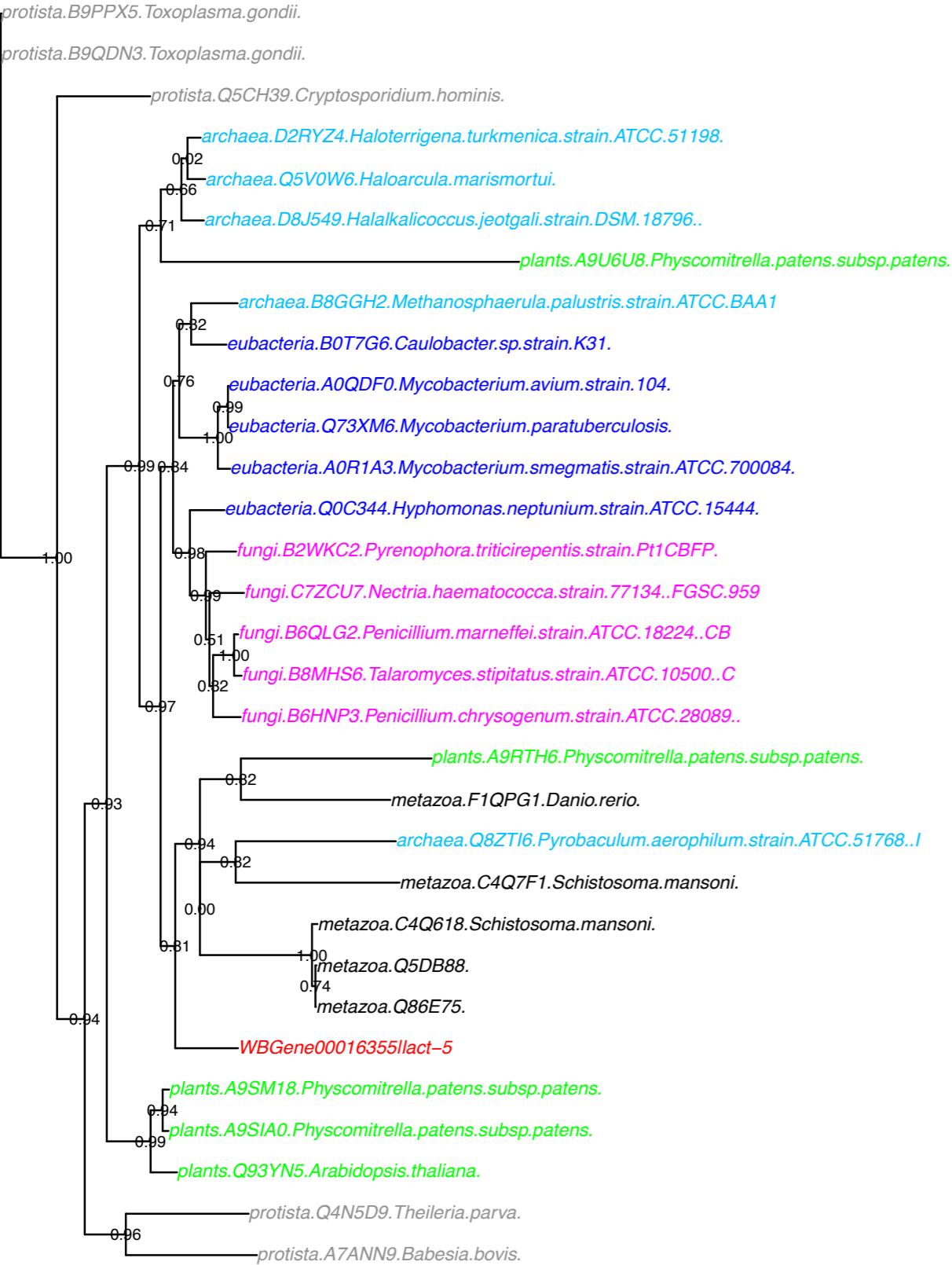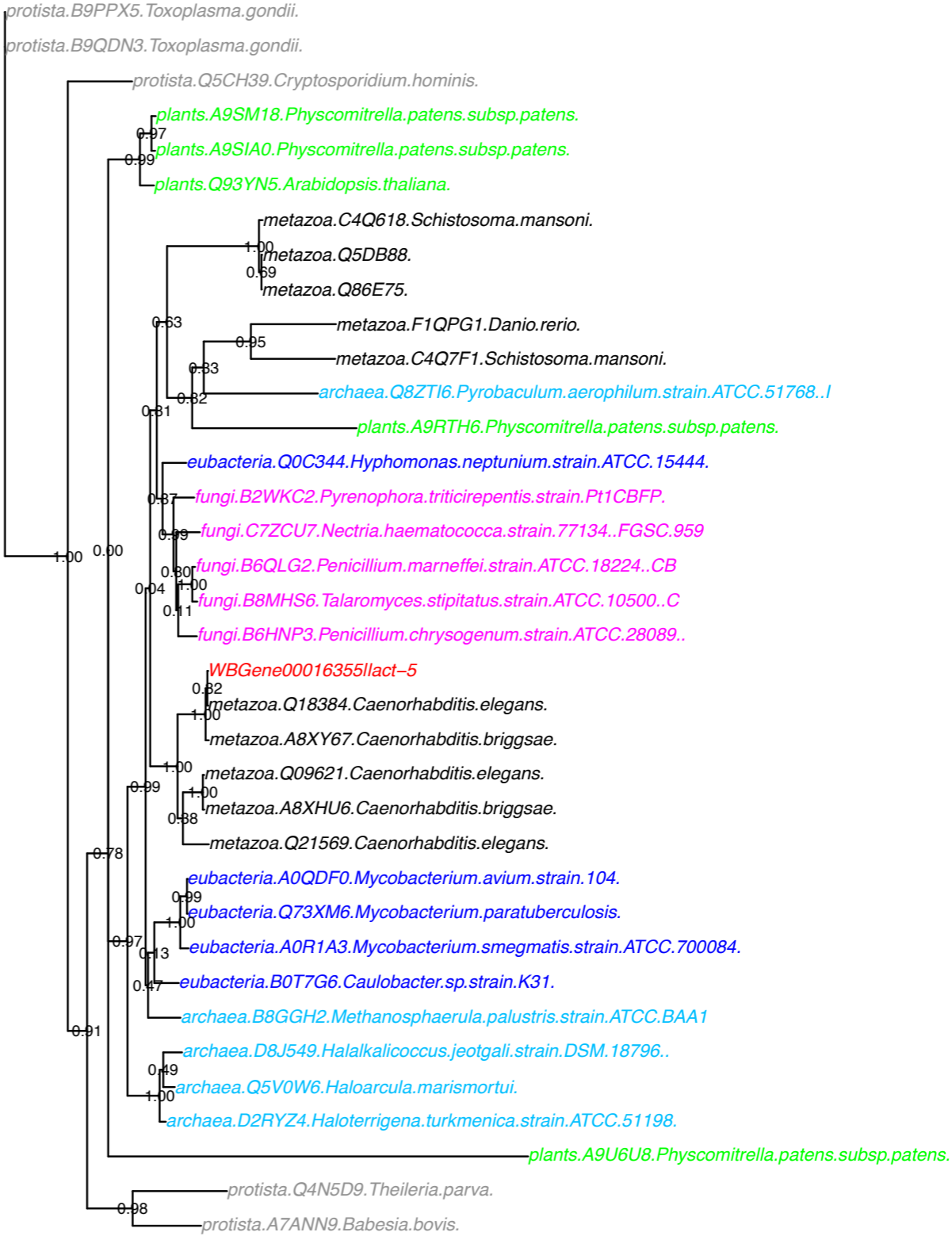

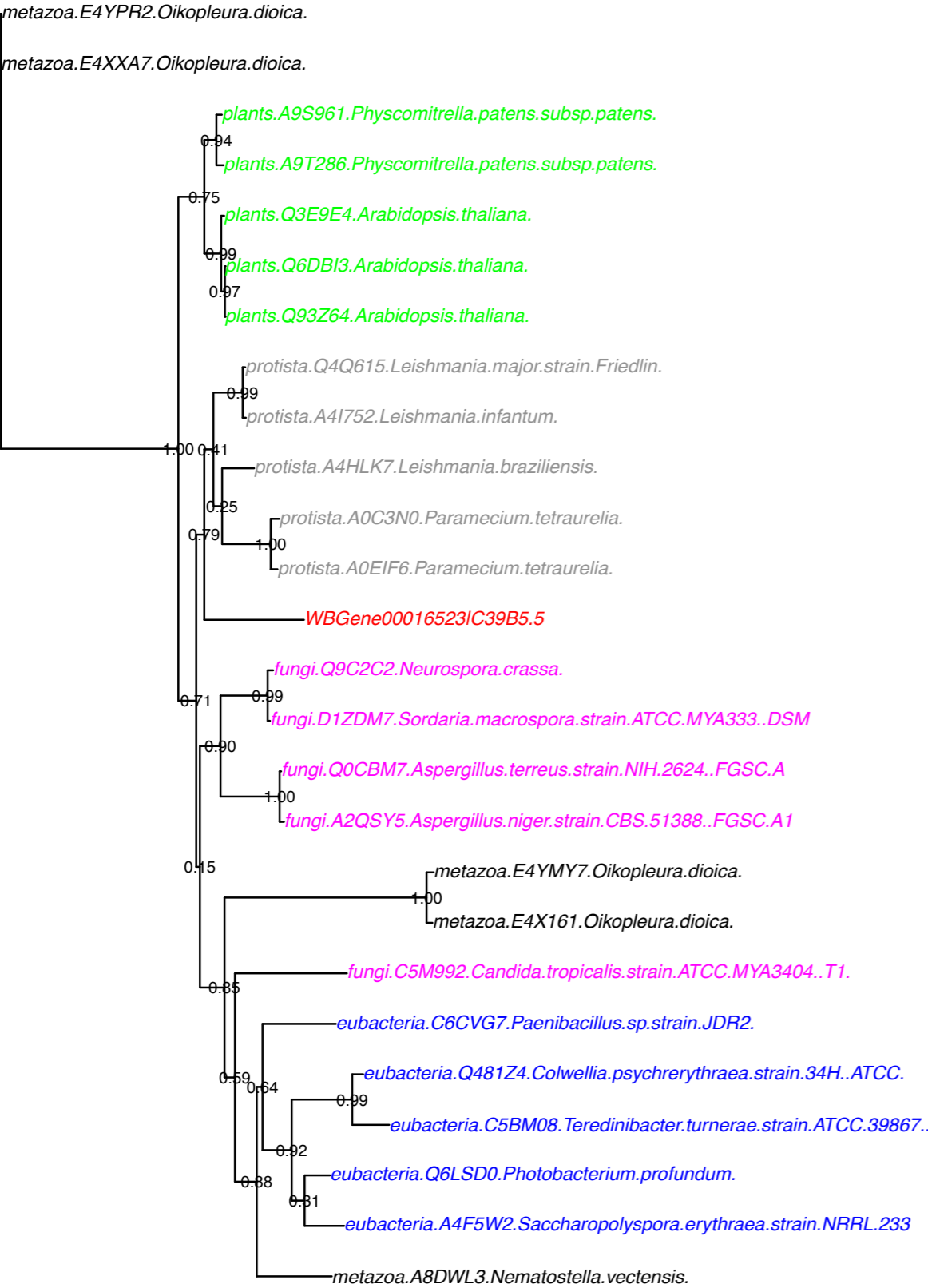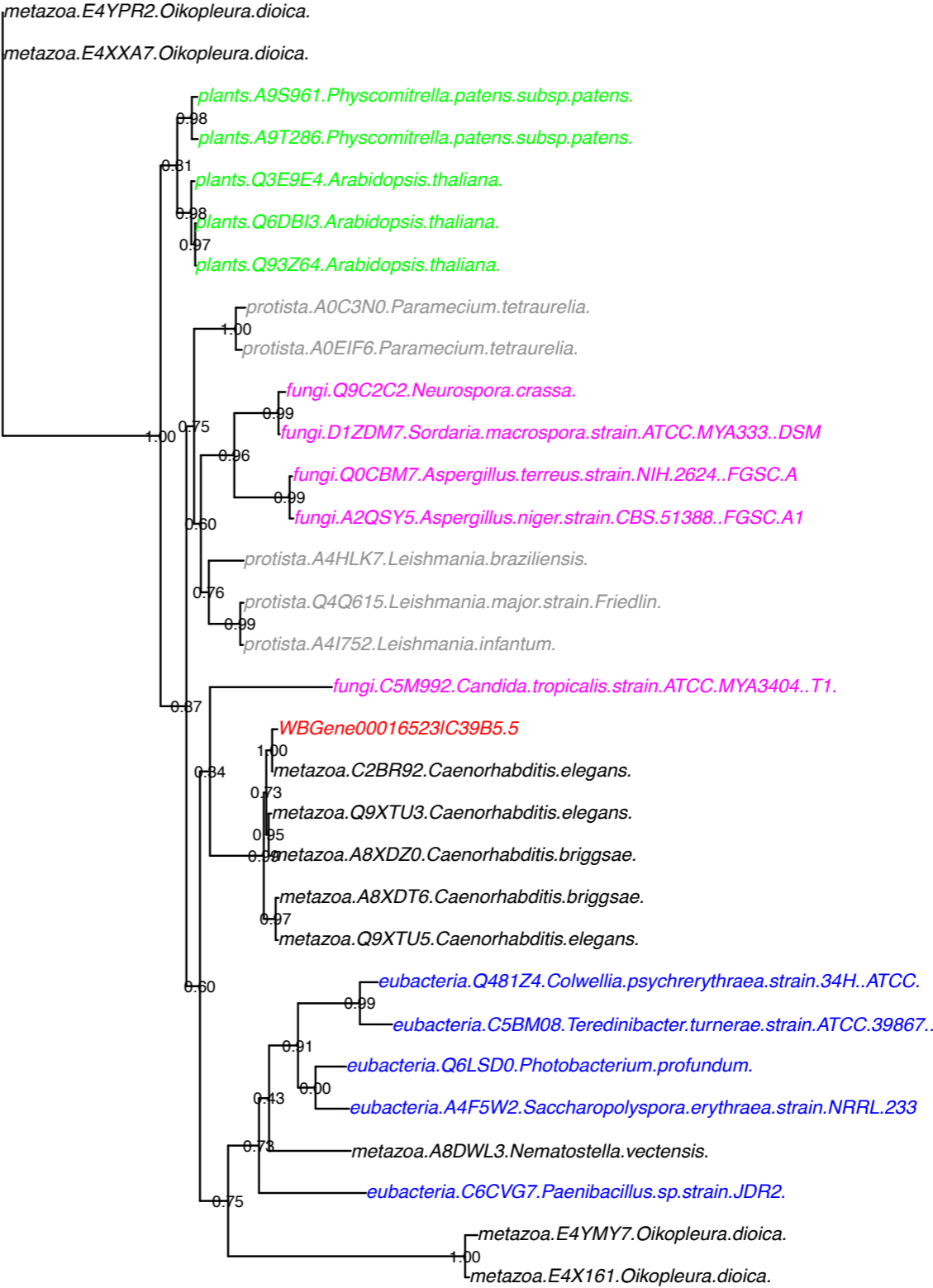

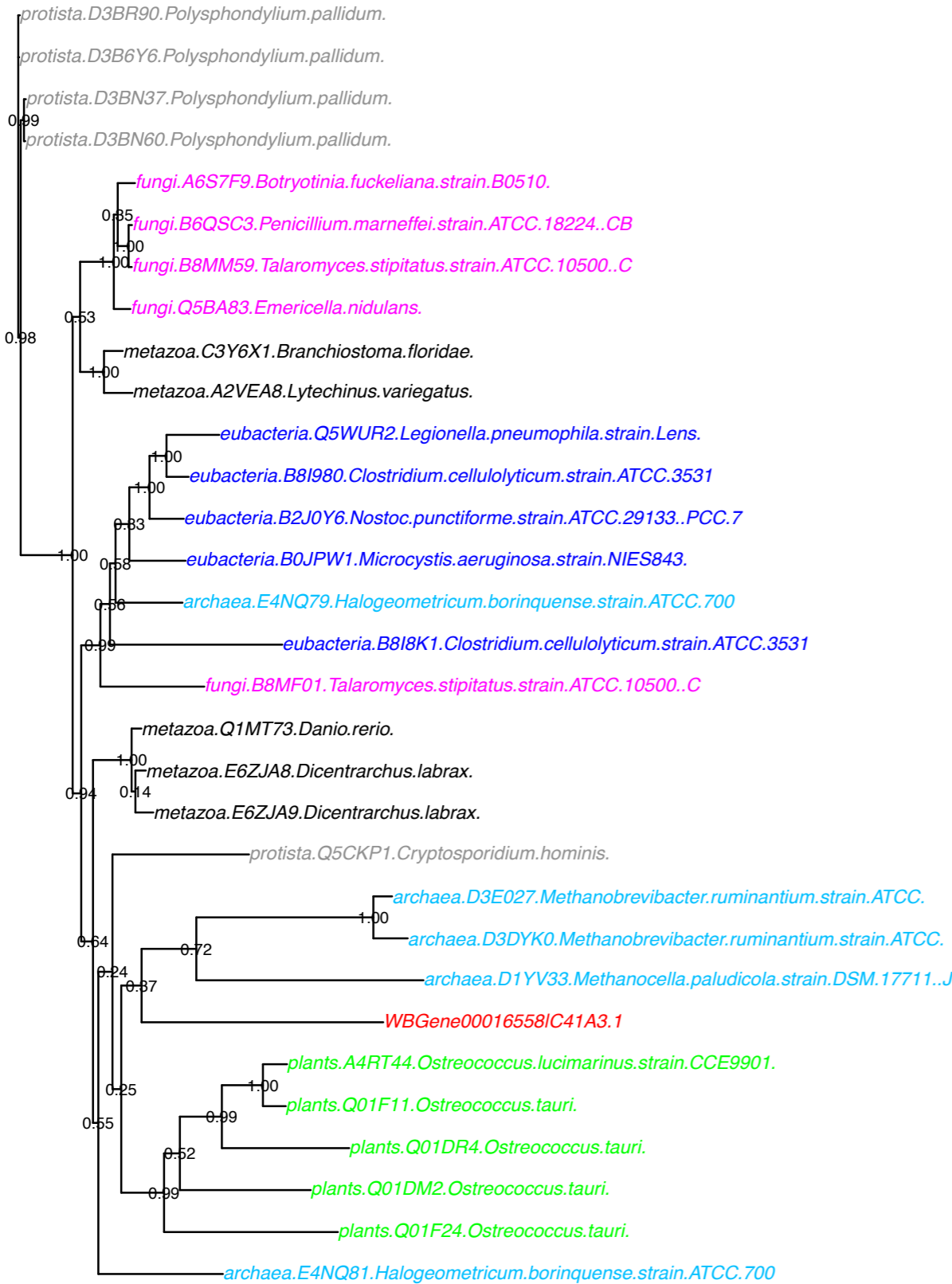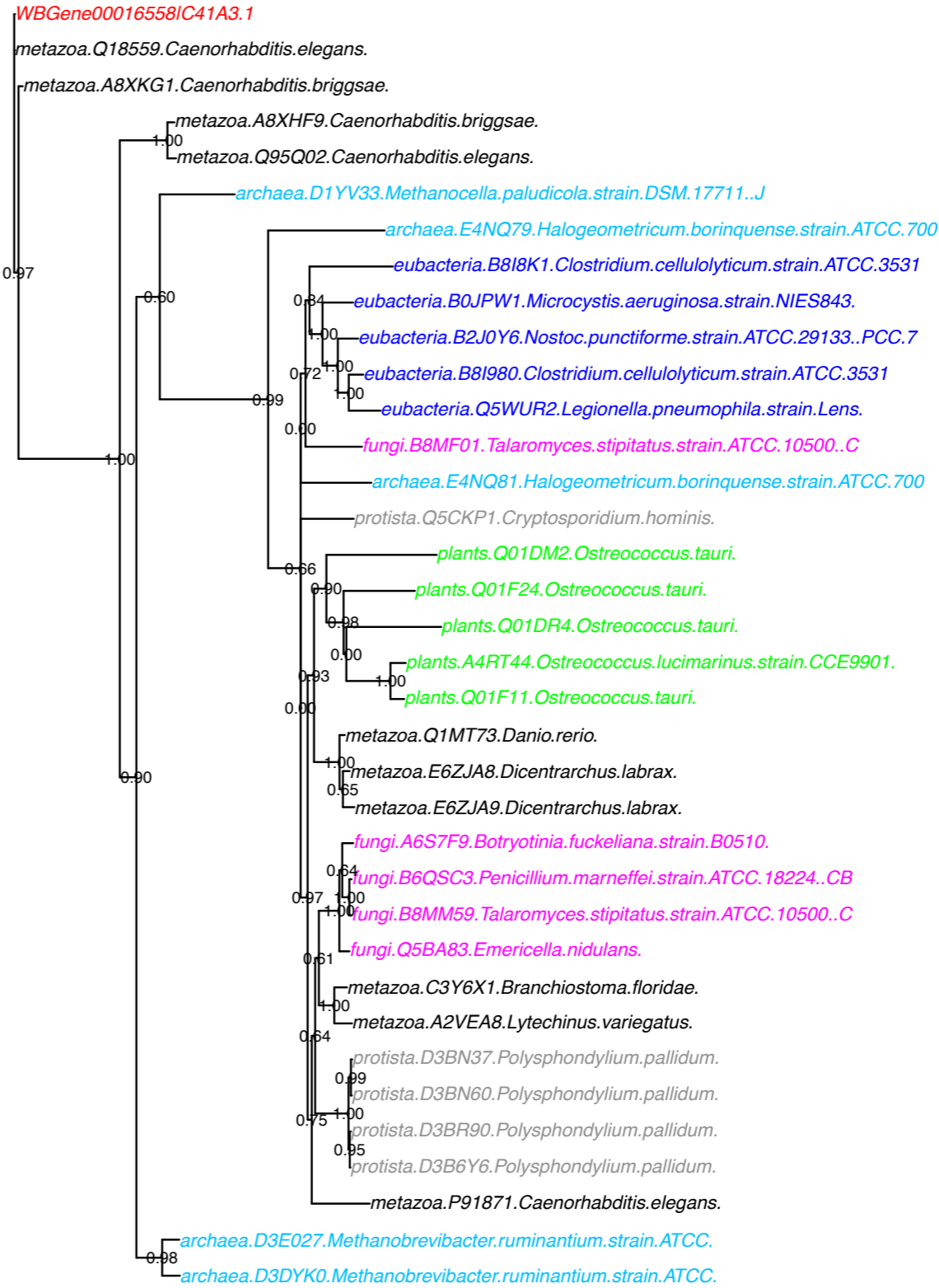

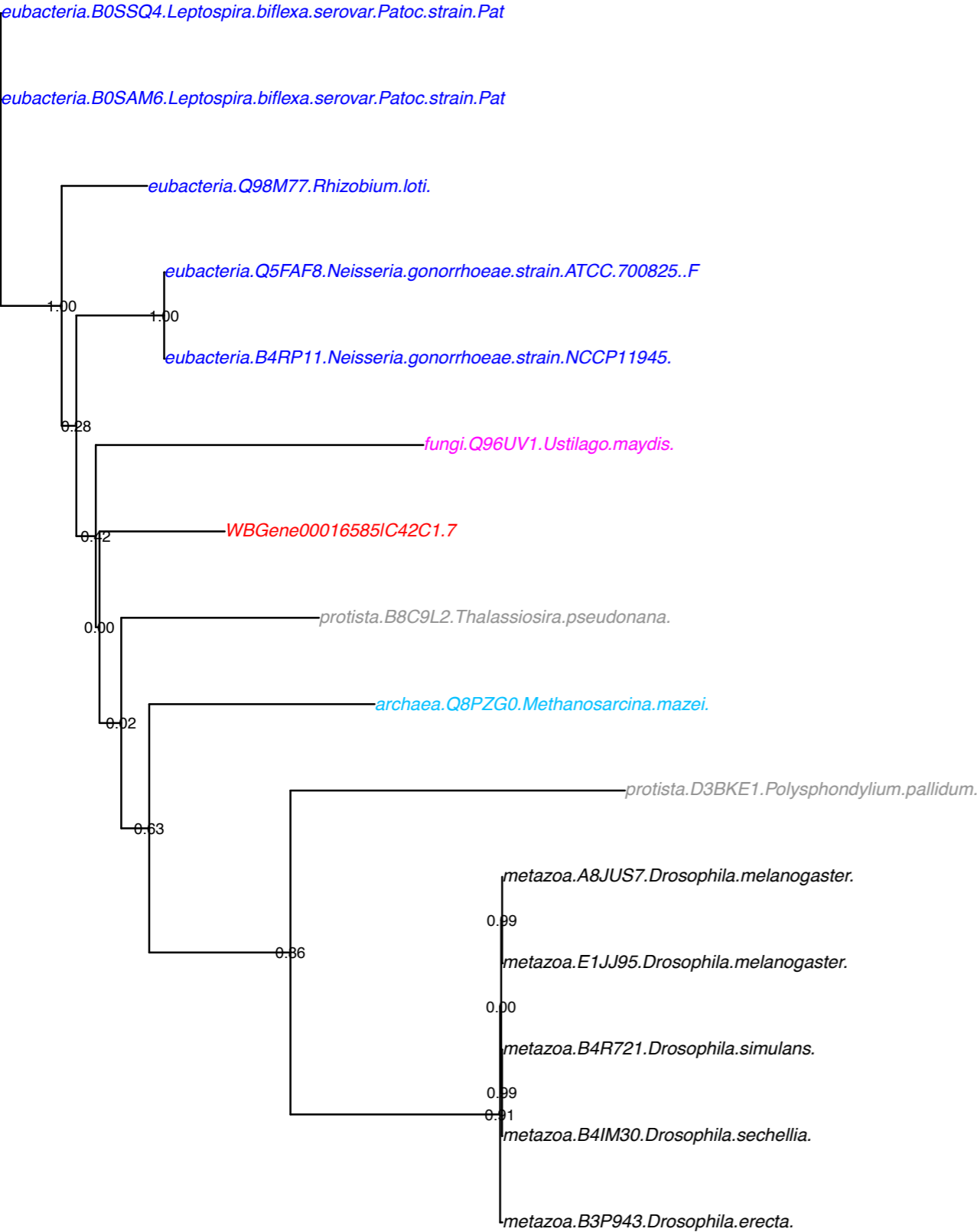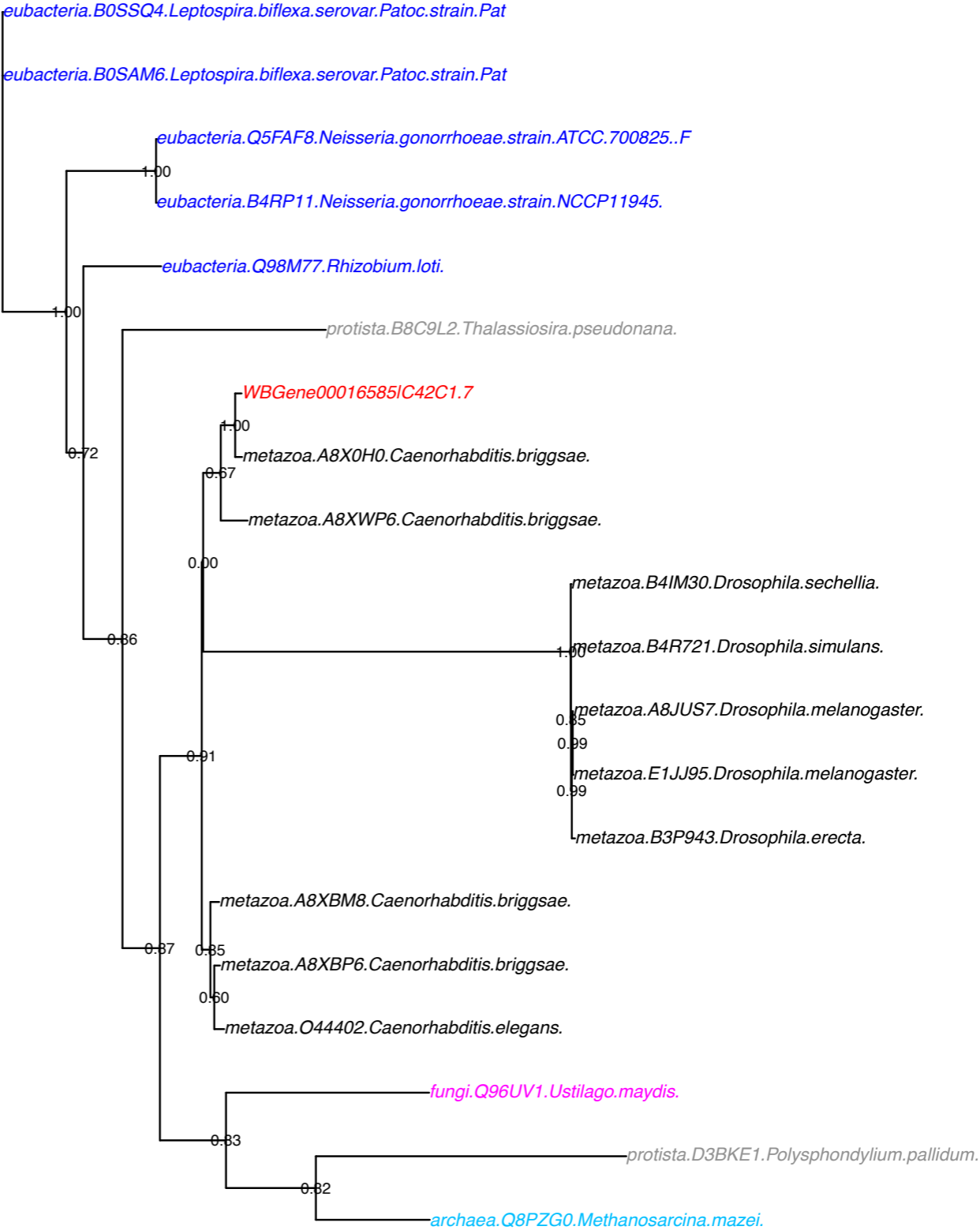

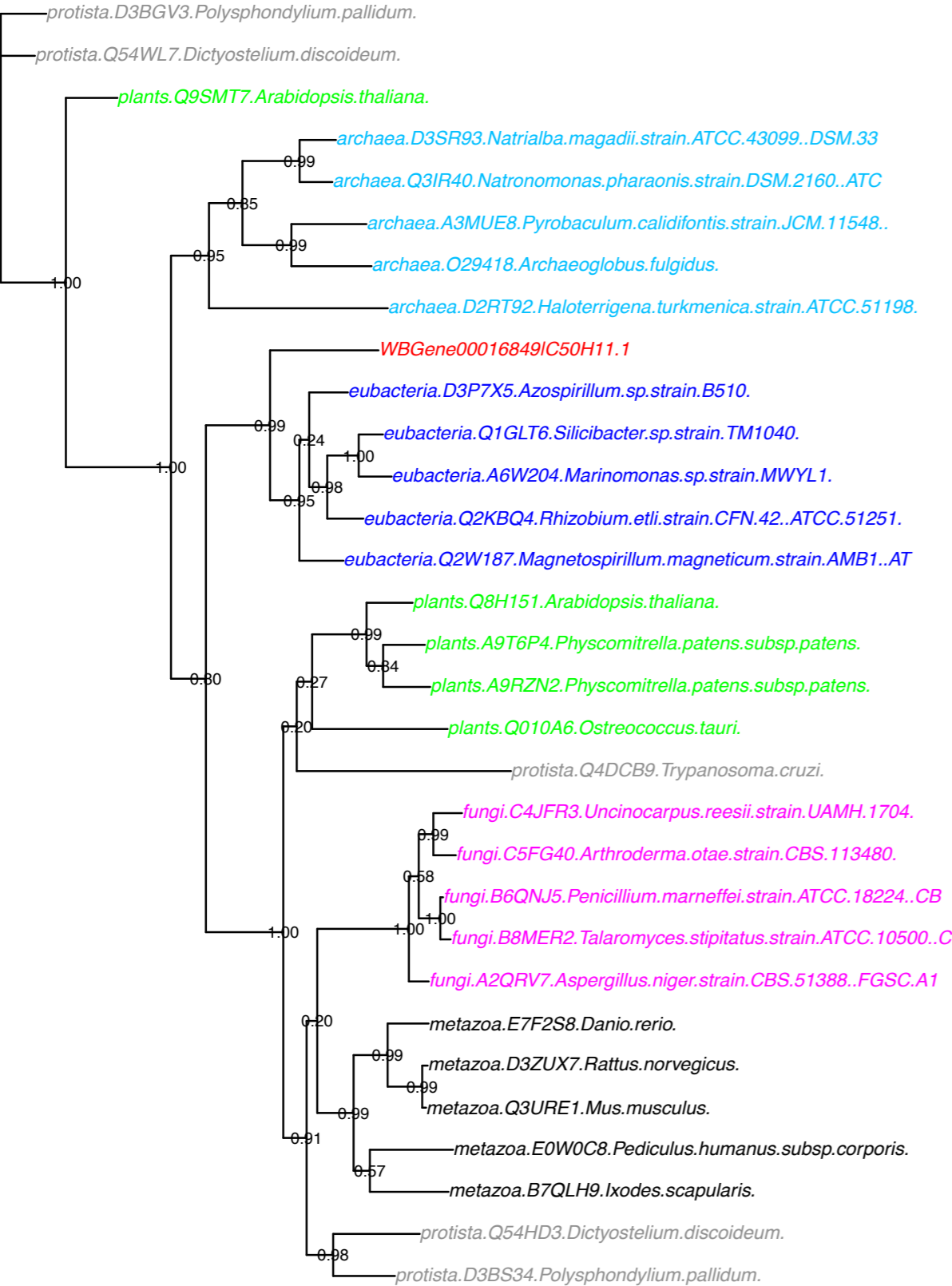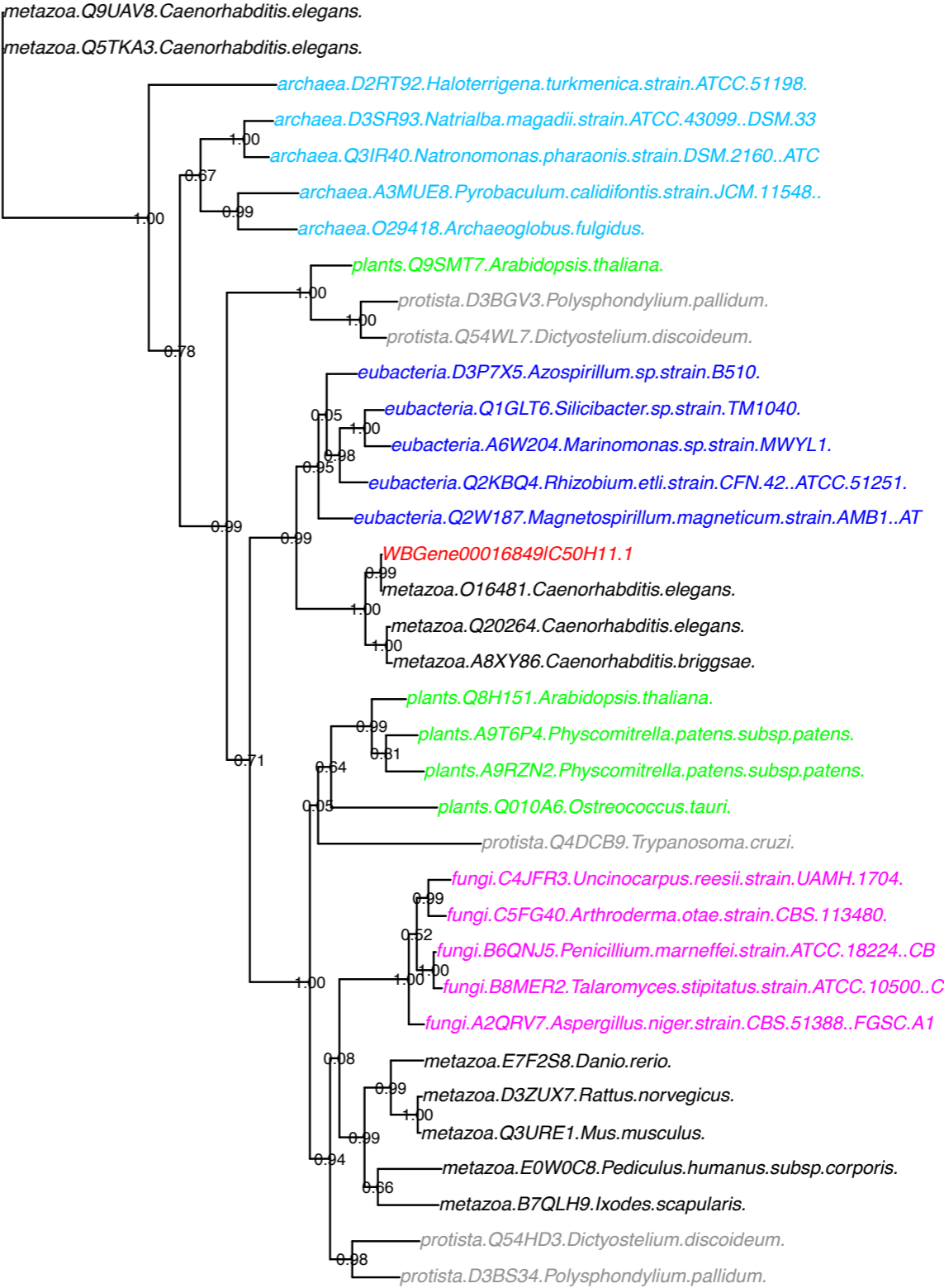

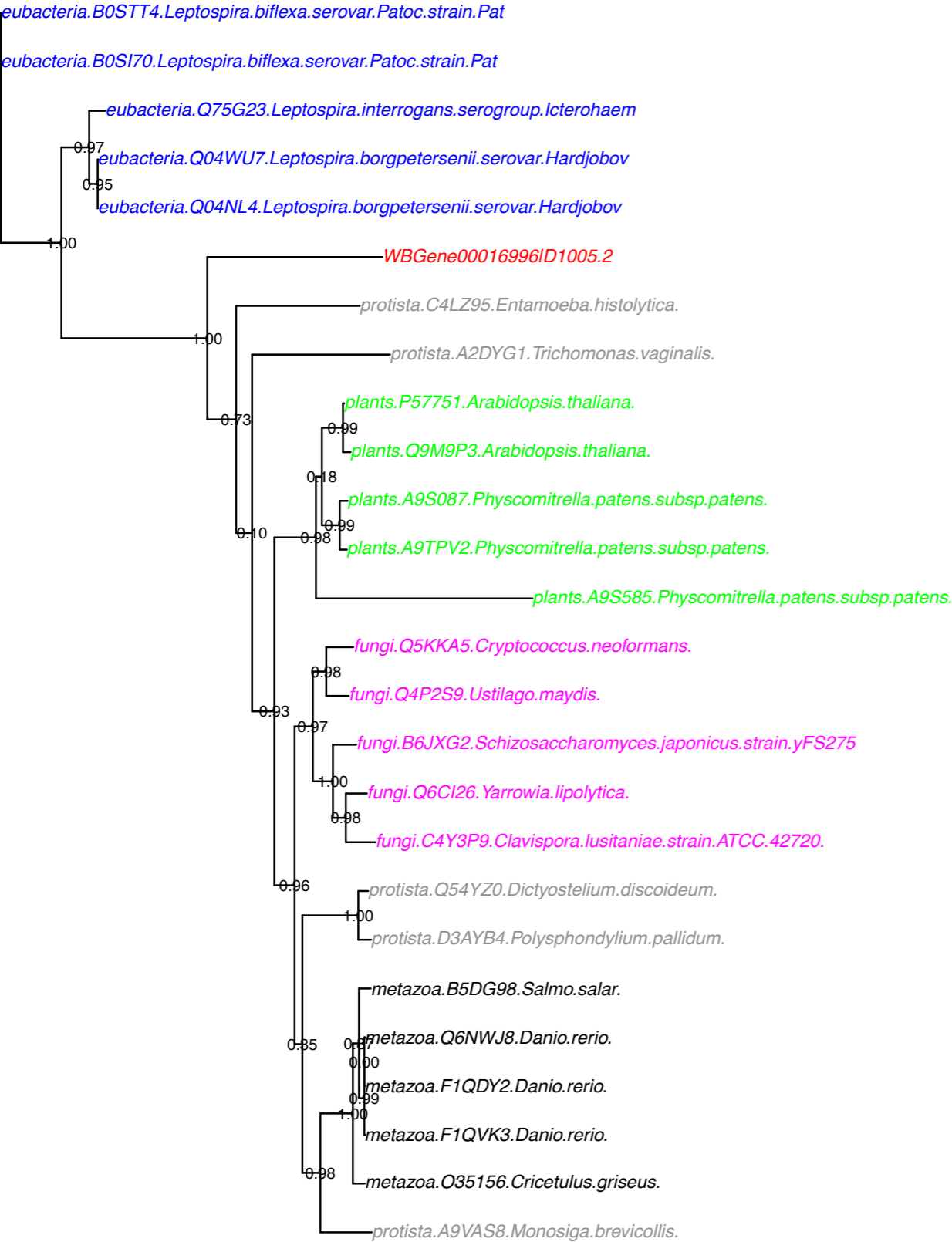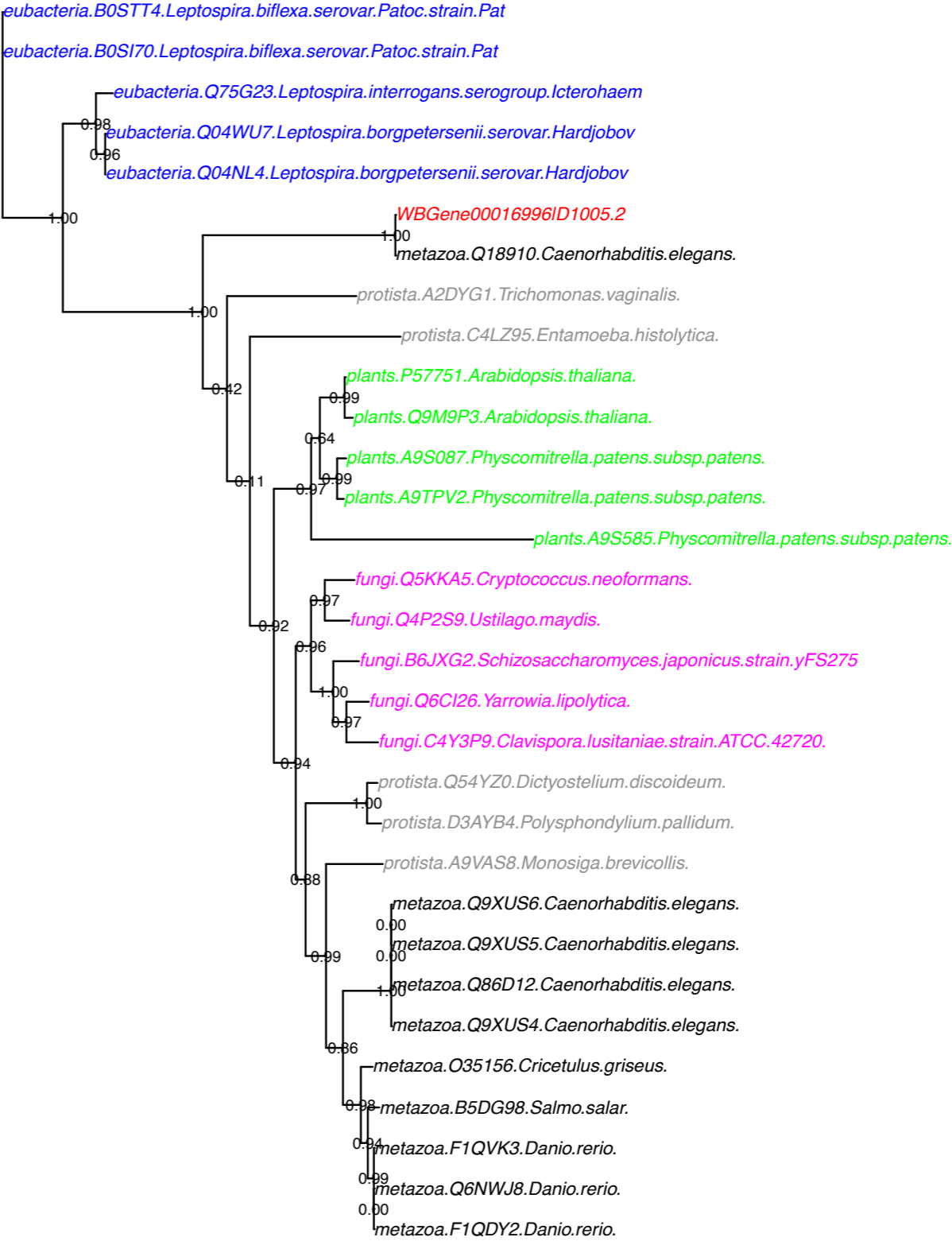

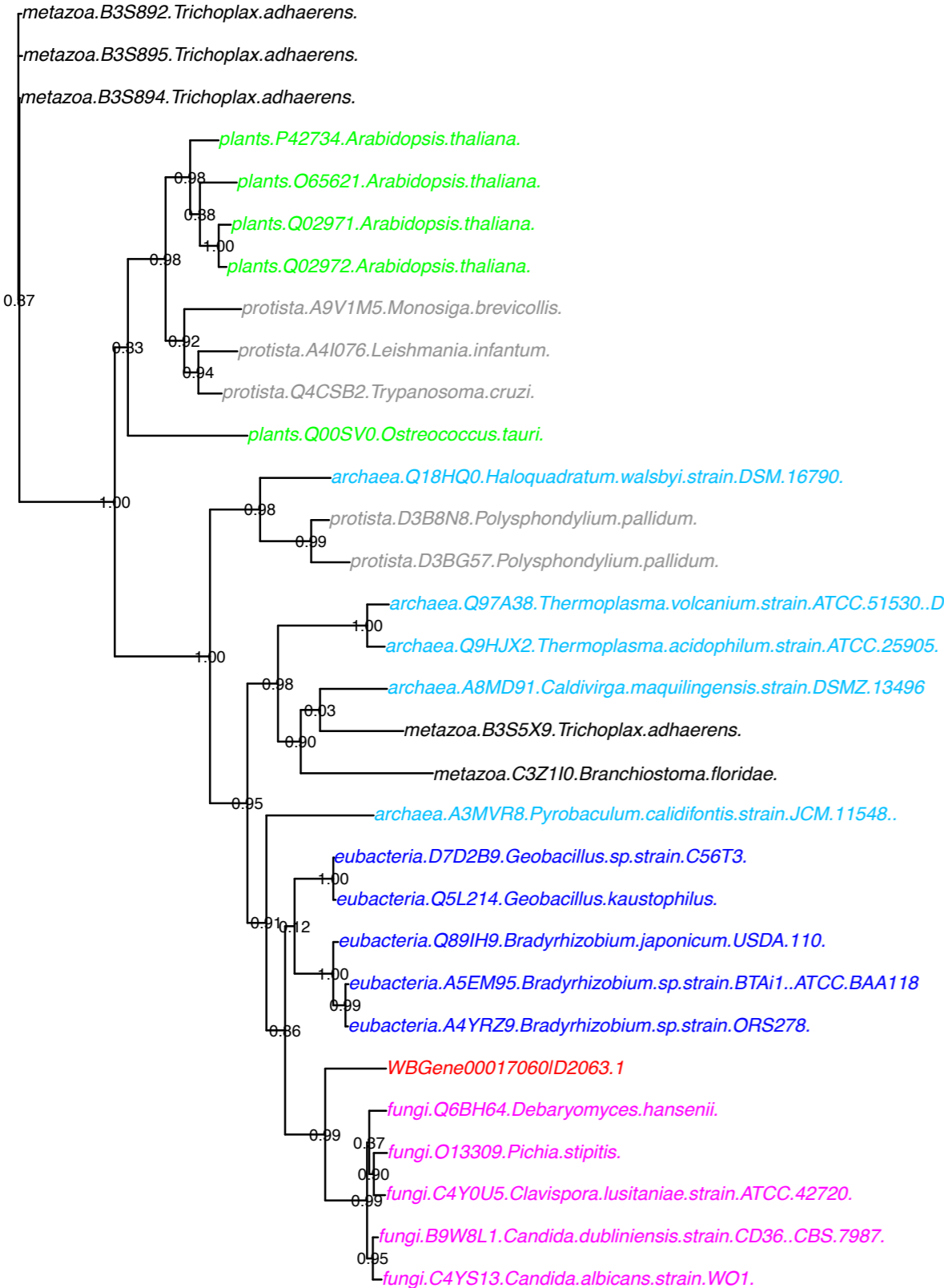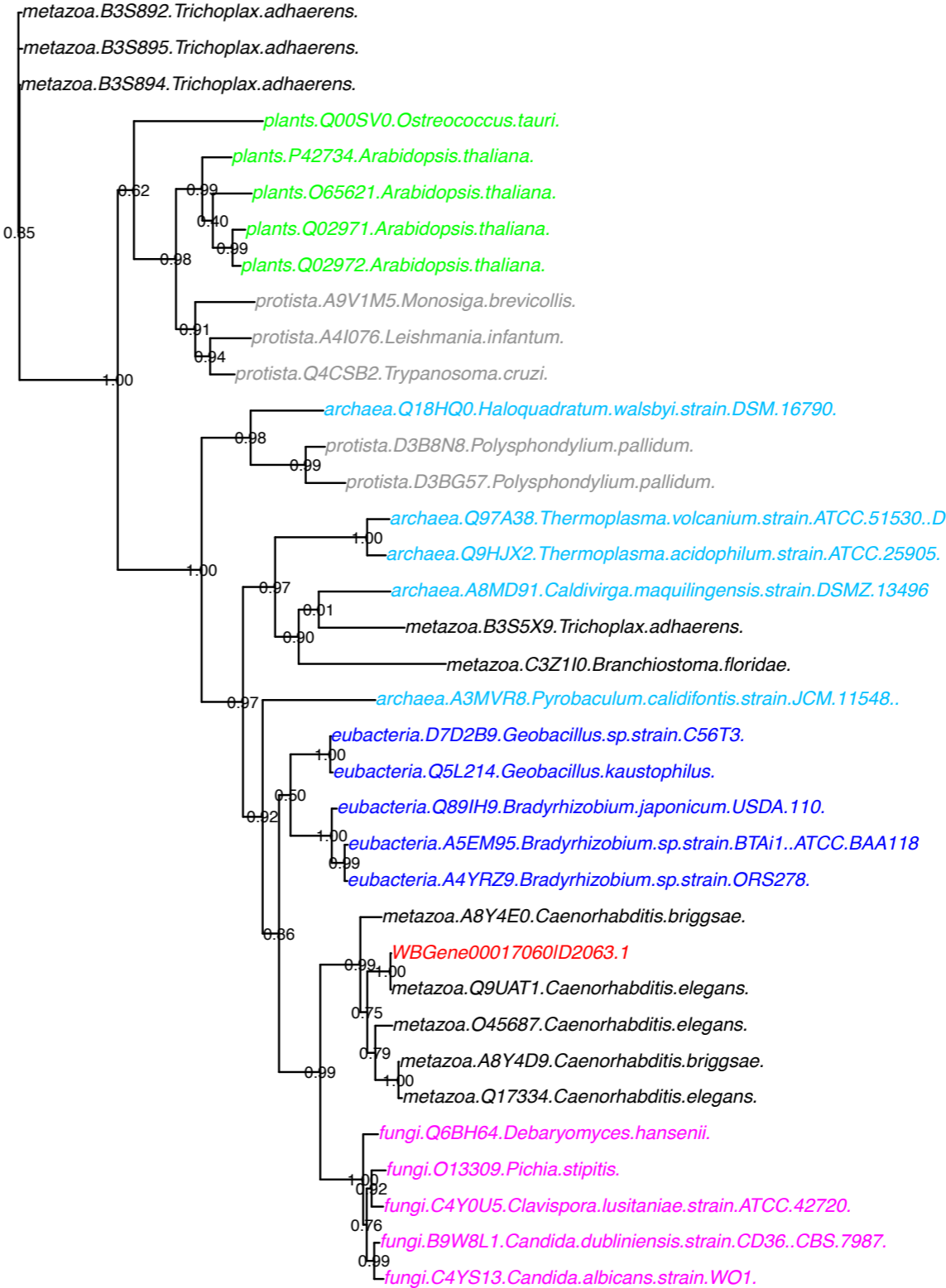

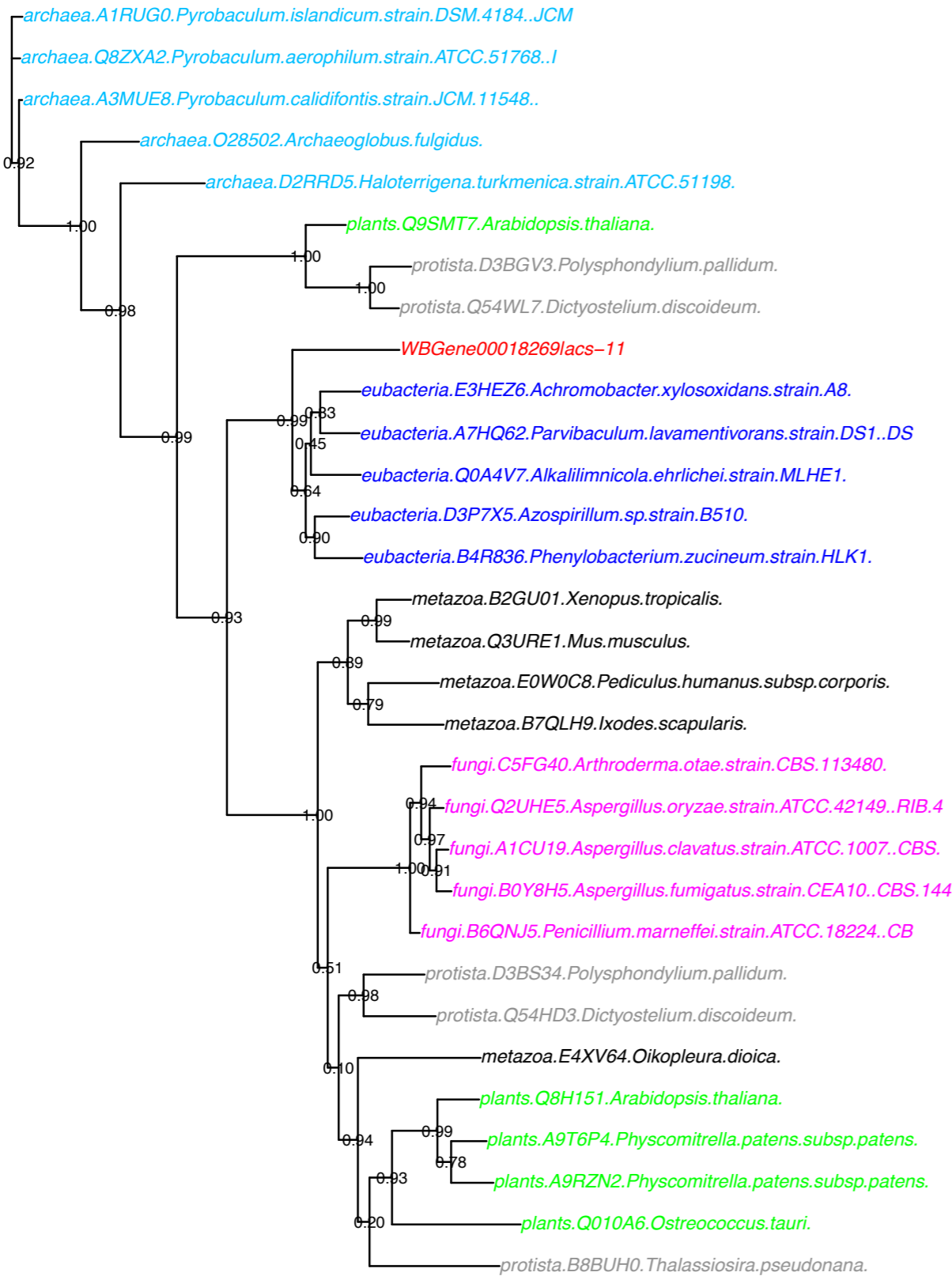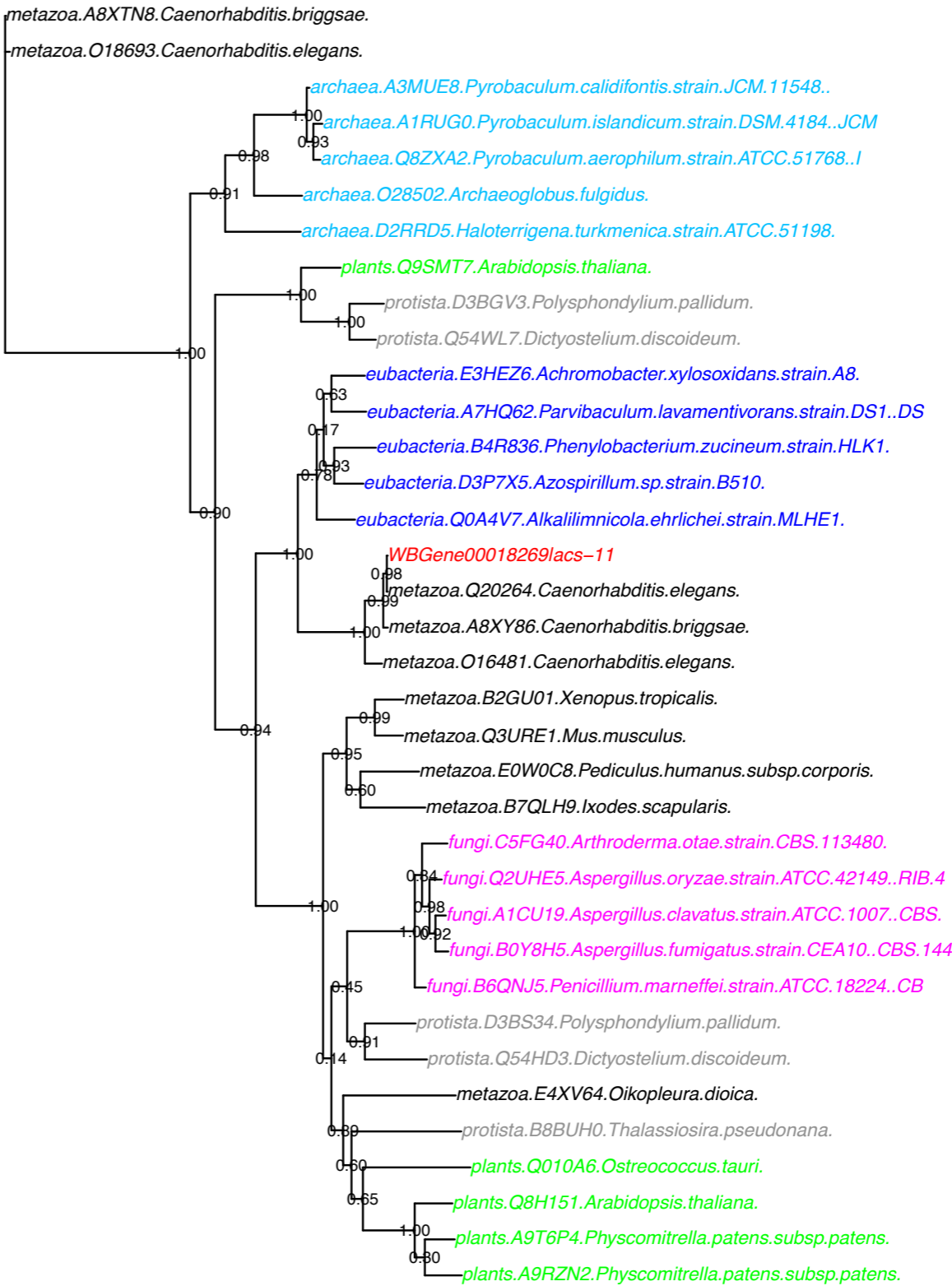

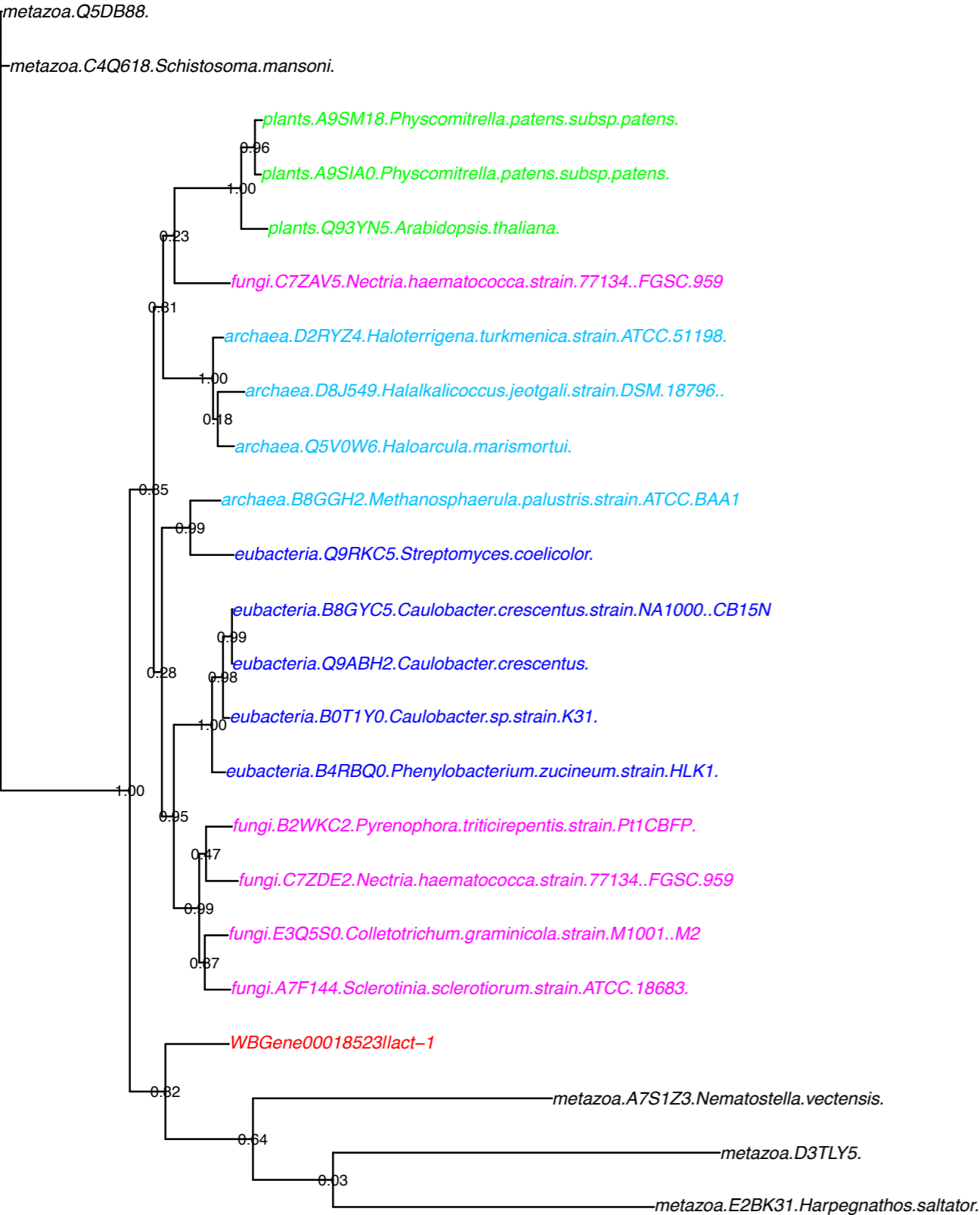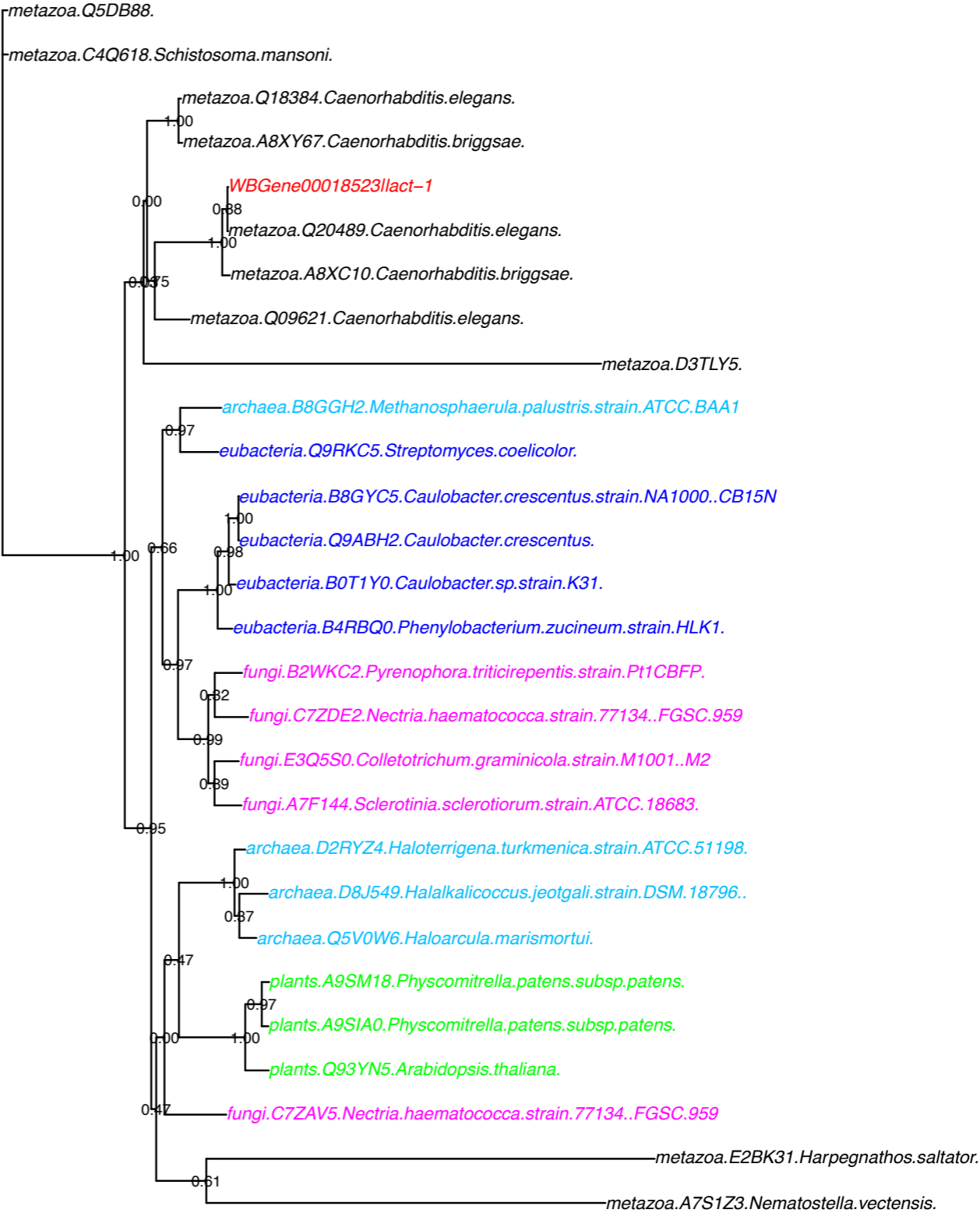

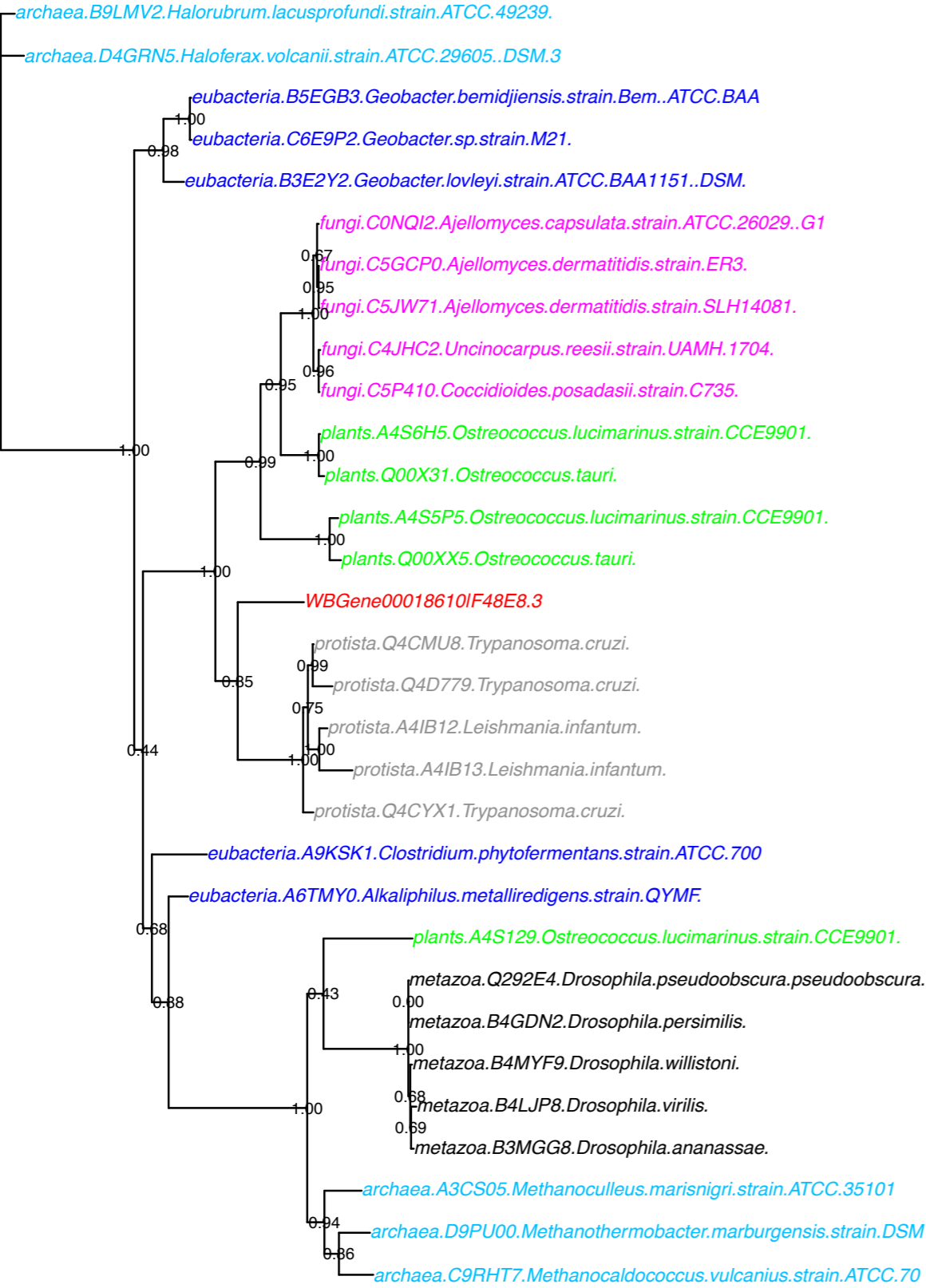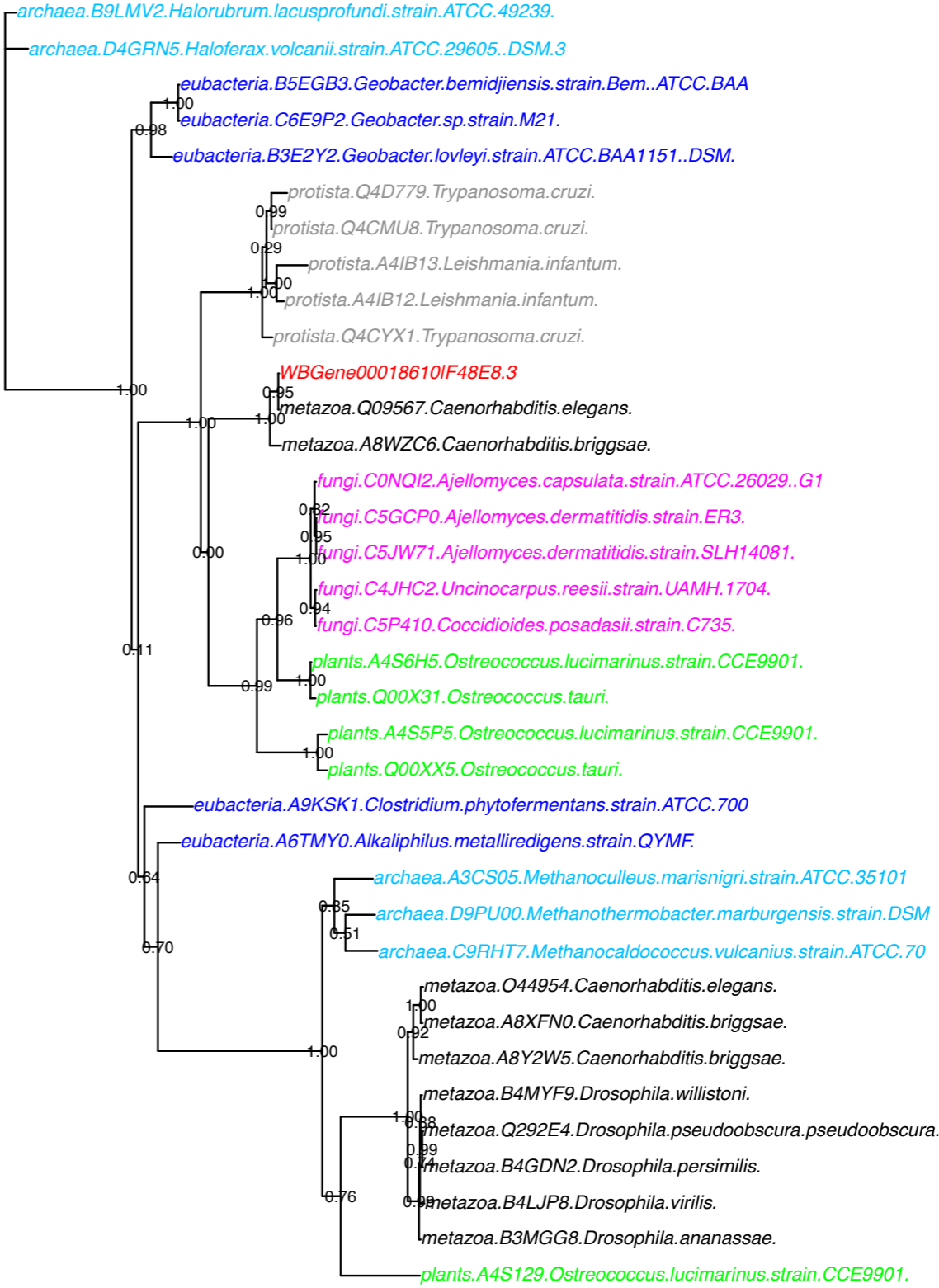

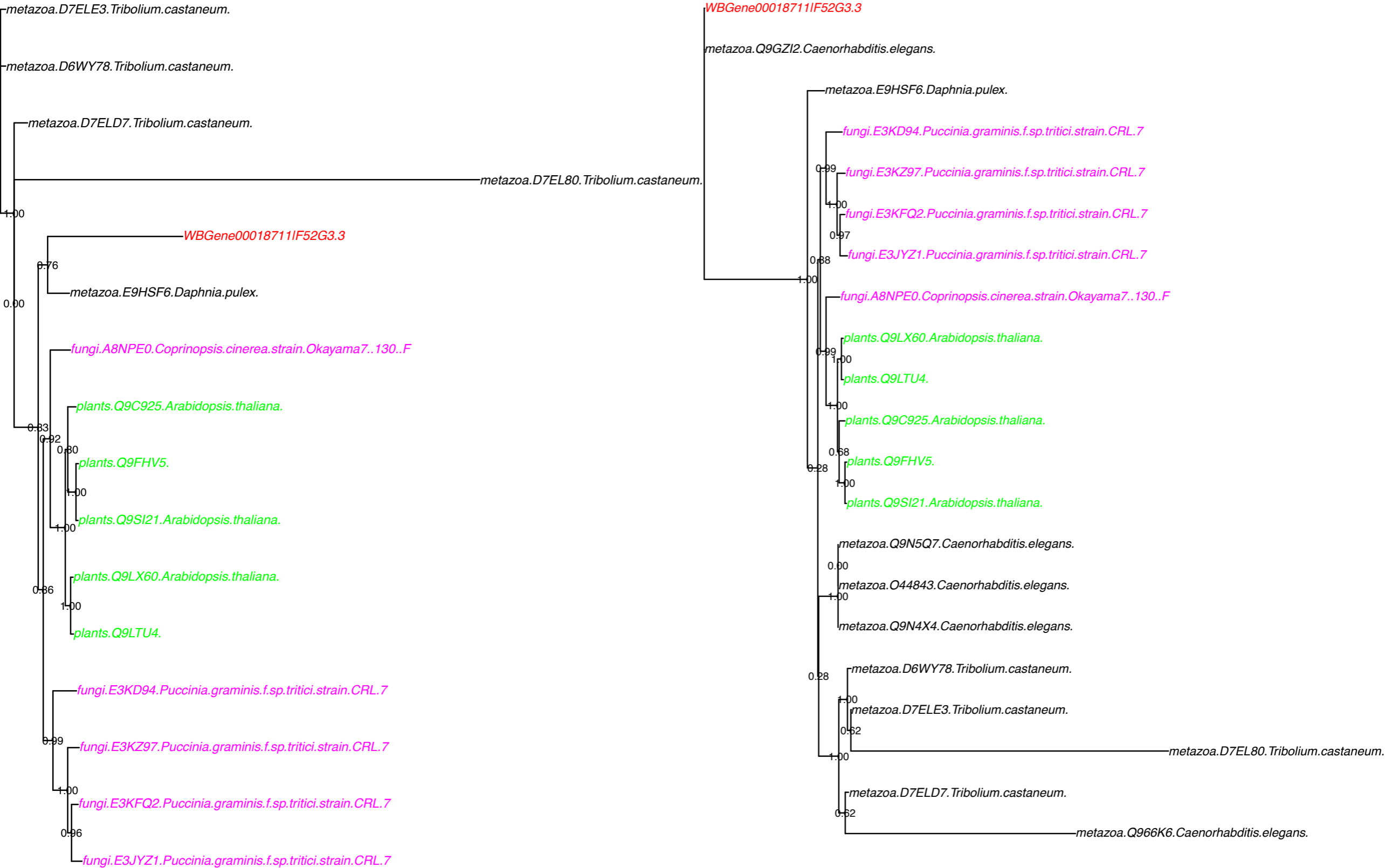

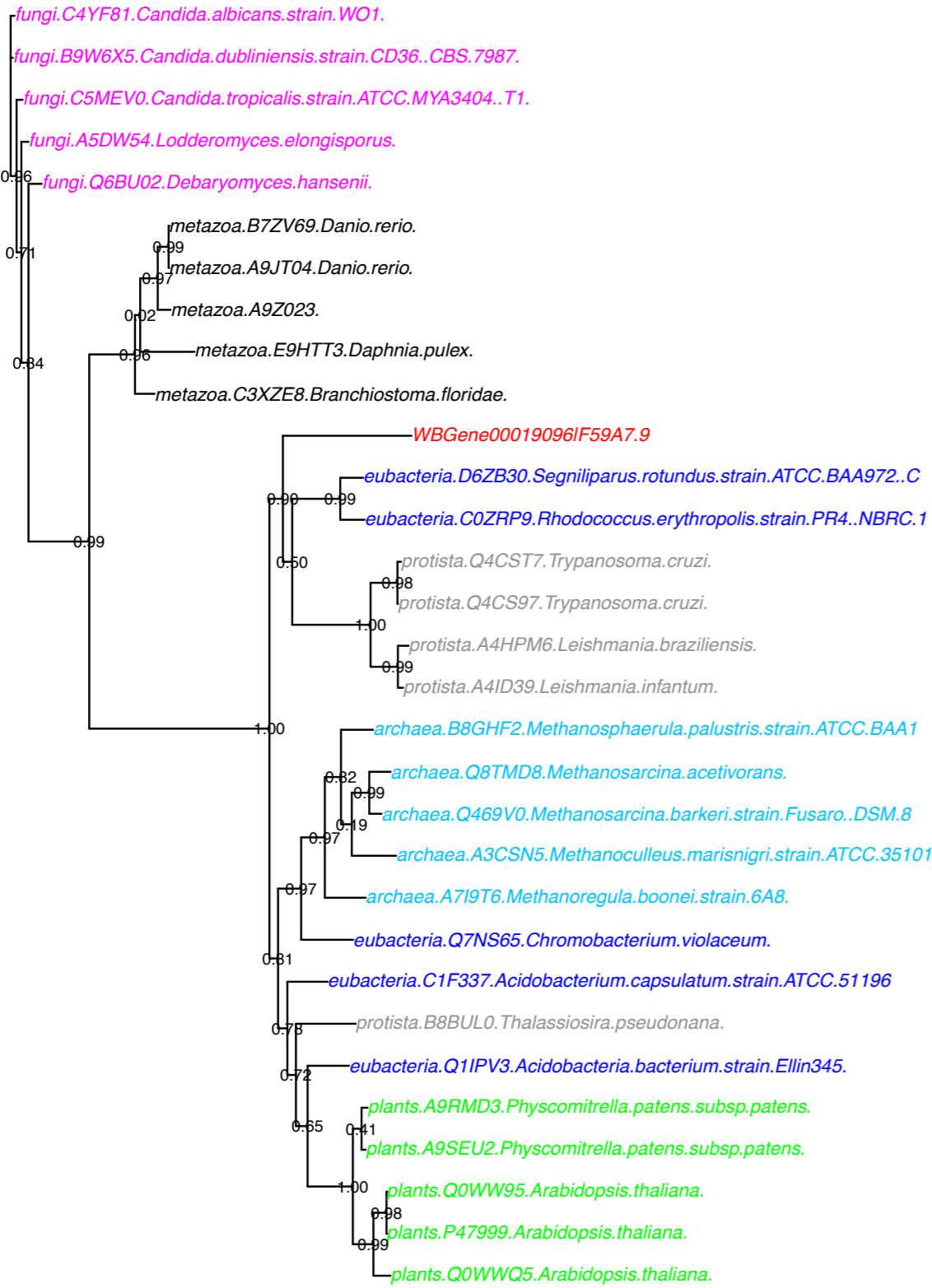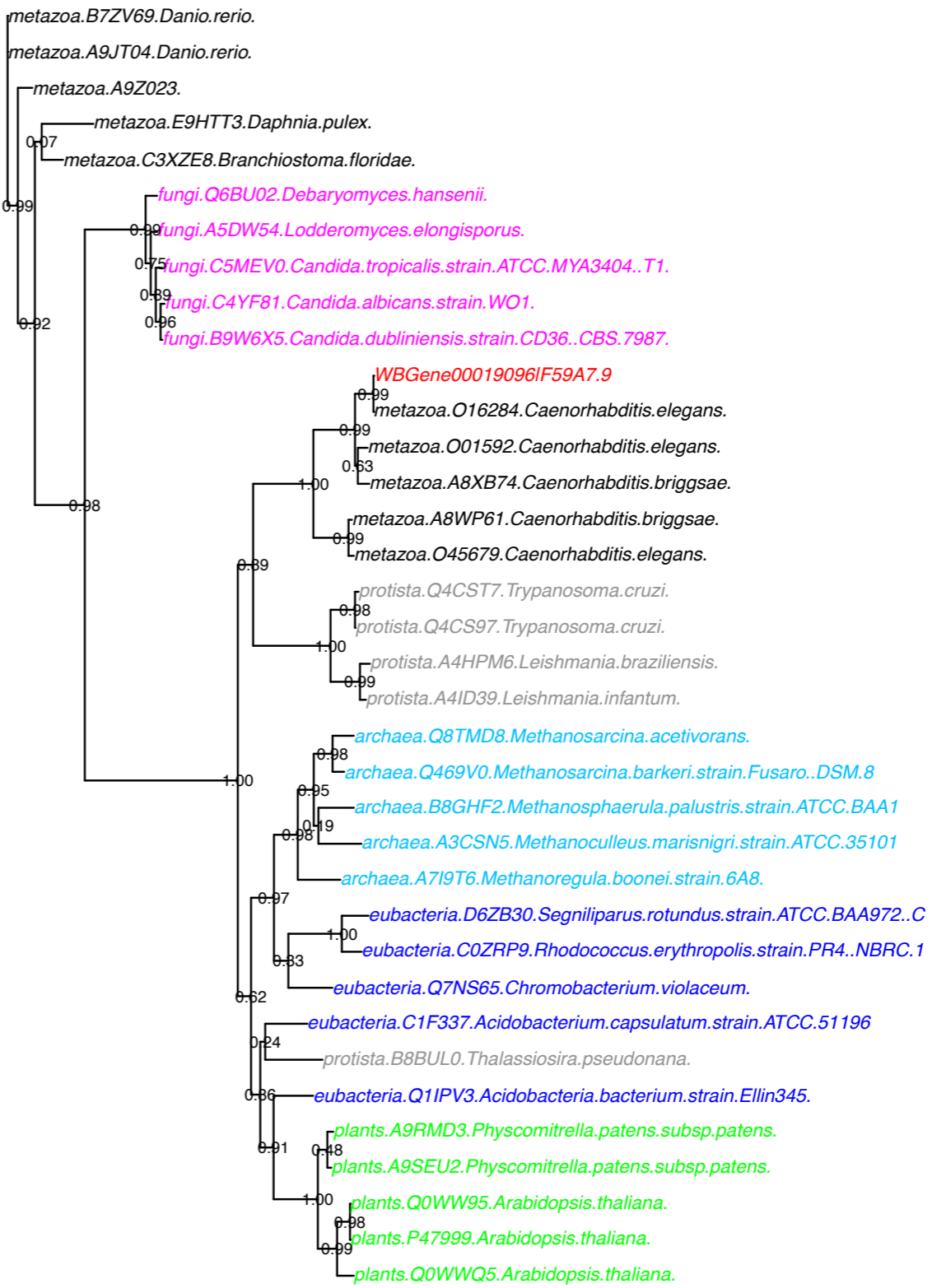

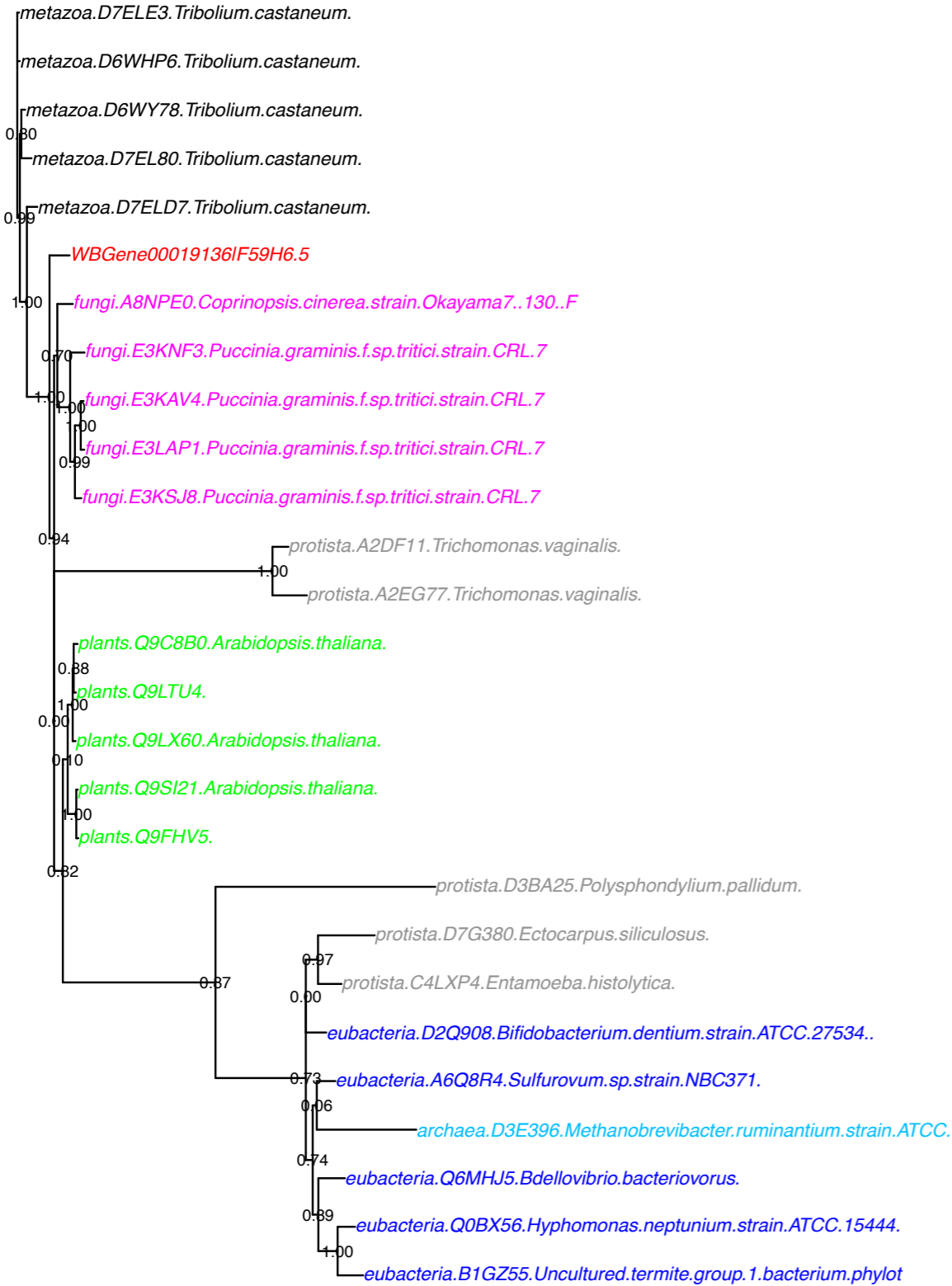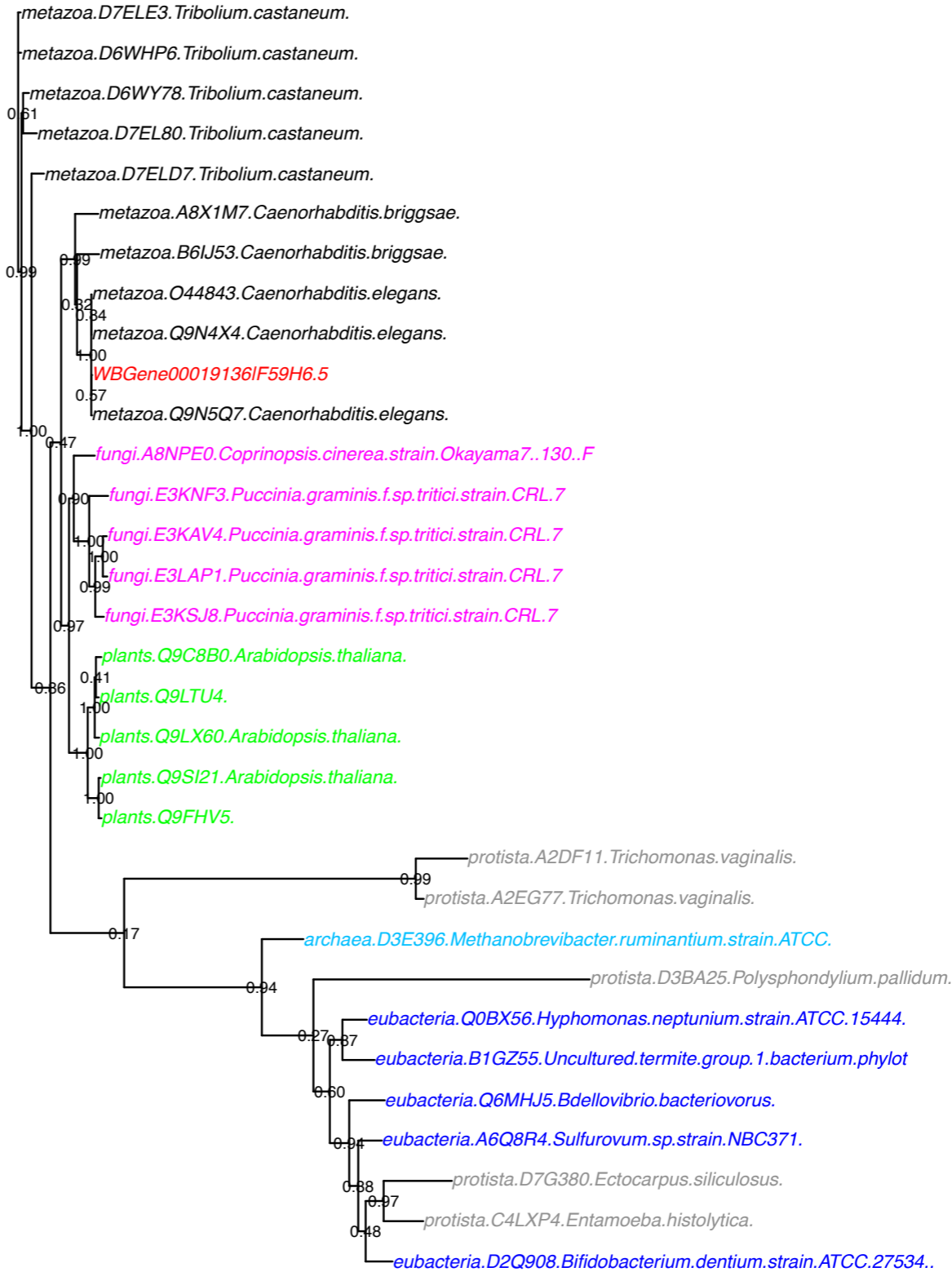

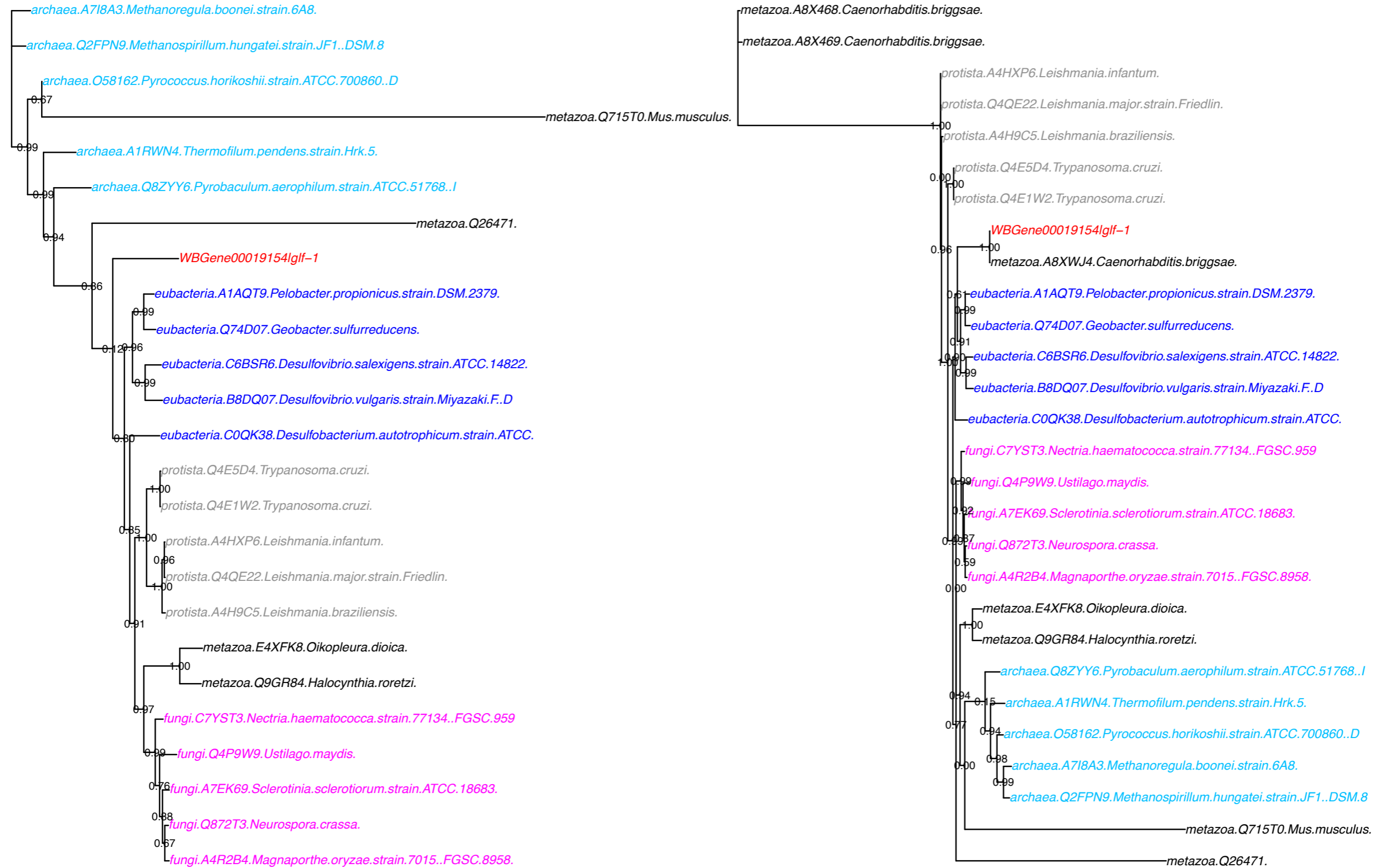

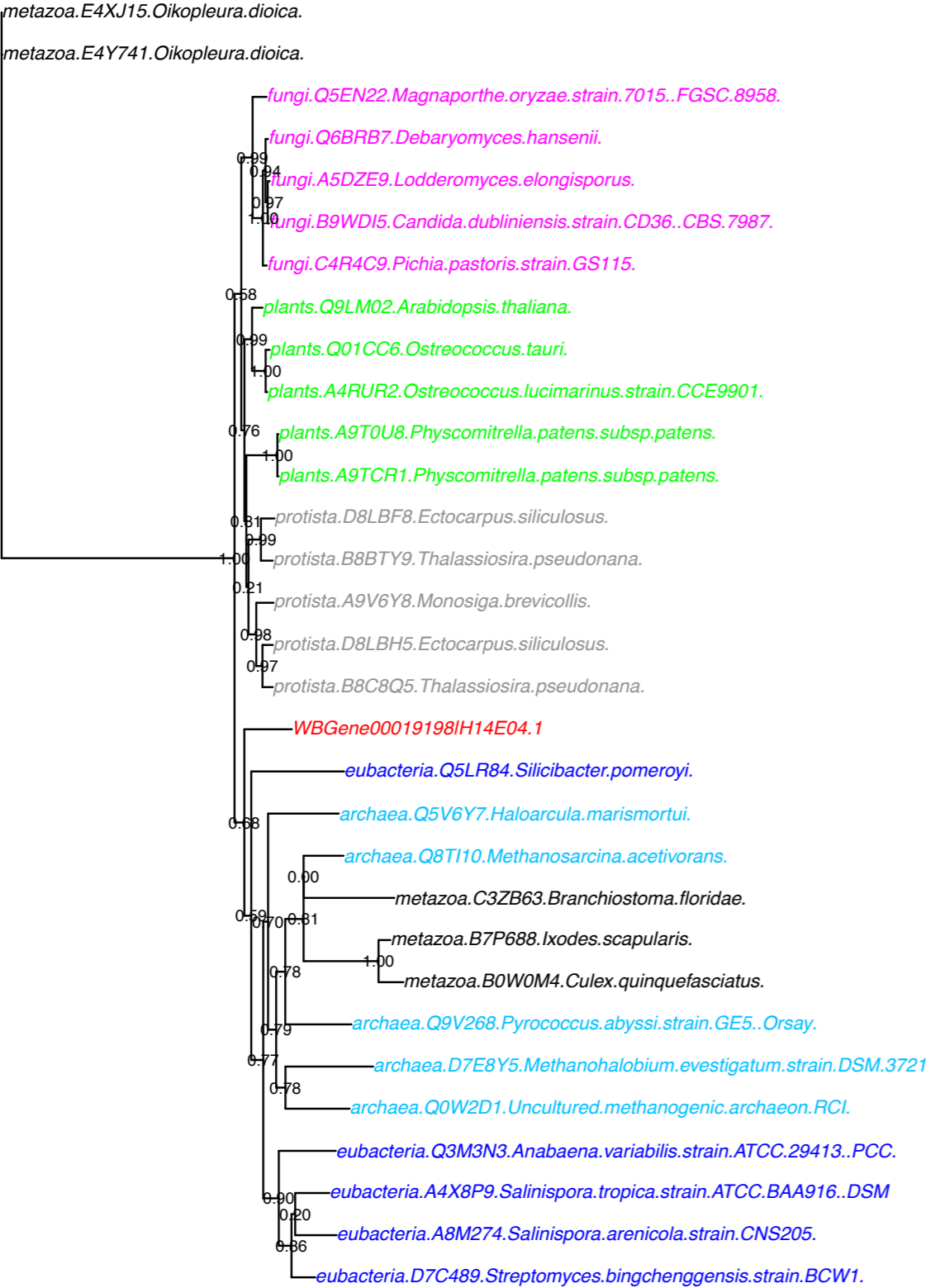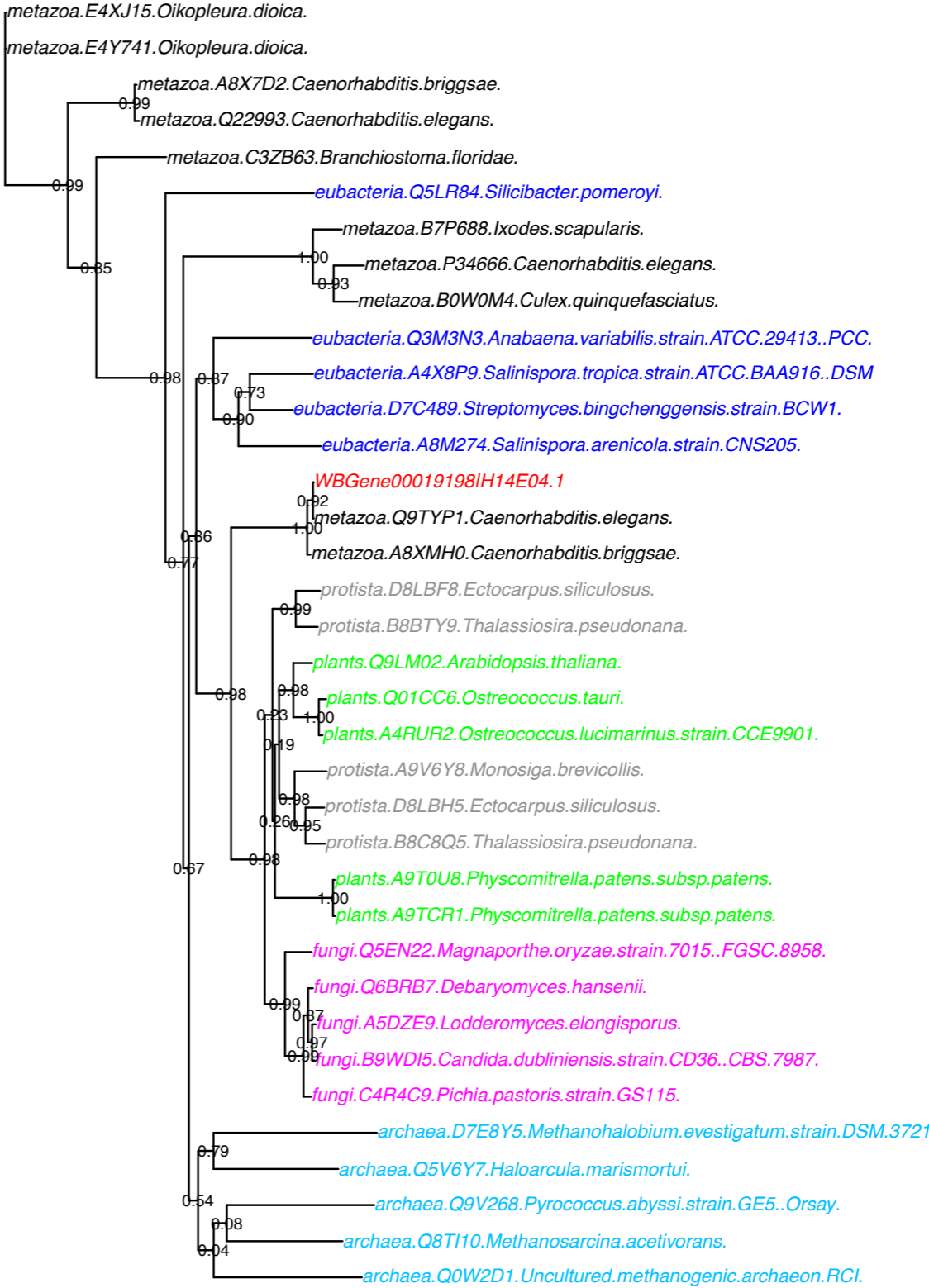

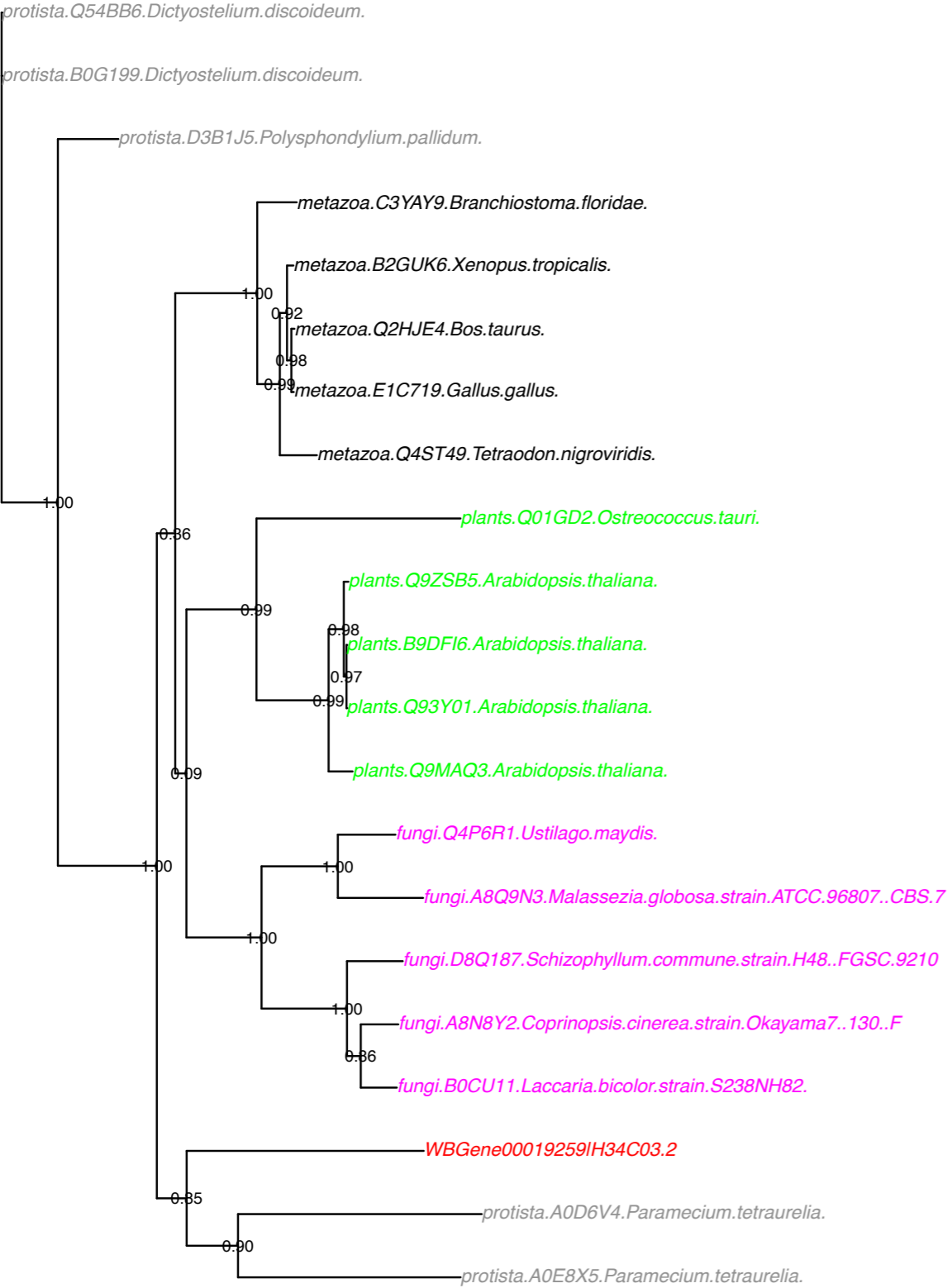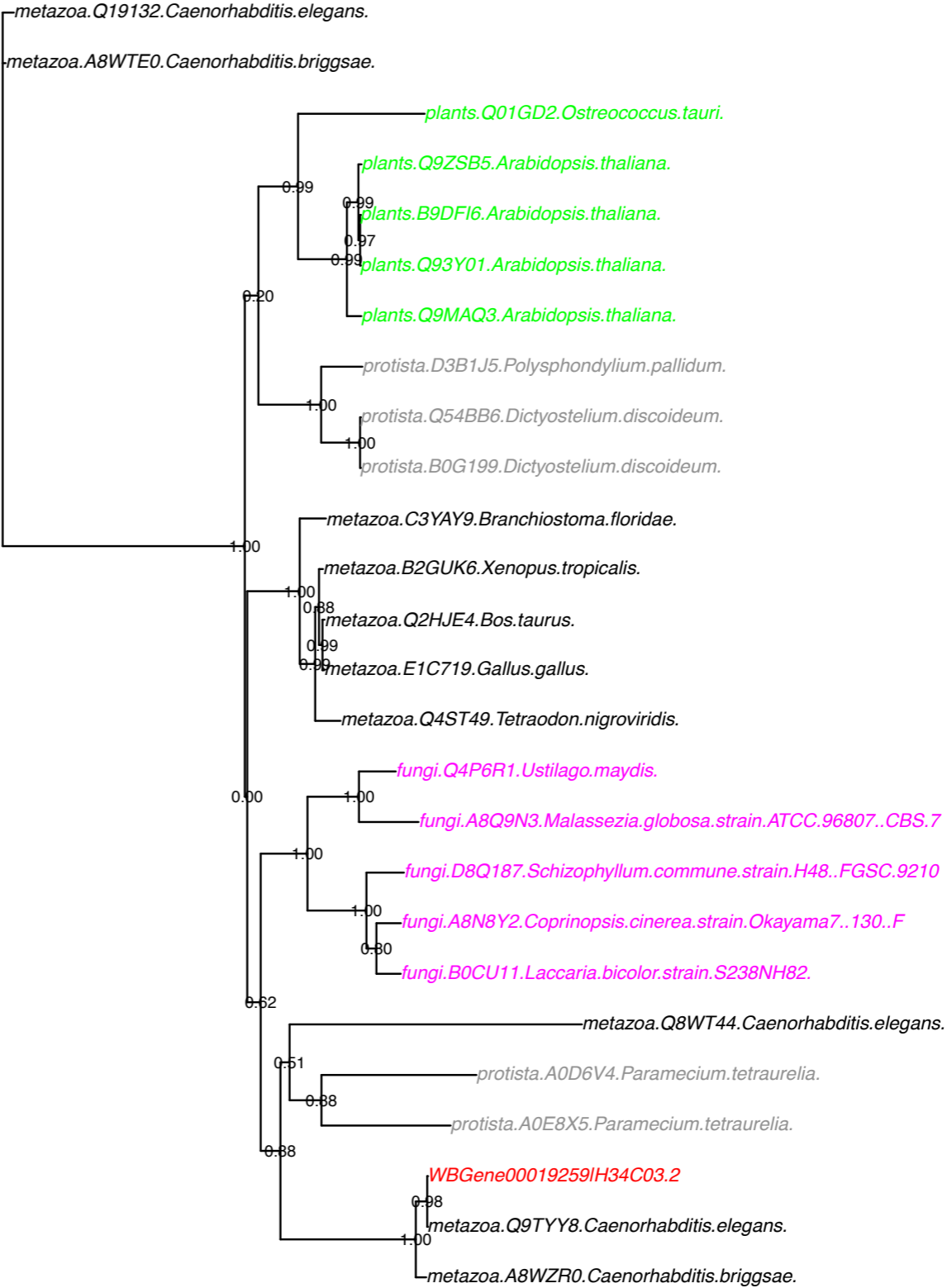

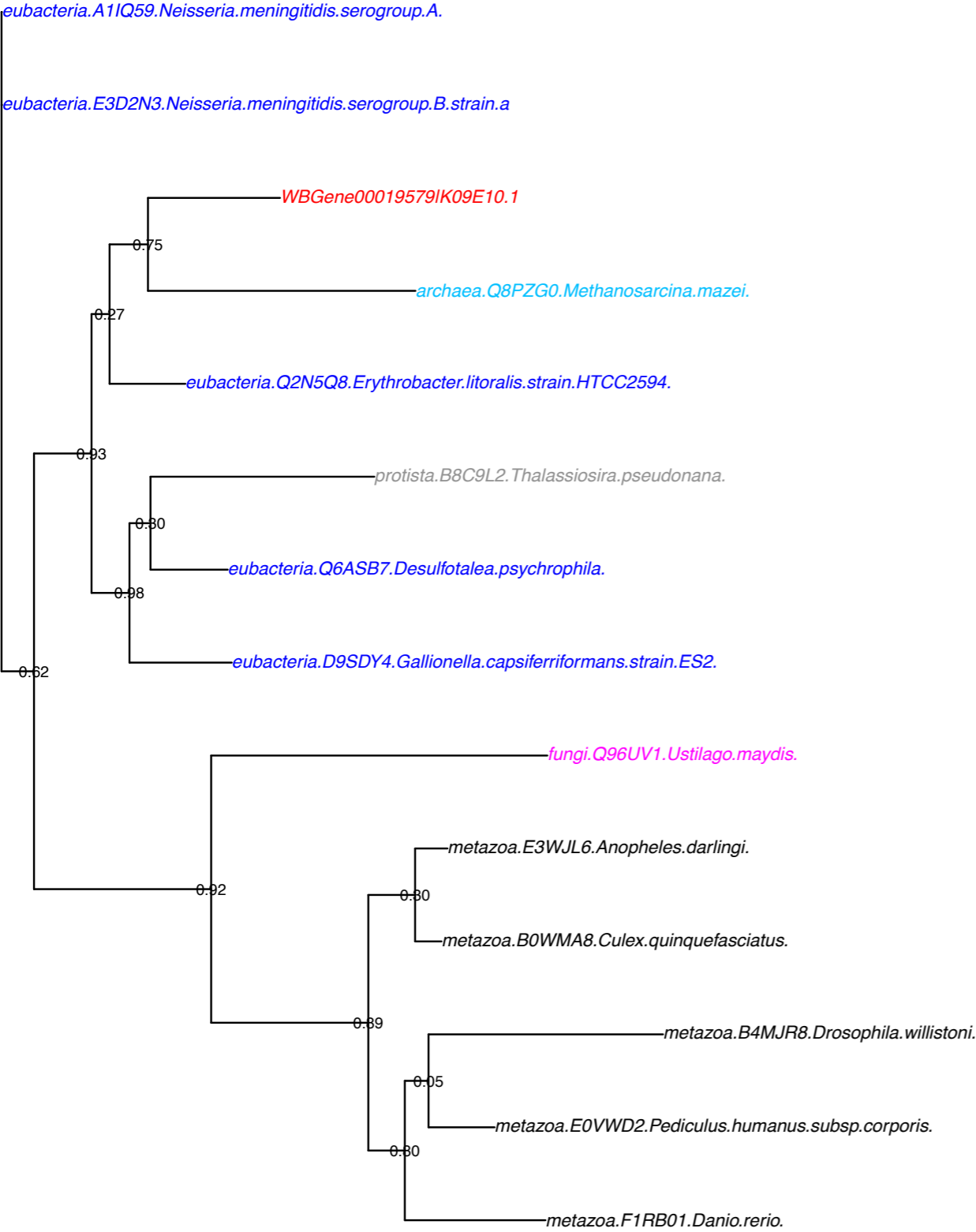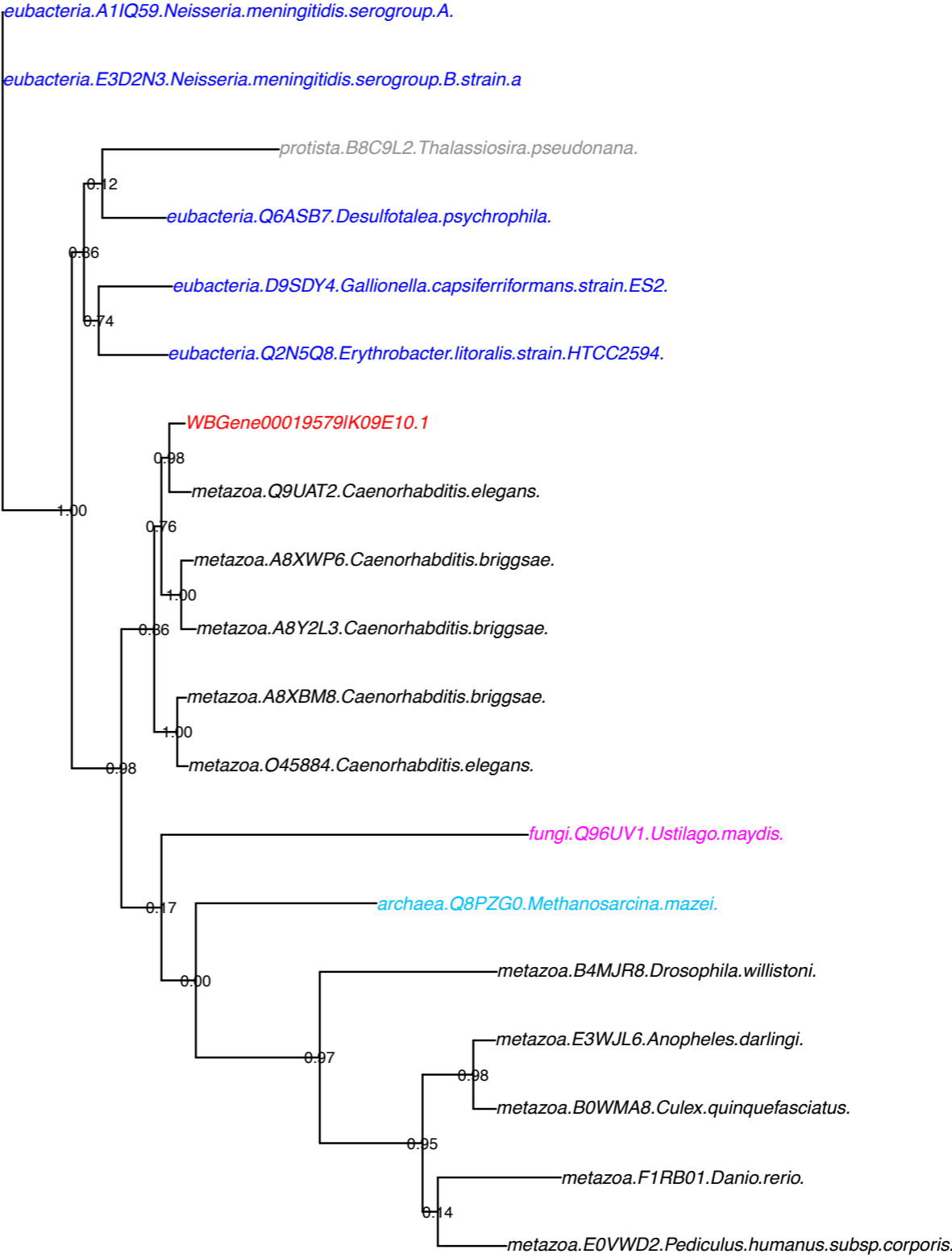

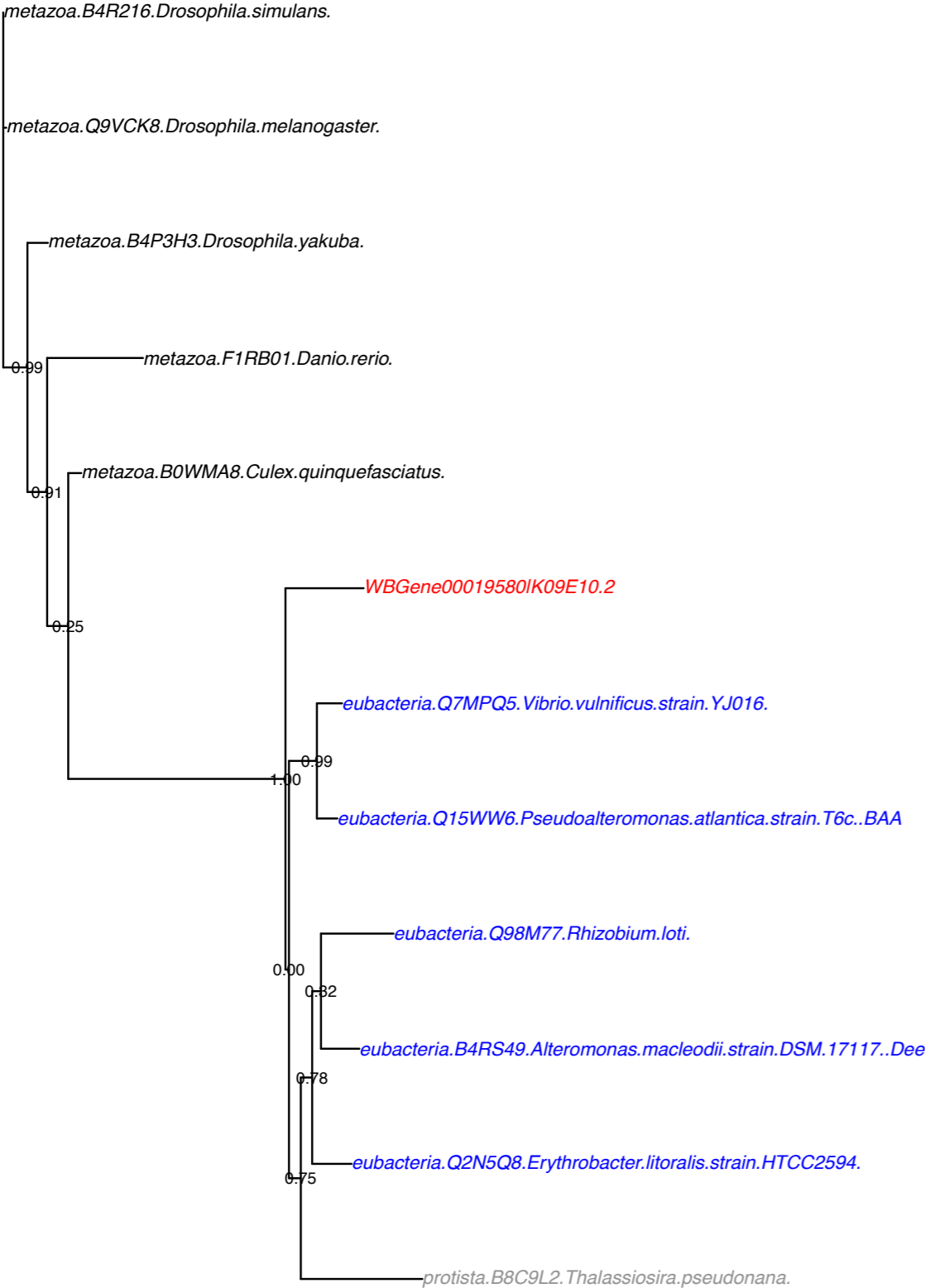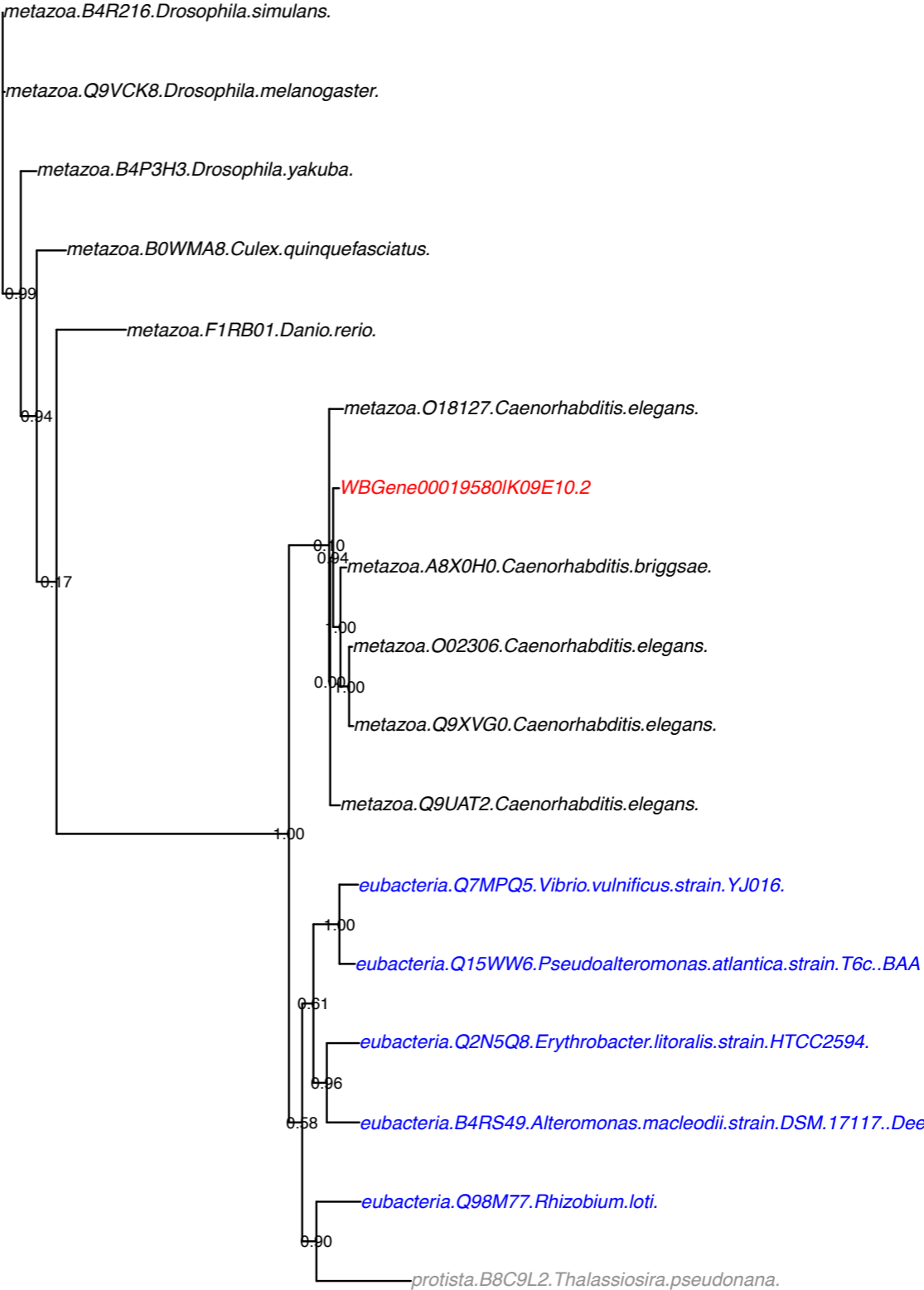

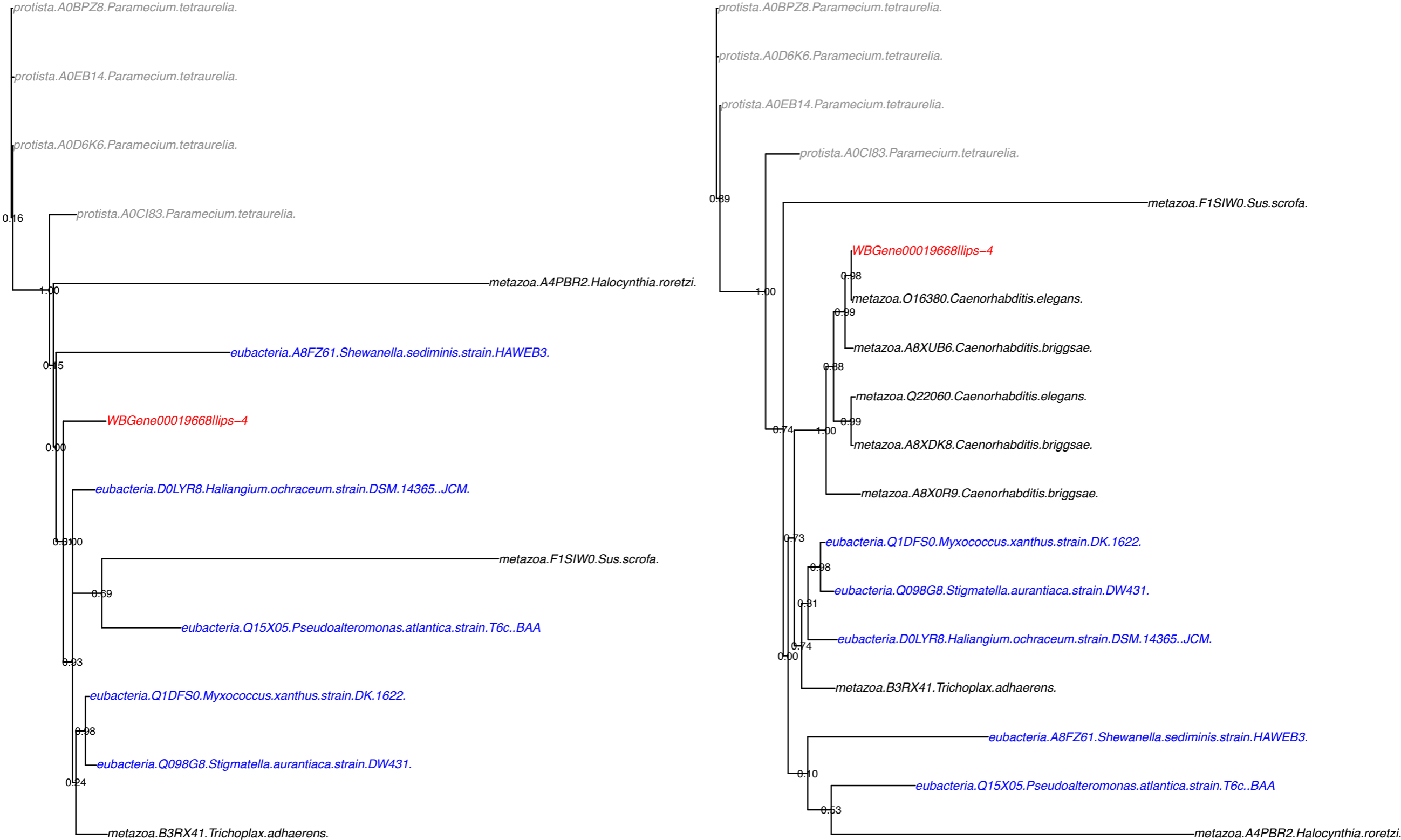

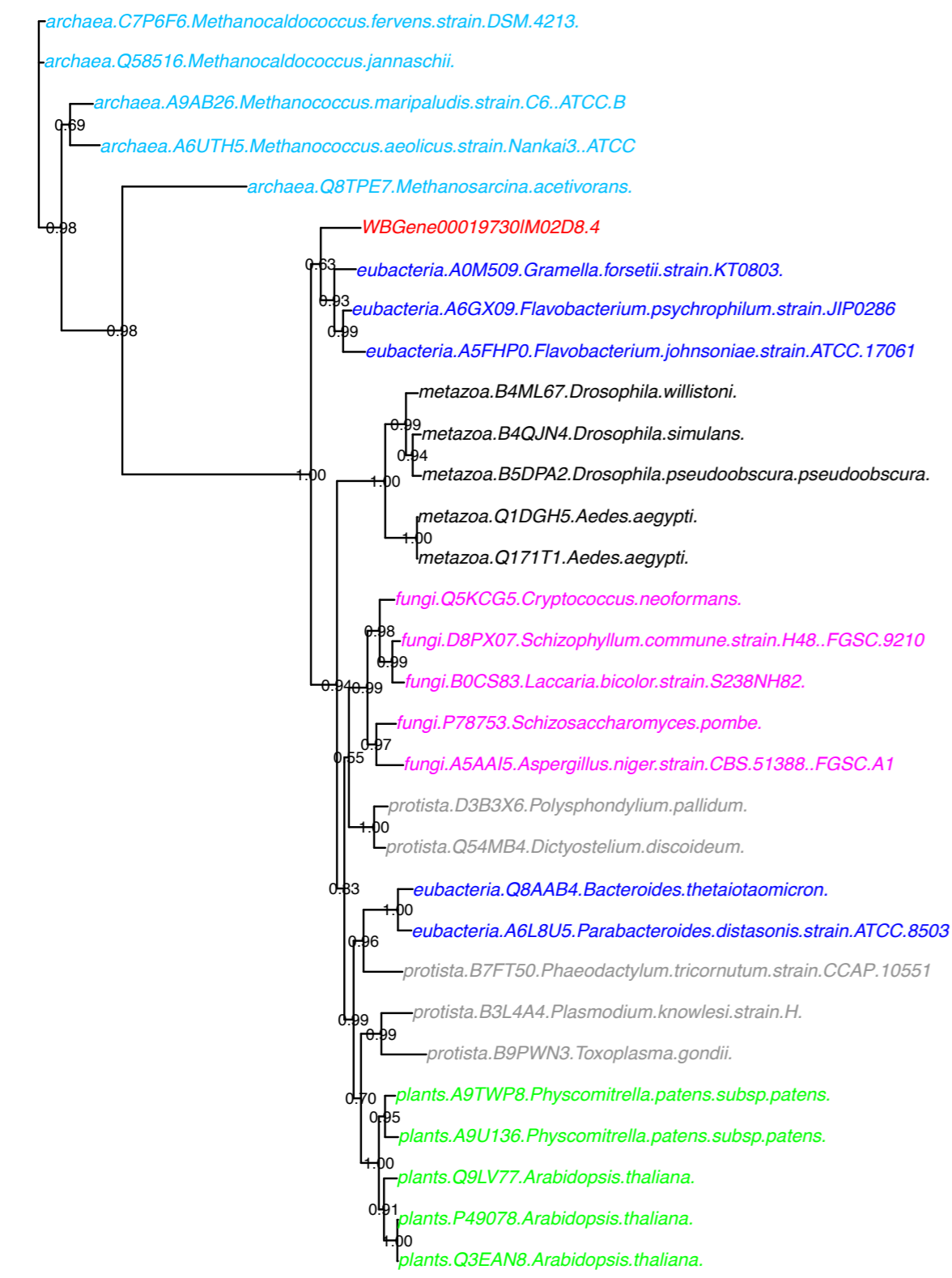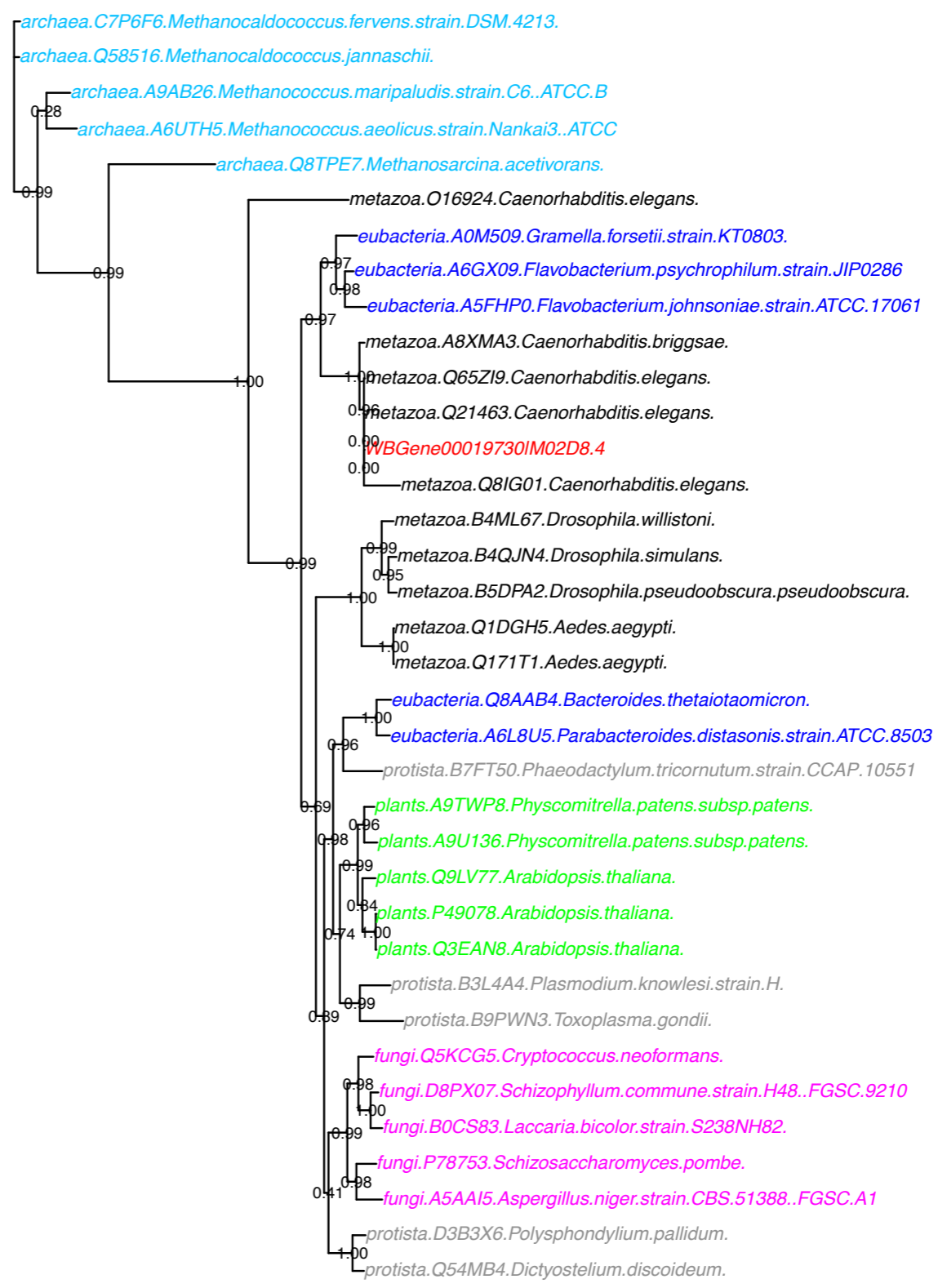

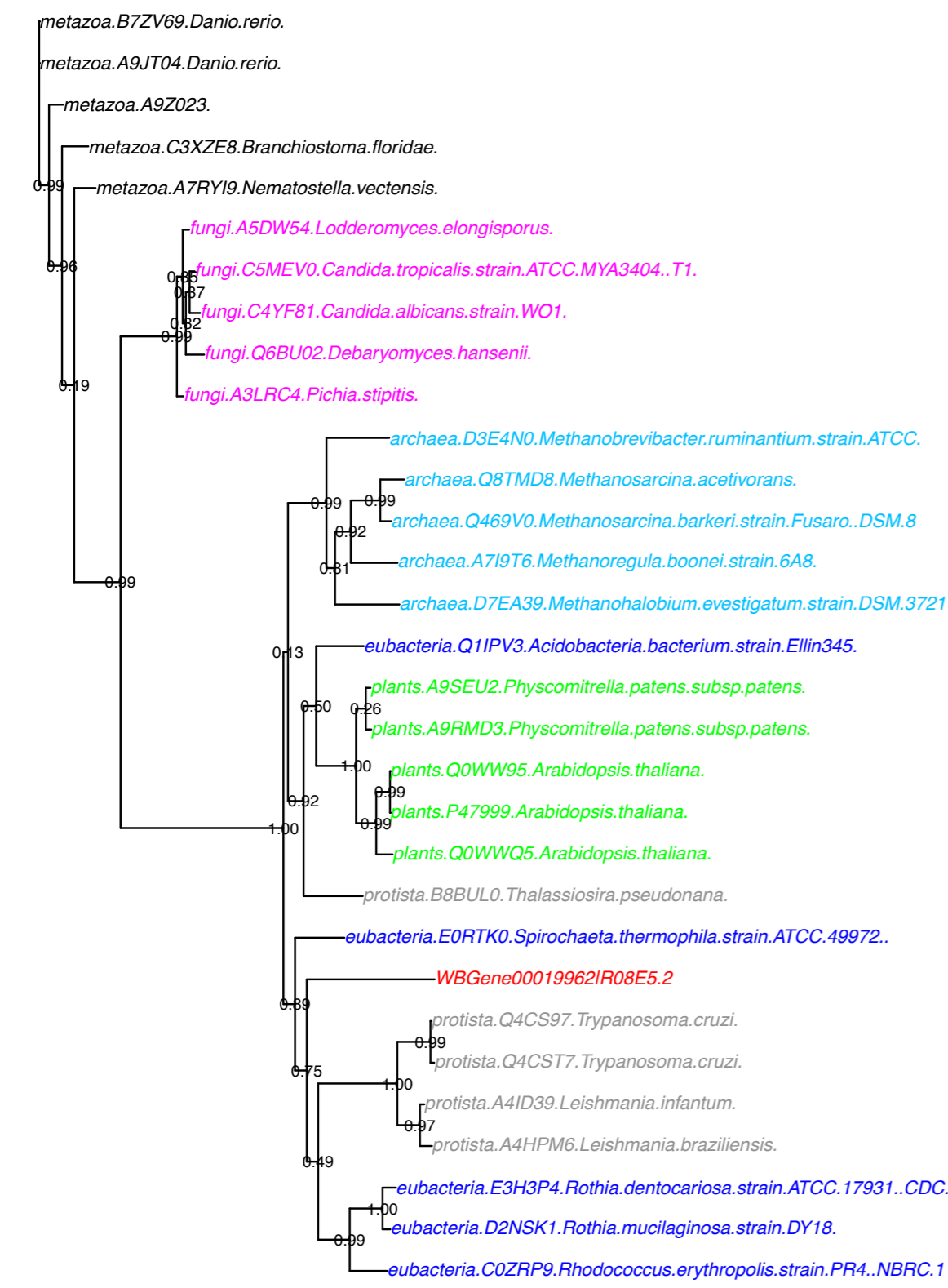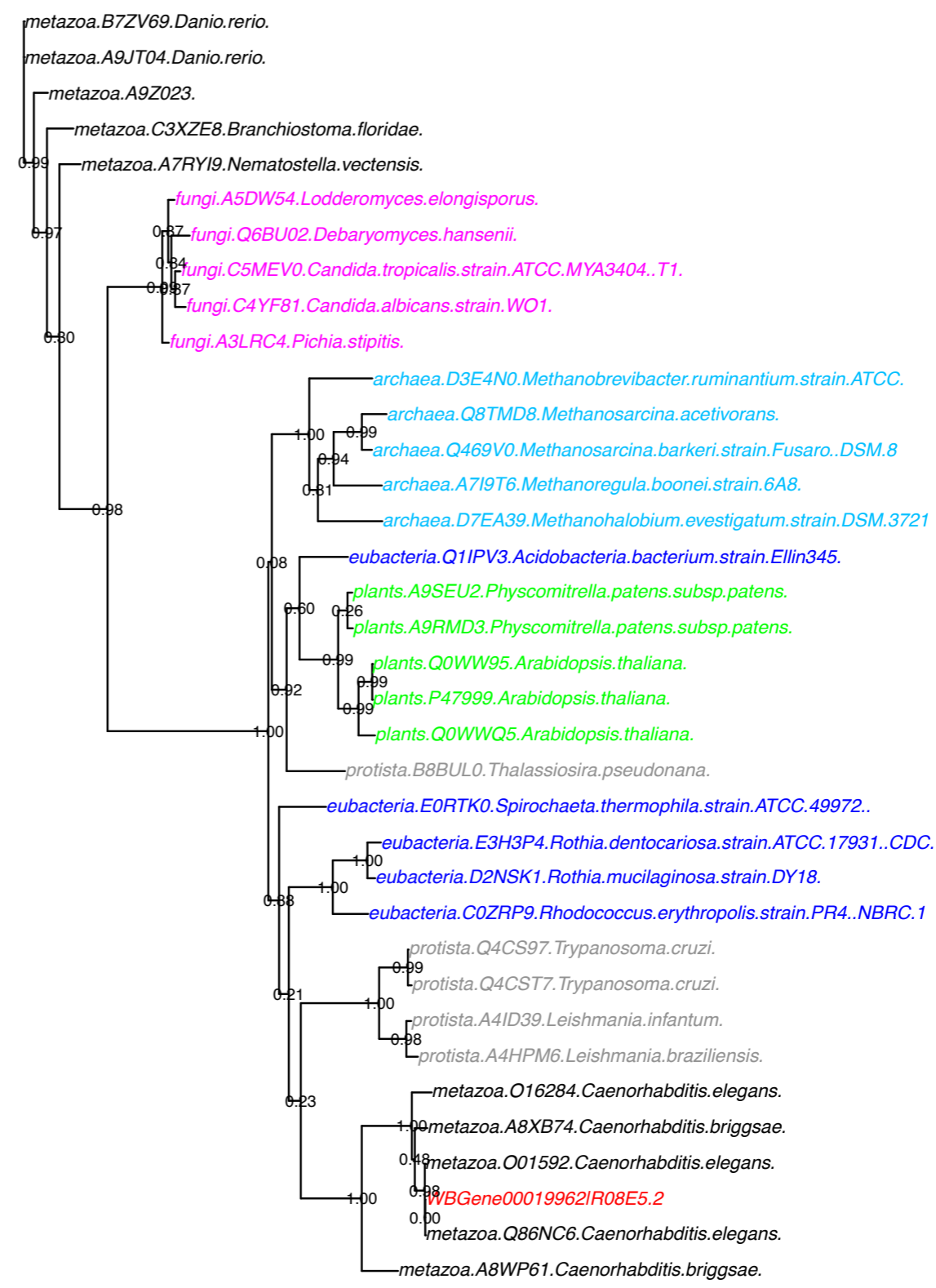

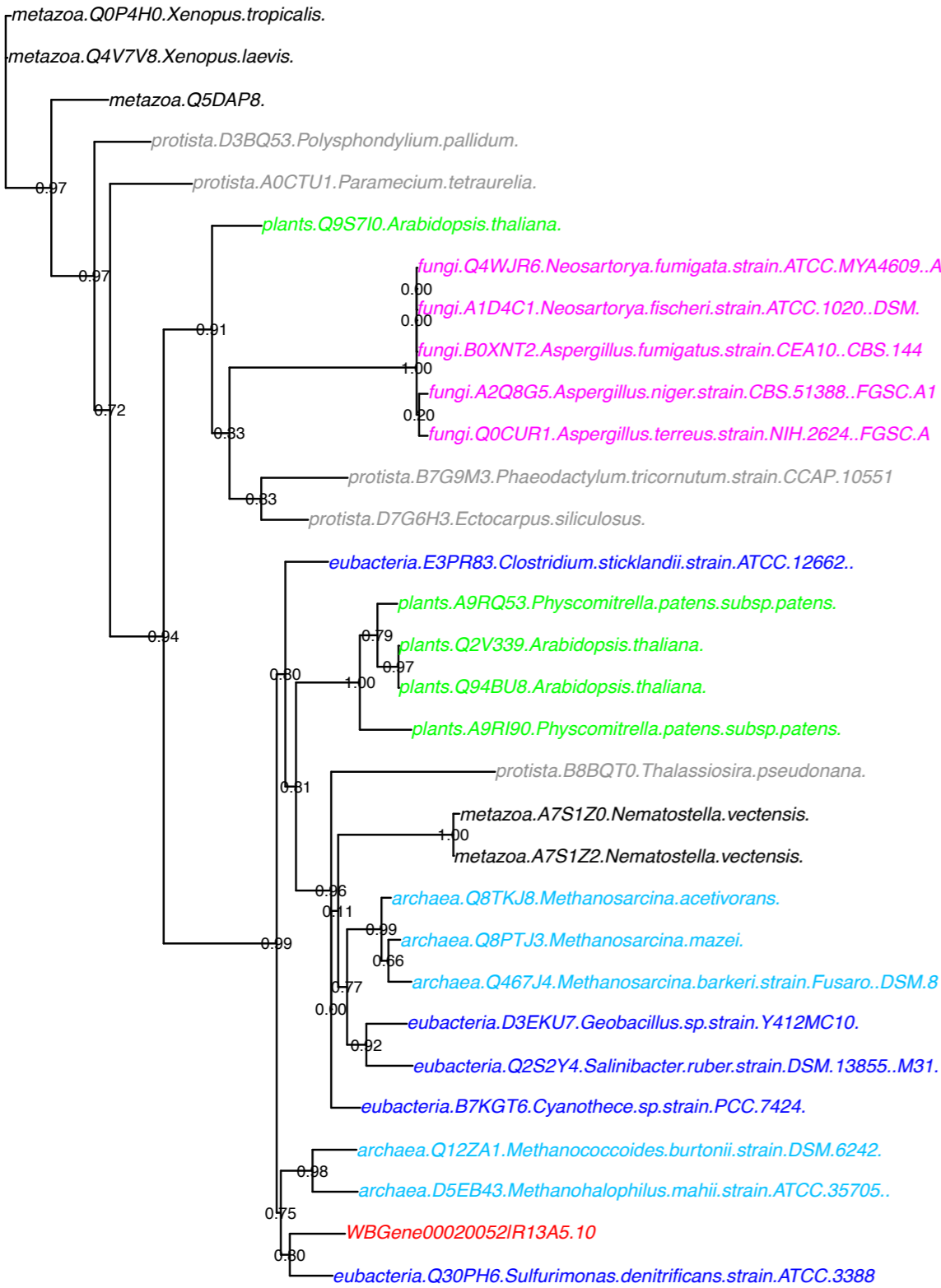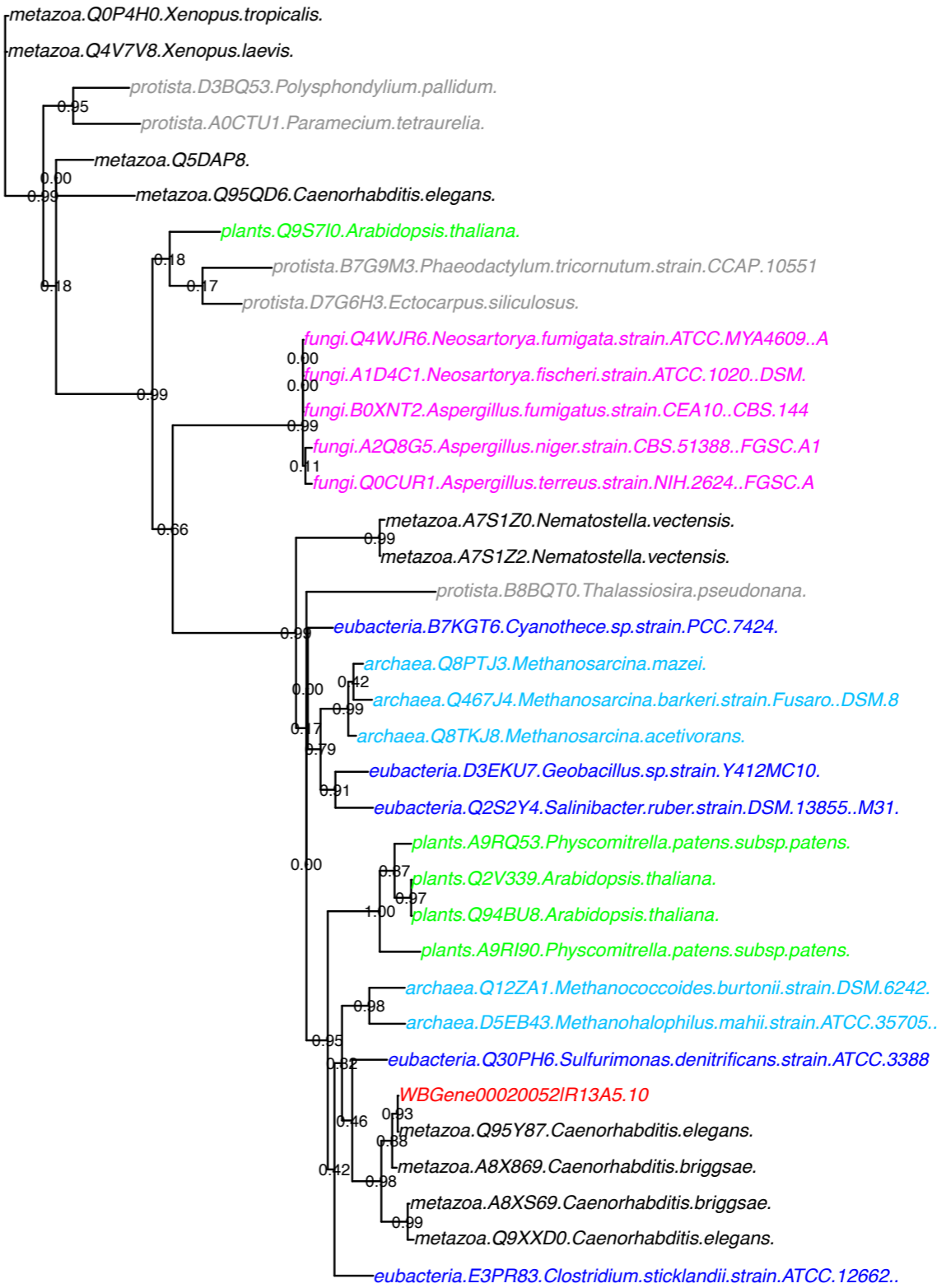

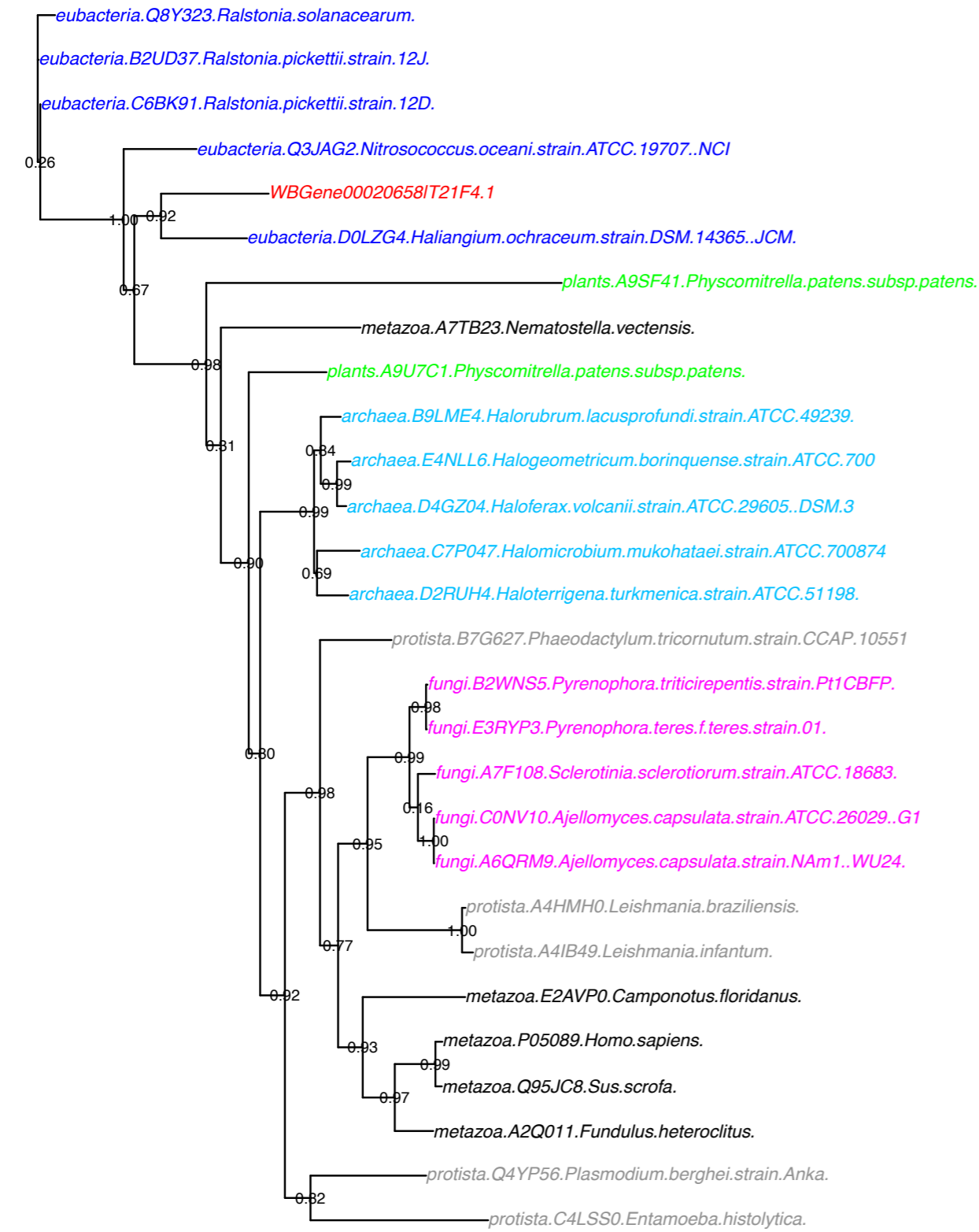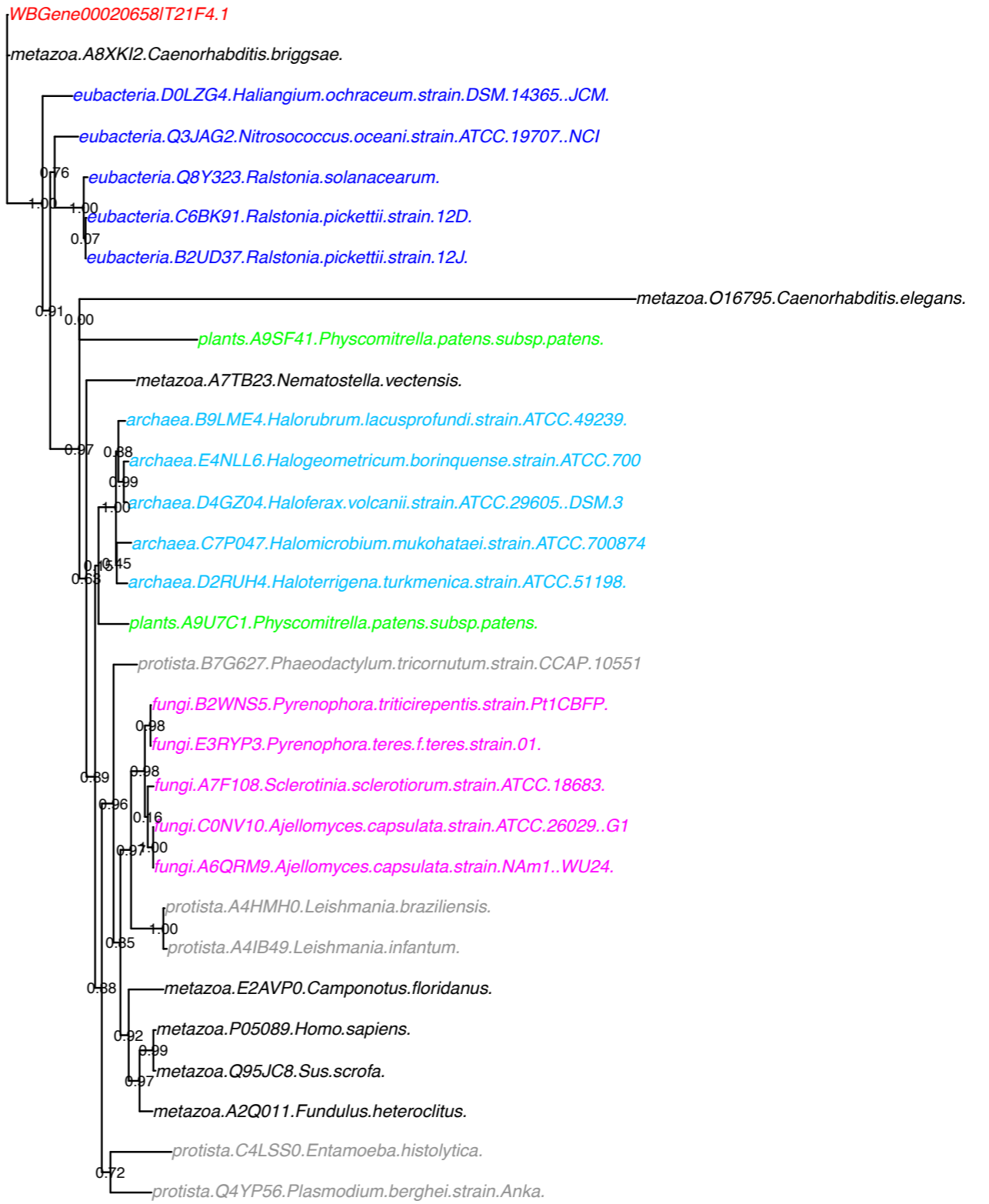

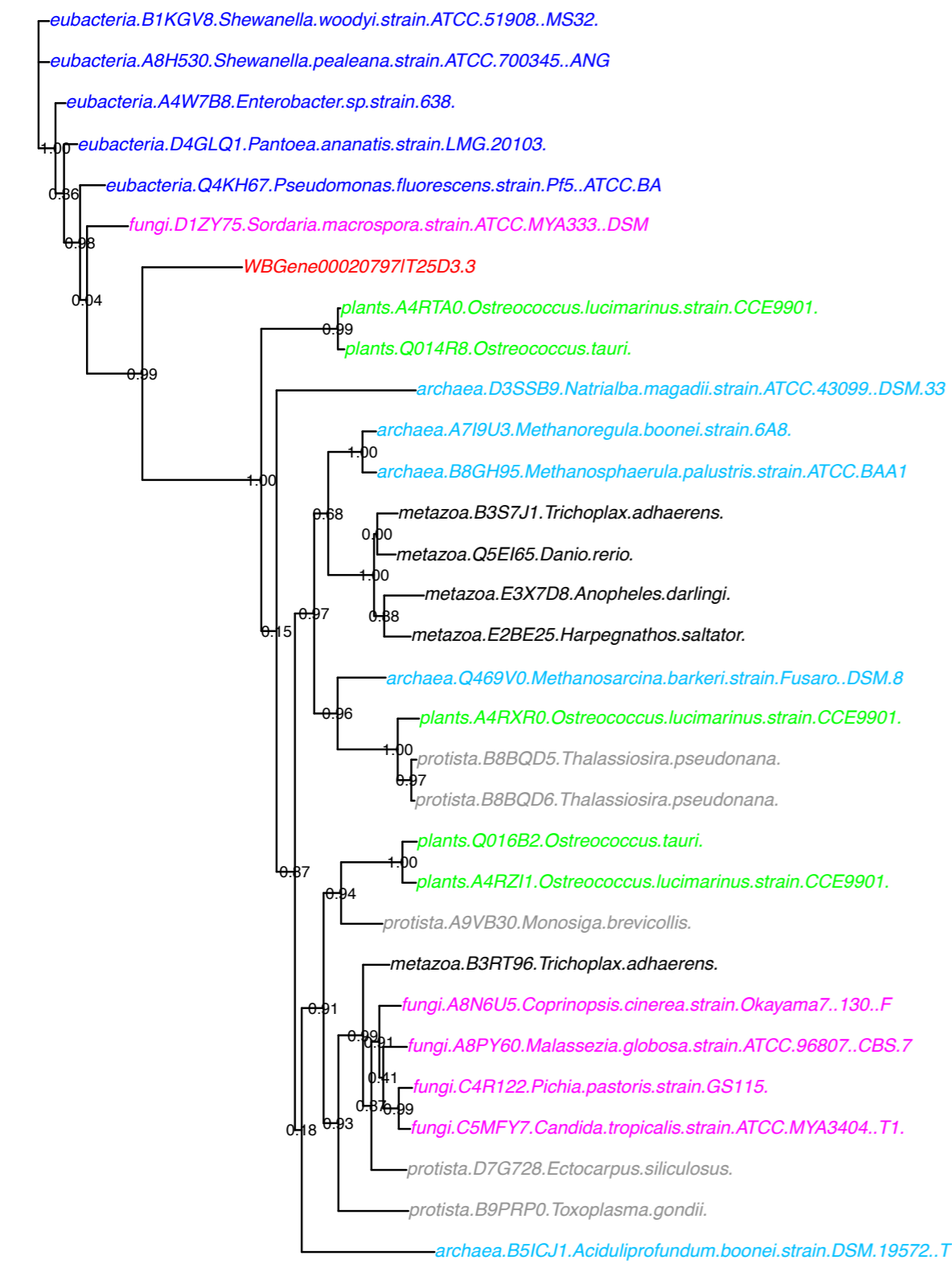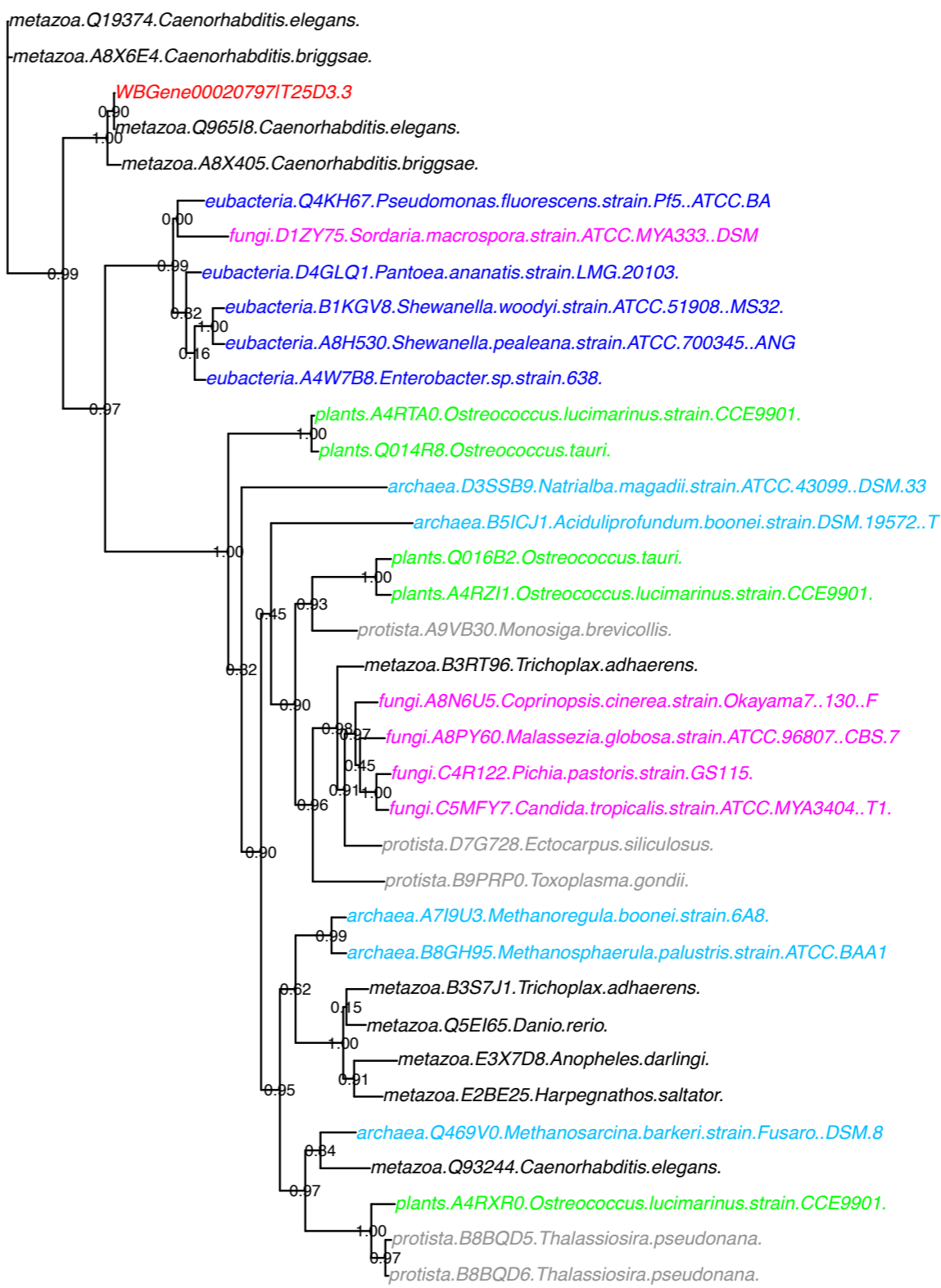

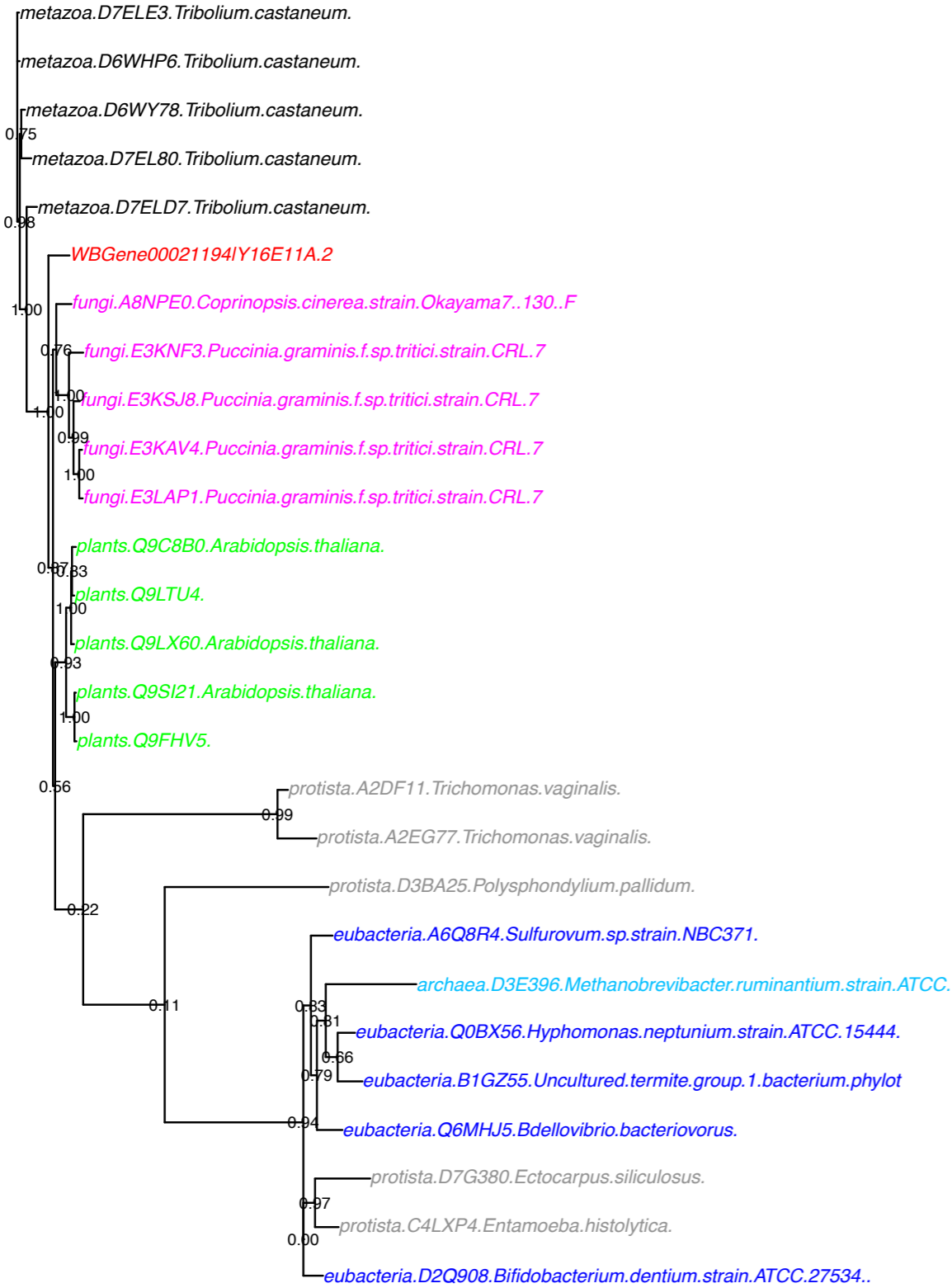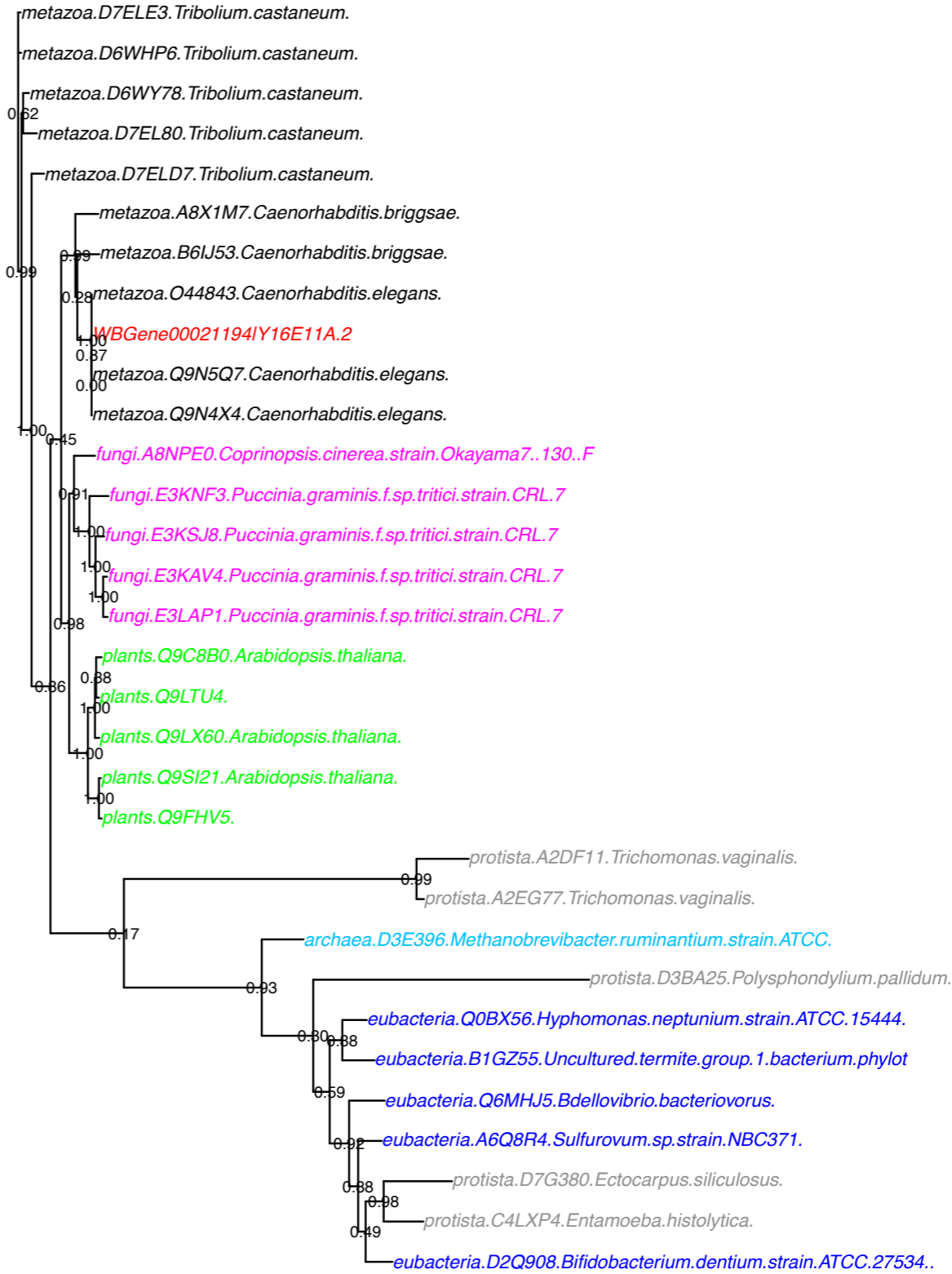

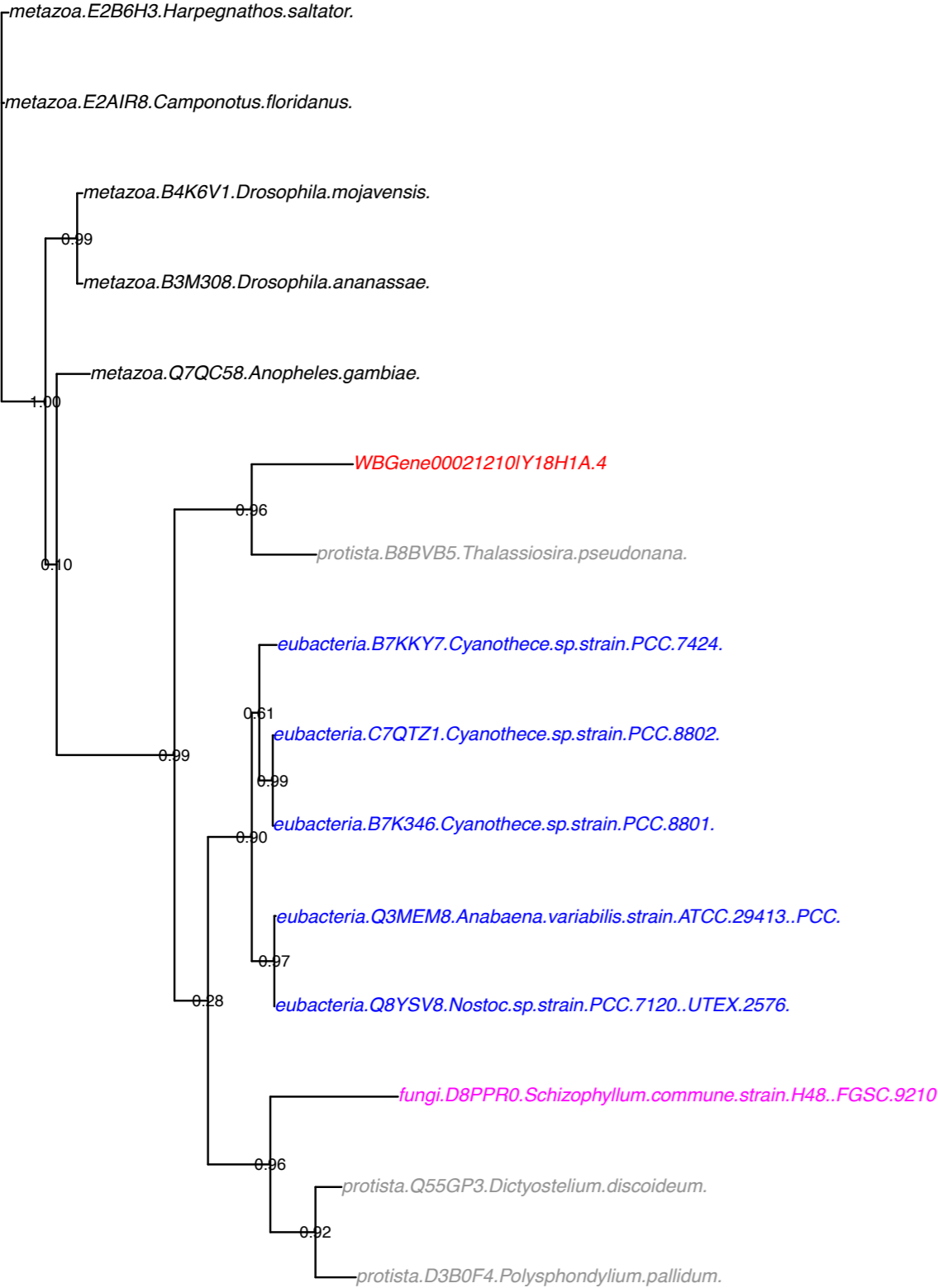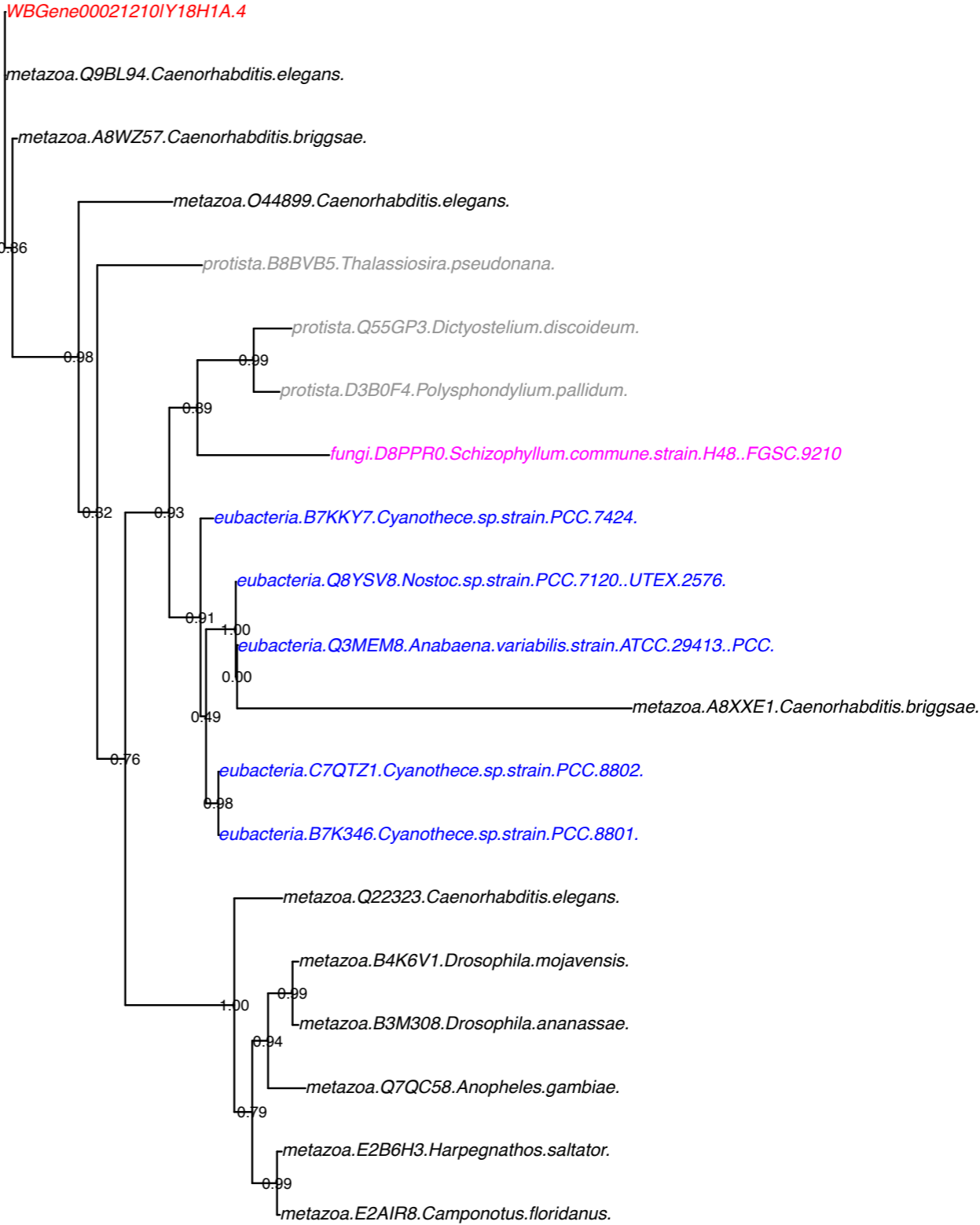

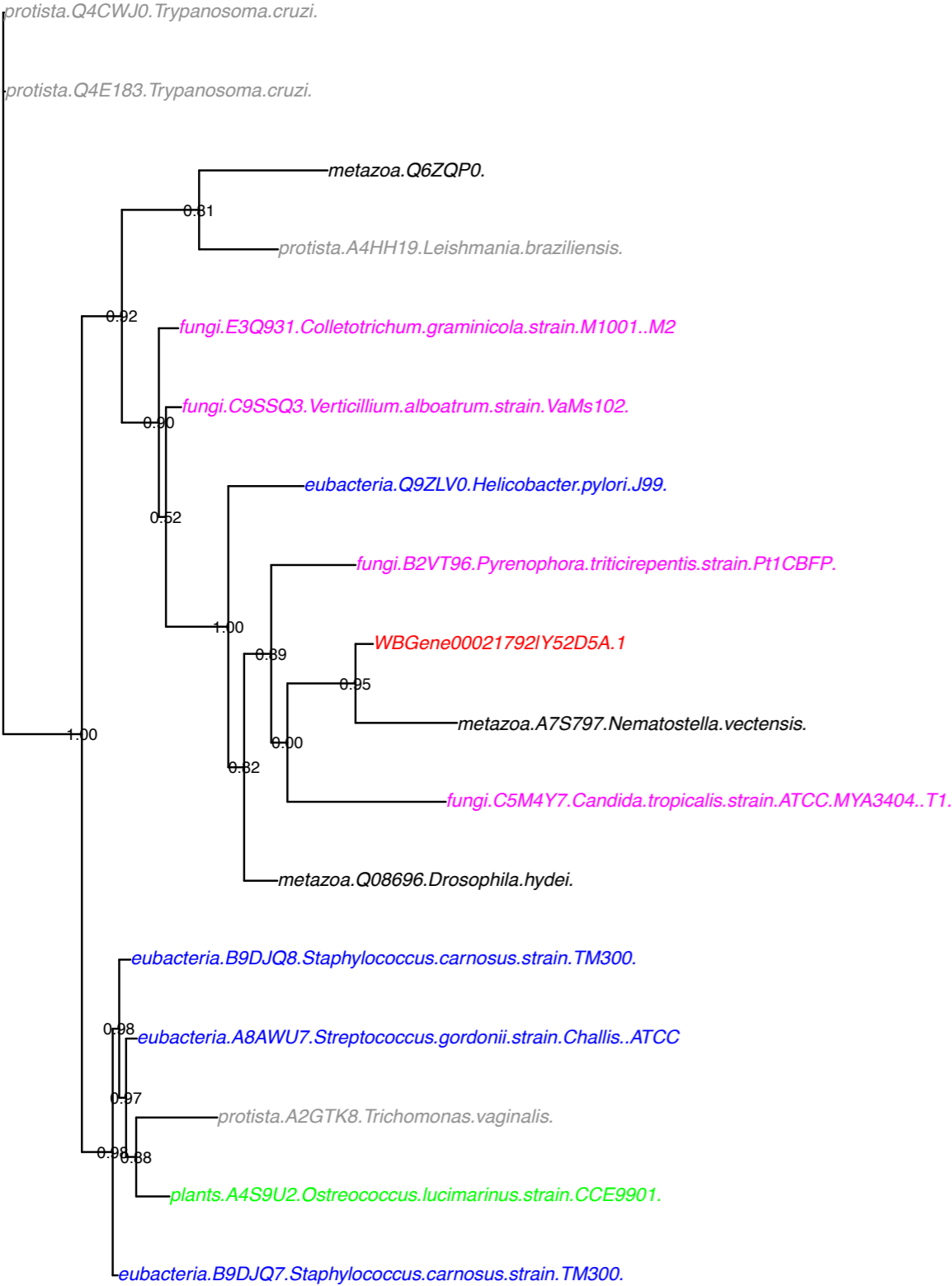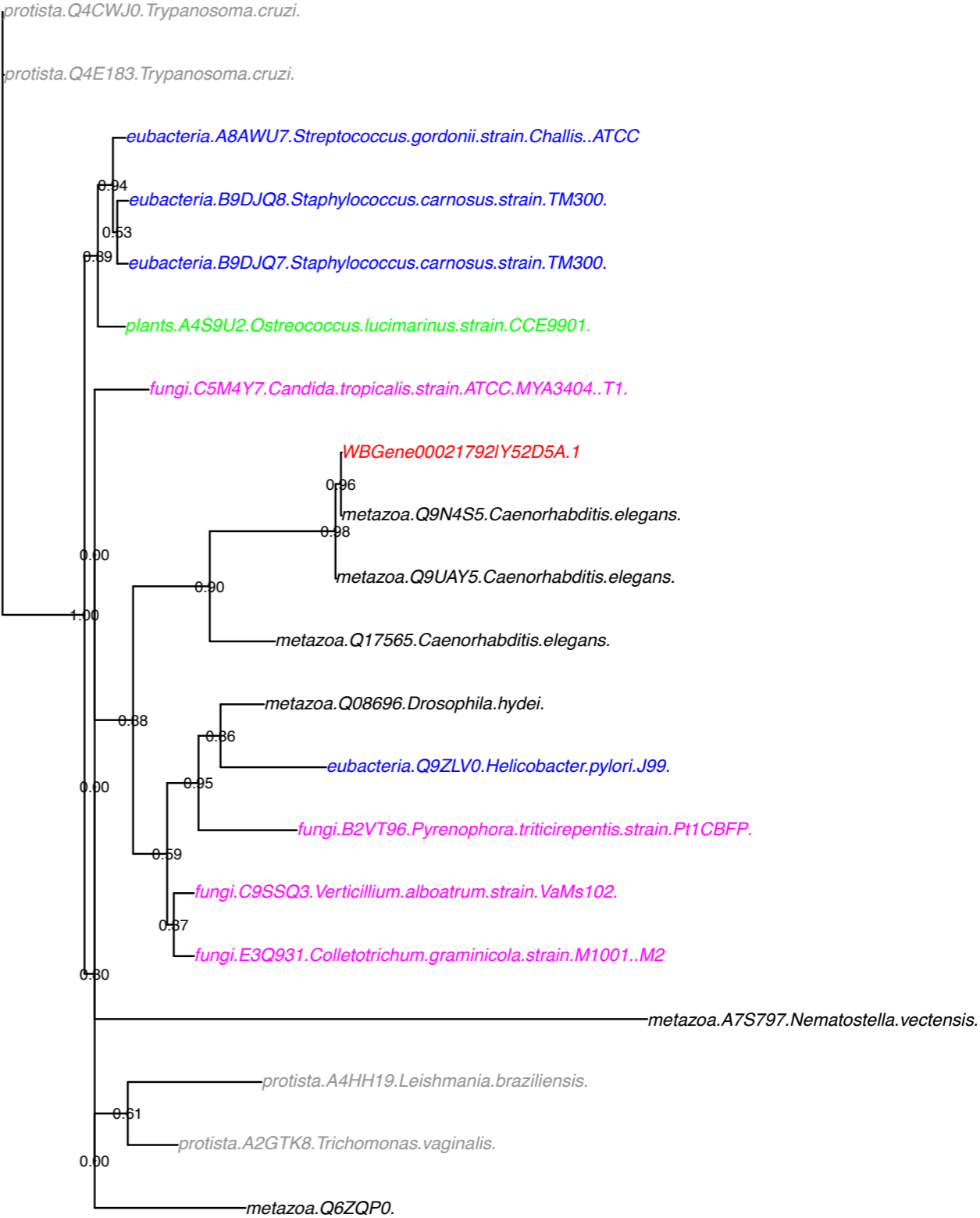

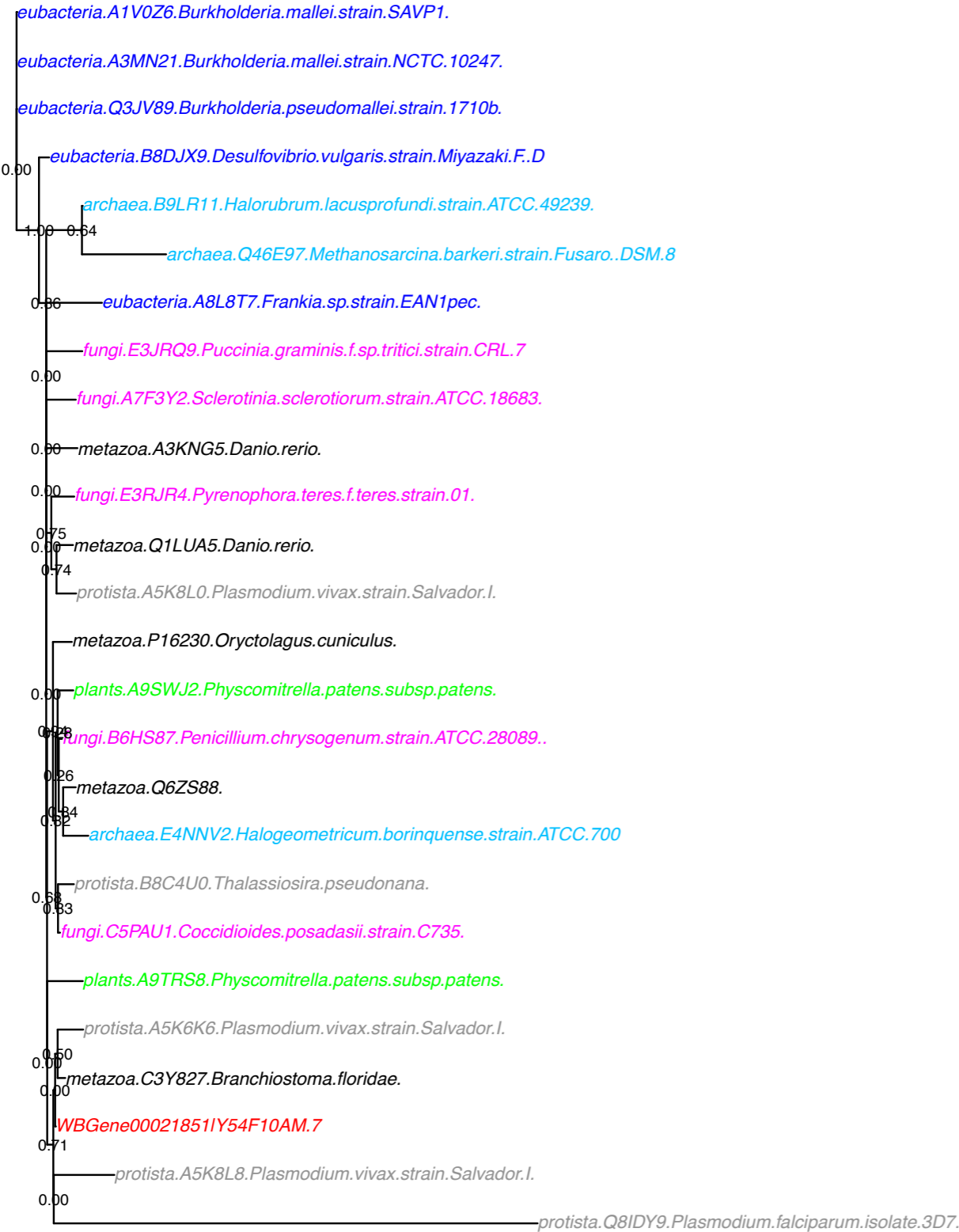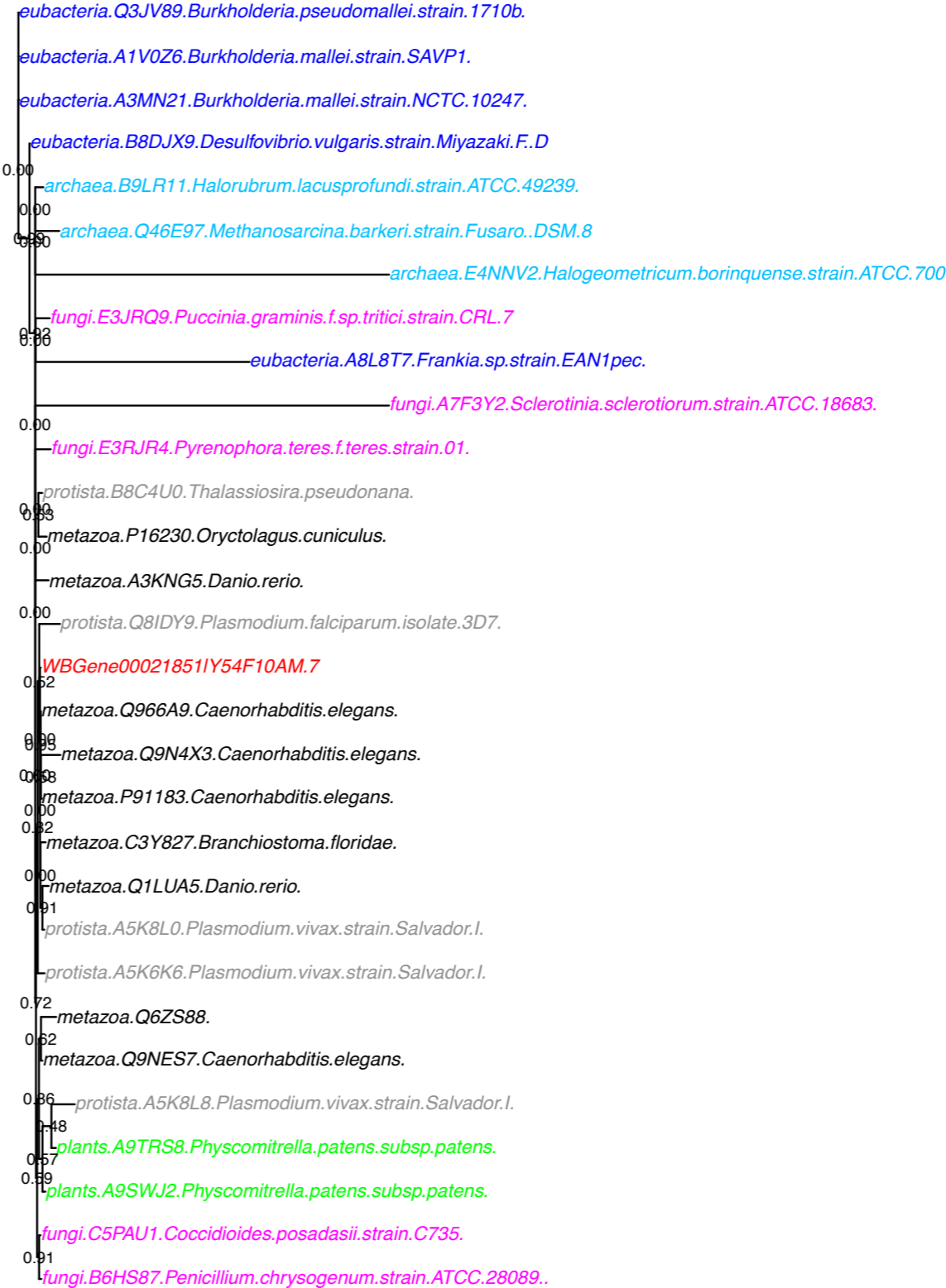

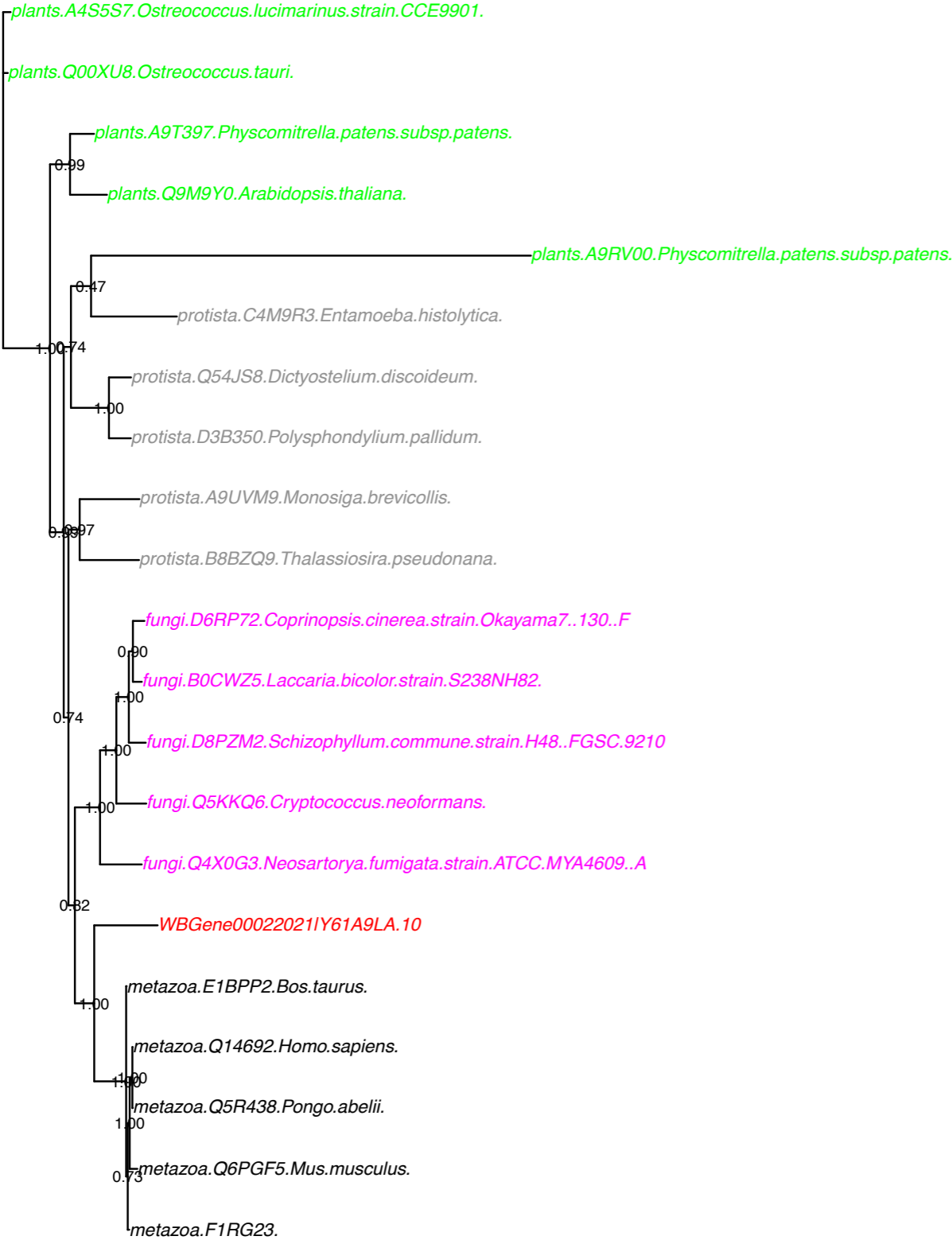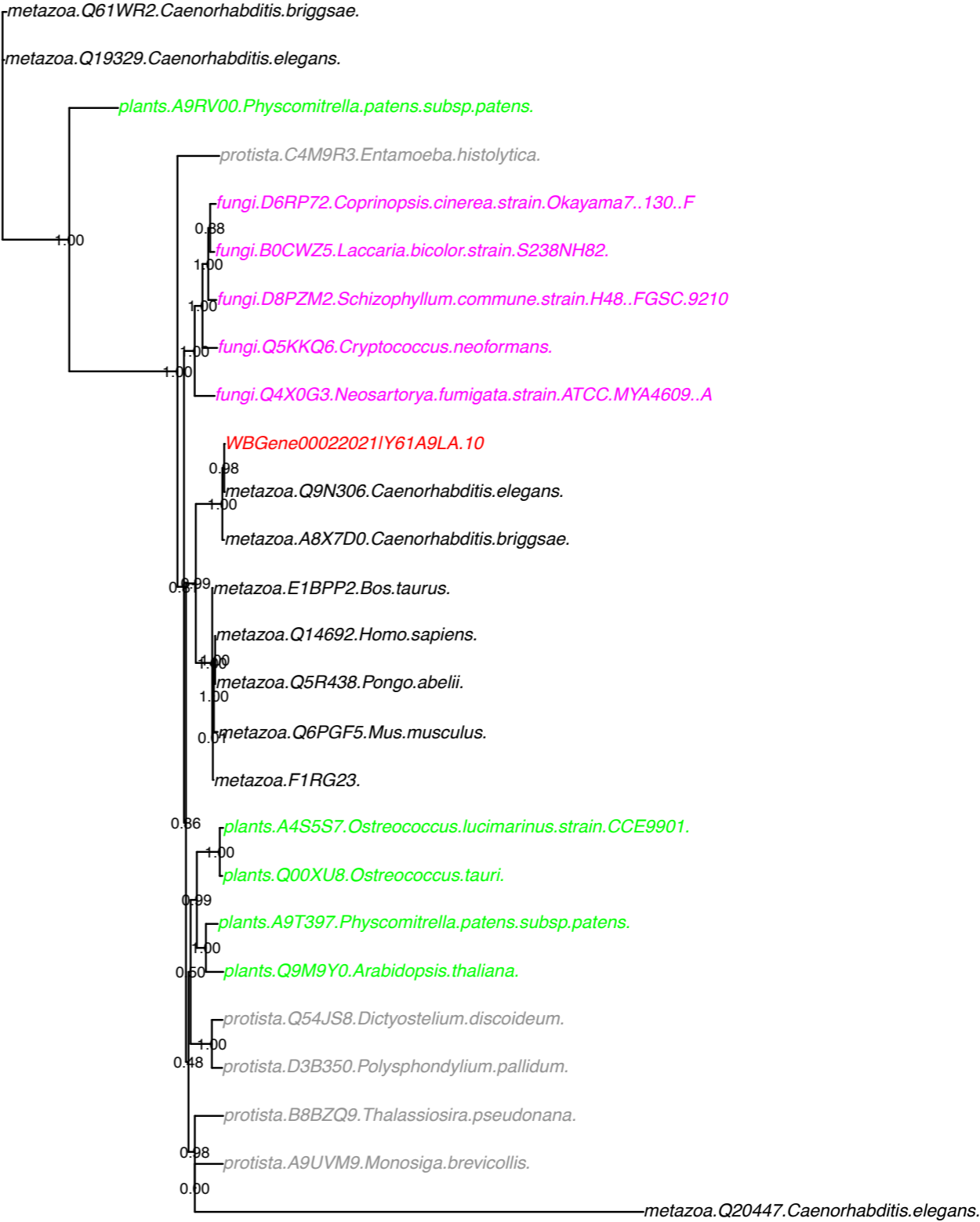

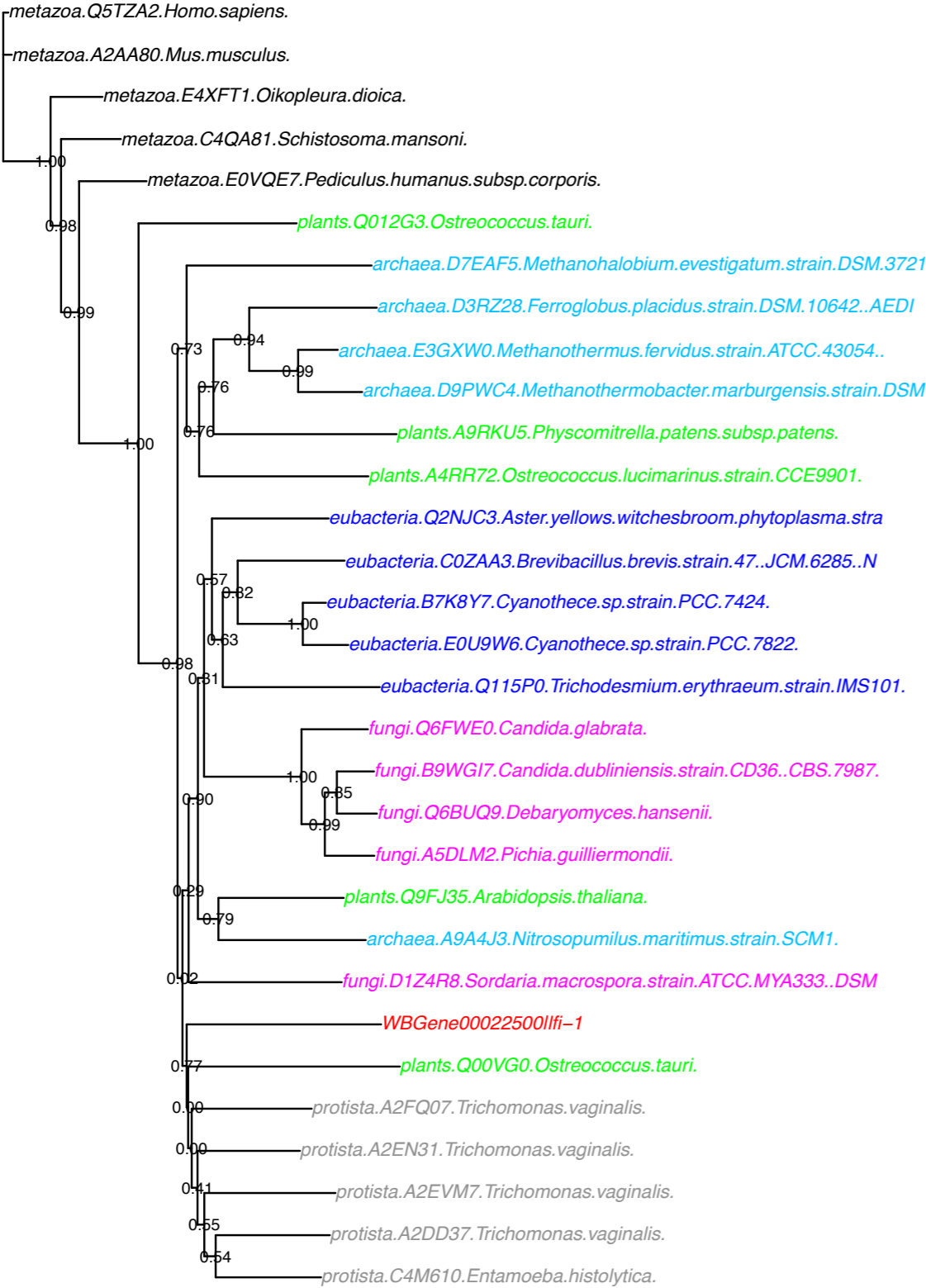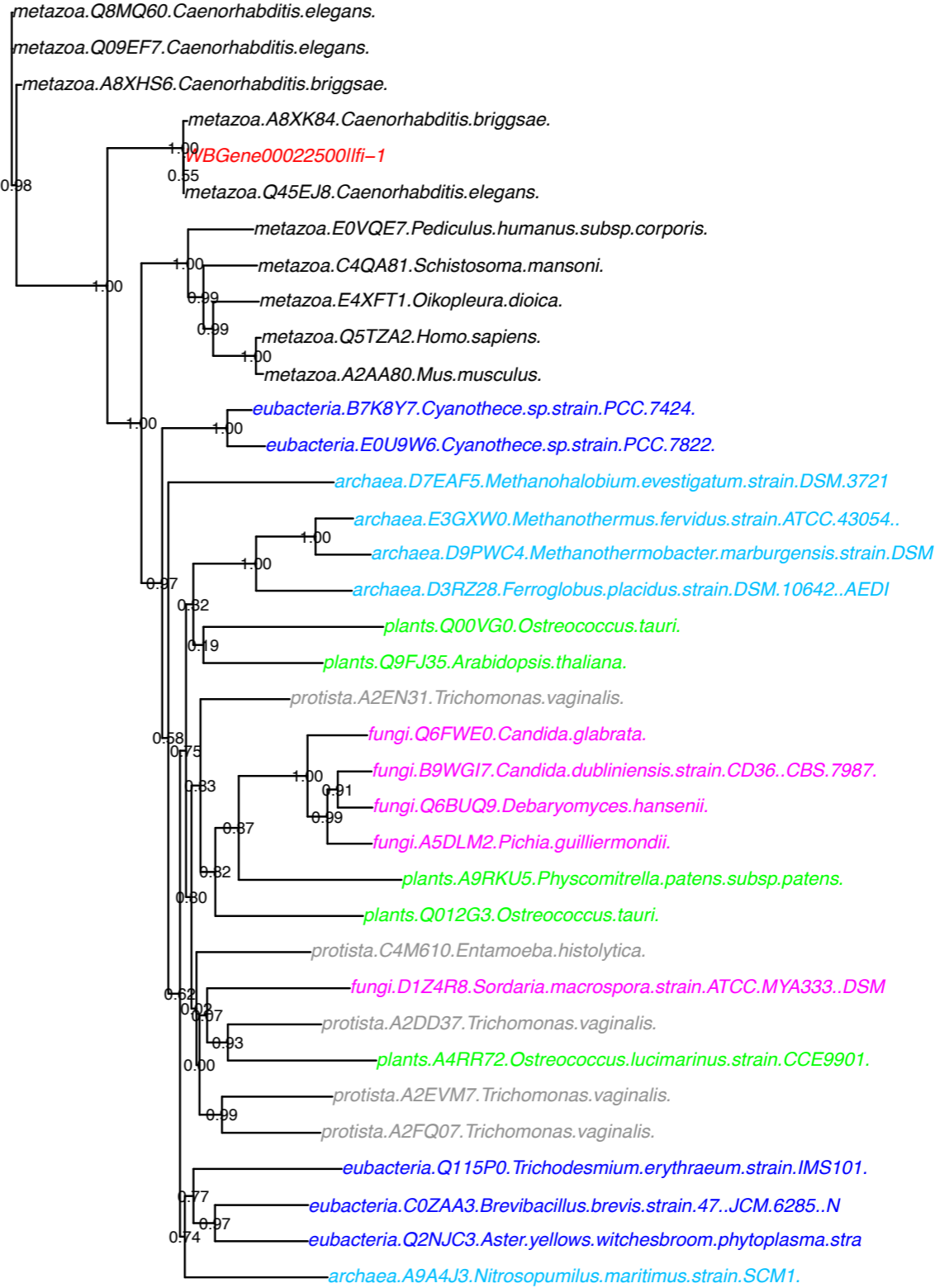

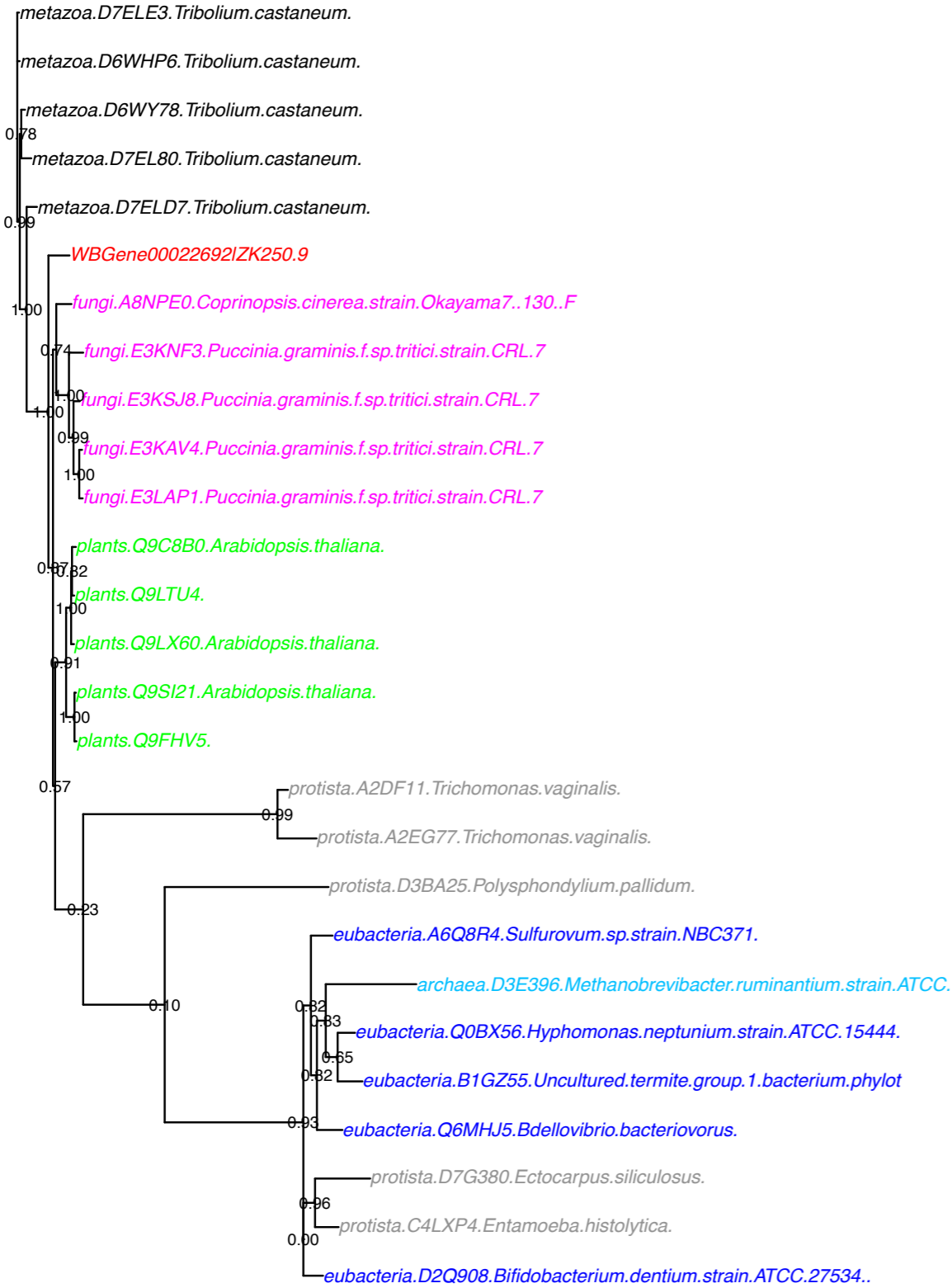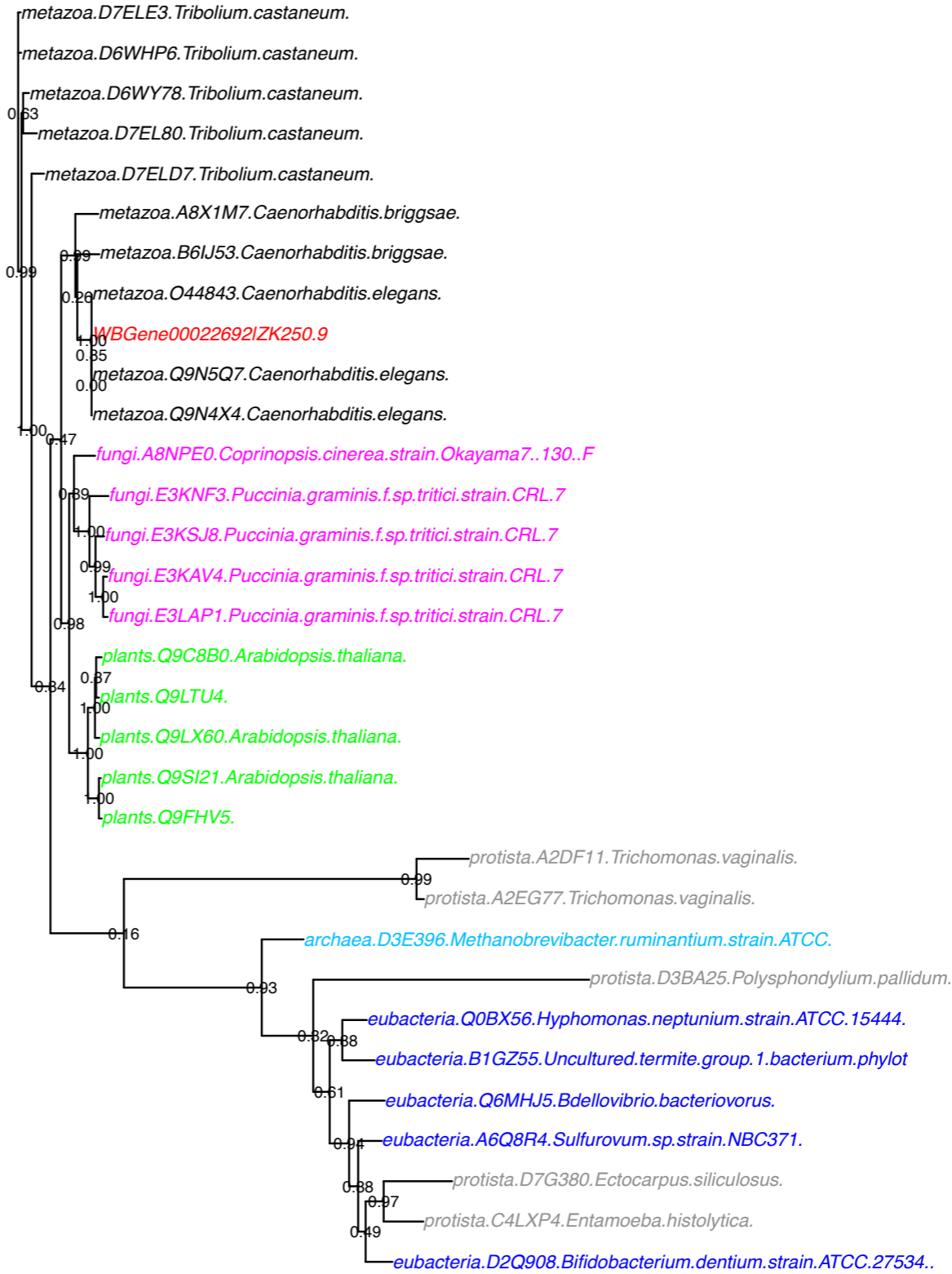

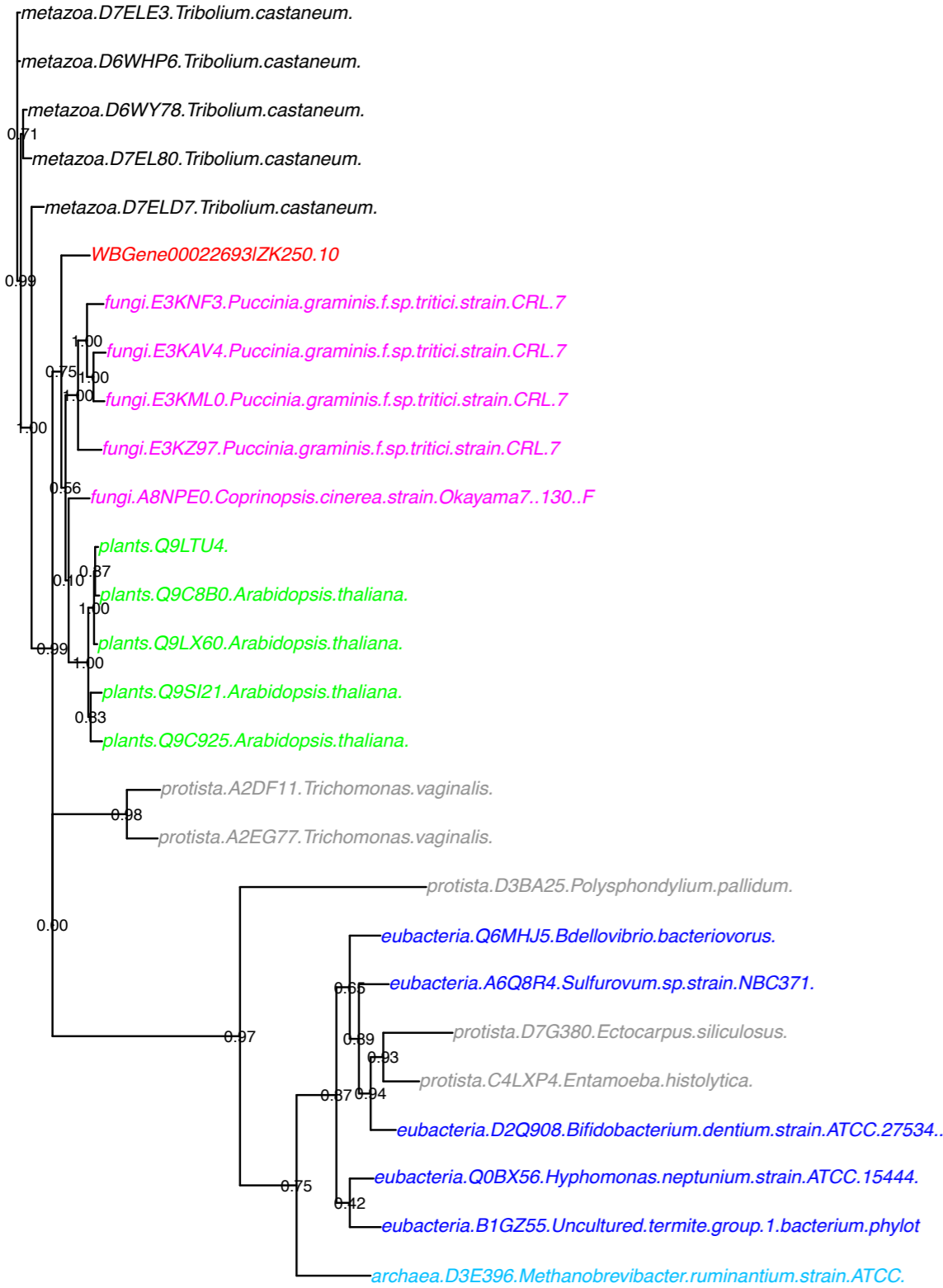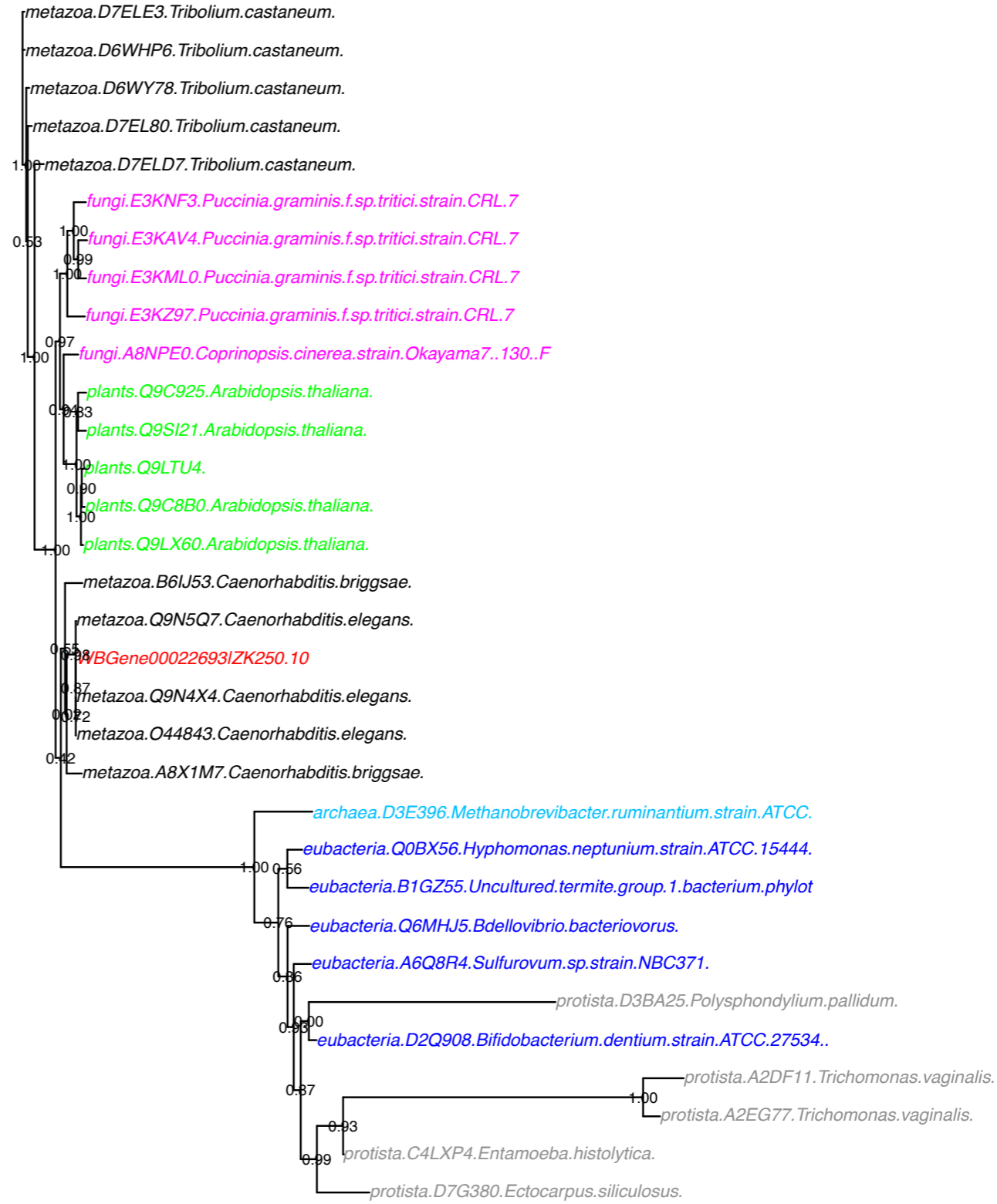

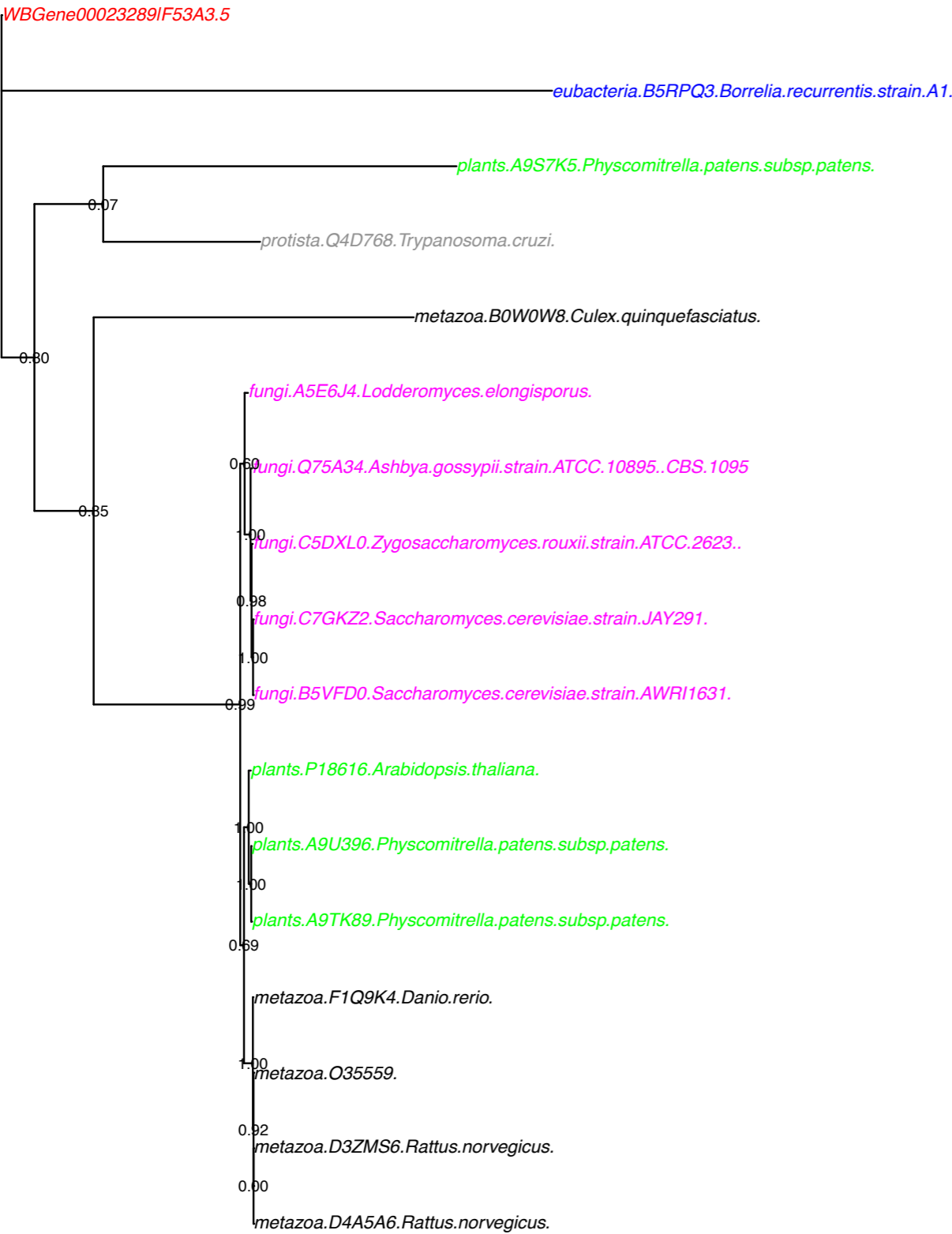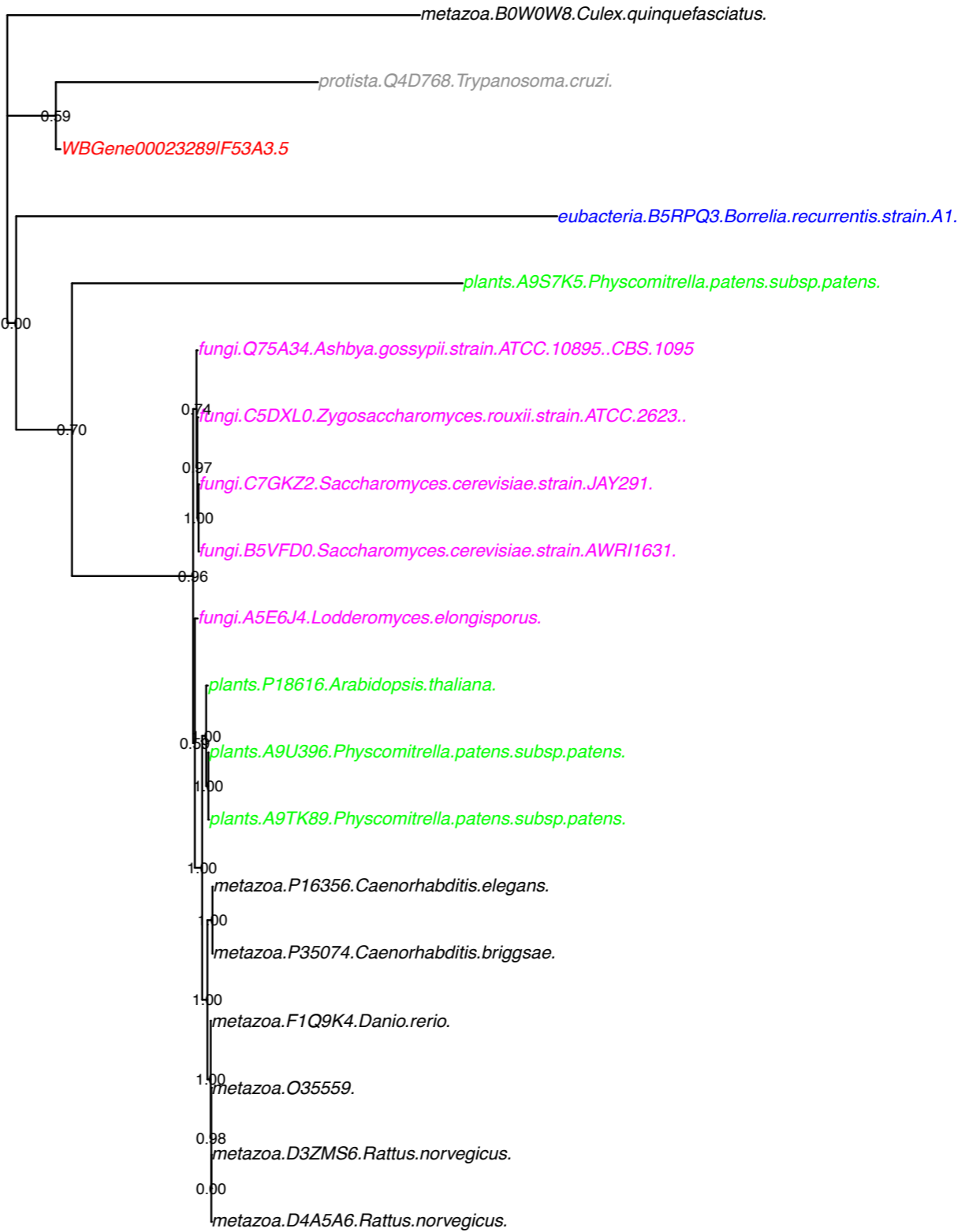

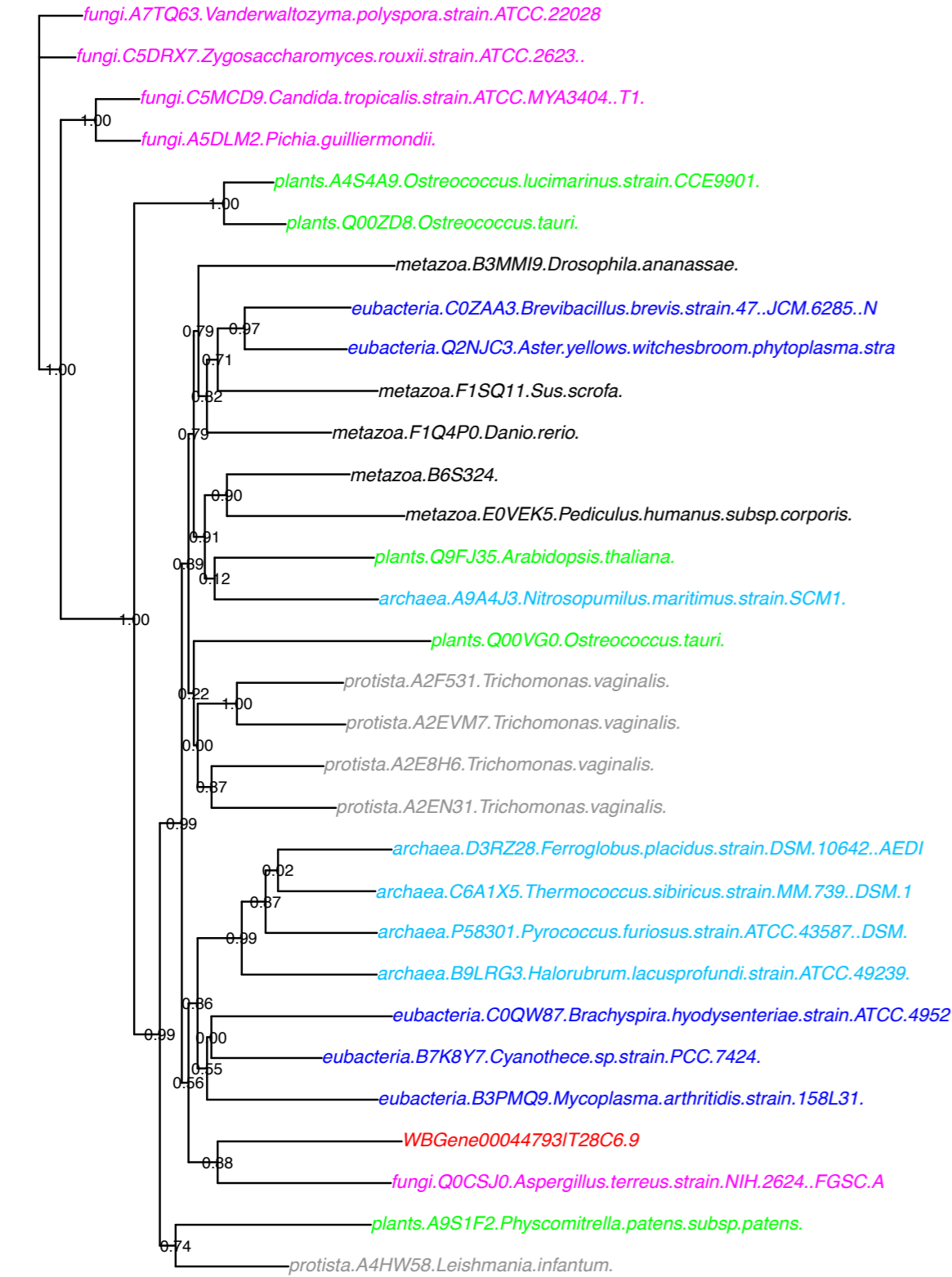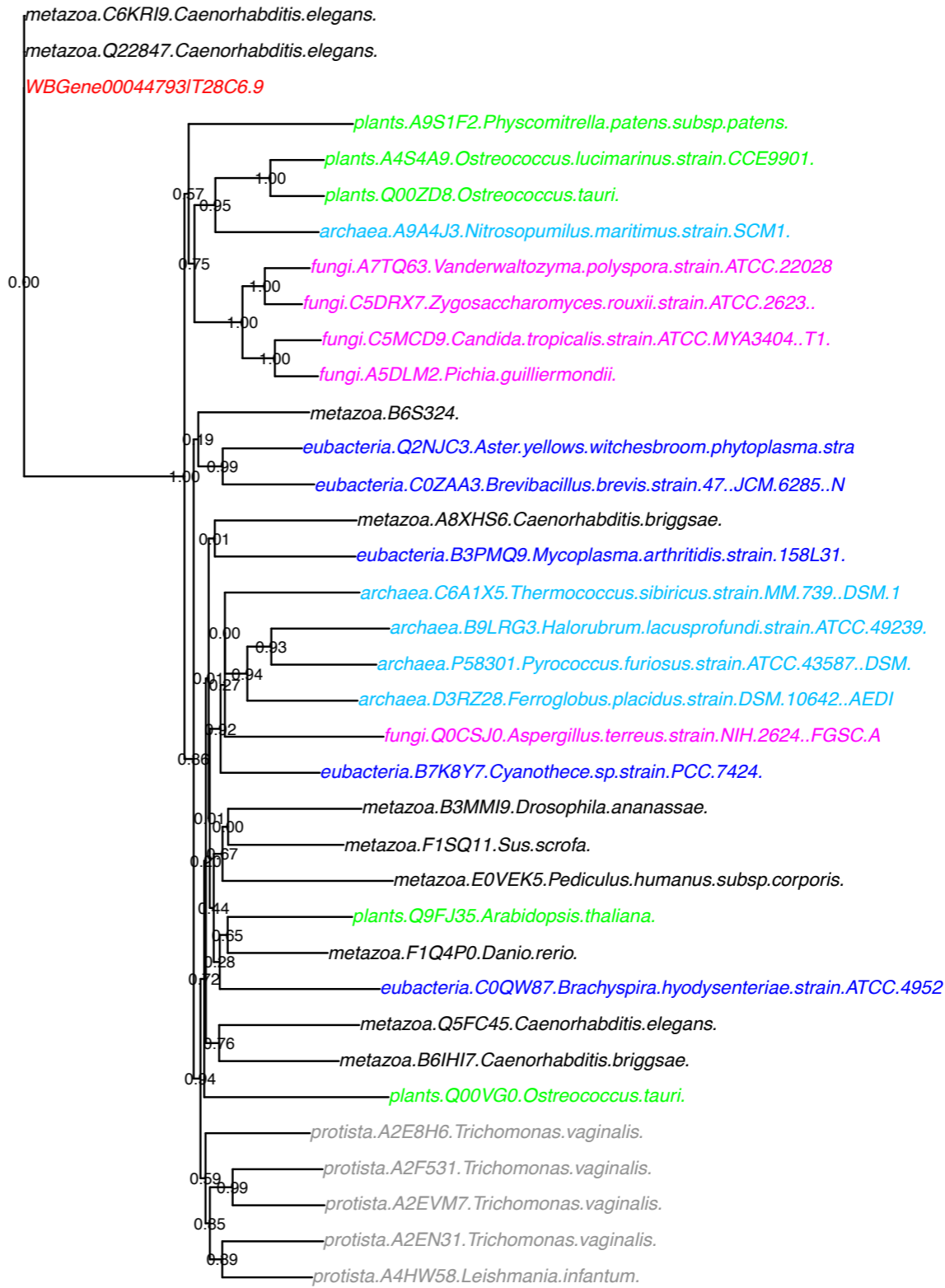

Supplement: Figure S3 — Phylogenetic trees for the 98 C. elegans transcripts with h≥30 and significant blast matches to metazoan sequences. Colour coding as in Figure S2: the nematode sequence under analysis is represented in red; metazoa, black; eubacteria, blue; archaea, light blue; fungi, pink; protists, grey. For each example, two phylogenetic trees are shown: the left-hand tree is constructed without nematode sequences other than the C. elegans test sequence, while the right-hand tree also includes the top five nematode matches. (PDF) [file pgen.1003035.s003.pdf]

Boschetti Fig S6

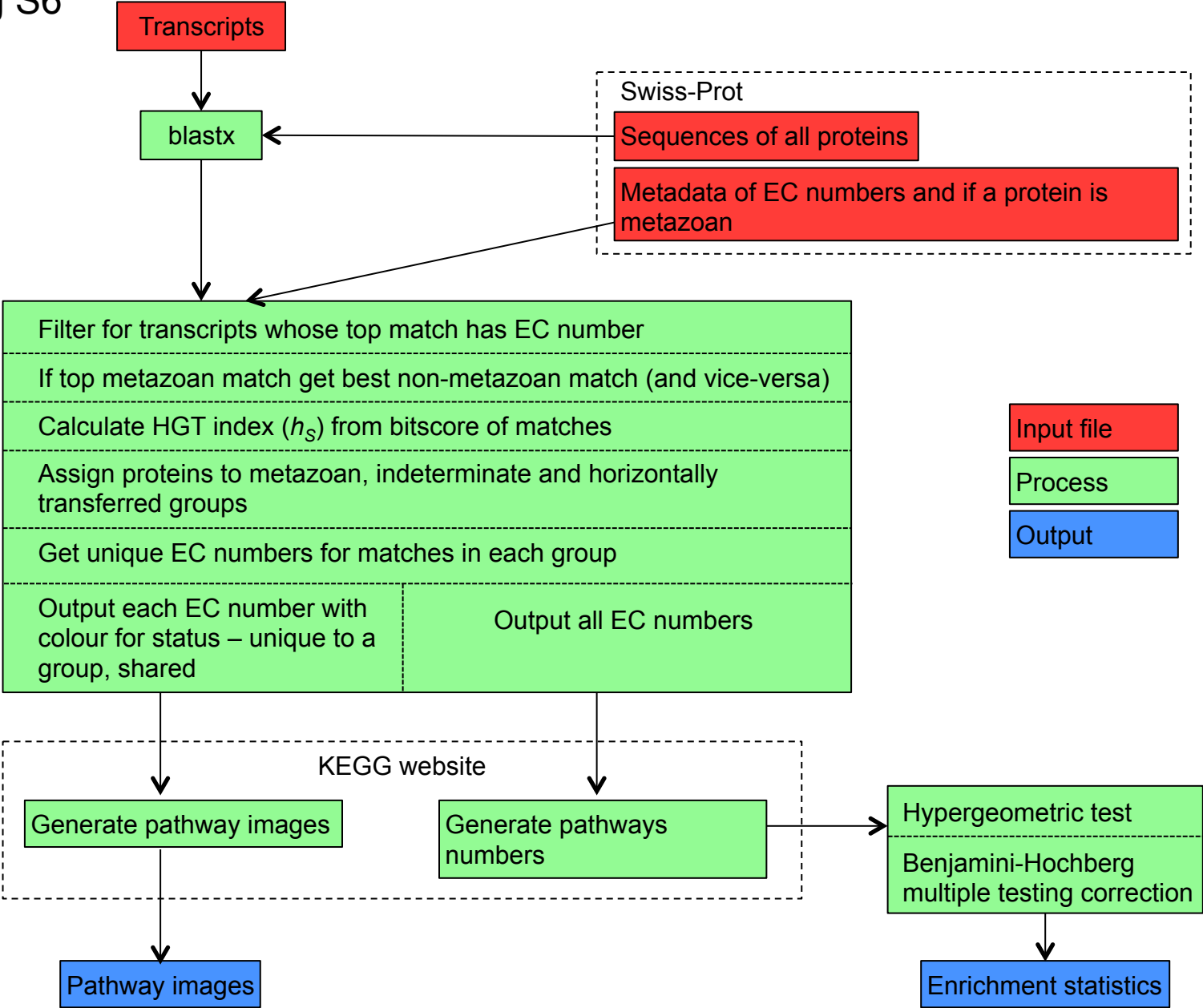

Supplement: Figure S6 — Workflow for determination of the number of horizontally transferred enzymes in each biochemical pathway. Rotifer transcripts were compared with blastx against Swiss-Prot and enzyme-matching transcripts were selected; the HGT index (hS) was recalculated and every enzyme (EC number) was assigned a colour according to it being represented by sequences that are purely metazoan (green), purely foreign (red), indeterminate (grey) or a combination (pink, red plus grey; orange, red plus green; or pale green, green plus grey). The outputs of these calculations were used to colour code enzymes in KEGG pathways and to calculate statistics for the over-representation of HGT genes in each pathway. (PDF) [file pgen.1003035.s006.pdf]
