# Supplementary material for: Biochemical Diversification through Foreign Gene Expression in Bdelloid Rotifers
Source: PLoS Genet. 2012 Nov 15;8(11):e1003035. doi: 10.1371/journal.pgen.1003035 (PMC3499245; doi:10.1371/journal.pgen.1003035)
Supplement: Figure S2 — Phylogenetic trees for a selection of A. ricciae transcript contigs. Colour coding: the bdelloid sequence under analysis is represented in red; metazoa, black; eubacteria, blue; archaea, light blue; fungi, pink; protists, grey. Figure 2A is shown again as (C) here to allow comparison in the same format. Panels G–M represent those examples from Figure 3 where it is meaningful to construct a tree (i.e. there are significant matches with metazoan counterparts). (PDF) [file pgen.1003035.s002.pdf]

Boschetti Figure S2

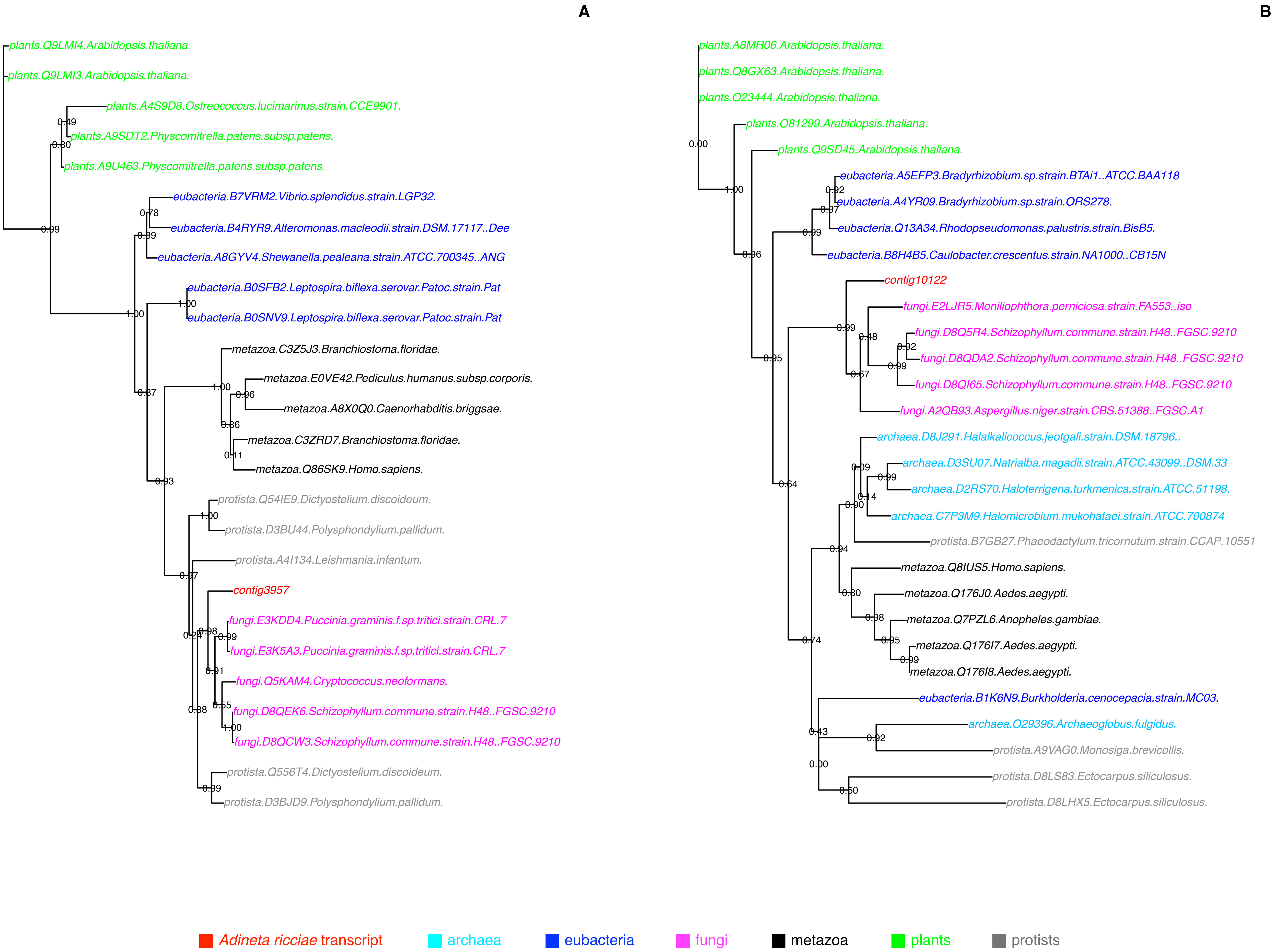

Thursday, 21 June 2012

Boschetti Figure S1

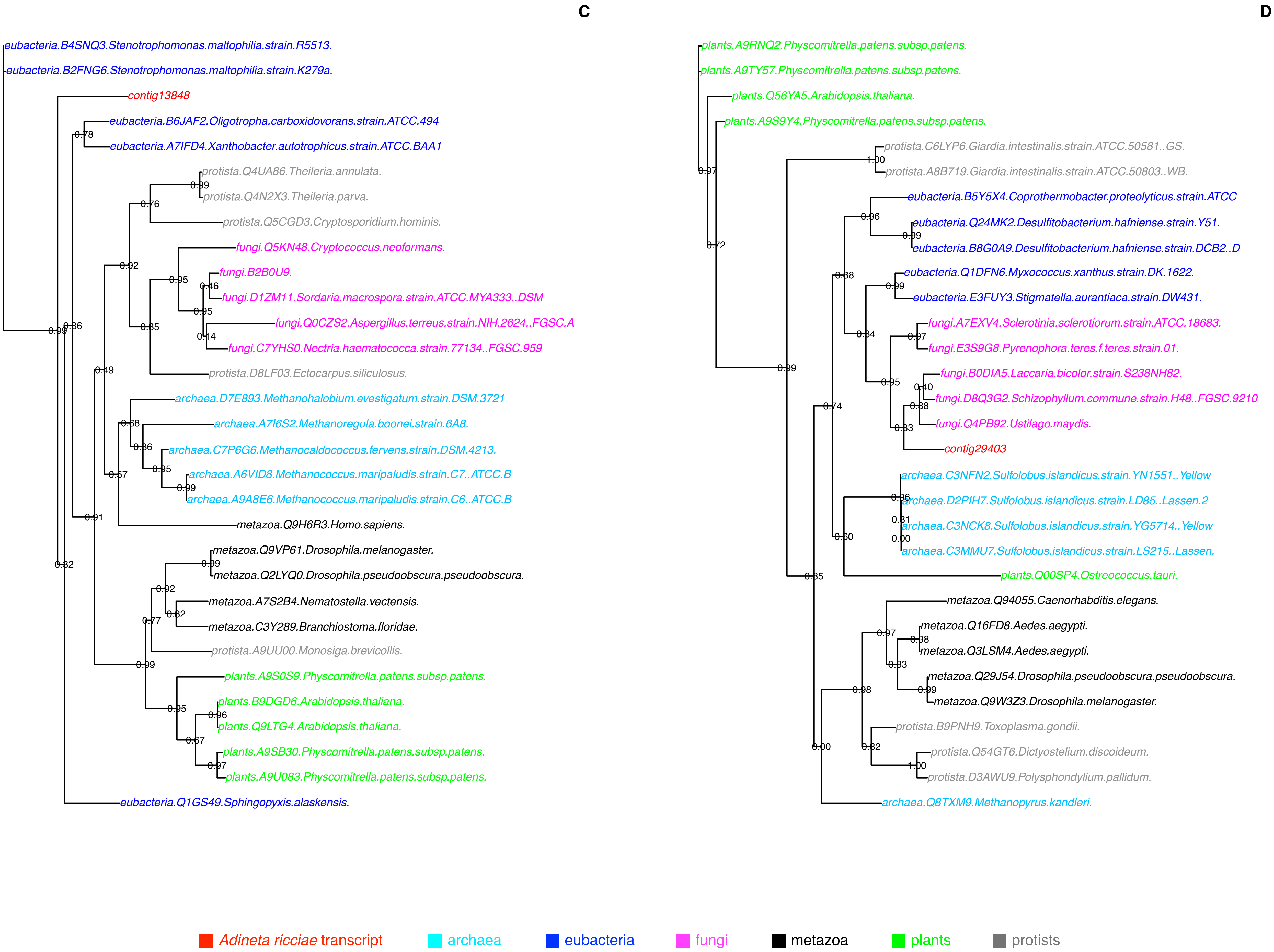

Boschetti Figure S1

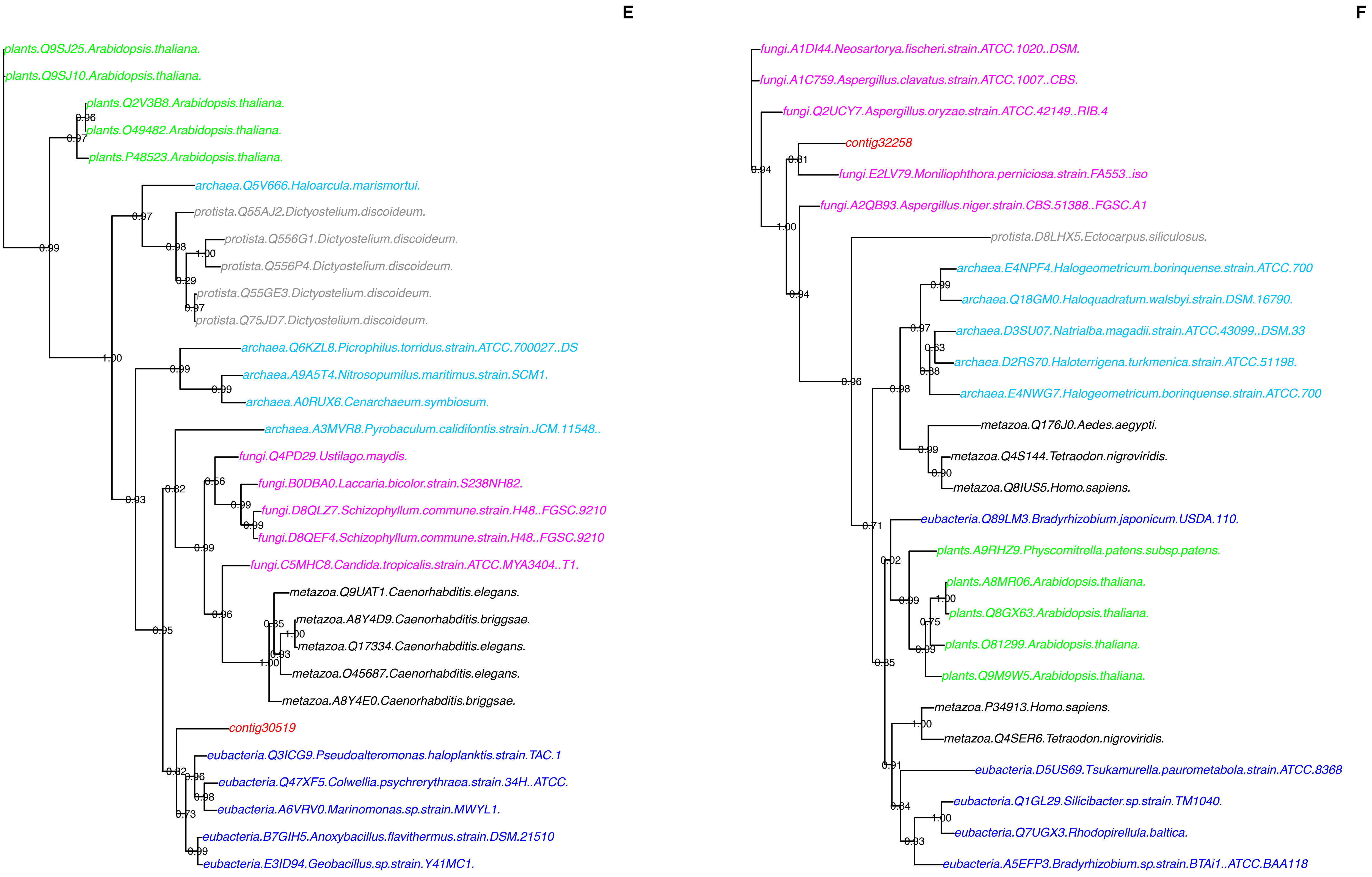

Adineta ricciae transcript   archaea   eubacteria   fungi   metazoa   plants   protists

Thursday, 21 June 2012

Boschetti Figure S1

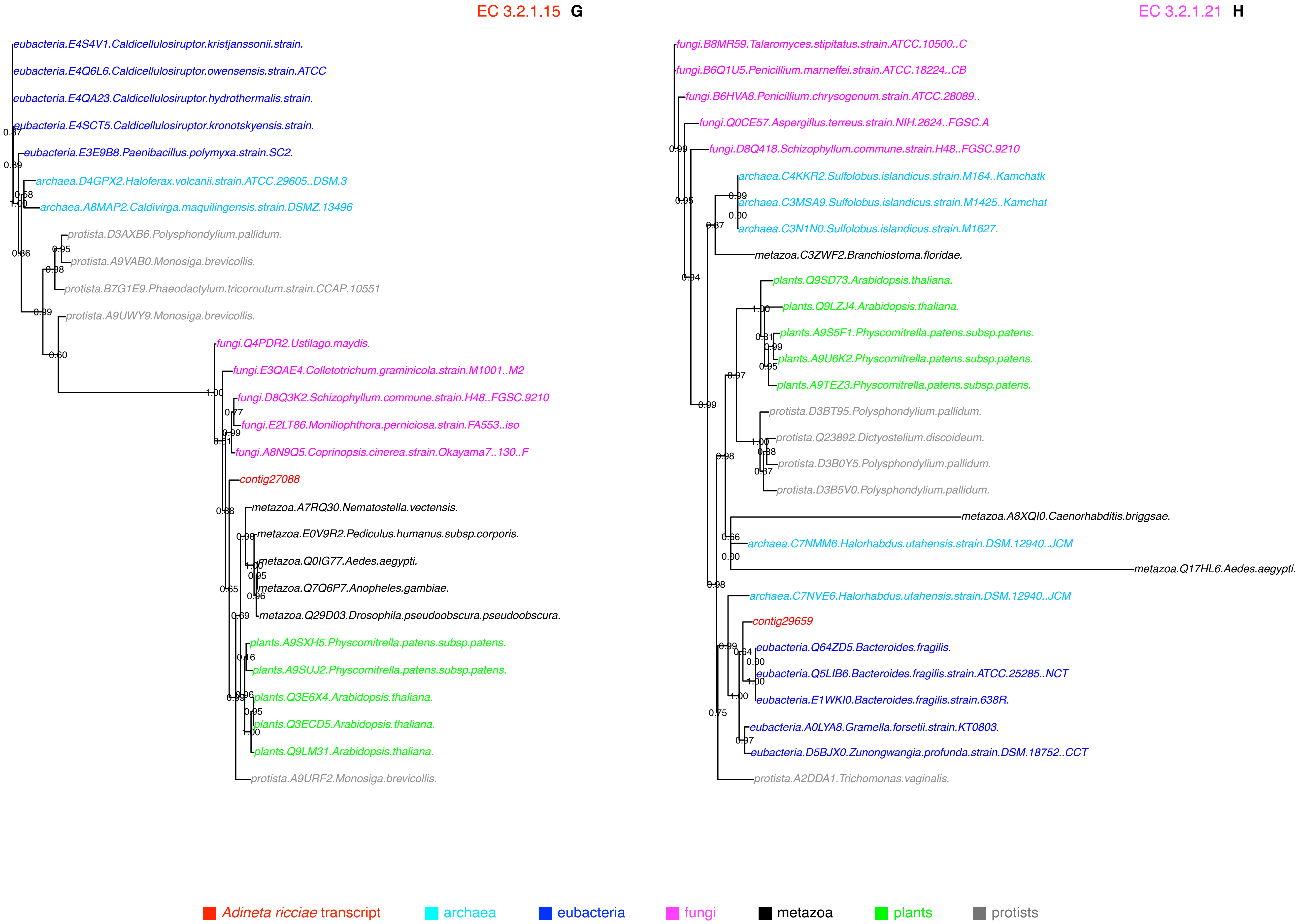

Boschetti Figure S1

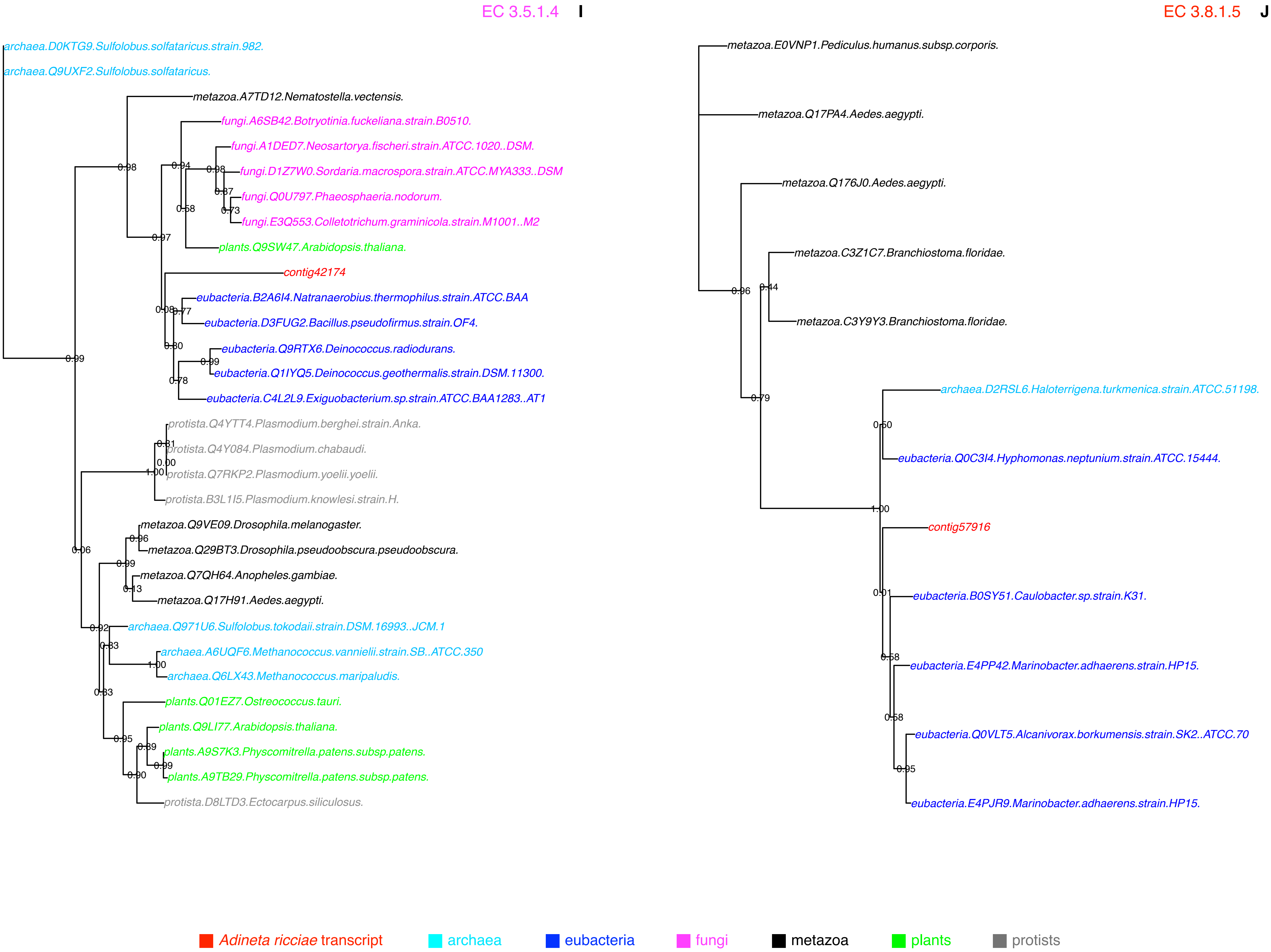

Thursday, 21 June 2012

Boschetti Figure S1

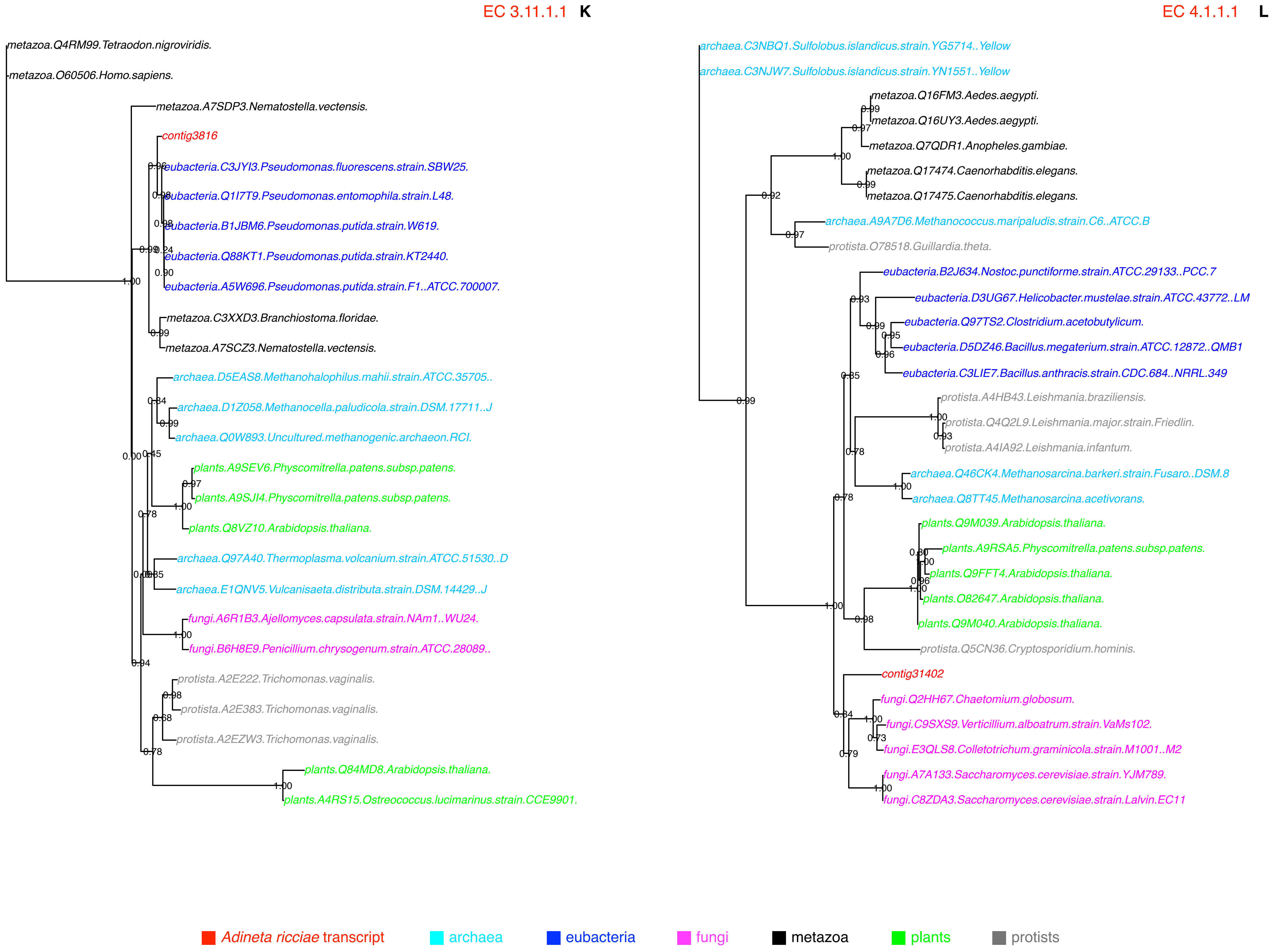

Boschetti Figure S1

EC 4.2.1.9 **M**

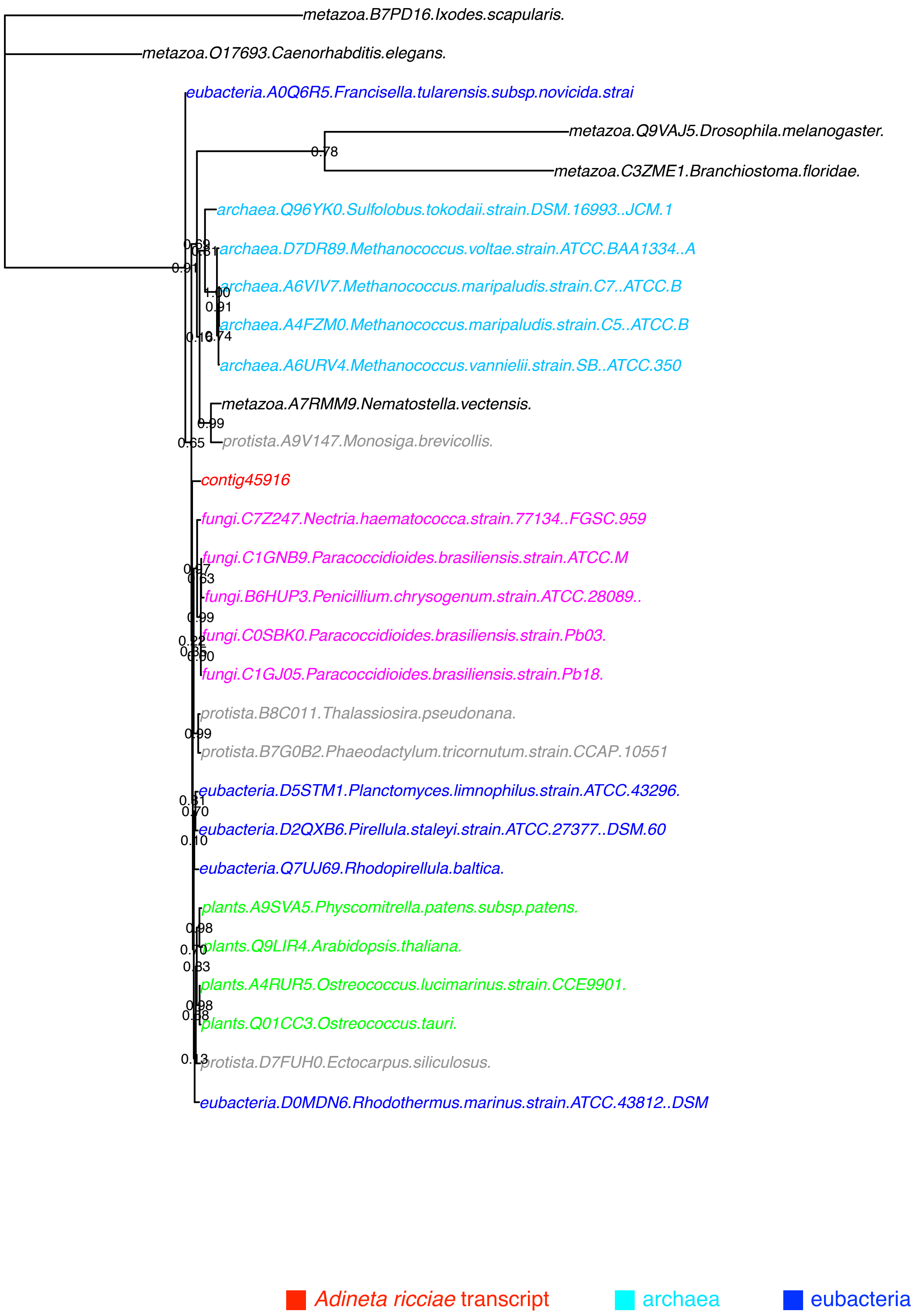

Thursday, 21 June 2012
