# Supplementary material for: Biochemical Diversification through Foreign Gene Expression in Bdelloid Rotifers
Source: PLoS Genet. 2012 Nov 15;8(11):e1003035. doi: 10.1371/journal.pgen.1003035 (PMC3499245; doi:10.1371/journal.pgen.1003035)

Boschetti Figure S4

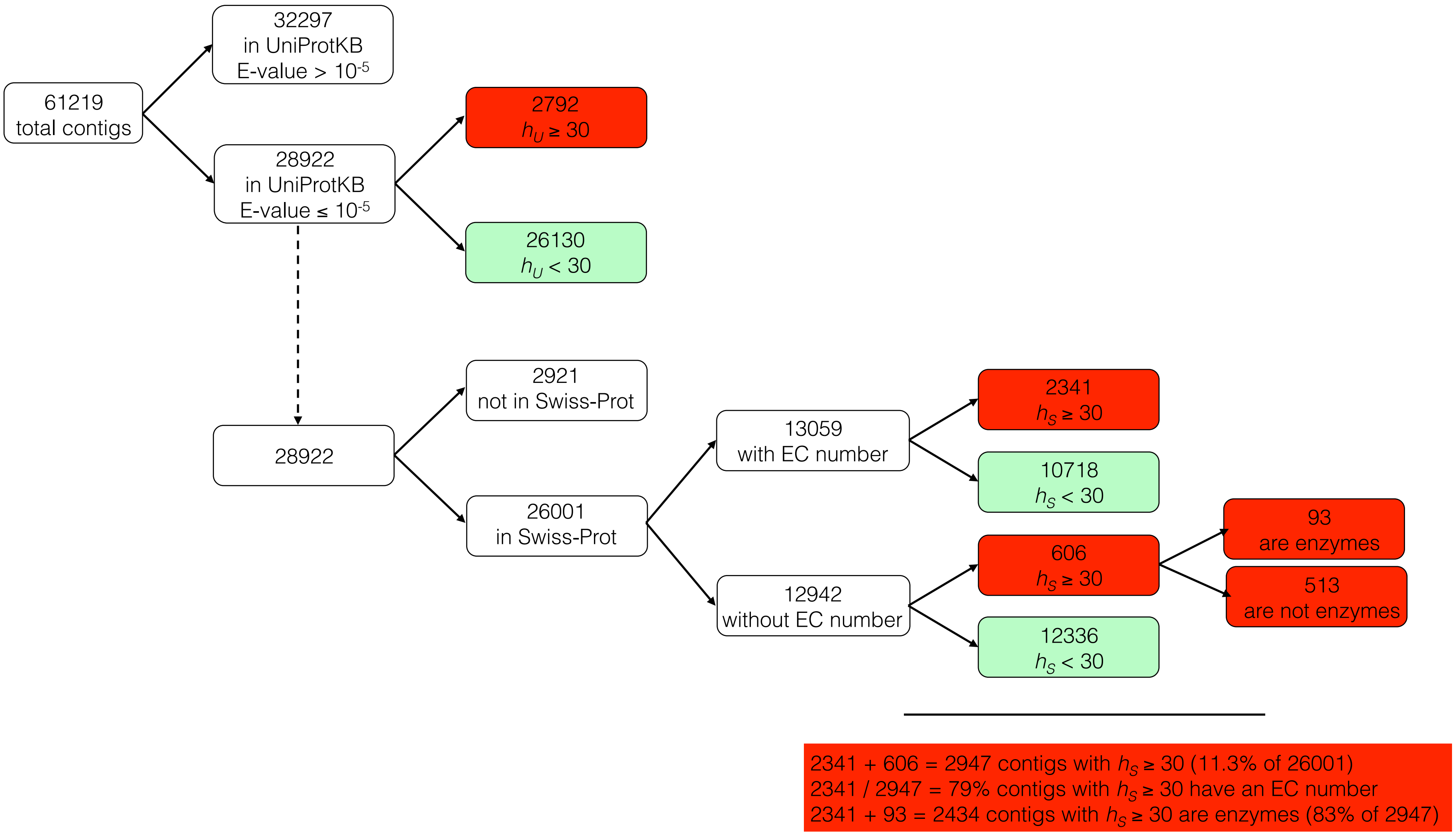

Supplement: Figure S4 — Summary of transcript assignment in UniProtKB or Swiss-Prot. Foreign contigs in red, indeterminate plus metazoan in pale green. (PDF) [file pgen.1003035.s004.pdf]
