# Supplementary material for: Biochemical Diversification through Foreign Gene Expression in Bdelloid Rotifers
Source: PLoS Genet. 2012 Nov 15;8(11):e1003035. doi: 10.1371/journal.pgen.1003035 (PMC3499245; doi:10.1371/journal.pgen.1003035)

Boschetti Figure S5A

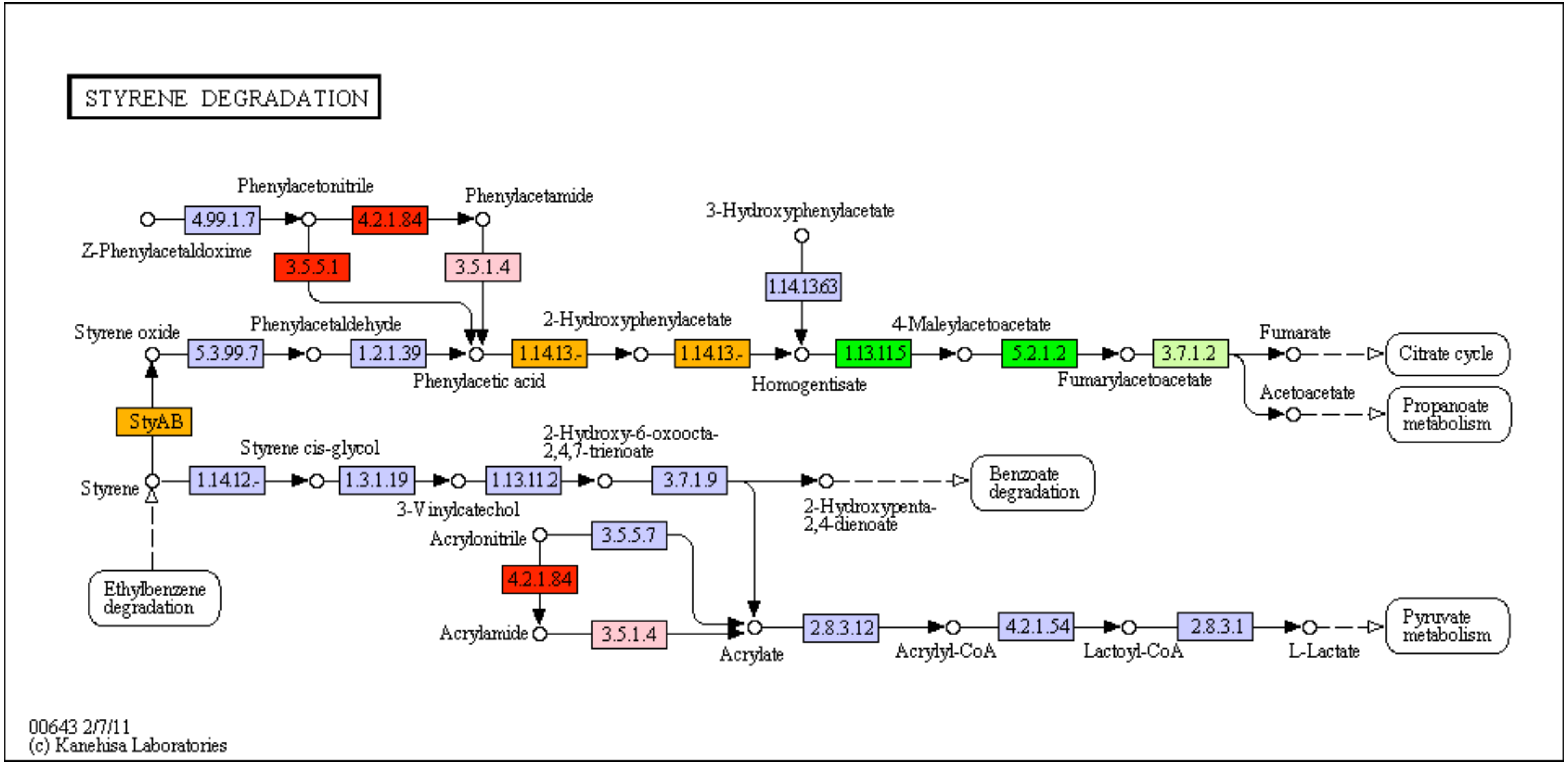

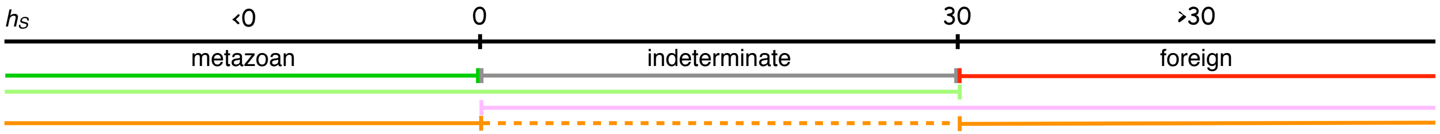

Boschetti Figure S5B

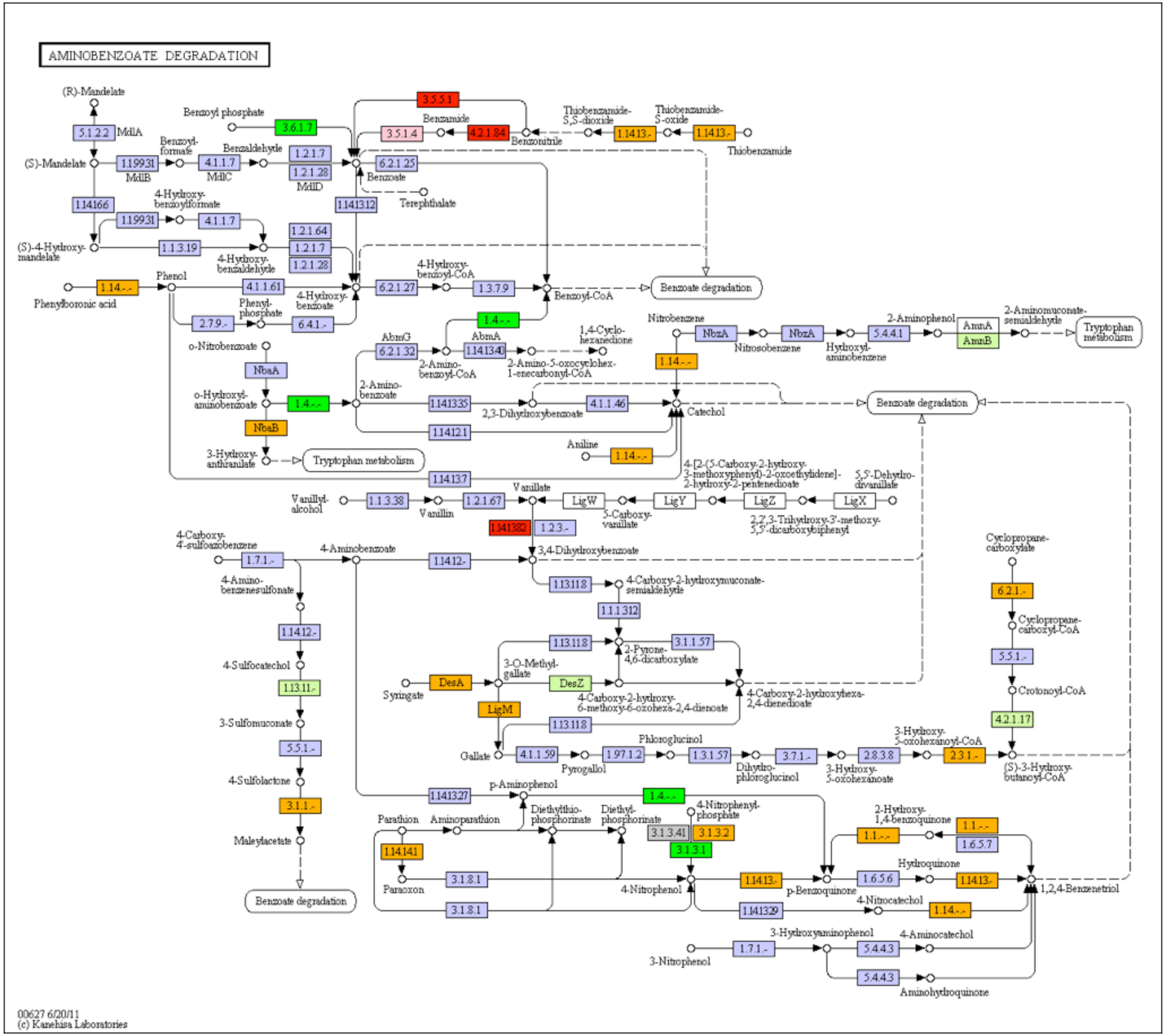

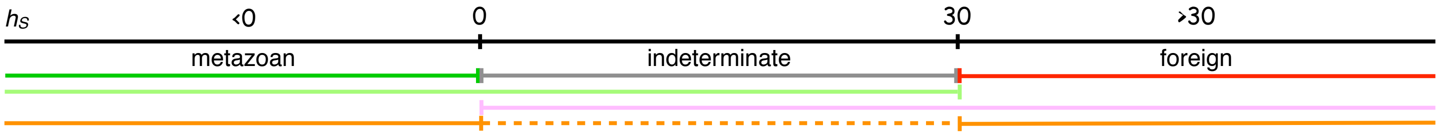

Boschetti Figure S5C

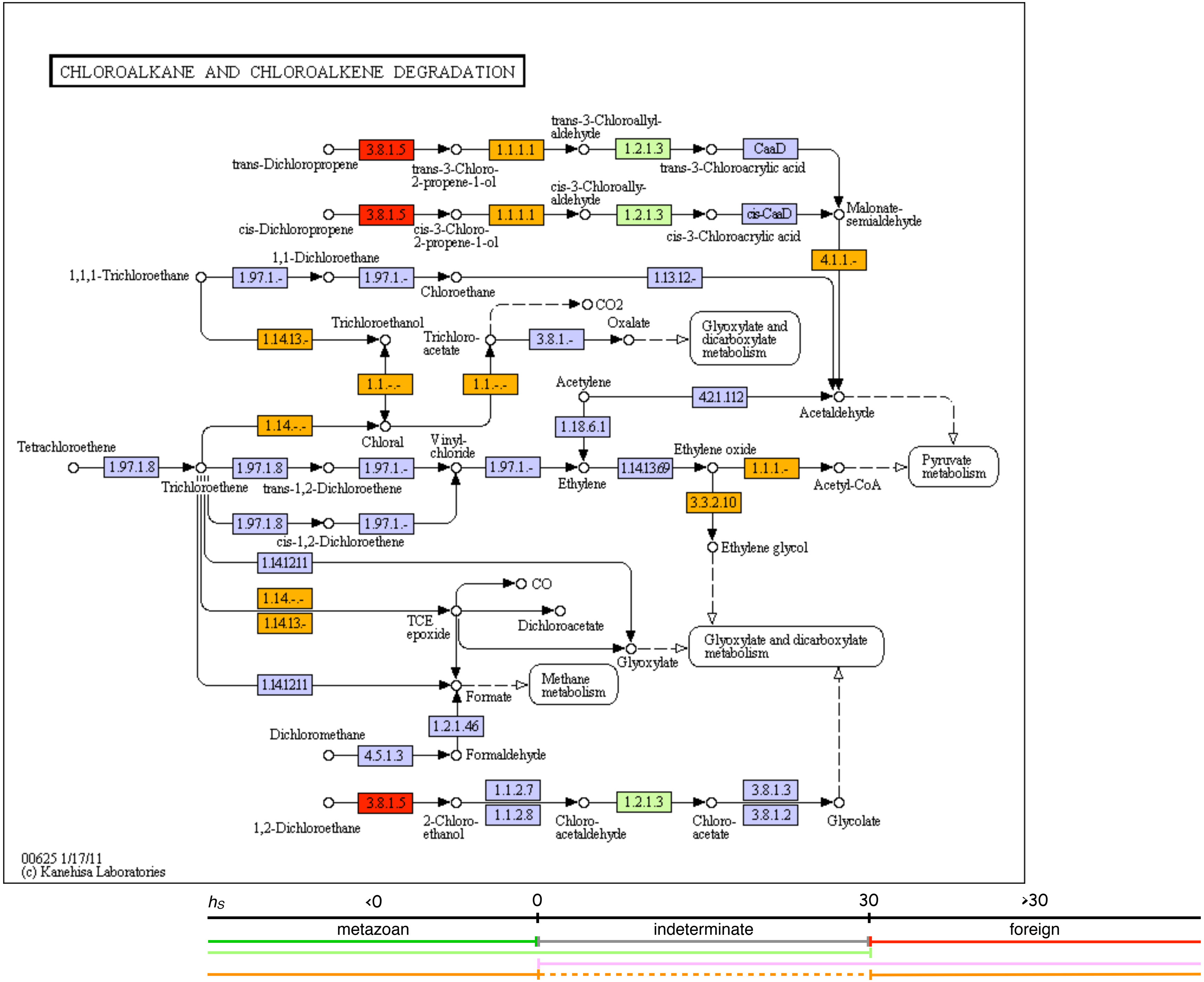

Boschetti Figure S5D

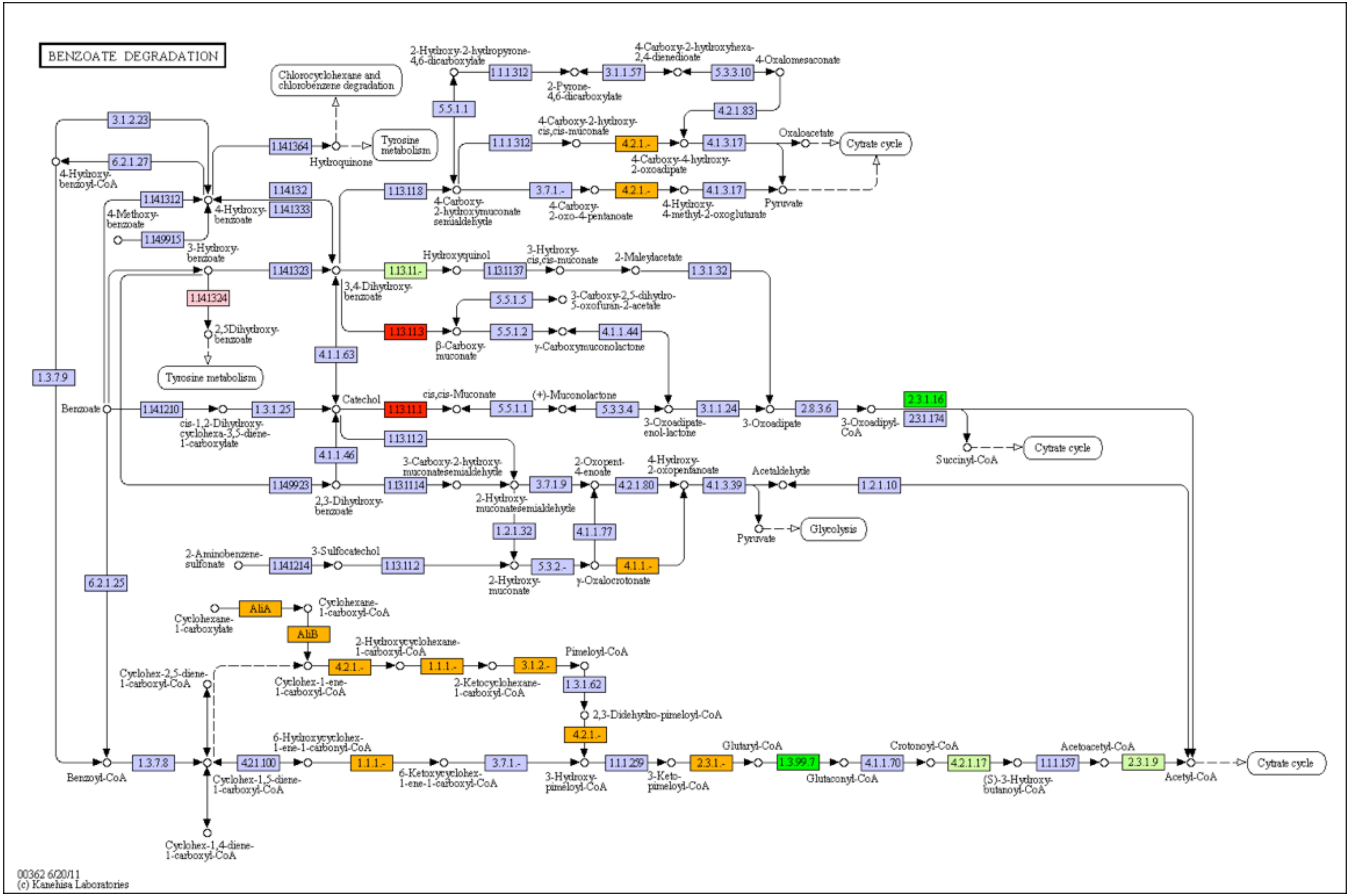

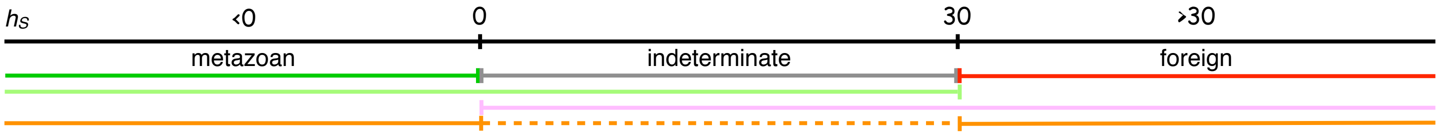

Boschetti Figure S5E

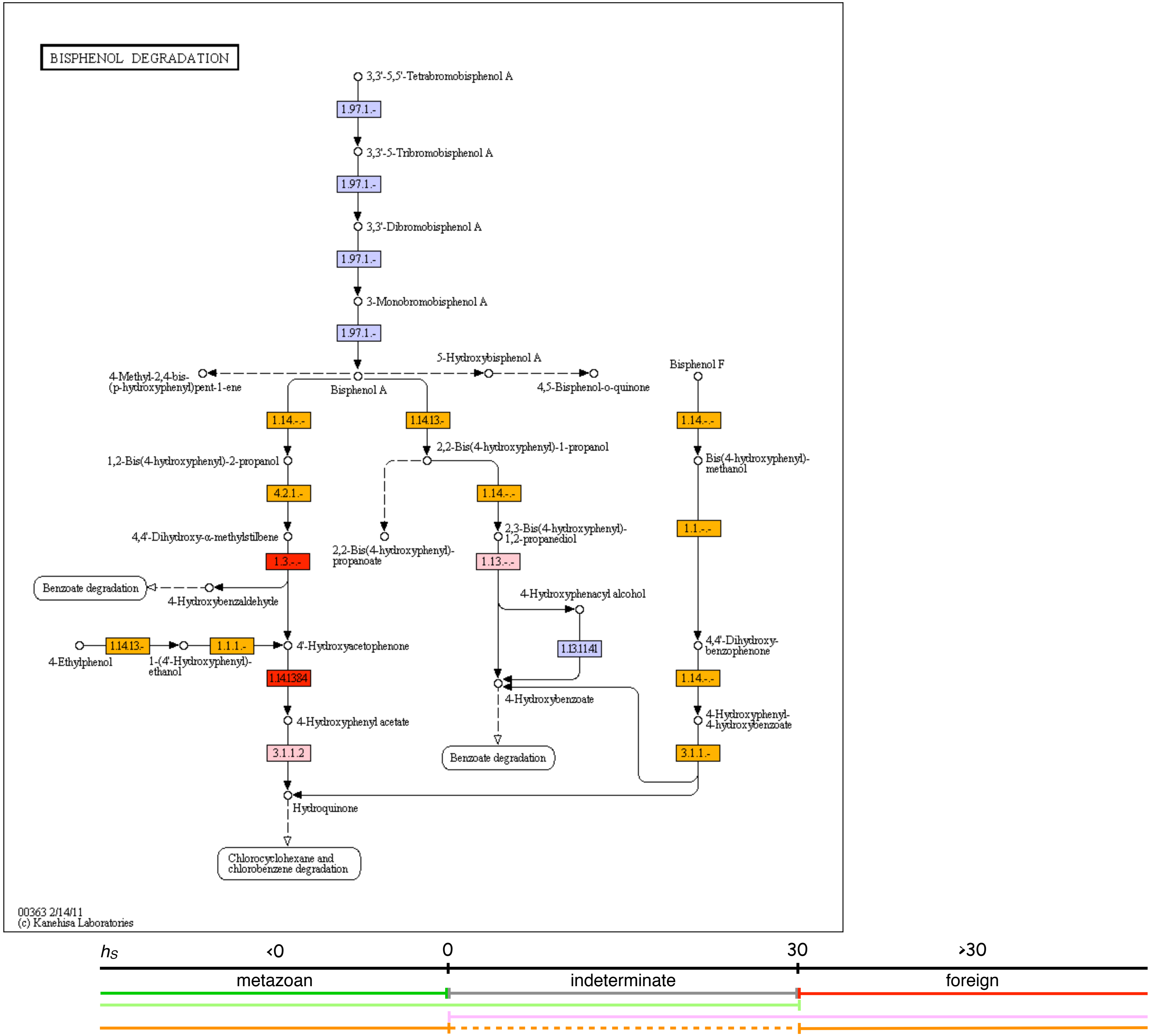

Boschetti Figure S5F

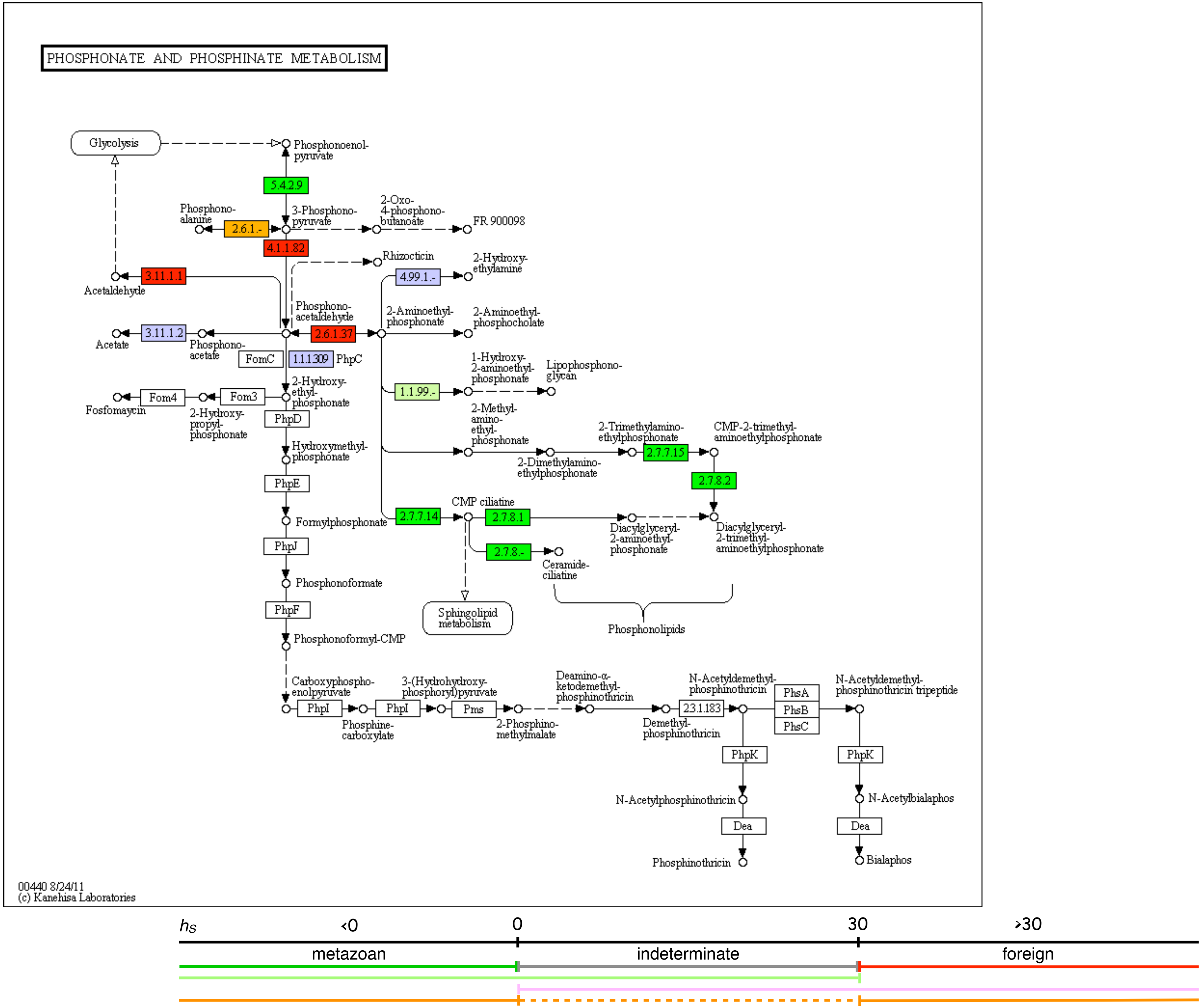

Boschetti Figure S5G

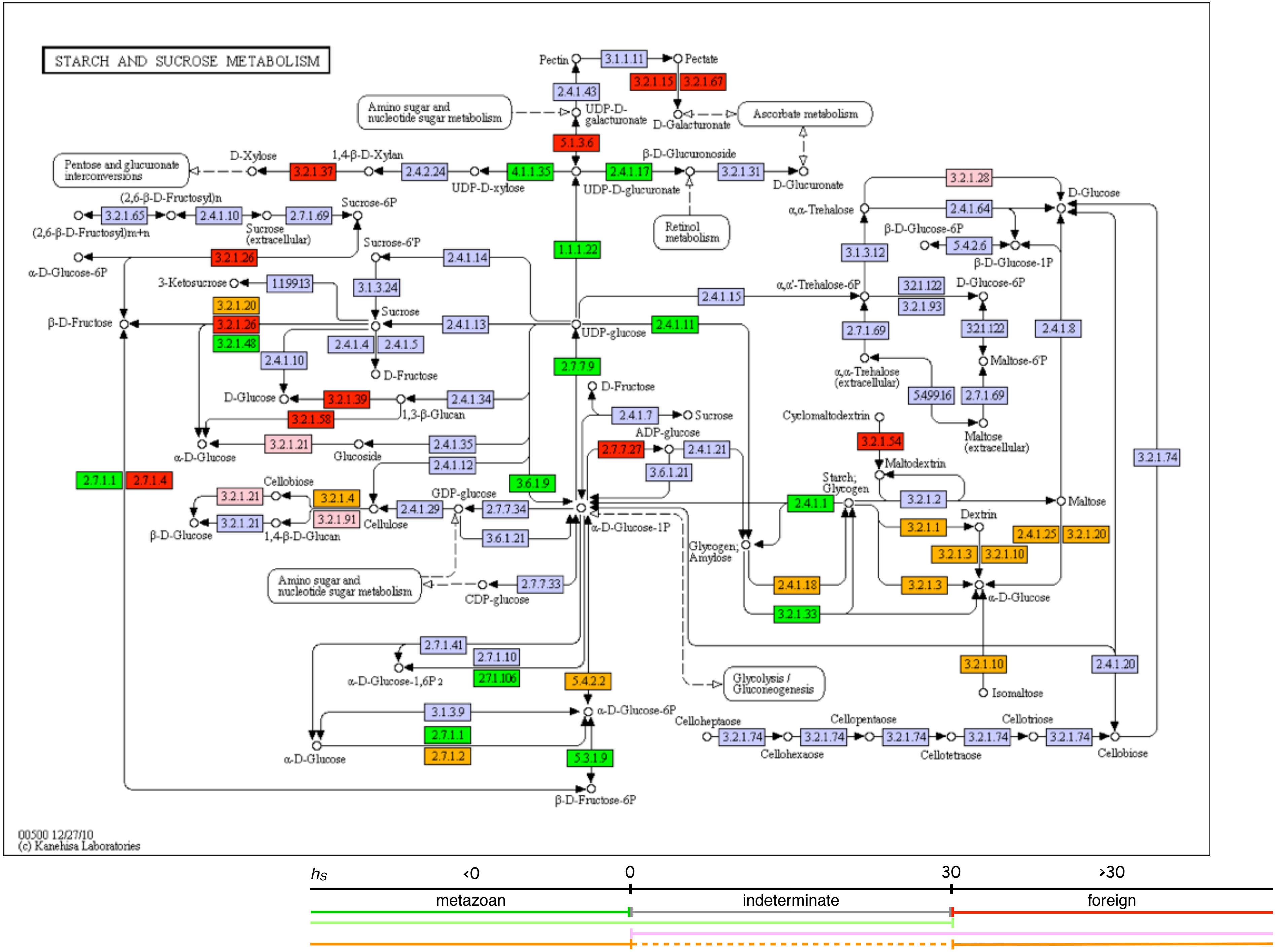

Boschetti Figure S5H

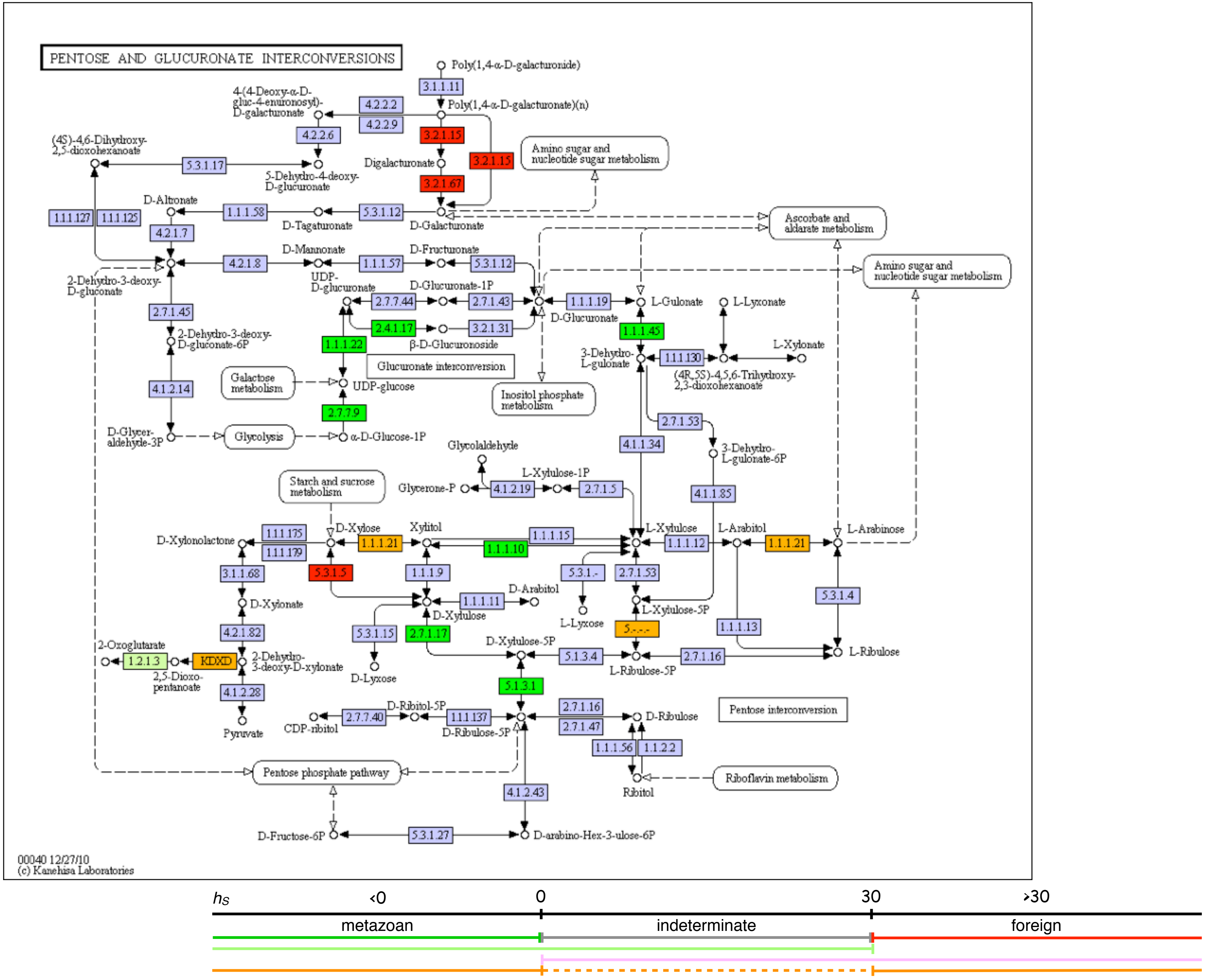

Boschetti Figure S5I

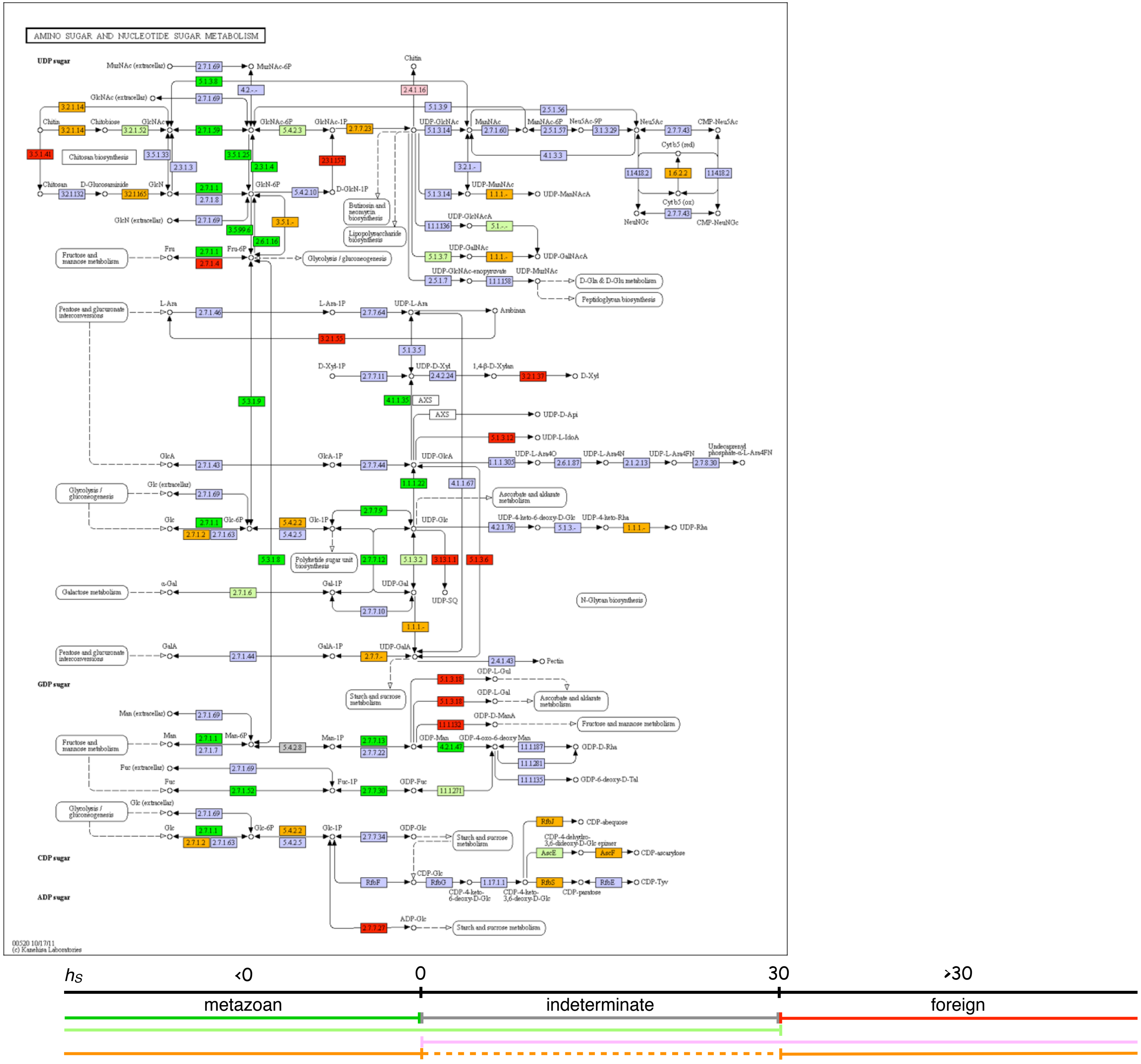

Boschetti Figure S5J

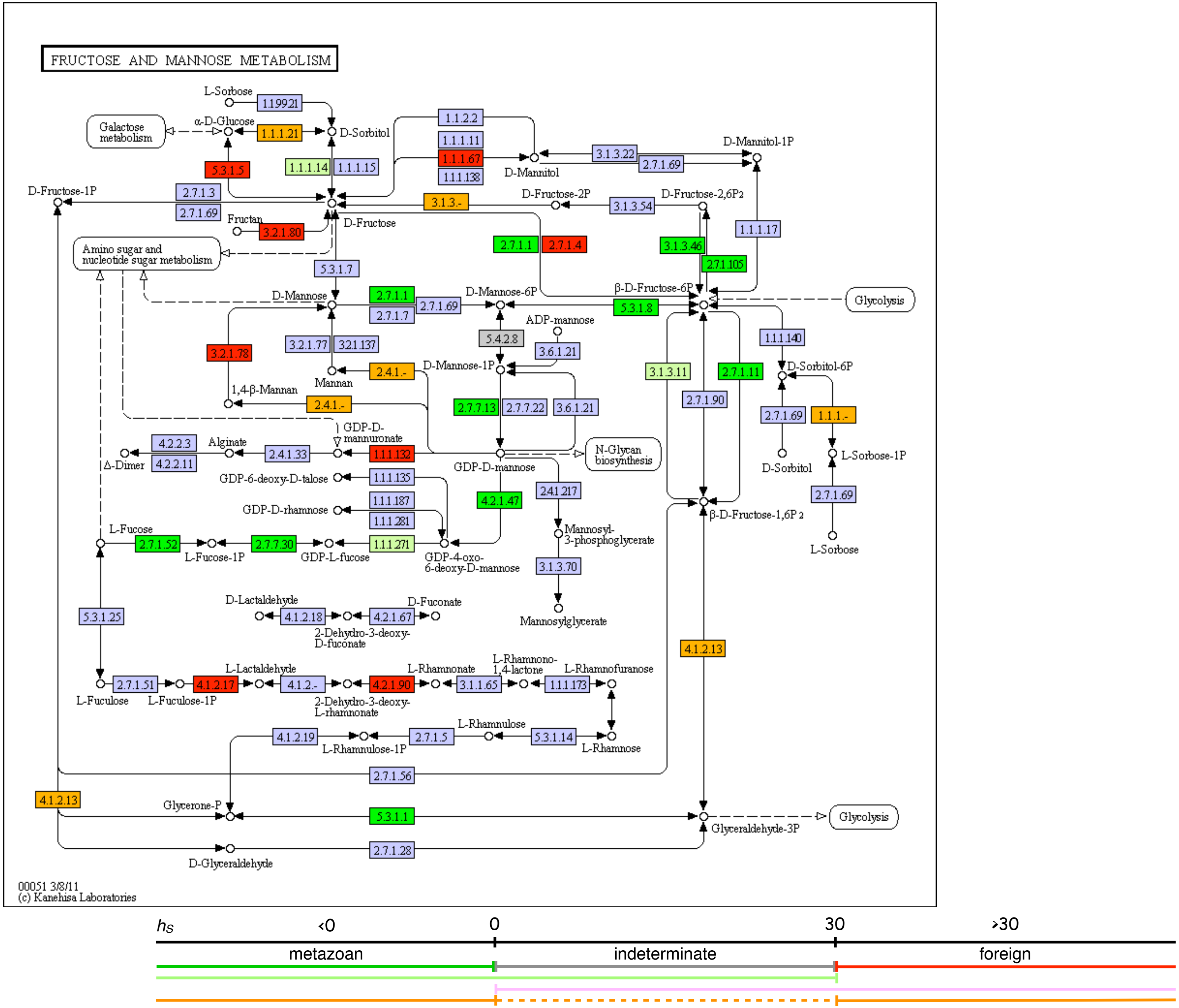

Boschetti Figure S5K

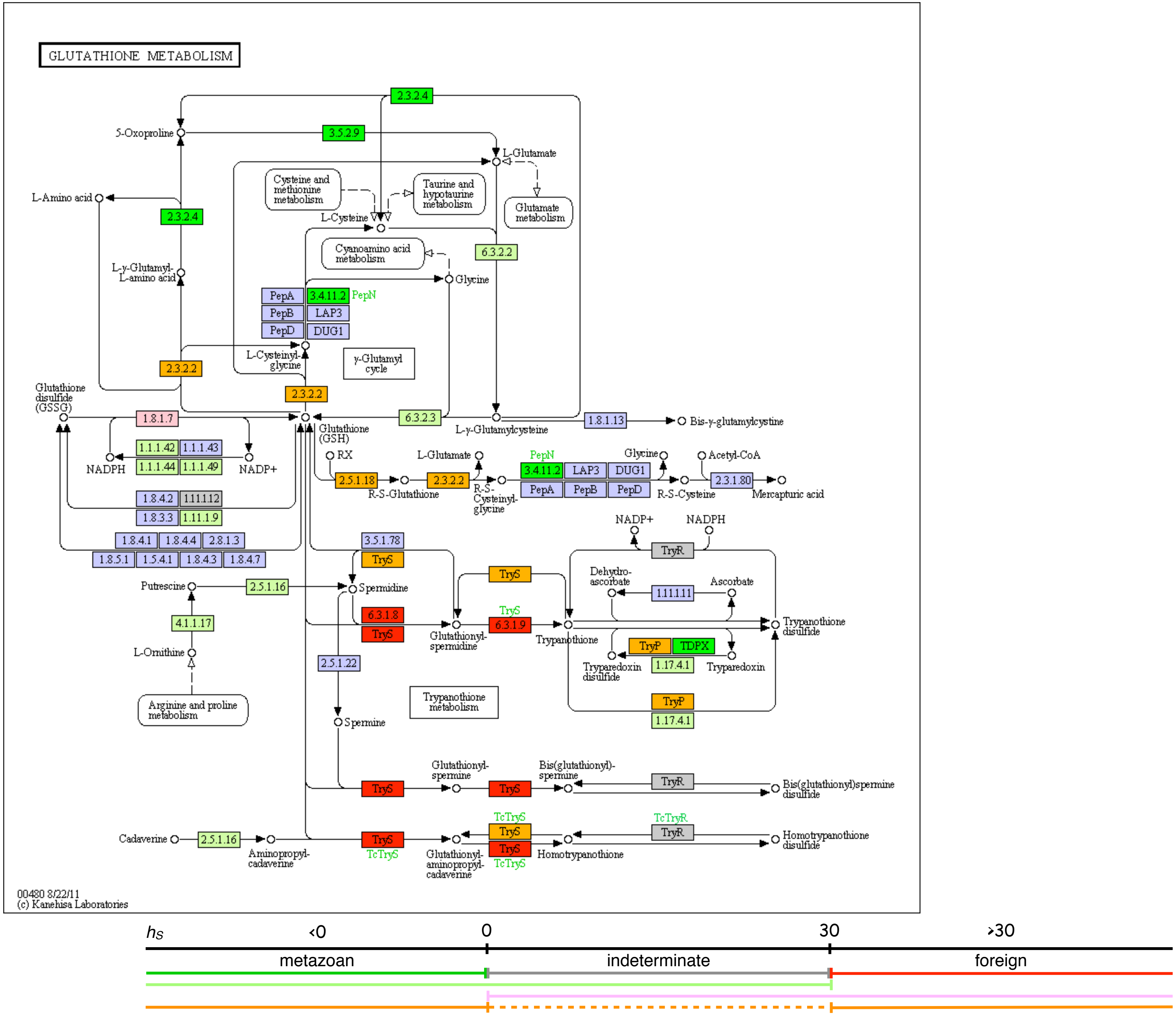

Boschetti Figure S5L

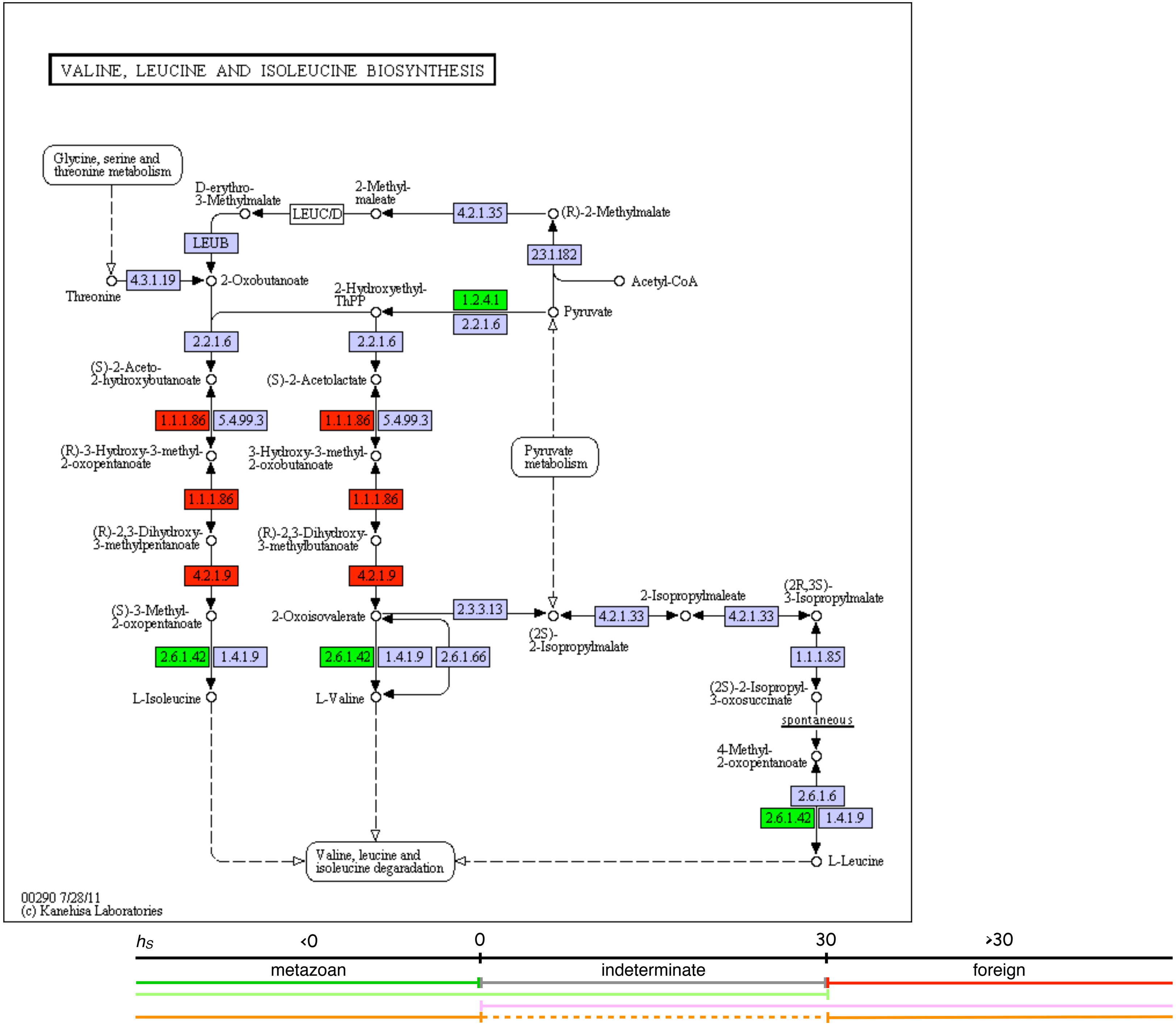

Boschetti Figure S5M

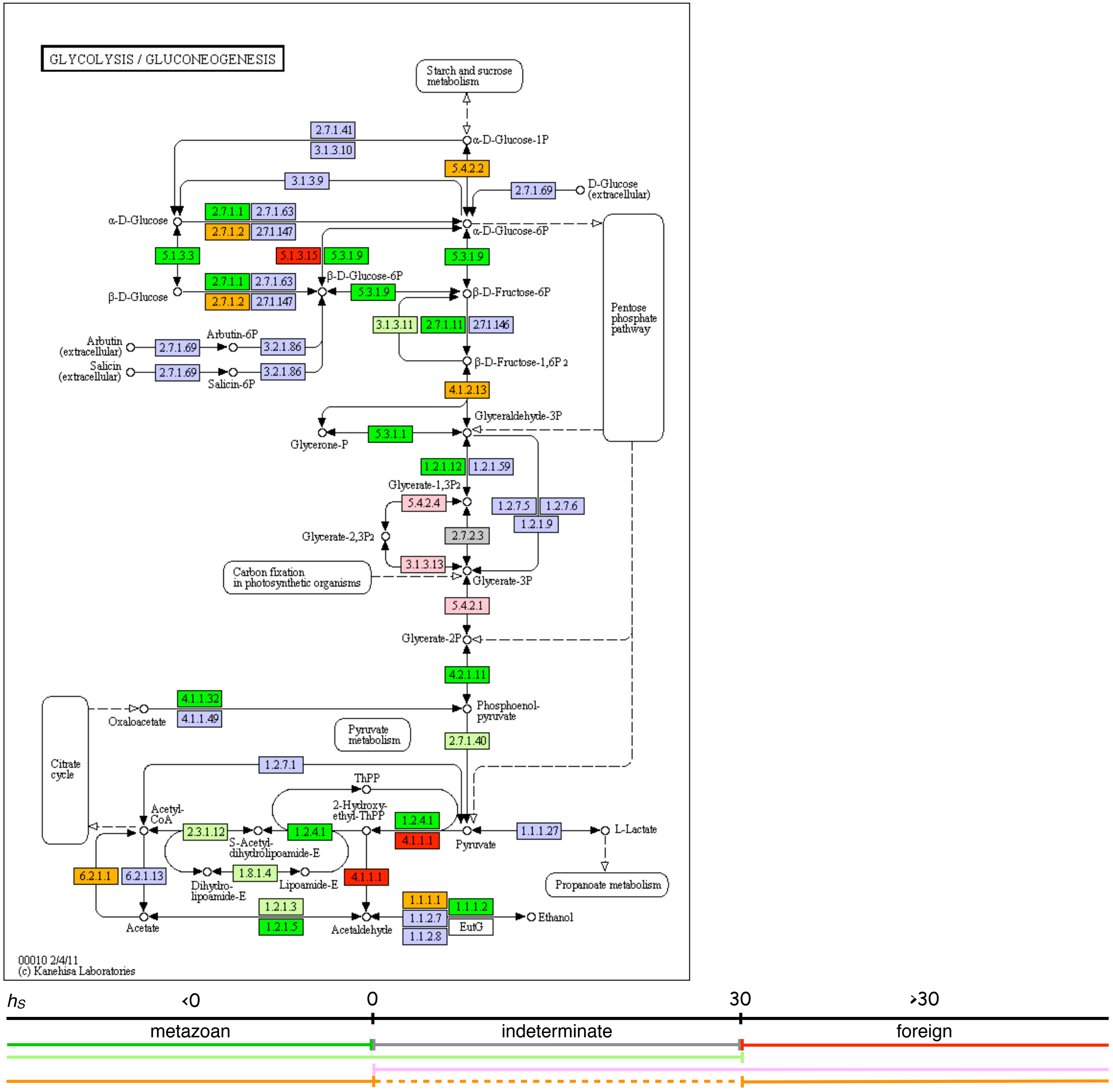

Boschetti Figure S5N

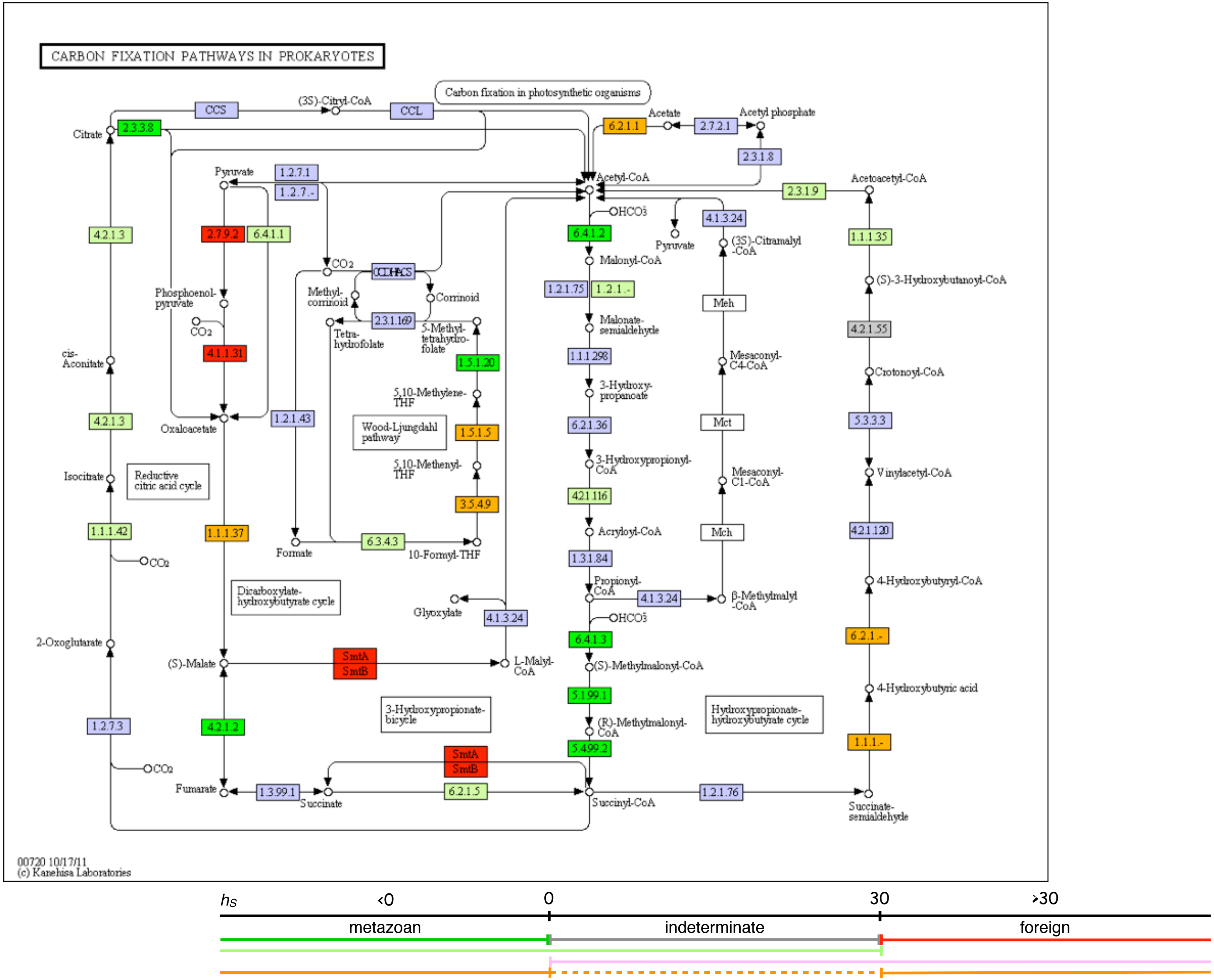

Boschetti Figure S5O

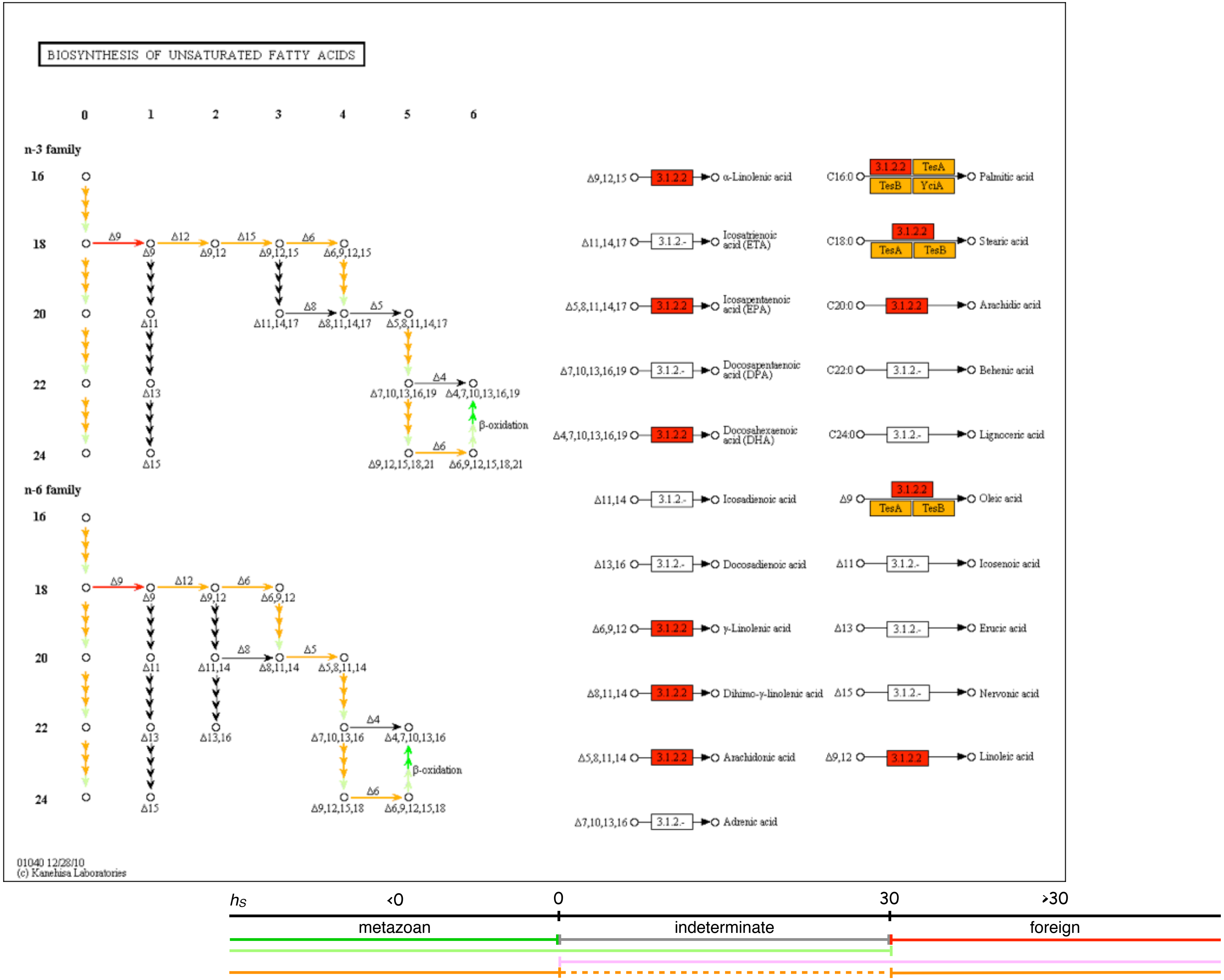

Supplement: Figure S5 — Complete KEGG representation of pathways listed in Figure 3. Colour coding as in Figure 3: green, metazoan; red, foreign; orange, both metazoan and foreign examples identified; pink, both foreign and indeterminate examples; light green, both metazoan and indeterminate examples; blue, not found in transcriptome. (PDF) [file pgen.1003035.s005.pdf]
