# Supplementary material for: Biochemical Diversification through Foreign Gene Expression in Bdelloid Rotifers
Source: PLoS Genet. 2012 Nov 15;8(11):e1003035. doi: 10.1371/journal.pgen.1003035 (PMC3499245; doi:10.1371/journal.pgen.1003035)
Supplement: Table S3 — Mapping of foreign transcript contigs corresponding to enzyme activities in Figure 3 to genomic contigs also containing genes of metazoan origin. (DOC) [file pgen.1003035.s009.doc]

| EC | Transcript contig | *h_s_* | Number of foreign transcripts mapping to same genomic contig | Number of metazoan transcripts mapping to same genomic contig | Example of metazoan transcript on same genomic contig |
| --- | --- | --- | --- | --- | --- |
| 1.1.1.86 | 3890* | 187 | 3 | 79 | 5226 |
| 2.6.1.37 | 28759* | 254.3 | 32 | 334 | 3559 |
| 2.7.9.2 | 18983* | 72.8 | 4 | 24 | 2868 |
| 3.2.1.15 | 27088 | 31.2 | 24 | 130 | 3688 |
| 3.2.1.21 | 29659 | 398 | 23 | 156 | 1349 |
| 3.2.1.67 | 279* | 51.2 | 3 | 4 | 26030 |
| 3.2.1.91 | 40343* | 46.6 | 7 | 89 | 4220 |
| 3.5.1.4 | 42174 | 31.2 | 4 | 21 | 6593 |
| 3.5.5.1 | 24559* | 84.3 | 11 | 195 | 2925 |
| 3.8.1.5 | 57916 | 48.5 | 10 | 16 | 2324 |
| 3.11.1.1 | 3816 | 376 | 14 | 20 | 5539 |
| 4.1.1.1 | 31402 | 224.9 | 22 | 130 | 1336 |
| 4.1.1.31 | 60355* | 123 | 20 | 32 | 9473 |
| 4.1.1.82 | 28603* | 83.6 | 24 | 110 | 472 |
| 4.2.1.9 | 45916 | 254 | 52 | 364 | 398 |
| 4.2.1.84 | 4027* | 158 | 4 | 2 | 32798 |
| 6.3.1.8, 6.3.1.9 | 60721* | 90.9 | 46 | 264 | 22 |

**Table S3.** Mapping of foreign transcript contigs corresponding to enzyme activities in Figure 3 to genomic contigs also containing genes of metazoan origin. *No significant metazoan matches and therefore no phylogenetic tree in Figure S1.
